# Acceptability of advanced Controlled Human Infection Models: The case of a pneumococcal challenge in people living with HIV and mycobacterial challenge with Bacillus Calmette Guerin in Malawi

**DOI:** 10.1371/journal.pgph.0006236

**Published:** 2026-08-18

**Authors:** Anthony E. Chirwa, Charity Gunda, Shalom Songolo, Edna Nsomba, Neema Toto, Lumbani Makhaza, Mphatso Mailboy, Clara Ngoliwa, Linda Chamtunga, Modesta Reuben, Chikondi Chakwiya, Pemphero Liwonde, Andrew Sigoloti, Evaristar Kudowa, Godwin Tembo, Ben Morton, Bridgette Galafa, Faith Thole, Lorensio Chimgoneko, Brenald Dzonzi, Vitumbiko Nkhoma, Edward Mangani, Mphatso Mayuni, Alfred Muyaya, Fanny Kapakasa, Morrison P. Kamanga, Tiyamika Nthandira, Diana Mwaipaya, Nengie Chirwa, Ndaziona P. K. Banda, Marc Y. R. Henrion, Tarsizio Chikaonda, Kondwani C. Jambo, Blessings Kapumba, Stephen B. Gordon, Anna C. T. Gordon, Deborah Nyirenda, on behalf of the Malawi Accelerated Research in Vaccines by Experimental and Laboratory Systems (MARVELS) consortium

**Affiliations:** 1 Malawi- Liverpool Wellcome Programme, Blantyre, Malawi; 2 Liverpool School of Tropical Medicine, Liverpool, United Kingdom; 3 Centre for Inflammation Research, University of Edinburgh, Edinburgh, United Kingdom; 4 University of Bristol, Bristol, United Kingdom; Universidade Catolica Portuguesa, PORTUGAL

## Abstract

Controlled Human Infection Models (CHIM) in which volunteers are experimentally exposed to pathogens are used to study pathogenesis and down select among treatment and vaccine candidates. Scientifically, CHIM studies are best conducted among at-risk populations in which the infection is endemic, and who are likely to benefit from new interventions tested. Ethically, the perspectives of these communities should be carefully considered in designing such research. CHIM studies are often conducted among healthy adults, but we wished to understand study participant’s and community views on conducting CHIM studies in groups other than healthy adults, particularly people living with HIV (PLHIV). We also wished to explore community perceptions of a tuberculosis CHIM (TB CHIM), initially using Bacillus Calmette Guerin (BCG) as a safe intermediate step. We conducted fourteen focus group discussions (FGD) and eight in-depth interviews (IDI) among a wide range of stakeholders including participants, health workers, community advisory groups, religious leaders and medical opinion leaders in Malawi. Discussions and interviews were recorded, transcribed and analysed using thematic and framework analysis. Four themes were described in the data, showing engaged stakeholder acceptance of CHIM studies, and confidence in the research practice and motivation. In the specific examples discussed in this work at-risk populations specifically PLHIV and complex pathogens specifically TB-CHIM, there was concern for safety, proper consent and community consultation. In addition, cultural views on gender roles in Malawi, tissue sampling and residential stays for research study were discussed. Overall, there was support for CHIM among PLHIV, and CHIM working through a BCG CHIM towards safe TB CHIM in future. Appropriate safety caveats and regulatory measures were suggested. Advice on wide, public awareness campaign methods was offered to researchers.

## Introduction

Controlled Human Infection Models (CHIM, also called human infection studies, HIS); involve deliberately introducing an infectious organism in humans to understand disease processes and immune responses and accelerate intervention development [1]. These studies raise important ethical questions for which guidelines have been developed and standardised [2–4]. CHIM studies conducted in line with these guidelines have been utilised as a tool to answer questions on disease acquisition and transmission dynamics and have contributed to vaccine development [5,6]. CHIMs are now widely accepted and have been conducted in both high-income, low disease-burdened settings such as the UK [7], and in low-income high disease burden settings such as Malawi [8]. As confidence in this method of infectious disease research grows, improvement of the models by testing at-risk populations will increase, and the range of pathogens used will expand. This provides an imperative to return to the ethical guidelines and to consult stakeholders in the experimental design.

Human infection studies have progressed to include at-risk populations such as a pneumococcal CHIM in elderly participants in Liverpool, UK [9], and malaria CHIM in Kenyan adults [10]. CHIMs in development include people living with HIV (PLHIV) [11]. HIV infected individuals are at increased risk of infections including pneumococcal disease and exhibit higher residual pneumococcal colonisation than HIV negative individuals [12,13], but PLHIV remain excluded from pneumococcal vaccination programs in sub-Saharan Africa. There is a case for advancing vaccine discovery in PLHIV using CHIM, but the community acceptability of this case has not been explored.

CHIMs targeting vaccine-preventable diseases were developed initially studying bacterial infections with clear rescue strategies [5]. Viral infections without clear treatment protocols have since been studied, including influenza, dengue and SARS-CoV-2 [14]. Among infectious diseases considered for CHIM, tuberculosis stands out as a particularly complex and high-risk candidate. Tuberculosis (TB) has risk and stigma, and persistent infection can be reactivated by immunosuppression in later life. CHIM using attenuated strains such as Bacillus Camille Guerin- (BCG) have been conducted in both tuberculosis endemic and non-endemic settings. These studies have provided useful information towards accelerating tuberculosis vaccine development [15–17]. Mycobacterial models, colloquially described as “TB CHIMs” in this work, have largely been understudied in endemic settings, where populations are most affected and the need for better vaccines beyond the licensed BCG is critical. BCG is the only current mycobacterial CHIM, but the possibility of genetically modified BCG or *M.tuberculosis* being used in future makes “TB CHIM” an appropriate topic for immediate and serious discussion [18]. Importantly, however, community views of such a model in an endemic area have not been explored. The PLHIV community are at high risk of tuberculosis and thus are stakeholders in this discussion, allowing the two topics in this study to be brought together.

In Malawi, the Malawi Accelerated Research in Vaccines by Experimental and Laboratory Systems (MARVELS) consortium has been conducting CHIM research since 2017. We have established a stakeholder engagement strategy to assess the acceptability of advanced CHIMs, called ACHIMA (Advanced CHIM Acceptability). The ACHIMA programme, through public and participant involvement and engagement (PPIE) has proposed ethical and practical considerations for a CHIM in PLHIV, and for a tuberculosis CHIM using BCG [19]. Using a consultative community engagement strategy and experience from an established pneumococcal CHIM methodology, we now present stakeholder views on both the conduct and ethics of the first controlled human infection model in PLHIV and a tuberculosis challenge using BCG in HIV negative adult Malawians.

## Methods

We conducted focus group discussions (FGDs) and in-depth interviews (IDIs) with a wide range of stakeholders to understand community views and to take advice on practical and ethical considerations for CHIMs in an at-risk population of special interest in Malawi - PLHIV. We also engaged these participants to understand community views and to take advice on practical and ethical considerations for CHIM methods being applied to a disease of specific interest in Malawi – tuberculosis. Malawi is a country with 11% of urban adults living with HIV, in which tuberculosis remains a high-burden disease and a national priority.

### Ethics statement

The protocol was reviewed and approved by the College of Medicine Research Ethics Committee at the Kamuzu University of Health Sciences (study reference number P/06/23/0095). Formal written consent was obtained from all participants and no person under the age of 18 years was recruited.

### Participants and recruitment discussions

As part of ongoing community engagement, we conduct CHIM community sensitisation events regularly (3–6 per year) and have recently conducted both live radio and television debates on CHIM topics. For this study, potential FGD and IDI participants who contacted us following advertisement, or who were in contact with MLW public engagement structures as community leaders, professional contacts or patients, or who had previously contacted the group in response to other study advertisements, were offered an information leaflet outlining the study and what participation would involve. The research team contacted the HIV and TB offices in QECH and Blantyre District Health Office to invite nurses, clinicians and health surveillance assistants to take part in focus group discussions. We requested contact of a list of any HIV and former TB patient groups and their guardians who might be interested in participating in the discussions from these facilities. Additionally, leaflets were provided at these facilities. MLW public engagement structures were requested to provide contacts of Community Advisory Groups (CAGs) and the study team requested their participation. Some leaflets were handed out in clinics including the HIV clinic at Queen Elizabeth Central Hospital. We requested participation of any experts in HIV and TB-related services directly for in-depth interviews, including senior doctors at QECH, researchers from private hospitals in Blantyre, and key national ethical and regulatory members in Malawi.

Following full written informed consent, recruitment took place from 14^th^ December 2023 until 30^th^ June 2024. Data collection was based on previous work to include a purposively recruited, maximum variety sample which offered adequate credibility, rather than recruiting to data saturation. The wide variety of cadres necessitated that several groups had a small number of representatives therefore cross-tabulation of themes by stakeholder category was not planned.

Focus group discussions were arranged at a variety of times of day (morning, afternoon, early evening) to accommodate participants and in-depth interviews were arranged at the convenience of the person being interviewed. For each activity, topic guides were developed from our previous experience of engaging CHIM stakeholders before, during and after previous studies. In each activity, we separated time in which the questions were focused on either CHIM in PLHIV or CHIM using mycobacteria (BCG or TB CHIM). We allowed for a rest break of at least 30 minutes in-between topic discussions. We collected data using topic guides. We iteratively amended the guides during the study to explore emerging topics. Each FGD lasted approximately 2 hours, and each IDI about 1 hour. Field notes were collected and kept in a “reflexive diary”. We audio-recorded both FGDs and IDIs, uploaded them on a secure server in REDCap, then transcribed the recordings verbatim. Discussions conducted in Chichewa were transcribed, then translated into English by the MLW translation and transcription team (DM, FK, NC). FGD were conducted separately for men and women from the general community (e.g., healthy adults, or previous CHIM participants) and in mixed groups for professional cadres. FGD were conducted by a team including at least two of a research physician (AC), qualitative researchers (CG and BK) and study nurses (SS and EN). The in-depth interviews were conducted by either the research physician or a female research assistant (CG) with experience in qualitative research who had no previous experience of the interviewed study participants. CG was supported in study design by a senior nurse researcher (NT) and by a senior qualitative research academic (DN).

### Data analysis

Transcripts were organised using NVivo 14 and thematically analysed using thematic and framework analysis methodology with broadly deduced themes (recruitment, practical or study-specific considerations) and sub-themes were inductively derived (CG, ACG) [20]. Consensus was reached on themes by AG and CG during regular analysis meetings, and later by all members of the team.

## Results

### Participants and recruitment discussions

A total of 115 participants were contacted and 114 participated. All participants were able to give written informed consent taken at face to face interview including questions to ensure that they fully understood the study, and all had access to a mobile phone. Demographic details are summarised in Table 1. Fourteen FGDs were carried out with a purposively recruited, maximum variety sample of 106 participants [21]; PLHIV recruited from clinics (9), former TB patients recruited from the TB registry (7), healthy adult community members identified by introduction by members of Community Advisory Groups (CAGs) (19), former participants of previous CHIM studies approached by phone or text message (14), community opinion leaders (local chiefs) (5), religious leaders (6), and CAG members (13) recruited through our ongoing public engagement, HIV care clinicians and nurses (10), tuberculosis care clinicians and nurses (13), and Health Surveillance Assistants (HSAs) specialising in HIV or tuberculosis care (10) recruited through local health services. We conducted separate FGDs for each of these groups to account for possible culturally oriented power dynamics, but we combined men and women in the groups. We also carried out eight IDIs focused on safety and ethical considerations, with HIV clinician scientists (2), tuberculosis clinician scientists (2) and local Research Ethics Committee members (4).

**Table 1:** Participant demographic details.

Demographic characteristics													
Characteristic	Overall	CAG	Community Leader	Community Member	Former CHIM	Former TB patient	HIV care provider	HSA	PLHIV	Policy maker	Religious Leader	Senior Doctor	TB care provider
Number	114	13	5	19	14	7	10	10	9	2	6	6	13
GENDER													
Female	65 (57%)	7	0	13	7	5	3	8	7	1	3	3	8
Male	49 (43%)	6	5	6	7	2	7	2	2	1	3	3	5
AGE^1^	43 (35,49)	48(43,52)	43(43,49)	46(38,67)	29(28,33)	47(45,51)	31(30,40)	47(41,51)	47(45,50)	44(35,53)	54(43,57)	46(36,48)	38(31,39)
INTERVIEW TYPE													
Focus Group Discussion	106	13	5	19	14	7	10	10	9	0	6	0	13
In Depth Interview	8	0	0	0	0	0	0	0	0	2	0	6	0

^1^n (%): Median (Q1, Q3).

### Thematic analysis

Thematic analysis identified four primary themes, and their sub themes as illustrated in Table 2. The primary themes were: (1) General views of CHIMs, (2) CHIM recruitment and consent, (3) CHIM study practical considerations including gender issues (4) Individual CHIM study specific considerations. Each theme, and the evidence to support it, are presented in order.

**Table 2:** Key Themes and sub-themes.

Theme	Sub-themes
Acceptability of advanced CHIMs	Knowledge of research or participation in previous research Recognised clinical need National or community interest
CHIM recruitment factors	Misinformation Community engagement strategies Informed consent and literacy Gender-specific factors and contraception Poverty and financial compensation
CHIM study implementation factors	Access to health care Residential stay – gender roles and community perception
Study specific factors for PLHIV	Vulnerability CD4 count
Study specific factors for TB CHIM	Biopsy and use of tissue Bronchoscopy Familiarity with BCG

#### General views of CHIMs.

**Fig 1:**
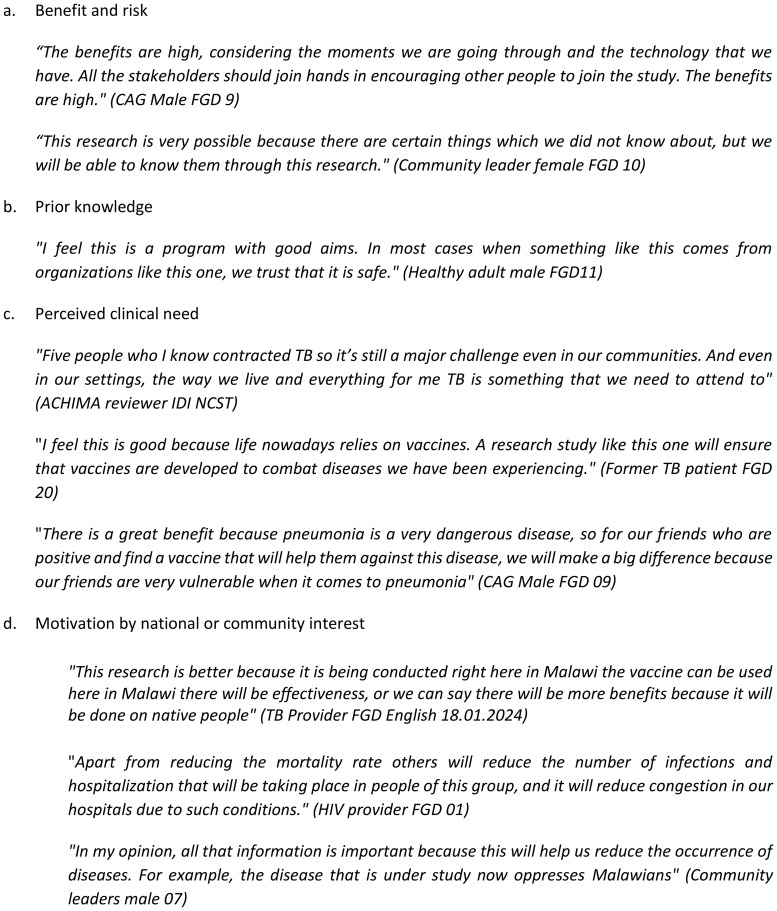
Primary Theme 1 General acceptability of CHIMs.

Participants across the focus group discussions and in-depth interviews understood the scientific potential of CHIM to contribute to clinical research work and development of new drugs and vaccines. Participants were supportive of proposals to conduct CHIM studies in Malawi. Many participants felt the anticipated benefit outweighed the potential risks (Fig 1a).

Participants with prior knowledge or participation in previous research expressed specific confidence in research conducted by the Malawi-Liverpool Wellcome Program, drawing from their prior experience with previous studies including community vaccine studies (community members), surveys and CHIM studies (mostly university students). Prior involvement appeared to build trust in both the research process and outcomes (Fig 1b).

Participants perceived an acute need for improving treatment and prevention of pneumococcal disease among PLHIV. Tuberculosis (TB) was seen as a priority disease for intervention in Malawi and this influenced participants from both focus group and in-depth interviews. Their reflections were focused on personal loss as well as broader national burden. Many felt that CHIM studies were justified due to this clinical need, and research was viewed as one potential solution to health challenges Malawi is facing (Fig 1c).

Participants were motivated by national or community interest and viewed participation in CHIM studies as a personal sacrifice, recognising the benefits of the research to the wider community. Participants believed that CHIM research could strengthen the local health sector and benefit future patients. A strong sense of national pride and belonging underpinned a belief that locally conducted research could foster trust in medication and vaccine development (Fig 1d).

#### Perspectives on CHIM recruitment.

Most participants supported the development of context-specific research volunteer recruitment strategies including briefing community leaders, posting on social media, public events and advertising on local radio and TV. They articulated a strong sense of risk that failure in this domain would make the research impossible and that success in this approach would address cultural and site-specific challenges as well as ensuring that participants’ recruitment was both ethical and safe. The sub-themes discussed were community engagement, literacy, misinformation, gender specific factors, poverty, compensation and incentives to research participation.

**Fig 2:**
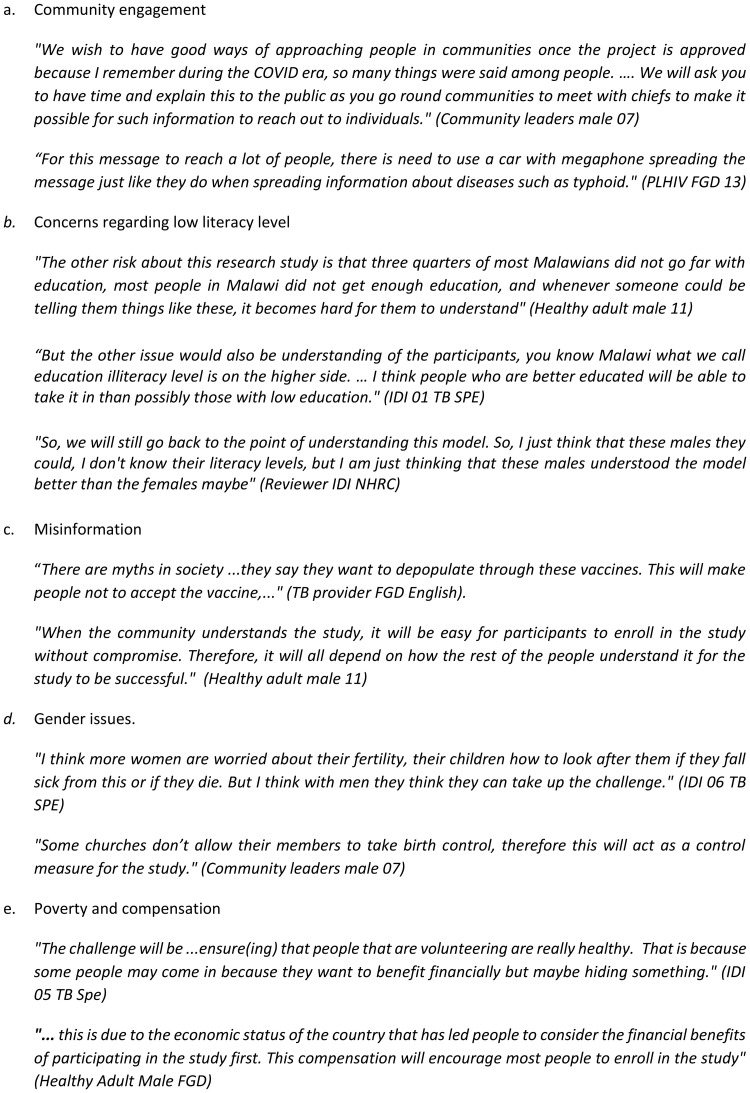
Evidence in Primary Theme 2: Malawi-specific CHIM study volunteer recruitment factors.

Participants highlighted the importance of mass awareness campaigns stating that effective communication efforts could contribute significantly to the likelihood of CHIM studies being well received and accepted by community members. Stakeholders recommended use of mass communication channels such as megaphones and radio adverts as effective tools for broad community outreach alongside partnerships between trusted community figures and research outreach workers (Fig 2a).

Concerns were raised by participants regarding low literacy levels among the community, and the complexity of CHIM studies which may pose challenges in obtaining fully informed consent. Both FGD and IDI participants noted that recruitment might be more successful among individuals with at least a secondary education, and potentially favour men, as they were perceived to have higher literacy levels. Participants in focus group discussions, however, expressed a good understanding of CHIM in PLHIV, or CHIM using mycobacteria, and agreed that while low literacy might be a challenge, this did not constitute a complete barrier to participation (Fig 2b).

Misinformation was widely discussed as a significant barrier to recruitment and participants discussed experiences in the COVID pandemic and subsequent vaccine roll out, including rumours that the vaccination was a population control measure being inflicted upon Malawian people. Some rumours originated from social media (rumours of control) while others were more local (vaccination can lead to cholera). Community members understood the impacts of persistent misinformation on both direct study recruitment and the risks of community misunderstanding (Fig 2c).

Participants anticipated that recruitment would be more challenging for women than men. Key concerns were the potential impact of study participation on childcare responsibilities, and the study requirement for contraception during study period (agreement to use contraception is an inclusion criterion in CHIM studies). Participants agreed that women enrolled in CHIM studies should have a contraceptive plan in place for the study duration viewing this as a necessary safety measure. Male contraception was not discussed as the groups did not perceive a specific risk to males or their unborn offspring associated with procreation during CHIM participation (Fig 2d).

Participants highlighted the complexity of determining appropriate compensation in a resource-poor context. Participants stated that compensation decisions should be influenced by perceived risk factors, family well-being and cultural expectations. Participants recognised that Malawi’s ongoing economic hardships further complicate the assessment of what constitutes fair and ethical compensation (Fig 2e).

#### Practical considerations for implementing CHIM studies.

Adapting CHIM studies to ensure alignment with the socio-economic and cultural context in Malawi was an important consideration found in both IDIs and in FGDs. Participants raised significant concerns regarding limited access to medical care in Malawi and how that would influence CHIM study implementation. They also discussed residential participation in the study, gender issues related to residential stay, and community perceptions of residential stay.

**Fig 3:**
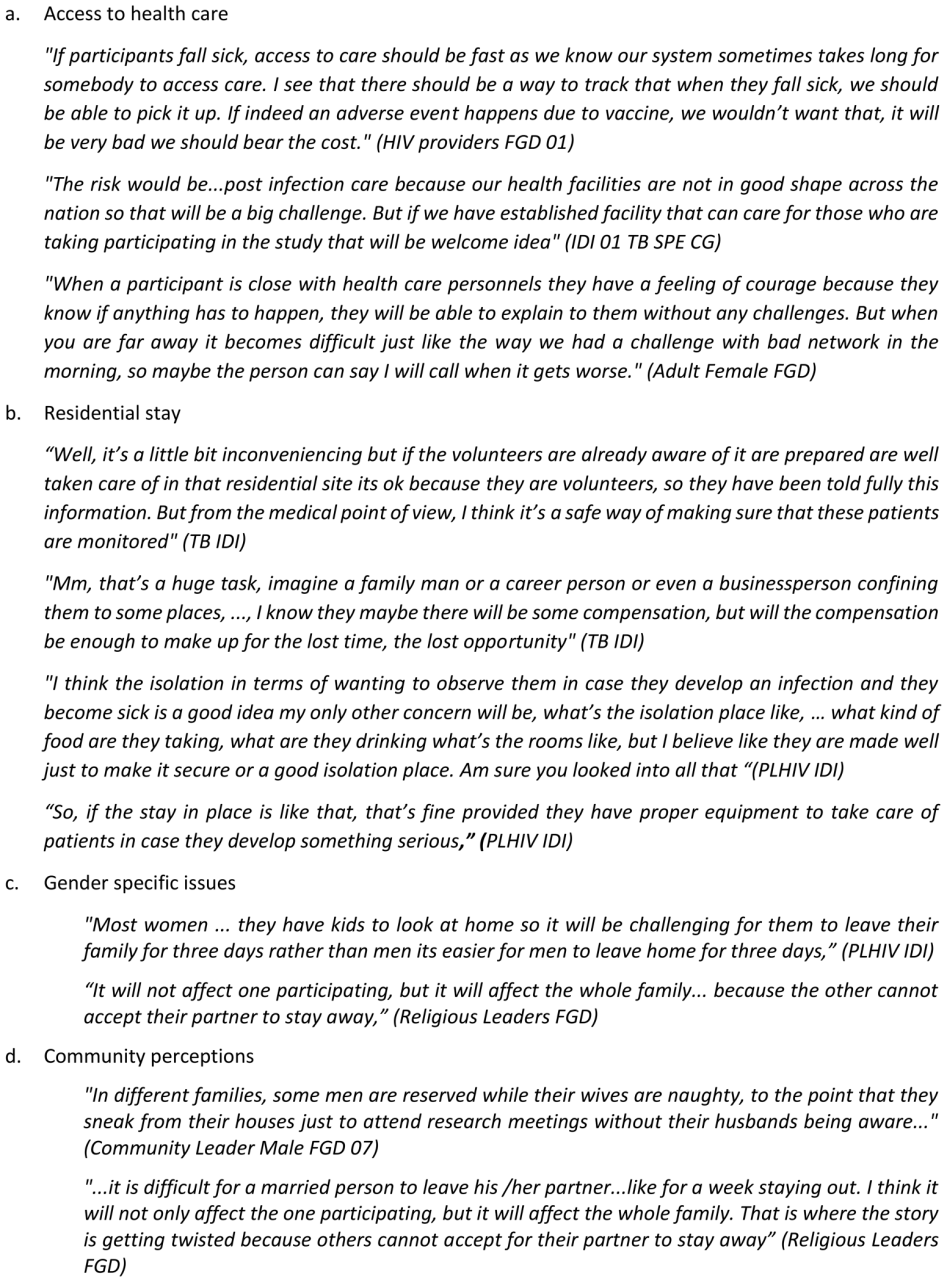
Primary Theme 3: Malawi-specific CHIM study implementation factors.

Participants emphasized the importance of ensuring that individuals taking part in a CHIM study could access quality health care both during and after the study period. They warned about the important need for clear and comprehensive health care provision as part of study design to ensure participants’ safety and ethical standards. Participants recommended that health care support should not be limited to study period but should safeguard long-term well-being of participants (Fig 3a).

Residential stay in a conference centre to ensure easy access to medical support, but not constituting inpatient medical supervision, was supported by most participants. This could also be viewed as an inconvenience, however, leading to loss of income during the stay. To address the concerns raised participants emphasized the need for the study site to be adequately equipped and include provision of essential needs including food and water to support participants’ well-being during the stay (Fig 3b).

Gender specific issues discussions were in relation to residential stay in the context of childcare roles and family life acting as a barrier to participation for female participants particularly married ones. This was not felt for male participants (Fig 3c).

Participants expressed concern that community perceptions might result in reputational damage to individuals, especially women, who would stay away from home for study activities. They noted it would be difficult for participants to enroll in the study without the wider community being aware of leading to stigma or gossip. Participants emphasized the importance of strong and transparent communication to build understanding and promote acceptance of study (Fig 3d).

#### Perspectives of participants specifically on Pneumococcal CHIM in PLHIV or TB CHIM.

Two specific conversations were conducted with each IDI and FGD. The first topic was discussion of a CHIM to develop pneumococcal carriage in PLHIV. The second topic was a CHIM to study immunological responses to either skin or lung BCG instillation, intended in HIV negative healthy adults. Participant concerns were separately discussed in the context of conducting a Pneumococcal CHIM in PLHIV or a TB CHIM in healthy adults.

**Fig 4:**
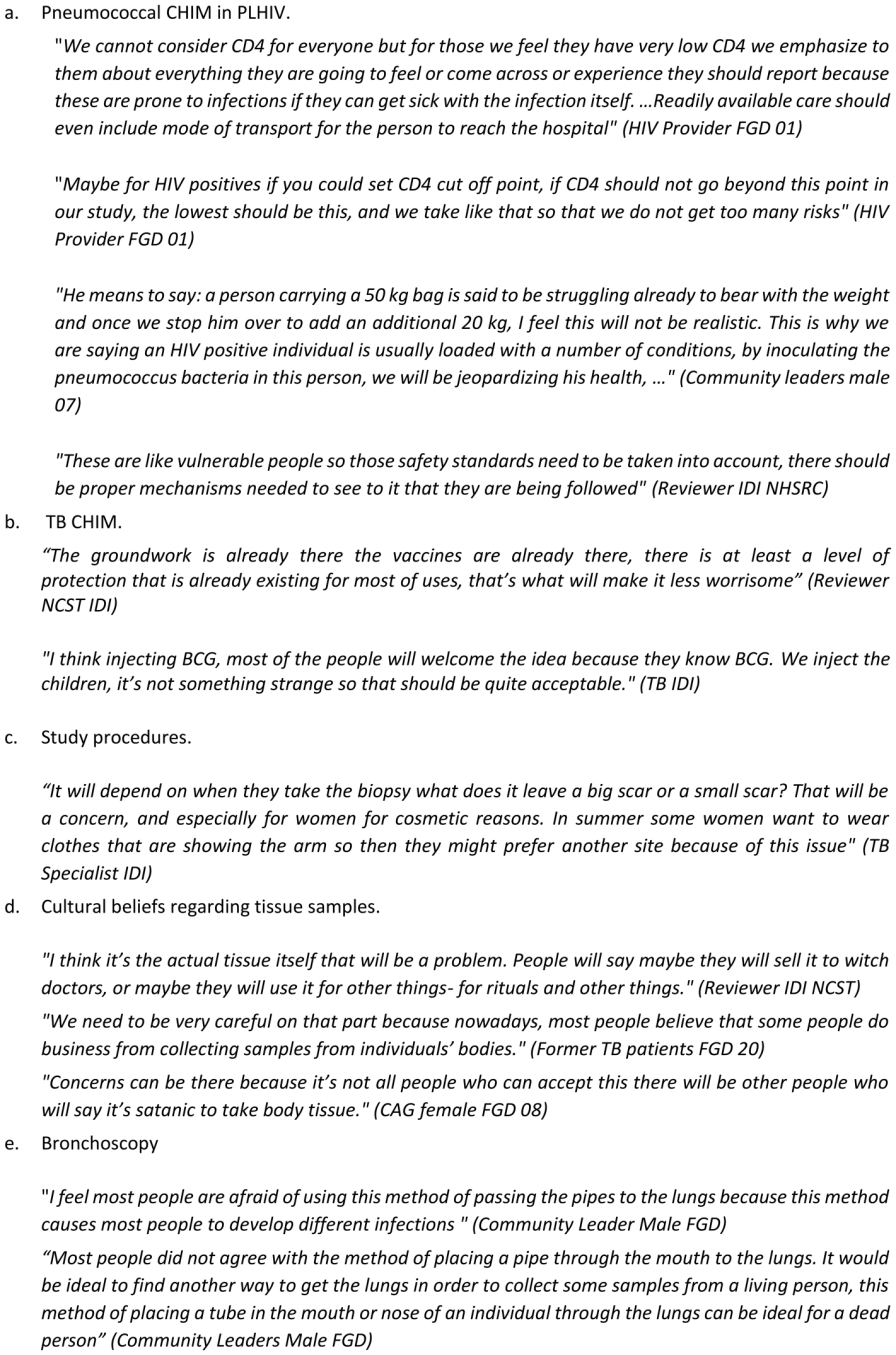
Primary Theme 4: Individual CHIM study specific considerations.

Regarding the pneumococcal CHIM in PLHIV, participants expressed concern about the clinical vulnerability of PLHIV, recognizing them as being at high risk of infections. They emphasized the need for caution when considering the inclusion of this group in CHIM studies. Suggestions were made to ensure that the study protocol adequately addresses these risks, including thorough health assessments based on CD4 count to ensure that only healthy individuals were enrolled to minimize risks (Fig 4a).

In contrast, there was broad support for the TB CHIM from stakeholders, largely due to familiarity with the BCG vaccine which was proposed as the infectious agent under study. BCG is well known as part of the routine childhood immunization programme in Malawi. Additionally, stakeholders recognized the significant burden TB places on the Malawian population and expressed hope that such studies could contribute to reducing TB cases in the long term (Fig 4b).

Participants expressed specific reservations about certain study procedures in the proposed BCG CHIM. The skin biopsy procedure to determine infectious load raised concerns related to cosmetic outcomes—especially among women. Participants associated the biopsy with potential pain, discomfort, and the possibility of scarring. Additionally, there were worries about delayed healing and the risk of infection at the biopsy site, which contributed to hesitancy about this aspect of the study (Fig 4c).

In addition to physical concerns there were notable fears rooted in cultural beliefs and misinformation. Some participants associated the collection of human tissue with local suspicions about its possible use in rituals or harmful practices. These misconceptions contributed to anxiety and mistrust around biopsy procedures. Participants emphasized the importance of transparent communication about the purpose of tissue collection, and how samples would be handled after use. Clear messaging on these points was seen as critical to optimise community trust and acceptance of study procedures (Fig 4d).

Bronchoscopy and bronchoalveolar lavage were discussed as a means of evaluating lung immunity. This was viewed by participants as being particularly invasive as a research procedure in the absence of a clinical indication. Many expressed concerns about the potential risk associated with the procedure, including infection, discomfort, and the possibility of complications (Fig 4e).

## Discussion

This study distilled topics and opinions found among a large, diverse group of Malawian stakeholders regarding CHIM studies at a time when studies either recruiting at-risk participants or trialling more complex pathogens are being developed. There were both expected and unexpected findings in each of the four primary themes that resulted from thematic analysis.

A strength of this study is its inclusivity of a broad range of study participants from different backgrounds increasing its transferability. Some of our study participants had prior knowledge and experience of MLW, while others had none. Some had experience of CHIM, and others had none. Participants were recruited from the health sector, community leadership, religious groups and policy makers. Additionally, findings were subject to rigorous interpretive challenge during analysis. The study was designed by Malawian researchers with experience in human challenge experiments, interviews were led by a research assistant (CG) not directly involved in the conduct of CHIM, analysis was led by an “outsider” not involved in any of our work (AG), the combination of which allowed for greater reflexivity.

This study had limitations which included the inherent weakness of focus group discussions as they lack individuality of voices, and our in-depth interviews were exclusively with experts. Additionally, the channels we have used to attract participants may have introduced selection bias, and social desirability may have influenced participants responses to questions and discussion topics. We have adopted a mixed-gender design in some FGD and may have collected subtly different responses had we segregated by gender. In many settings, including a low-income setting like ours, it is likely that study compensation influenced participation, and the success of current ongoing studies may have influenced public opinion. Finally, the observed confidence in the MLW brand discussed below may also have influenced opinion, and so further work might be designed to address these several issues. In such new work, we would like to reduce the heterogeneity of the dataset by focusing on specific groups and topics.

CHIM studies have been conducted in Malawi for 7 years, so a level of acceptance was expected, particularly among those with direct experience of the programme [22,23]. An unexpected finding was the trust placed in the MLW programme, and this mirrors data from Kenya where the KEMRI/Oxford brand is very strong [24]. The positive opinion of the research programme contrasted starkly with opinion regarding local government health services. This resonated with the authors experience and current reporting in the Malawi press about public health services. Discussion of the motivation of volunteers resonates with experience from other parts of the world, including the UK during the COVID pandemic when the SARS-CoV CHIM was developed [25].

The importance of public awareness expressed aligned with our previous work showing that recruitment is mainly from word of mouth [26], possibly supported by community information programmes. We use radio phone-in programmes, live TV debate and public meetings to disseminate information, but most volunteers present after contact with friends who are previous volunteers. It is very important to understand that our study participants were discussing the ethics of conducting CHIM in general and not whether they would personally participate in a CHIM study. There was a clear understanding that CHIM volunteers should make their own decisions, and at no point were discussion participants asked if they would personally participate in such a study. In most communities (the authors have experience of London, Sheffield, Liverpool, Oxford and Malawi), participants are a small minority, often found among students, and their decision making is often informed by community opinion.

Comprehension is important to facilitate informed consenting in CHIM studies. Our local ethics review board requires that recruitment should be conducted in either English (often the preferred language of those with secondary education) or Chichewa, the national language. Limited community understanding and misinformation led to low COVID vaccine uptake in Malawi (11%) but established infant vaccination programmes are trusted and have more than 90% coverage. This highlights the value of this study to understand nuanced participants perceptions and improve understanding with informed voluntary participation in CHIM studies.

FGD participants expected that women might be less likely to recruit than men. Our lived experience is that the opposite is true which may be due to the age demographic (university students) of many of our participants making it unusual for us to recruit women with caring responsibilities. In our published CHIM studies testing pneumococcal vaccines, more women than men were recruited in Malawi, but more men in Liverpool, UK. Our early feasibility work recruiting PLHIV from anti-retroviral therapy (ART) clinics found that 75% of recruited volunteers were women. We attribute some of this to the reluctance of men to attend clinics as is well-recognised in the health system but even using other recruitment settings, it has proved challenging to recruit men [27]. This gender imbalance highlight the need to further explore context-specific recruitment strategies targeting men to ensure inclusive participation in CHIM studies.

Appropriate compensation for research participation is very important. We have published specifically on this topic and use our own local guideline when estimating compensation for out-of-pocket expenses, travel, loss of earnings and discomfort [28]. There is no perfect solution to this challenge, as the structural challenges in the Malawi health system are beyond our control. It is generally accepted, however, that it is better to conduct excellent research in low resource settings than to exclude these populations from scientific enquiry [29]. We ensure that our remuneration is optimised by frequent review of compensation offered, and rigorous screening to avoid over-recruitment. The introduction of biometrics for identification is helpful in preventing over-volunteering. Thorough community engagement, transparent communication and culturally sensitive implementation are also essential to improve community understanding of biometrics as a protective measure rather than a threat. In exit surveys, we find that most participants are satisfied, and some always report that the remuneration should be increased; this balance of opinions also provides some reassurance of fairness and good practice.

Limited access to health care in the community has once again been reported as a motivation for participation in health research in Malawi. We first described this for studies involving bronchoscopy over 20 years ago [30]. Despite the relative abundance of doctors graduating from local medical schools, health care provision has not kept pace with population growth and access to health care remains a pressing problem in Malawi. In subsequent ethics workshops [28] and specific project discussions, local opinion leaders have concluded that it is better to offer excellent research projects, carefully governed by national regulators, rather than to exclude all research in any context where local community health care provision is scarce. In this context, it is therefore essential to address any therapeutic misconceptions and ensure voluntary informed participation in CHIM studies. In this way, the individual’s autonomy and the community justice principles can be supported to promote public health.

Residential care for the period immediately following experimental inoculation is reassuring for participants, staff team and regulators in our context. Some concerns were raised in focus groups regarding community perceptions about this residential approach, particularly regarding reputational damage for women. Our experience, however, has been that the feedback from participants in pneumococcal challenge studies, and early CHIM studies recruiting PLHIV, has been universally positive. We have very recently [31] started piloting BCG CHIM which is conducted as a solely outpatient study.

Community perceptions on PLHIV participation in CHIM in a context where 11% of adults are taking ART were critical to this study. Clearly the message regarding good viral suppression and healthy living with HIV have not turned the community dialogue away from the immediate association of HIV infection with AIDS that was so prevalent 20 years ago [31]. This may reflect the lived reality of our participants in a community where many people still die of HIV related diseases. It is of course appropriate that our current and planned studies align with the expectations of the focus group participants that CD4 count/viral suppression are necessary pre-requisites of entry into these studies.

The participants enthusiasm for tuberculosis research was a clear finding in this study. tuberculosis is a feared disease in Malawi, and the failure of BCG to protect adults is widely understood. It is therefore important to recognise this community priority and conduct research on tuberculosis vaccines that may protect adults. The implementation of BCG-CHIM studies is therefore essential to accelerate the development of tuberculosis vaccines that not only safeguard adult populations but also help mitigate the emergence of drug-resistant tuberculosis over time.

Specific concerns about biopsy, tissue collection and bronchoscopy were noted, along with concern about scarring. There was reporting of community superstition about tissue misappropriation in discussions. The concern about bronchoscopy was very useful as our programme has a 28-year history of safe research bronchoscopy spanning many projects and feedback has been positive, including many repeat volunteers [30]. Despite this long record, clearly these procedures are source of community concern and additional community discussion specifically about bronchoscopy safety could be of value in the community engagement programme.

In summary, this extensive consultation exercise has reported engaged stakeholder support for locally relevant research, along with raising concerns about recruitment, safety, consent, information, and remuneration in CHIM. The participants were able to confirm a respect for volunteering in research based on community benefit, even when the studies suggested involved both participants with increased susceptibility to infection, and pathogens of special interest.

## Supporting information

S1 Data All data files. (ZIP)
