# Supplementary material for: Acceptability of advanced Controlled Human Infection Models: The case of a pneumococcal challenge in people living with HIV and mycobacterial challenge with Bacillus Calmette Guerin in Malawi
Source: PLOS Glob Public Health. 2026 Aug 18;6(8):e0006236. doi: 10.1371/journal.pgph.0006236 (PMC13485124; doi:10.1371/journal.pgph.0006236)
Supplement: Supplementary file 1 [file pgph.0006236.s001.zip › Documents/ACHIMA Findings summary v3.pdf]

ACHIMA FINDINGS

Charity Gunda

Anna Gordon

Shalom Songolo

METHODS

- 14 Focus group discussions
- 8 IDI's
- All FGDs and IDIs were audio recorded and transcribe verbatim and translated into English.
- Data were organised using NVivo and iteratively coded with broadly deduced themes

(recruitment, practical or study considerations) with inductively derived sub-themes

DEMOGRAPHICS

Characteristic	CATEGORY												
	Overall N = 114[1]	CAG N = 13[1]	Community leader N = 5[1]	Community member N = 19[1]	Former CHIM N = 14[1]	Former TB patient N = 7[1]	HIV Care Provider N = 10[1]	HSA N = 10[1]	PLHIV N = 9[1]	Policy maker NCST N = 2[1]	Religious Leader N = 6[1]	Senior Doctor N = 6[1]	TB Care provider N = 13[1]
**GENDER**													
Female	65	7	0	13	7	5	3	8	7	1	3	3	8
Male	49	6	5	6	7	2	7	2	2	1	3	3	5
**AGE**	43 (35, 49)	48 (43, 52)	43 (43, 49)	46 (38, 67)	29 (28, 33)	47 (45, 51)	31 (30, 40)	47 (41, 51)	47 (45, 50)	44 (35, 53)	54 (43, 57)	46 (36, 48)	38 (31, 39)
**INTERVIEW_TYPE**													
FGD	106	13	5	19	14	7	10	10	9	0	6	0	13
IDI	8	0	0	0	0	0	0	0	0	2	0	6	0

[1] n; Median (Q1, Q3)

KEY THEMES

- **Acceptability of ACHIMs**
  - Knowledge or participation in previous research
  - Recognized clinical need
  - National / community interest
- **Malawi-specific recruitment factors**
  - Misinformation
  - Community engagement strategies
  - Informed consent and literacy
  - Gender-specific factors & contraception
  - Poverty and financial compensation
- **Malawi-specific practical considerations**
  - Access to healthcare
  - Residential stay
    - Gender differences
    - Community perception
- **Study-specific considerations**
  - TB ACHIM
    - Screening
    - Biopsy
    - Bronchoscopy
    - BCG familiarity
  - PLHIV ACHIM
    - Vulnerability
    - CD4

ACCEPTABILITY OF ACHIMS

Participants across all IDIs and FGs generally agreed that overall, both ACHIMS studies were acceptable, and that potential benefits outweighed risks.

"The benefits are high, considering the moments we are going through and the technology that we have. All the stakeholders should join hands in encouraging other people to join the study. The benefits are high." *(CAG Male FGD 9)*

"Let me thank you for conducting this type of study and having sensitization done in the first place. I believe the main aim of the study is to help people and not any other benefits. I believe that the end of all this will bring us a good thing." *(Healthy adult male 11)*

ACCEPTABILITY OF ACHIMS

1.1 KNOWLEDGE OR PARTICIPATION IN PREVIOUS RESEARCH

Participants expressed confidence in MLW and research conducted by MLW, based on experience of previous research conducted or studies they had participated in. Previous community engagement work was felt to have been important, and successful increasing the likelihood of ACHIMs studies being well received.

"Based on the experience I have had during this research I think it is something important for the study to be conducted, because what we are looking for is not for now, we are focusing on the future. We are eager that in future we will have people who have overcome many diseases… and that there is a suitable way vaccine. So, I feel while considering that there are many risks or other things but a study like this is important to conduct."
(Former CHIM Male FGD 04)

"I feel this is a program with good aims. In most cases when something like this comes from organizations like this one, we trust that it is safe." *(Healthy adult male 11)*

"This research is very possible because there are certain things which we did not know about, but we will be able to know them through this research." *(Community leader female 10)*

ACCEPTABILITY OF ACHIMS

1.2 RECOGNIZED CLINICAL NEED

Participants discussed the clinical acuity of both pneumococcal disease in PLHIV, and TB in Malawi in terms of personal loss, and nation-wide impact. It was felt that ACHIM studies were justified given clinical need.

"Five people who I know contracted TB so it's still a major challenge even in our communities. And even in our settings, the way we live and everything for me TB is something that we need to attend to" *(ACHIMA reviewer IDI NCST)*

"I feel this is good because life nowadays relies on vaccines. A research study like this one will ensure that vaccines are developed to combat diseases we have been experiencing." *(Former TB patient FGD 20)*

"…what comes to the top of my mind is the issue of resistance to antibiotics that we have experienced so far… with that resistance that is there it means even the strains that are coming in are also stronger… so, it means we have those gaps that are existing there so if there is something that is going to come to strengthen those challenges, I think that's going to provide a big benefit to the TB community" *(Reviewer NCST IDI)*

"There is a great benefit because pneumonia is a very dangerous disease, so for our friends who are positive and find a vaccine that will help them against this disease, we will make a big difference because our friends are very vulnerable when it comes to pneumonia" *(CAG Male FGD 09)*

"One of the benefits that can be there is that people who are on HIV treatment will not be getting sick frequently. They will be able to do their daily chores in their homes with physical strength because of this vaccine." *(ACHIMA female adult NC)*

ACCEPTABILITY OF ACHIMS

1.3 NATIONAL OR COMMUNITY INTEREST

Participation in ACHIMs studies was generally viewed as a personal sacrifice, but the benefits of participation, and of running the studies in Malawi were generally expressed as in the interest of Malawi, the Malawian health service, their local communities or future patients. Additionally, there was widespread belief that research conducted in Malawi helped to foster trust in medications or vaccines developed later, as Malawians could be certain that they are effective for them.

"This research is better because it is being conducted right here in Malawi the vaccine can be used here in Malawi there will be effectiveness, or we can say there will be more benefits because it will be done on native people" *(TB Provider FGD English 18.01.2024)*

"What we are doing here will create a good Malawi, and people should be healthy because if people are frequently getting sick the government spends much money. And this is to minimize diseases among people so that people's health should go forward." (*Community leader female 10)*

"In my opinion, all that information is important because this will help us reduce the occurrence of diseases. For example the disease that is under study now oppresses Malawians" *(Community leaders male 07)*

"Apart from reducing the mortality rate others will reduce the number of infections and hospitalization that will be taking place in people of this group, and it will reduce congestion in our hospitals due to such conditions." *(HIV provider FGD 01)*

2. MALAWI-SPECIFIC RECRUITMENT FACTORS

Participants discussed the cultural and location- specific challenges of successfully recruiting participants in an ethical, safe manner in a Malawian context. This was expressed in terms of

1. Misinformation
2. Community engagement strategies
3. Informed consent and literacy
4. Gender-specific factors & contraception
5. Poverty and financial compensation

2. MALAWI-SPECIFIC RECRUITMENT FACTORS

2.1 MISINFORMATION

Misinformation was widely discussed as a risk to the study, and particularly to recruitment. This was prevalent during the COVID pandemic and subsequent vaccine roll-out, and participants described both lessons learnt from this and the impact of persistent misinformation on study recruitment, and risks of participation.

"There are myths in society …they say they want to depopulate through these vaccines. This will make people not to accept the vaccine, they might say maybe this vaccine is for that." *(TB provider FGD English)*

"We wish to have good ways of approaching people in communities once the project is approved because I remember during the COVID era, so many things were said among people. That is just exactly what will happen in the process of waiting for the vaccine under development. We will ask you to have time and explain this to the public as you go round communities to meet with chiefs to make it possible for such information to reach out to individuals." *(Community leaders male 07)*

"Will there be a better strategy of spreading the information or not but using- us it would be very difficult for us, and they can actually beat us in our community meeting just to say in this program they will be giving you a bacterium to monitor you and how your body will respond to it. And the response will be "look now they want to give us diseases in order to kill us" then it will not go well. But maybe because you are doctors you will see how you are going to do it for someone to understand it very well." (*Community leaders female 10)*

"In terms of risk on the part of people conducting the study there is a risk they can face hostile environment because the places they are going there is a lot that is said misconceptions, many misconceptions related to blood… when people hear you are going for a study like this especially in a community that did not understand, chances are high you can be attacked, because they can see that you are doing unethical things so, I feel that is the first risk for people conducting the study." *(Former CHIM Male FGD 04)*

2. MALAWI-SPECIFIC RECRUITMENT FACTORS

2.2 COMMUNITY ENGAGEMENT

The importance of successful community engagement was felt to be of paramount importance to the success of the study, and the safety of participants recruited. A wide variety of techniques to facilitate accurate, appropriate community engagement were suggested, generally including using mass information distribution by megaphones, or radio adverts, supported by key community stakeholders' support.

"...maybe you should have drama group that they can give as example that among the drama group some can be doctors, laboratory demonstrating like they are taking your sample or like injecting vaccine." *(Former CHIM Male FGD 04)*

"We saw MLW coming on radio they can give information that other studies we do this, other studies are like this, people should not be afraid because all this promotes our health." (*CAG female FGD 8)*

"You should as well employ people who will conduct door to door visits. When the community sees a health worker disseminating information regarding health, they feel safer compared to having a mere community member disseminating that" *(Former TB patients FGD 20)*

"For this message to reach a lot of people, there is need to use a car with megaphone spreading the message just like they do when spreading information about diseases such as typhoid. So I think for the message to reach more people quickly, they should use a vehicle that has megaphone/ PA system mounted on it." *(PLHIV FGD 13)*

"When the community understands the study, it will be easy for participants to enroll in the study without compromise. Therefore, it will all depend on how the rest of the people understand it for the study to be successful." *(Healthy adult male 11)*

2. MALAWI-SPECIFIC RECRUITMENT FACTORS

2.3 INFORMED CONSENT & LITERACY

A major challenge to recruitment of participants and community engagement was the baseline lower literacy of the Malawian population and the complexity of the ACHIMs studies. It was suggested that recruitment might be more successful amongst those with secondary education and may favour male participants who were felt to be more likely to have higher literacy levels. These concerns were not felt to represent a complete barrier to participation as all participants in FGDs were able to understand the study, instead a challenge to community engagement and to obtaining fully informed consent.

"But the other issue would also be understanding of the participants, you know Malawi what we call education illiteracy level is on the higher side. For them to understand the mechanism you need to do more explanation. I think people who are better educated will be able to take it in than possibly those with low education." *(IDI 01 TB SPE)*

"The other risk about this research study is that three quarters of most Malawians did not go far with education, most people in Malawi did not get enough education, and whenever someone could be telling them things like these, it becomes hard for them to understand" *(Healthy adult male 11)*

"So, we will still go back to the point of understanding this model. So, I just think that these males they could, I don't know their literacy levels but I am just thinking that these males understood the model better than the females maybe" *(Reviewer IDI NSRHC)*

2. MALAWI-SPECIFIC RECRUITMENT FACTORS

2.4 GENDER SPECIFIC FACTORS

Participants anticipated that recruiting women would be more challenging than men, due to concerns around the potential impact of participation on fertility, childcare requirements, contraception and lower female literacy. Participants were clear that all women participating would have to be willing to take birth control throughout the study period.

"I think more women are worried about their fertility, their children how to look after them if they fall sick from this or if they die. But I think with men they think they can take up the challenge." *(IDI 06 TB SPE)*

"Some churches don’t allow their members to take birth control, therefore this will act as a control measure for the study. In this case, only people willing to do what the study requires of them will be allowed to participate in the study" *(Community leaders male 07)*

2. MALAWI-SPECIFIC RECRUITMENT FACTORS

2.5 POVERTY AND FINANCIAL COMPENSATION

Discussion around appropriate compensation for the studies focused on complexity, risk to the individual and their family, and the challenge of ensuring participants are truthful during screening, adequately informed of risk and not excessively financially motivated to participate. Further, participants highlighted the financial insecurity in Malawi and risk of devaluation of currency during the study period.

"The model itself is sophisticated… so one can expect at least a bigger compensation" *(Reviewer IDI)*

"The challenge will be …ensure(ing) that people that are volunteering are really healthy. That is because some people may come in because they want to benefit financially but maybe hiding something." *(IDI 05 TB Spe)*

"… this is due to the economic status of the country that has led people to consider the financial benefits of participating in the study first. This compensation will encourage most people to enroll in the study" *(Healthy Adult Male FGD)*

"I think the risk that can be there in case that person starts getting sick that means all her house chores will stop and if he/she was doing business that means the business will also stop. And if she/he was also helping his/her children around the household that means his/her children will suffer. That means the organization or the people who are doing this research they need to take part in helping that participant's household like maybe sending those children to school, providing food on daily basis… rather than you just monitoring the participant or registering the participant under different organizations or giving him/her soya and the like- you are only helping that person, but his/her children might be at risk." *(Community leader female 10)*

"We are in hard times devaluation…the package you are planning today can be good but come the time to start implementation of our study money has been devaluate, want to request if you can leave it flexible that you can adjust depending on status of economy," *(HSA FGD)*

3. MALAWI SPECIFIC STUDY CONSIDERATIONS

Once recruited, participants discussed ongoing challenges presented by ACHIMs in a Malawi setting to ensure studies are safe and ethical. These discussions focused on

1. Access to healthcare
2. Residential stay
    - Gender differences
    - Community perception

3. MALAWI-SPECIFIC STUDY CONSIDERATIONS

3.1 ACCESS TO CARE IN STUDY

Participants discussed challenges of accessing healthcare in Malawi, and importance of having designated, high quality and accessible care for participants throughout the study, exceeding the quality of care normally available to them beyond the duration of the residential stay.

"If participants fall sick, access to care should be fast as we know our system sometimes takes long for somebody to access care. I see that there should be a way to track that when they fall sick, we should be able to pick it up. If indeed an adverse event happens due to vaccine, we wouldn't want that, it will be very bad we should bear the cost." *(HIV providers FGD 01)*

"The risk would be... Post infection care because our health facilities are not in good shape across the nation so that will be a big challenge. But if we have established facility that can care for those who are taking participating in the study that will be welcome idea" *(IDI 01 TB SPE* CG)

"When a participant is close with health care personnels they have a feeling of courage because they know if anything has to happen, they will be able to explain to them without any challenges. But when you are far away it becomes difficult just like the way we had a challenge with bad network in the morning, so maybe the person can say I will call when it gets worse." (*Adult Female FGD)*

3. MALAWI-SPECIFIC PRACTICAL FACTORS

3.2 RESIDENTIAL STAY

Participants recognized the importance and necessity of a residential stay during the studies to ensure access to care in emergencies, good monitoring and infection control. They discussed challenges presented by the stay including inconvenience, loss of income for duration of stay, and ensuring the location was adequately equipped and acceptable to participants in terms of food and water quality.

"Well, it's a little bit inconveniencing but if the volunteers are already aware of it are prepared are well taken care of in that residential site its ok because they are volunteers, so they have been told fully this information. But from the medical point of view, I think it's a safe way of making sure that these patients are monitored" *(TB IDI)*

"Mm, that's a huge task, imagine a family man or a career person or even a businessperson confining them to some places, …, I know they maybe there will be some compensation, but will the compensation be enough to make up for the lost time, the lost opportunity" *(TB IDI)*

"I think the isolation in terms of wanting to observe them in case they develop an infection and they become sick is a good idea my only other concern will be, what's the isolation place like, in view of the factor as I said these people might have all sorts of comorbidities but be held from an HIV point of view so what is happening in that kind of setting what kind of food are they taking, what are they drinking what's the rooms like, but I believe like they are made well just to make it secure or a good isolation place. Am sure you looked into all that " *(PLHIV IDI)*

So, if the stay in place is like that, that's fine provided they have proper equipment to take care of patients in case they develop something serious**,"** ***(****PLHIV IDI)*

3. MALAWI-SPECIFIC PRACTICAL FACTORS

3.21 GENDER SPECIFIC ISSUES

Specific to the residential stay, participants discussed the potential for this to act as a barrier to participation for female participants, particularly those who are married or have children. This was not felt to be a limitation for male participants.

"Most women ... they have kids to look at home so it will be challenging for them to leave their family for three days rather than men its easier for men to leave home for three days," *(PLHIV IDI)*

"It will not affect one participating, but it will affect the whole family... because the other cannot accept their partner to stay away," *(Religious Leaders FGD)*

3. MALAWI-SPECIFIC PRACTICAL FACTORS

3.21 COMMUNITY PERCEPTION

Concerns were raised about the community perceptions of individuals (particularly women) staying away from home, as it would be difficult for participants to enroll in the study without their whole community being aware of this. This emphasised the need for good community engagement to minimize stigma and gossip.

"In different families, some men are reserved while their wives are naughty, to the point that they sneak from their houses just to attend research meetings without their husbands being aware..." *(Community Leader Male FGD 07)*

"...it is difficult for a married person to leave his /her partner…like for a week staying out. I think it will not only affect the one participating, but it will affect the whole family. That is where the story is getting twisted because others cannot accept for their partner to stay away” *(Religious Leaders FGD)*

4. STUDY SPECIFIC CONSIDERATIONS

Findings were generalizable between the 2 ACHIM projects discussed in terms of recruitment and basic practicalities of study format. The following specific concerns were discussed for each study.

- TB ACHIM
  - BCG Familiarity
  - Biopsy
  - Bronchoscopy
- PLHIV ACHIM
  - Vulnerability of patients
  - Screening and CD4 count

4.1 TB ACHIM

4.11 BCG FAMILIARITY

The fact that the majority of Malawians are familiar with the BCG vaccine as part of the standard childhood vaccine programme was a strength of the study design, increasing the perceived acceptability.

“The groundwork is already there the vaccines are already there, there is at least a level of protection that is already existing for most of uses, that’s what will make it less worrisome” (*Reviewer NCST IDI)*

"I think injecting BCG, most of the people will welcome the idea because they know BCG. We inject the children, it’s not something strange so that should be quite acceptable. And BCG is almost given to every child if you can tell the people every child receives BCG, I don’t think that will cause worries it will be accepted" *(TB IDI)*

"I think the ideal place to have the BCG injected should be the upper arm just the way we do it when giving the BCG to a baby" *(Former TB patients FGD 20)*

4.1 TB ACHIM

4.12 BIOPSY

Concerns around the biopsy focused on the potential cosmetic impact of removing tissue from the upper arm, and those around the potential for misinformation or misconceptions around cultural phenomenon of using human tissue for ritual purposes.

"It will depend on when they take the biopsy what does it leave a big scar or a small scar? That will be a concern, and especially for women for cosmetic reasons. In summer some women want to wear clothes that are showing the arm so then they might prefer another site because of this issue" *(TB Specialist IDI)*

"I think it's the actual tissue itself that will be a problem. People will say maybe they will sell it to witch doctors, or maybe they will use it for other things- for rituals and other things." *(Reviewer IDI NCST)*

"We need to be very careful on that part because nowadays, most people believe that some people do business from collecting samples from individuals' bodies. Before this process, participants should be told about the procedure earlier." *(Former TB patients FGD 20)*

"Concerns can be there because it's not all people who can accept this there will be other people who will say it's satanic to take body tissue, they put infection in the body the person will refuse, it will look like you belong to the satanic." *(CAG female FGD 08)*

4.1 TB ACHIM

4.13 BRONCHOSCOPY

Bronchoscopy was viewed as being concerningly invasive, and thus less acceptable. Participants were concerned particularly about infection risk, discomfort, risk of complications, and doubt around the efficacy of the technique.

"I feel most people are afraid of using this method of passing the pipes to the lungs because this method causes most people to develop different infections to the point that some individuals don’t get cured from their conditions. This is the reason why most people don’t agree with this method" (Community Leader Male FGD)

“Most people did not agree with the method of placing a pipe through the mouth to the lungs. It would be ideal to find another way to get the lungs in order to collect some samples from a living person, this method of placing a tube in the mouth or nose of an individual through the lungs can be ideal for a dead person” *(Community Leaders Male FGD)*

4.2 HIV PNEUMOCOCCAL ACHIM

Participants discussed concerns around the clinical vulnerability of PLHIV to infection, and discussed ways to ensure the study protocol adequately reflected this, including access to care and screening based on CD4.

"We cannot consider CD4 for everyone but for those we feel they have very low CD4 we emphasize to them about everything they are going to feel or come across or experience they should report because these are prone to infections if they can get sick with the infection itself. So with low CD4 there should be readily available care for them not to deteriorate. Readily available care should even include mode of transport for the person to reach the hospital" (*HIV Provider FGD 01)*

"Maybe for HIV positives if you could set CD4 cut off point, if CD4 should not go beyond this point in our study, the lowest should be this, and we take like that so that we do not get too many risks" *(HIV Provider FGD 01)*

"He means to say: a person carrying a 50 kg bag is said to be struggling already to bear with the weight and once we stop him over to add an additional 20 kg, I feel this will not be realistic. This is why we are saying an HIV positive individual is usually loaded with a number of conditions, by inoculating the pneumococcus bacteria in this person, we will be jeopardizing his health, making it hard to treat the pneumonia, unless this bacterial is tried on a health individual for effective results" *(Community leaders male 07)*

"These are like vulnerable people so those safety standards need to be taken into account, there should be proper mechanisms needed to see to it that they are being followed" *(Reviewer IDI NSRHC)*
