# Supplementary material for: Acceptability of advanced Controlled Human Infection Models: The case of a pneumococcal challenge in people living with HIV and mycobacterial challenge with Bacillus Calmette Guerin in Malawi
Source: PLOS Glob Public Health. 2026 Aug 18;6(8):e0006236. doi: 10.1371/journal.pgph.0006236 (PMC13485124; doi:10.1371/journal.pgph.0006236)
Supplement: Supplementary file 1 [file pgph.0006236.s001.zip › Documents/ACHIMA_ FORMER CHIM FEMALE_FGD_05_English.docx]

Video play:	It’s a given that vaccines prevent diseases and saves countless lives, but this takes ten years or more to develop new vaccines. We need to find a faster way, one way to speed up the process is by making better use of human infection studies, volunteers are given a sample of vaccine under development followed by a careful monitor infection medical care is always close to hand if volunteers fall ill and if they stay well, it means the vaccine works and can be made available much earlier. The idea is almost 300 years old but using todays advanced technology this type of study is not just scientifically sound but ethically responsible too and of course follows strict rules. Human infection studies show huge promise against fighting diseases like Typhoid and malaria but to develop vaccines most effective at the people at risk we need to test on those at most risk by expanding human infection studies and targeting them better the people most at risk could help develop vaccines of the future and save millions more lives.

Interviewer 1:	Let’s repeat it.

Participant:	Yes.

Interviewer 1:	In the video they were explaining about how vaccines protect us from diseases and how it saves many lives, but it takes ten years or more or it takes along time for this to be done. So, as one way to develop vaccines or getting vaccine quickly. Away of conducting this research the one you took part is one way to help to find vaccines faster or to find medicine for medicine to be developed quickly unlike how it happens when it’s going well that they are making the vaccine from a scratch.

Video play:	It’s a given that vaccines prevent diseases and saves countless lives, but this takes ten years or more to develop new vaccines. We need to find a faster way, one way to speed up the process is by making better use of human infection studies, volunteers are given a sample of vaccine under development followed by a careful monitor infection medical care is always close to hand if volunteers fall ill and if they stay well, it means the vaccine works and can be made available much earlier. The idea is almost 300 years old but using todays advanced technology this type of study is not just scientifically sound but ethically responsible too and of course follows strict rules. Human infection studies show huge promise against fighting diseases like Typhoid and malaria but to develop vaccines most effective at the people at risk we need to test on those at most risk by expanding human infection studies and targeting them better the people most at risk could help develop vaccines of the future and save millions more lives.

Interviewer 1:	So this was a short video that was explaining about developing vaccine, it has been known that vaccines protects us a way of developing vaccine takes many years but through this modern method which is like using bacteria to people and monitor how their body will react to the bacteria that has been given is a way that helps to come up with vaccine quickly unlike the usual way it takes long like it takes 10, 15,20 years its what is explained in the video. Before going into the discussion there is anything that you want to say or where we need to explain clearly.

Participant:	We do understand.

Interviewer 1:	You understand ok, we can continue with the discussion now. Welcome to the discussion where we are discussing about controlled human infection model or CHIM in TB as explained our discussion mainly will focus on TB. We MARVELS group from Malawi Liverpool Wellcome Trust	are the ones conducting the study we have plans to extend our work in pneumococcal challenge to reach people with HIV and TB. Your views on the study will help us to know the procedure to follow in this type of study in future. Who should participate are former TB patients, healthy adults, people who participated in CHIM study, doctors, nurses taking care of people with HIV or TB, health surveillance assistants, community leaders, religious leaders, community advisory committee members, research ethics committee. We are saying groups of people that will be engaged we are engaging different people health providers, those who participated in CHIM study, community leaders, religious leaders are some of the groups that we will have discussions with them. We want to know what your views on TB CHIM study in health adults are. People conducting the research will explain in brief about CHIM and purpose of conducting the research in people who are at risk like people with HIV and TB this has been explained. Now we can start our first section. I just want to know on everything that has been explained about this study that we are planning to conduct what message have you heard.

Participant:	No response (silent)

Interviewer 1:	What message have you heard or what message have you captured on what has been explained. Speak louder.

Participant 3:	On what you have explained, I have heard clearly that you want to know if pneumococcal bacteria can be given to people with HIV or TB to receive it.

Interviewer 1:	Ok she has answered on her part, we hear differently everyone has picked a certain point we want you to share with us points that you have captured on what we have told you.

Participant:	No response (silent)

Interviewer 1:	Let’s go points that you have captured on everything that we have explained, what message have you captured, if someone ask you where you went what was it all about what can you explain.

Participant 6:	What I have captured is that developing vaccine takes a long time more than 10 years, nowadays vaccines need to be developed faster using modern way of using volunteers to take part to receive this vaccine.

Interviewer 1:	Ok, others what have you captured.

Participant 1:	What I have captured is that you want to see the difference in heathy people and those with HIV and TB to see how it will work.

Interviewer 1:	Mmhi, others our views or I should ask in English.

Participant:	He has already spoken

Interviewer 1:	No, opinion can she say precisely from the information that you have been given, what message do you see as very important.

Participant 4:	Based on what you have told us, from what you read about TB as we joined we negative people the bacteria that you gave us the same bacteria you want to try in people with HIV and TB those with low immunity to see how it can help them or will they be on risk, or at what risk would you put them

Interviewer 1:	Other views/opinions let’s not get discouraged its working let’s go on in your views on what has been discussed, which message have you seen is the most important of what has been discussed here, what have we captured from the message and which message do you think is the most important on what has been explained.

Participant 1:	The message that I feel is important is the one differentiating between those people that are infected and people that are healthy when they receive the vaccine how will it work in them.

Interviewer 1:	How important is the message to you lets help each other, when I ask a question it’s for everyone it’s not the question is for the person who answered all of us participated, we all have been explained we want your opinions from everyone from your point of view.

Participant:	Maybe come again

Interviewer 1:	I should come again I asked a question what message you think is important of all the things that have been explained, which message do you think is important.

Participant 6:	The message that I feel is important is on risk and benefits that can be there when a person participates in the study, you have explained that there are no major risks that can fall on a person volunteering it’s just the time they can take participating no like health risk or anything that can happen to him.

Interviewer 1:	Others what are we saying.

Participant 5:	You have explained of people who can take part in the research, people from 18 years going upwards those that are healthy are the ones to join the research study.

Interviewer 1:	What do you think is the reason why we have said the people supposed to take part are from 18 years upwards.

Participant 6:	Because an 18-year-old person can at least think properly can make decisions on their own unlike those under 18 years.

Interviewer 1:	Ok, any other reason that others feel that.

Participant 7:	Yes, I feel like a person who is 18 years to compare with a child who is 10 or 9 years their bodies there are some things that their bodies cannot overcome like the body is not strong compared with an adult person.

Interviewer 1:	Ok before we go further do you feel there any point that need to make further explanation, there is anywhere you feel need further explanation or anything.

Participant 4:	You have explained, we have understood still more it’s inevitable, but we have understood.

Interviewer 1:	You have understood but you are not saying anything others we are just quite listening, we want to hear from you, you are the ones to talk more in our discussion when we ask a question you are the ones to control flow of discussion after one has shared her views another one to share like that , in the discussion one can give supporting views another one can give disagreeing opinion not so. There is no right or wrong answer we are taking views/opinions when giving views/opinions everyone gives opinions according to his point of view it’s the same feel free to give opinions according to your point of view and how you understand the question, It will not base on who answered the question at first, or he answered in this way that means we have to respond towards that way no.

Interviewer 2:	Ok maybe just to answer the question or to clarify so that we can be together, we explained that we are here to hear your opinions/views on two things understand me. We were giving pneumococcus bacteria to healthy people meaning people who are HIV negative. After giving the bacteria, we were going through all the process looking after the people they were spending night at Grace Bandawe they were coming to clinic for check up to see how your body reacts and to saw that of all of us no one suffered any serious illness.

Participants:	All mm (agreeing)

Interviewer 2:	So we said that we want to extend this research in these people with HIV we put pneumococcus bacteria because we already know that people with HIV their immunity is low and these people are the ones who suffers from pneumonia in hospitals. We can be on risk but our friends can be on higher risk unlike us. So, we want to see how our friends can react the ain is in the end it will be away to find vaccine that can deal with pneumonia. We want to give pneumococcus bacteria to people with low immunity those with HIV we want to know your views/opinions. When asking questions, you must focus on these two things that am explaining when answering questions. The second thing we have explained about TB CHIM understand me on this for TB we are not giving to someone with TB no understand me, but we are taking TB bacteria give it to a healthy when I talk of a healthy person, we mean the person is HIV negative who does not have TB. We have vaccine for TB all of us we know that, and we received it when we were young children despite receiving BCG vaccine still more when we go in hospitals, we will find TB ward is full why is it that people are still suffering from TB despite having the vaccine that is being given. Can’t we make it that we see how the vaccine works or find out if the vaccine is effective to come up with solution to this, we are conducting research. The research that we want to conduct about TB is we will take TB bacteria give it to a healthy person and see how he reacts we will look after them at the end of it we will find solution to TB disease be it coming up with a new vaccine to boost the vaccine that is already there so, we are discussing on these two points.

Participant:	Mm

Interviewer 2:	So, we are giving pneumococcus bacteria to people with HIV and giving TB bacteria to healthy people who do not have TB or HIV it is understood there.

Participants:	Mm

Interviewer 2:	I have explained clearly on the question you asked when answering let’s focus on the two points that I have explained we can continue, you have questions.

Participant:	Yes ok, on the issue that we are discussing you said that for those people that have disease we are giving vaccine. I want to ask that for some of us we were healthy, and some were reacting, they were developing flue like suffering from flue that were probably fine so, for these people they are like they have other problems when we give them this vaccine will that not cause other serious problems.

Interviewer1:	We want your views/ opinions like that to give as your views that the way I feel as you have put it I feel this can cause what you have mentioned, these are the thoughts that we are looking for, what you will think that they were giving us we w ere healthy so, we take a person already on a risk, what you think can happen is what we are looking for to help us to have views of people, in your opinion just looking at that this can happen it’s what we are looking for or if this can be a better way explain the benefit that can be there, the risk that can be there that is the purpose of the discussion

Participant:	Mm

Interviewer 2:	I believe you wanted to say bacteria not vaccine.

Participant:	Yes, bacteria

Interviewer 2:	Your question has been answered, you understand clearly not that we give you answers that it will go like this, but you are giving us.

Interviewer 1:	Answers are with you, you are the ones to give us answers, we can continue ok. What questions do you have, questions that you have reaching this point?

Participant 4:	My question is that you are saying there will be a vaccine or bacteria that will be given to a healthy person on TB, when you give then the bacteria there will be no any other risks like the person suffering from the disease for someone who was ok

Interviewer 1:	As we have explained that you must give as your views for that to help us, the views that we want you to give us don’t give them as questions they should be your views on how you look at it. I feel when we do this there will be no any problem that can come to a person the aim is we want your views/ opinions on this topic. Taking a bacteria like this one what can happen we want what can happen to the person at that time concerns that you can have or how you feel or you feel there will be a benefit or what can happen. Let’s go on we should start your views after explaining like this how do we look at doing this type of research study.

Participant 3:	In terms of giving bacteria for pneumococcal to a person with HIV I feel it will depend on how viral load of that person is, if a persons’ viral load is high the person can get sick but if a person is taking medication properly viral load can be low they say 1000 they can survive because looking at the bacteria I feel it just make one to feel like you are starting flue but you will not get sick you just feel symptoms that you want to get sick like that but many not side effects

Interviewer 1:	Thank you, other views.

Participant 5:	I feel like this can help giving bacteria to people with low immunity because in the end it will find solution that you explained that people will be given medication for TB when you research there are a lot of people meaning to say the drug is not effective meaning to say there need to find out another way that can support to reduce number of number of patients in the wards, because when we look at TB ward people stay in the ward longer some have been sick suffering second time that they were not healed but if they are given the bacteria the reactions can help to come up with permanent solution.

Interviewer 1:	Ok, good opinion other opinions/views

Participant3:	On TB issue my views because we get TB through air I feel it spread faster because most times its not that a person’s immunity is low but because people react differently to diseases when immunity is low to fight TB bacteria so coming of the vaccine will help people to gain like resistance on TB and that they will increase strength of TB.

Interviewer 1:	Mmhi, other views

Participant 4:	Here I see that maybe TB medication given to people in hospitals it shows that it’s not effective because when we look at numbers in communities’ others sick others even dying if this idea of research can be done it can help a lot and it can save many people from this disease.

Interviewer 1:	Ok, other views

Participant 3:	On TB I feel like giving a person bacteria for TB cannot cause him to reach complication stage that it can get worse because TB treatment heals of course other people get healed later they start again because they did not finish treatment but because you will look after them the dose will be completed. A person who will be found getting sick due to the bacteria will get healed properly but of his immunity is ok does not have any diseases.

Interviewer 1:	Ok thank you very much let’s continue from there.

Participants:	Mm

Interviewer 1:	What additional information that you feel people must be informed before making decision joining in this type of research study.

Participant 7:	I feel like before a person joins you must know if they already have HIV already before joining the study, they have to be informed that if they have been given bacteria and it has affected him explain to him that you will support him in this way.

Interviewer 1:	So, can you explain what you mean, what you think can be the support that can be there like the package that can be in place on care/support can you explain care/support in what way.

Participant 7:	In terms of medication that maybe it does not affect his	daily life like if he goes to work you will see what to compensate with

Interviewer r 1:	Mmhi, let’s help each other.

Participant6:	Just to add on what she is saying adding on informing them if they get sick be during the night you can help them taking them to hospital for treatment to meet with senior doctors to see what the problem is to be helped.

Participant 1:	On the same point I see that before joining test them to check their body immunity if it’s very low to take good steps on how you can handle those things.

Participant 3:	I see that after conducting test you will counsel them to take medication and explain to them benefits that they can get after joining research study getting pneumococcal bacteria benefit for example you will have a way to give to HIV positive people vaccine.

Interviewer 1:	Number 4 you wanted to say something.

Participant 4:	Has already been said.

Interviewer 1:	Has already been said, any additional before we move on here, we are done with the first section, section A we are done we are going to section B. Just want you to explain benefits that can be there conducting a study like this giving people the bacteria, what benefits can be there.

Participant 4:	The benefit that can be there conducting the research study it can save lives of many people those that will follow the advice that you will give them. They must adhere to advice if they are to take the drugs. It can be right because their lives will be safe it can be different with those people who did not join study they are just staying.

Interviewer 1:	Another benefit that can be there.

Participant:	Another benefit that maybe can be there it’s a lot of benefit it will help people who are sick to get help for them to get proper care up to when they are healed

Interviewer 1:	On that you did not speak, what are your views on benefits that can be there.

Participant:	When people take part

Interviewer 1:	Number

Participant 2:	Benefit that can be to a person with TB giving them bacteria it can help to know.

Interviewer 2:	Wait there a person with TB or you want to explain.

Participant 2:	A person with TB you are giving the bacteria.

Interviewer 2:	No bacteria we are not giving to a person with TB already we are giving to a person who does not have TB or HIV, wea re giving them bacteria causing TB you understand. A person who has HIV we are giving bacteria that cause pneumonia you understand here or there is breakdown.

Participant:	A person with

Interviewer 2:	Those without TB

Participant:	You are giving them TB bacteria

Interviewer 2:	Yes, as you are healthy you don’t have HIV, you don’t have TB we take TB bacteria and give you to see how your body will react, how your body immunity will react depending on the results that we will see in you it will help to find vaccine another vaccine to boost BCG vaccine for TB which is already in existence to reduce TB cases in the country. But for those people with HIV already we are giving them bacteria that I was saying for pneumococcus we are extending to give people with HIV. You received pneumococcus bacteria we conducted blood test to check if you are ok when you are ok, we move on giving you bacteria so now we are taking people who are not healthy in their body those that are HIV positive we take the bacteria give them and see how people react, results that can be found in these people and what we can found in you will help us to come up with vaccine to deal with pneumonia, have we left anyone behind

Participants:	No response (Silent)

Interviewer 2:	We are all moving together now, if there is need for further explanation say it, I want us to move together, we all agree that it’s understood.

Participants:	Yes

Interviewer 1:	What benefit can be there in conducting TB CHIM research study.

Interviewer 2:	There we are taking TB bacteria giving a healthy person who is not suffering from any illness and then we say what benefit can be there, what benefit can we have, some are what they have already started explaining.

Participant 3:	The benefit is that a person who has been given TB bacteria has to be without HIV and TB it will help to see how a person reacts to TB bacteria if a person will suffer from TB you will give treatment it will help to boost drug treatment and it will help in terms of people to develop resistance for TB when he will be attacked again by TB that one will not get critically sick like how would have get sick before introducing bacteria

Interviewer 1:	Ok, other views/opinions

Participant 1:	Giving TB bacteria to people will help to know how long it takes to see the outcome when one has TB.

Interviewer 1:	Mm other views/opinions

Participant 4:	In terms of giving bacteria to people not suffering from TB it will help to know body functioning of that person if he gets TB in his body how long it will take to have full borne TB. Because if you have given bacteria if like you that he is sick or has show signs of illness quickly or showing any other symptoms that can make to know that person will not take long to suffer from TB. When he gets TB quickly you can have the idea on the ways how you can help him in the ways how you know the disease

Interviewer 1:	Thank you there are other views on benefits that can be there.

Participant:	The benefit will be that it will help to reduce TB cases because research will be carried out to see if a person responds well to the drug that will be developed based on the results of the research

Interviewer 1:	Ok what do you think are the risks that can be there conducting TB CHIM, risks that can be in conducting this type of research.

Participant 4:	There are other people suffering from TB they can go to hospital and be told not to smoke, not to drink beer it turns out those people are not listening when that person get sick and you give that person TB bacteria the person is smoking with bacteria will that can put him on a certain risk to the body of a person in the end they will say its because of the bacteria that was introduced yet he was not adhering properly.

Interviewer 2:	How do we do on that for these people, can we put bacteria to these people, and they put themselves on risk at home

Participant 1:	I feel like after giving them bacteria you tell them if they drink beer or smoke marijuana they have to stop until you get the results after they can resume	because when you do that, that will bring you other problems you will say we have given you bacteria not to destroy you yet that is not the case

Participant 3:	Also to stay away from children as well as older adults suggesting that children and older adults their immunity is low, they can transmit to these people.

Participant 7:	I see that after giving them bacteria it is better to put them like as we used to at Grace Bandawe they should stay there atleast longer before going to their homes.

Interviewer 2:	You were thinking if we could keep them for how long.

Participant 7:	Maybe for two weeks

Interviewer 2:	2 weeks

Participant:	Mm

Interviewer 2:	Ok, thank you.

Participant 3:	On the same point I see that it’s good to say they should avoid over clouded places they can contract other contagious diseases through air that can cause TB bacteria to mix with other diseases making a person to get sick easily

Participant:	Let me ask when a person is given bacteria how long does it take to know about his body

Interviewer 2:	The bacteria

Participant:	Those that have been given how do you know this is the reaction like it takes one or two days.

Interviewer 2:	Ok, on that we have not started conducting the research it will be difficult to explain that it will take two weeks or one week.

Participant:	Oo, like the one we participated

Interviewer 2:	Yes, that one we can know, that is the reason after giving bacteria we were telling you not to go home because we believed the first 3 days is what is critical for pneumonia. But on this as it was said how long it takes but you can share everyone’s views (44:58-45:07 could not get what participant was saying)

Participant:	The one we were given last study it was known that 3 days we were communicating, looked after that if anything can happen there was adequate care/support this one as you are saying you need to arrange the period

Interviewer 2:	In other words, how long should we keep them there.

Participant:	Yes, what you can (audio not clear what participant was saying)

Interviewer 2:	No, thank you very much, very good thoughts.

Interviewer 1:	Ok, looking at risks and benefits what are your thoughts for this research to be conducted.

Participant 6:	I feel like when we weigh the two benefits is what outweighs because it will help us to find drugs or vaccine (audio not clear background noise sensitization vehicle passing) many people this disease is in the group of dangerous diseases in the country. On the risk that we have most of the risks can be prevented like what people were saying that if the drink beer or smoke when conducting our study, it’s better we encourage only those who don’t smoke or drink beer for them to be part of people taking part to reduce some of the risks

Interviewer 1:	Ok other views when we weigh risks and benefits for the research to be conducted what is outweighing the other between the two.

Participant 3:	Maybe with what my friend has said that benefits outweighs because this will help to develop vaccine or to come up with drugs unlike how it was before basing on the reactions that people will show after receiving the vaccine.

Interviewer 1:	Ok other CHIM’s have been conducted in Malawi or to say other research like these have been conducted here in Malawi using pneumococcus in healthy people (those not sick) how can you differentiate that research and this one in people with HIV.

Participant;	Repeat the question.

Interviewer 1:	Other CHIM research like this have been carried out here in Malawi using pneumococcus in healthy people how can you differentiate that research with this one in people with HIV

Participant 6:	I feel like the difference is that those people that participated in this study were healthy and now we want to use people with HIV those that their body immunity is low, the main difference will be that that they can be on small a risk unlike those that were healthy they can get sick or they can have some side effects unlike healthy people

Interviewer 1:	Ok other views let’s go.

Participant:	No response (silent)

Interviewer:	These are the views that are there on differences that can be there conducting the study in healthy people and people with HIV

Participant 3:	The other difference is that it will help for vaccine for pneumococcal to negative people on how it will help them and how you will help people with HIV on pneumococcal issue you can not give vaccine to healthy people only you will find a way to give even those that are	HIV positive

Interviewer 1:	Ok

Participant 4:	There some people who are HIV but they are healthy they look they can have protection in a certain way and there are other	people who have HIV but their body looks weaker with the vaccine that they will be given it can put them on risk maybe in these people who look weak because other people looking at them you can tell that they have HIV, others when you say they have HIV they are healthy because of way of living in homes, others eat as advised at hospital while others live in poverty at their home, others don’t eat adequate food with the disease that affects their health with the bacteria in these people they can be on risk at risk with their body

Interviewer 1:	Ok risks that can be there how can we explain them to people when giving information to people how can we explain about the risks.

Participant 3:	Risks that can be there to people receiving vaccine, but they are positive it’s not that some will get sick others will get sick, but some will be ok depending on their viral load in their body.

Interviewer 1:	So, how can we explain that what can be the best way of explaining this risk.

Participant:	We will explain to them that it is good to go to hospital for viral load checking to see how their viral load is as well as taking medication in order when the bacteria is introduced low viral load causes serious illness unlike someone with high viral

Interviewer 1:	Ok we move on to the other section where we want to ask your views on differences in participating in research study like this between men and women. Just want to know how you can feel a woman or man taking part in this type of CHIM research.

Participant:	No response (silent)

Interviewer 1:	How can you feel a male or female person taking part in this type of research.

Interviewer 2:	Or you feel who can be fit to participate between male and female participating in this research.

Participant:	I feel like everyone you reach out to them everyone be it male or female can come

Interviewer 1:	Mm

Participant:	I feel like everyone must participate

Interviewer 1:	Other views

Participant 3:	I feel women are the first to take part in research because women are at risk of facing with different problems and they can pass on to other people as they take care of children most of the times they are guardians, women are involved in most activities they are the ones who can spread diseases faster when they get sick they can cause a lot of people to suffer

Interviewer 1:	Ok other views

Participant 4:	But research is voluntarily it just depends on how one has understood be it a man or woman because you responded that research is voluntarily when a man doesn’t want you cannot force him, if a woman doesn’t want you cannot force them. A person must feel how he has understood if they want to join research or not.

Interviewer 1:	In our previous research work for example a lot of men joined the study unlike women what do you thin can be the reason why it was like this.

Participant 4:	Ok, most of the time when research studies are conducted people talk different things in the communities’ others even the research that we went to Grace Bandawe people talked a lot of things that we did not see them there that there are snakes what. We found that among ourselves discouraging each other I work at Queens, they ask me about the study I joined if I faced ant problem, they say they remove uterus people talk a lot we found people are afraid that I like the research I will join but to get uterus removed, and they say there are snakes there we tell them there are no snakes the way we experienced it in the study we joined

Interviewer 1:	Explain what the reason can be why most men are the ones who joined the study unlike women.

Participant 1:	I feel the reason is that women we are the first to be afraid of everything unlike men. With what we heard there was a lot said we are obsessed with what is said unlike to face it ourselves while men they would go to see that is the difference, that is the reason you find more men are the ones who participated more than women.

Interviewer 1:	Ok

Participant 6:	The other reason I see it can be here in Malawi most women we are the ones staying at home taking care of children and other things, so to tell you to go to Grace Bandawe 3 days maybe you are married it’s difficult they will ask where you are going this and that maybe that is the reason that turned out most not to participate

Interviewer 1:	Ok

Interviewer 2:	Another maybe before continuing you have explained women are the ones taking care of the family, spending a night outside this is true how can we help women that this time maybe more women can take part.

Participant 1:	I feel that when they are coming to join study at first sit down with them explaining to them in details so that they communicate to their family, if they can agree they are they are welcome to continue.

Participant 5:	But I feel if there can be suggestion if men, they can understand faster unlike we women when we hear something we believe on hearses, and we get discouraged. If there can be a way to reach out to them as you did at the beginning, we were many we were saying we heard from friends. If there can be another way where women are found in different groups if they can be approached teaching them separately maybe they can understand maybe they can take part. Most of them maybe they are afraid of what they have herad

Interviewer 1:	Ok

Participant 1:	Still on the same I feel like at the beginning follow them as it is here in Ndirande when you reach at the market share information of what you have with people, the people with what they have heard will follow you or not.

Participant 3:	I feel like giving people chance to choose how they want to be a assisted by giving them several options like spending night at Grace Bandawe and going to monitor them or staying at their home don’t be close to people I don’t know what ways you can use for them not to stay at certain places long

Participant:	I feel like if the approach of spending nights if it can be dropped just call them that if you have anything call or calling them regularly after three 3 days it’s like men start losing trust for married people on that

Interviewer 2:	Ok, maybe to assist each other on that the reason people should spend nights there we want to protect people at home. We have given bacteria we said the first 3 days is what we call it very critical you can find neighbours child don’t know that you joined study run to you cough in presence of the child we see that at home you can transmit to another person that is the reason we say you sleep at Grace Bandawe. We can discuss now for people at home to be safe as well as the person participating to be safe.

.

Participant 1:	I feel when people receive the bacteria they should stay at Grace Bandawe because we can nor relay of following them in their homes. People can be getting sick at night these days our government the way its running sometimes fuel is scarce we find that the car that runs does not have fuel it will be to reach out to people to be helped properly.

Participant:	The ones taking part in the study must go to their family discuss with the family when the husband understands he should give a go ahead to join study let it has been given ok by the husband to join study

Participant 3:	I feel like can it be possible for married people to come with their spouse to explain to the husband benefits of staying at Grace Bandawe, other people can lie home they are going other ways that can be difficult for a woman to join study.

Interviewer 1:	Ok

Interviewer 2:	In other words, we are agreeing for people to sleep at Grace Bandawe, but we must explain to their families than you.

Interviewer 1:	On still on the same issue, we have talked of going to study site this just came in what we were discussing was difference in men and women participation in the study. Now we come on another topic women that are of childbearing age who are ready to join study should be ready to follow family planning method during the time they are participating in the study. What are your views on this issue. The first part is a statement explaining that women of childbearing age this is an adult and those women who are ready to join the study should be ready follow family planning methods. Am asking what your views on this on people are joining the study in future to practice family planning methods during the time of participating in the study.

Participant 4:	Those that are to join study those receiving vaccine a person who is pregnant or those about to mensurate after a week you join study they can be at a risk they can receive in the middle of the study they can be found to be pregnant, the child can born with problem they can blame you yet he did not manage things properly

Interviewer 1:	On the point that I want to say women that want to join this study they must practice family planning during the time they are participating in the study they have to practice family planning methods because they are joining study what are your views.

Participant 3:	I feel it is not good to force women to practice family planning because some are teenagers who feel they have not started sexual life that can discourage them to join study the best way is what you were doing testing for pregnancy my opinion

Interviewer 1:	Your views that side you seem you want to say something.

Participant:	Mm, for me it’s a good way because some people go to clinic as we do others go to take family planning services knowing that they are already pregnant. When they go there at the beginning, they don’t conduct test they just receive family planning services. You find they are pregnant they say am pregnant, yet I was using family planning, yet they were already pregnant if they can come now conduct pregnancy test to confirm if they are pregnant or not because you can put them on family planning while they are already pregnant.

Interviewer 1:	Mm

Participant 4:	They should come with health passport that they use when getting contraceptives, they can come there, you conduct pregnancy test nit pregnant that time they can join study others don’t follow family planning they can get injection this month they are given date to receive another injection they miss the date they get it the other month there can be a problem. They must bring health passport to check how it has been going. These days people start sexual life at 12, 13 years old they know men it cannot be possible to find 18 years old who don’t know a man you will find problem in the middle of study she has become pregnant while in study the child having problems.

Participant 5:	I feel its good that they should not get pregnant during the time they are in the study because when a person is expecting there are complications maybe immunity others are on other problems in addition to pregnancy that can compromise unborn child and health of the woman it can be affect the study

Interviewer 1:	Different views have been shared helpful I don’t know at the end the point that we can conclude on this point of giving family planning methods during the time women are participating in the study tha is coming.

Participant:	Let it continue

Participant:	Best way

Participant:	But I think it should be choice of a person there are other people they don’t practice family planning they are not married; I feel it should be personal choice to take family planning or not or I will just abstain from

Interviewer 2:	It’s what I want to clarify there are several family planning methods for women, you can see on your own that I can manage this if you see that you can manage to abstain during the time you are in the study it ok. Other counting their dates these dates I can be at risk. I want to address number 3 concern	I want to address this concern mainly we are not saying take loop or implant (Norplant)) because you are in a study, but we want just the time they are in the study for them not to conceive because if they conceive as said a person is weak already pregnancy comes with it’s issues when we give the bacteria when you are pregnant we can put them at risk. So, family planning we are not going to say practice we were thinking to support with any family planning that they can follow but during this period you should not be pregnant is that clear.

Participants:	Yes

Interviewer 2:	We have agreed people have to practice family planning during the time they are in this study.

Participants:	Yes

Interviewer 1:	Ok we can continue I want before going there want to ask looking at this who can be a proxy for a person participating in the study.

Interviewer 1:	Whom do you think can be a proxy for a person who is participating in a study. A proxy who can stand for me when I want to join study, I will go there I will have someone who the research team can contact when they need me my number is not available someone whom they can contact on my behalf as a proxy when I join study I have written SS have submitted her name as a proxy, in your views who can be this person as proxy for the one taking part in a research study.

Participant 1:	I feel like when everyone is coming they will be counselled everything explain what, you are at the point of joining to start the study they should give number for people whom they live with if there are children for those that are not independent they have to give contact number for the people they live with if you did not get in touch with participant the one living close to them can communicate well.

Interviewer 1:	Has said her part the rest of us our side.

Participant 4:	Or your sister you can put her contact number and you give the ones close to you not a person living in the village yet you are in town you give contact of that person no that is not on but for someone living within town you can take the contact number and give if there can be any problem or his phone not available they can contact the sister

Participant 5:	For those that are married their spouses.

Participant 3:	They can give any guardian the one they live with that time be it parents or sister.

Interviewer 1:	Why did you choose that one as a proxy, we have chosen a relative staying close by, other guardians for those that are married the spouse why are we mentioning these people to be the proxy.

Participant 3:	I feel like because these people are close by if something happens, they can hurry, and you can reach them quickly.

Participant 4:	Maybe his phone is off you call the other person (proxy) he will bring the message to me.

Interviewer 1:	Ok there is another reason.

Participant 6:	The other is for people whom you stay with you trust them you can put them to be aware of what you are doing foe example when you get sick they can know where to go or who they can contact, and that if you fall sick suddenly he should not be surprised that you were doing when they are aware there will not be many questions they are already aware of the idea.

Interviewer 1:	Ok let’s continue now let us come to the other section where we want to discuss about study activities. These activities are the ones to be done in the study that we are calling it TB CHIM. Now we want to go into that section to discuss about those activities. As one way of the study participants will be injected with BCG as a way of introducing the bacteria. This BCG is the same as the one children in Malawi receive as part of immunization but this one will be in different doses to be able to collect different samples like taking part of skin from injected site biopsy to carry out test if BCG is available on the skin and testing on body immunity to BCG the explanation is clear before asking I ask a question.

Participant:	No response (silent)

Interviewer 1:	Let me repeat it here am explaining that participants will be injected with BCG which is a vaccine, I believe we all know BCG we have heard about it, BCG as a way of introducing bacteria here we mean that you will be given bacteria these are the ones in connection to TB CHIM they will be injected with BCG which will be away that will be used to give bacteria. The bacteria that will be given will be in form like BCG this is like the same vaccine children receive under immunization policy	in Malawi but this one will be given in different doses it will not be as the way vaccine are administered It will be given depending on the study at different doses to be able to conduct different tests like taking part of the skin from injected site to check if BCG is still on the skin they will also check if BCG is available in the body. How can you feel to be injected with BCG?

Participant 3:	For me I feel injecting is it’s ok because most times when receiving medication, some medications are injectable but also testing if vaccine is on the site that is ok. Taking part of skin is ok because they give medicine so that you don’t feel pain, but when they take part of skin how will it look a person will be worried that he will have a scar.

Interviewer 1:	Ok others wat are we saying on this arrangement that a person to be injected and they take part of skin from injected site to test if BCG is still present.

Participant:	After injecting vaccine, they will take part of skin once or each time they want to see you

Interviewer 2:	Once

Participant:	There is no any other way apart from taking part of the skin.

Interviewer 2:	According to research study process other ways can not be there yet because the purpose is where they have injected we want to see if vaccine is present at the place that was injected, has the vaccine really circulated in the body or is still at the thing is we want to know the reason people are still suffering from TB despite receiving vaccine what is happening that is the reason they thought of taking part of injected site. But if you can have other views how we can do it

Participant 1:	I want to lead on the plans put in place they are ok but taking part of the skin will depend on how they will cover it for the person not to have deformity.

Participant:	As we don’t know size of skin piece taken because on that others will be afraid

Interviewer 1:	How can we explain your concerns we want your views on that.

Participant:	That will need special civic education on the issue of skin part if you can ask us to take part everyone will be afraid that is the reason he asked for another options, maybe if you can educate people properly

Participant:	How big is part of the skin, will you take skin part on the same day or after some time how long

Interviewer 2:	I believe there will be some time for the injection to circulate, just to answer on the issue of size of skin sample maybe it is difficult how to explain it but skin sample taken for biopsy is very small that that you cannot hold it in hand, it’s like a prick is what we take for testing its not that when we take there will be a wound to cover it

Participant:	People were surprised people are different you find someone has joined a study they will take that telling people other people can be discouraged saying they are taking a big skin piece

Interviewer 1:	Ok we want you to help us here on how we can do it your views will help us on how we can go about how to do that the aim is that when giving information it should not discourage people.

Participant 1:	I feel like making a video like study in another we were like seeing the steps, they are putting it they will do this if people can watch at the beginning on how things will be like. Because people’s concern is on taking skin part how they take skin part that will depend on how ready a person is to take part or not

Interviewer 1:	Ok we can continue here.

Participants:	Yes

Interviewer 1:	Which site would you prefer to have BCG injection, site that you can recommend in your views to inject BCG, what can you see would be a good place.

Participant 1:	on the thigh

Participant 4:	On the arm

Participant 3:	Arm

Interviewer 1:	Others what are saying.

Participant:	Thigh

Interviewer 1:	For those that have mentioned thigh, why have you mentioned thigh.

Participant:	There is more steak unlike the arm

Participant:	Arm

Interviewer 1:	For those opting for arm why the arm

Participant:	on the thigh if you can have a scar, we are covered most of the time (all laughed)

Interviewer 1:	On the arm

Participant 3:	I feel like because thigh people resist to expose because you enroll different people other men the arm we can give in easily

Interviewer 1:	What are your views on taking part of skin where BCG was injected anything you want to add on what you said in taking biopsy on the site BCG was injected that you want to add.

Participant 3:	I feel like there are other things when you take skin part you want to make sure that you have informed people of size to be taken and make sure after taking skin part there should be a way to prevent infection.

Interviewer 1:	Ok anything to add, there are different ways of introducing BCG in the body one of the way is putting BCG in the lungs, the methos uses a pipe that passes through the nose or mouth passing through the throat up to reaching the lungs, the methos is used to collect samples from the lungs, what are your views on this procedure

Participant 3:	I feel the methos is very uncomfortable even if it can be effective because like the other option pricking the nose it was irritating this one can be very uncomfortable maybe we need discuss another way that can be used this one will scare people to participate.

Interviewer 1:	Ok other views, let us give our views help us get things done.

Participant 1:	I feel like if you can put other ways others may use the annual what because most people will be scared of the mouth or nose, they will say they put me in the mouth they can be scared.

Interviewer 1:	There are other additional views on BCG issue.

Participant 4:	We can be on a risk because we can think differently, the way one can think others can think the pipe is too big it can cause other problems there is need to find another way.

Interviewer 1:	So, another way that we can identify help us to come up with another way the way you look at it.

Participant:	I said a way like this using the nose can’t there be another better way that you can use unlike using the pipe one

Interviewer 1:	You have given views that if there can be another way apart from this using the nose other views, let us come to the other section F, your friend or relative if he can tell you he wants to join a study like this how can you feel. Here we are discussing about TB CHIM study.

Participant 4:	When he wants to participate in a study like this first, we will talk to him explaining what you read to us, it is up to him to feel if he can participate or not it will depend on the side that he wants because if we tell him, it will be we have explained and has understood. When he will be coming here when you are explaining again that can help if he is HIV he will not suffer from TB when you put bacteria to find out how it will work that can give another good option, they can enjoy how his immunity is working.

Interviewer 1:	Other views/opinions

Participant 3:	I feel I can feel pity for him because like the way of inserting a tube to go in the lungs it’s uncomfortable unless if there can be another way unless if this can be changed even though there is benefit that can help a person.

Interviewer 1:	Ok, other views

Participant 6:	I can encourage him if he wants to participate but if he is not sure or doubting a bit can tell him not to participate but if he feels he has fully accepted that he want	participate

Interviewer 1:	Why can you encourage him.

Participant 6:	Because as said at first, it’s research study that will help to find TB drugs for the drug to be discovered, when vaccine is developed it will help the whole nation to keep people safe.

Interviewer 1:	other views

Participant:	No response (silent)

Interviewer 1:	Your concerns that you may have, what concerns can you have for your friend or relative participating in TB CHIM research study.

Participant 3:	The concern that I can have is that a person receiving TB bacteria of course how will the results be in his body will he not get sick

Interviewer 1:	Apapa nde tiyang’anore zonse zija tafotokoza kuti zimene ziti akhale zikuchitika mundondomeko yakafukufukuyu yolowets kukaika mapapo yobaya kaya kutengapo kachani tiyang;anire zonse nkhawa imene mungakhalepo nayo pa nzanu kapena wachibale kutengapo gawo mukafukufukuyu ndi nkhawa yanji.

Participant:	Ndifunse kaye ndisanayankhe, nde mene mukufotokozera ndi zosiyana ndi imene timapanga ife ija yopanga ndizosiyana kwambiri kapean ndiyofananirako ndi imene tinapanga ija

Interviewer 2:	Zolowetsa mapapo ndizosiyana, ee chifukwa choti inu ngati inali ya hard inali yongotokosa mphuno osati chi pipe kakang’ono koti kakafike mpakana mkati ee kakafike mapapo ndithu

Interviewer 1:	Nde nkhawa yanu imene mungakhale nayo tikufunano mufotokoze pa zonse zimene tafotokoza kuti zikhala zikuchitika pa gawo la kafukufukuyu

Participant 6:	Nkhawa yanga ina ngati ali wachibale wanga kuti apange nawo participate mukafukufuku ameneyu nkhawa yanga ikhoza kukhala panthawi imene mwina akupanga kafukufukuyu kuti ngati akulowetsa ka pipe sadzamva kupweteka olo kapena mwina after sadzakhala ndi ma complications ena ndi ena.

Interviewer 1:	Koma poyambilira paja munanena kuti inu munthu atakuuzani mutha kumulimbikitsa nde aoa mwabwea ndi nkhawa imene mungakhale nayo kutsatieano ngati ndondomeko zakafukufuku

Participant 6:	Yes

Interviewer 1:	Other views

Participant 3:	Nkhawa yanga yomwe ndingakhale ndiyomweyo kuti ndondomeko iti ikuoneka zoti somehow part makamaka iyoyo ndiya uncomfortable basi komano mabenefitwo nde alipo

Interviewer 1:	Ndemanga imene mungakhale nayo apano tikupita kumapeto ndemanga imene mungakhale nayo pa zokambirana zimene takambirana munozi pa mene mwaonera mungakhale ndi ndemanga yotani tingoyambira apa kuti chizingoyenda.

Participant 6:	Ndemanga yanga ndiyakuti kafukufukuyu akhala oythandiza kuti tipeze mankhwala kapena vaccine wa TB amene ali matenda amene akuzunza anthu ambiri m’Malawi muno. Lomaso china pakhale njira imene ingadzatichotsere uluku ngati ululuo udzakhalepo anthu akamadzapanga ma participant kuti ngati akuwalowetsa kapipe komaso akamadzachotsa kanyama ngati pali kuthekera kuti anthu adzamva ululu tiyesetse tipeze njira kuti uluku uja adzauchepetse kuti anthi asadzakhalae affected kwambiri koma adzapange participate mapaka kumaliza opanda vuto lina lililonse.

Interviewer 1:	Ok comments

Participant 1:	Ndemanga yanga ndiyoti pamfundo zonse zimene takambirana ndikuona kuti mfundo zonse ndizoyenera ndi zoti zitithandiza kuti tidziwe zeni zeni pachimene chikumachitika komaso chomwe chikumavuta kwambiri kuti nthendayi idzikgala ikupitilirabe.

Interviewer 1:	Chabwino zikomo tiyeni

Participant 3:	Ndikuona ngati study imeneyi ndiyothandiza kwambiri chifukwa itipamgitsa kuti papezeke mankhwala omwe atapange omwe atathandize ku anthu omwe zomwe zitapangotse kuti chiwerengero cha anthu odwala TB chichepe ngakhale kuti plai njira zomwe zili za uncomfortable. Komano tingopangako weigh kuti benefit ndi yochuluka nde anthuwo afotokozeredwe njira zokuti asakhale ndi mantha kwambiri kuti aange participate kuti njira ipindulire anthu

Interviewer 1:	Your comment on that

Participant:	Ndikuona kuti nkukambirana kwathuku eti kutithandiza kuti study yathu ikamadzapita kumapeto kuti zonse zatheka makhwala ajaso kuti mpakana zatheka titha kuchepetsako kufala kwa matenda enanawa sinanaga anthu aja adzikhala kuti akuthandizidwa .

Interviewer 1:	Comment on that

Participant 4:	Speaking in low voice.

Interviewer 1:	You reduced voice we can’t hear you.

Participant 4:	Ndemanga yanga ndiyakuti apapo kumbali yakufala kwa Tb kukuonetsa kuti mwian kutha kuchepa chifukwa mankhwala eni eni mupanga zoti apezeke mukawapatsa anthuo bacreria kuti nthenda imeneyi kwa munthu kuti sinafikire msanga mutha kupeza njitra ina kuti makhwala apezeke ndikuwathandiza anthu

Interviewer 1:	Ok

Participant:	Ndemanga yanga ndiyakuti ndikupanga kafukufuku wabwino amene adzathandizire kuti zinthu zidzasinthe ngakhale pakuoneka kuti njira yake ya kafukufukuyo ndiyo opsyerabe pang’ono. Nde patapezeka njira ina yofewerako ingathe kuthandiza.

Interviewer 1:	Ok

Participant 7:	I feel the study will benefit a lot of people but on the procedure of introducing BCG I feel if there can be another way because most people will be afraid to use this method

Interviewer 1;	Ok before winding up if there is anything that you want to share or subtract from what has been discussed.

Participant 1:	On the same point if you can like your relative participating like for me I can be proud for him to take part and the people who are around for those that would want I can tell them first to take example from me they should see results they take it from me that I participated for them not to be afraid.

Interviewer 1:	Alright thank you, if there are no any additions this is the end of the discussion. I would like to thank you all for commitment to help us in the discussion today thank you very much.

Interviewer 2:	I don’t have much to say I would like to thank you all when we contacted you there were concerns that who are these people inviting us to Ndirande but you tried to come you have left a lot of things to do at home but you made it to be part of the planning team, what we were doing is planning for the studies coming in future. Thank you for your time thank you, I believe when we call for you next time you will come.

Participant:	Thank you that we were among the selected

Interviewer 1:	Thank you.
