# Supplementary material for: Acceptability of advanced Controlled Human Infection Models: The case of a pneumococcal challenge in people living with HIV and mycobacterial challenge with Bacillus Calmette Guerin in Malawi
Source: PLOS Glob Public Health. 2026 Aug 18;6(8):e0006236. doi: 10.1371/journal.pgph.0006236 (PMC13485124; doi:10.1371/journal.pgph.0006236)
Supplement: Supplementary file 1 [file pgph.0006236.s001.zip › Documents/ACHIMA_ FORMER CHIM MALE_FGD_04_ENGLISH_.docx]

Interviewer:	Welcome to the discussion today this afternoon where we want to discuss with about research study

Video play:	It’s a given that vaccines prevent diseases and saves countless lives, but when it takes ten years or more to develop new vaccines. We need to find a faster way, one way to speed up the process is by making better use of human infection studies, volunteers are given a sample of vaccine under development followed by a carefully monitor infection medical care is always close to hand if volunteers fall ill but if they stay well, it means the vaccine works and can be made available much earlier. The idea is almost 300 years old but using todays advanced technology this type of study is not just scientifically sound but ethically responsible too and of course follows strict rules. Human infection studies show huge promise against fighting diseases like Typhoid and malaria but to develop vaccines most effective at the people at risk we need to test on those at most risk by expanding human infection studies and targeting them better the people most at risk could help develop vaccines of the future and save millions more lives.

Interviewer:	In short, it’s explaining that it’s known that to develop vaccine takes a long time for them to come up with vaccine. But using the study approach the one you participated o pneumococcal it helps to develop vaccine faster. For example, we can say how Covid vaccine was developed, Covid vaccine did not take long because after developing it was tested in people who volunteered who were not sick it’s the same as this approach in the study. The approach has shown. This can be a faster way to develop different vaccines to use. Our discussion this afternoon we will look at giving bacteria to people with TB, I will ask you to feel free to share your views/opinions on how you understand what I have explained for today. Getting here we can start the discussion.

Participants:	All we can start.

Interviewer:	Firstly, just want to know on what I have explained today what message you have grasped or heard

Participant:	Aa, from my side that message

Interviewer:	Mention your number

Participant 5:	On my part what I have heard from what you have explained, you want to hear our views mainly about vaccination in the people who are on a risk with other diseases they receive it as part of the bacteria.

Interviewer:	Ok, others what message have we got if someone ask us where you went what did they tell you

Participant:	We can say that Malawi Liverpool Wellcome Trust

Interviewer:	Number

Participant 1:	Ok, Malawi Liverpool Wellcome Trust is continuing to conduct research they want to develop vaccine for different diseases so, you have just sampled out people who participated in pneumococcal first study thus pneumococcal bacteria, the people who were enrolled at first were healthy they did not have any illness/disease they were given the bacteria. After they were given the bacteria others were given vaccine others were not for those who were given vaccine it proved that maybe they are ok so you want to do the same bacteria study, but you want to try it in people suffering from TB those people whose immunity is weak unlike those who participated the first one that is what we have heard.

Interviewer:	Ok, what are your views on a study like this that you want to conduct in people who had suffered from TB or those suffering from TB

Participant 1:	Ok, I see it’s a good study when we take on the part of those people working on to develop the vaccine it’s good research because it will help to come up with vaccine that will help to save more lives in future that is to the part of those conducting the study. In this case we are talking of Malawi Liverpool Wellcome Trust. But on the part of the people who want to participate in this study I feel like somehow its risky because we are talking of people whose immunity is low because they are already sick suffering from TB at the same time you want to test on them to give the bacteria to see how they are going to survive. I feel like on the part of people that are to participate there is a risk considering the study in general I feel like there can be several risks that can be there unlike what you come across in the first pneumococcal study. When we look at the first study, I feel like you considered literacy levels the people who participated in the first study were people who could understand English, they can read and write we were saying that. Now we don’t know those who will participate in this study is someone suffering from TB at the same time he has been to school. I feel like considering literacy level in this study will be difficult. Another thing for selecting people who are educated is that these people are easy to understand in other words they understand things quickly so, when you do with any other person others are willing to participate in the study, I feel that part can be difficult because a person who is a right candidate can refuse simply because of low literacy level just to answer in brief.

Interviewer:	Let’s go on we can pass on after the other one has responded, you can continue

Participant 6:	I don’t know if its good to try the study in those people who had TB not the ones that are currently sick now, but they already had and they are well because they have experience in TB that can help those developing vaccine to see that this one had TB, I mean will they have immunity. It’s different from testing it on healthy person test on them TB that will not be good the thing the study is benefiting those doing it for them to know but for the patient the benefit is not clear to them it will be like he has just sacrifice for the people that will benefit in future my opinion.

Participant 5:	Number 6, no 5 I see that as explained by number 6 saying this is good, but they must take those people who have had TB, and they are healed for those people but for the people who are sick those they have to benefit from the vaccine that will be developed from the study. It will help to say medical personnel to know to say if a person has TB as well as the bacteria in the body how strong will his immunity be like if immunity will be weak how will the vaccine help this person. I see that can be one way to someone who is had Tb and is fine that cannot be good to someone who is sick.

Interviewer:	Ok

Participant 3:	I feel like the idea of focusing on those people who are already at risk in terms of body immunity and those suffering from TB on that point you cannot try doing something on a something that has not being on that problem to find solution you need to get to a problem in this case for those with HIV, as well as are suffering from TB to find vaccine they have to participate

Interviewer:	Keep on talking someone is calling

Participant 3:	They must participate, and it will help to know the exact problem or solution that can be identified from the samples that can come from these people those that have contributed mainly to the research study.

Participant 4:	To add to the point said by number 3 it can be difficult because of course one person is suffering from two illnesses let’s say has TB and HIV, it can be difficult at the same time receiving this bacteria the risk can be there maybe immunity is already low because the person is already sick suffering from two illness, unlike the group that participated in the first immunity has to be different considering that they are on drugs which they take at some point. So, for them to get the bacteria there are some days may experience something because he has received the bacteria, and that body immunity is low I feel that can be a problem than a person who is healthy.

Participant 2:	Aa I want to ask for something that when trying the vaccine in people suffering from TB or those with HIV, request to say people to participate should be less than 18 years limit 45 why am I saying that 50 , 70 years a person if they are found with TB a person is going towards low immunity, immunity is getting low because if you take someone 50 years to try vaccine on you they can be on risk that immunity is low has TB or HIV and they are suffering, old age things become difficult. When you try on people 18 – 45 years yes there will be some who will have HIV, or TB but they are still strong. Request when conducting the study these should be people who are suffering from TB, HIV I feel it will not be wise enough to test on people who are sick because he is the one whom you have given in his body it means you will have real information about the vaccine that you have given to people who will be found with TB the people will get healed

Interviewer:	Ok, thank you very much let’s continue the discussion we have discussed about the message that we got on the study that is to be conducted, anything to add o what has been discussed

Participant;	Silent

Interviewer:	So, we can continue, I just want to know which message you do feel is the most important on what has been discussed about the study

Participant 3:	The most important message is for people to volunteer participating in the study with the aim to develop vaccine.

Interviewer:	Others

Participant 6:	The main thing is results of this study will being a good impact to other people if the study goes well the study can be recognized and vaccine will help to save lives in future.

Interviewer:	Mhhi

Participant 4:	Just to add on the point that number 3 raised it will help mostly those people that are suffering from TB when they get the bacteria and see if they don’t survive or how they survive and then they can find a way to help these people I want to add on that.

Interviewer:	Ok, anything else

Participant 7:	When giving information maybe there should be understanding for people for them to be free and secure in the process of the study, they should see that there is no risk.

Participant 5:	Maybe on the issue of those people participating in the study they have to understand the main aim of the study that it will be useful not themselves even to other people who will be affected by the same problem for them to get help quickly so that they will not suffer with the disease.

Interviewer:	Ok, other views

Participant 2:	Request to say when starting the study when inviting people to join this study there is need to engage people, you said you will go to villages you will meet with chiefs, religious leaders it should be like from sample one person who received the first vaccine for the people not to fear in that when they give vaccine maybe it’s not for TB they just want to give any other vaccine because people in the villages they don’t understand that we are conducting a study, but for someone who participated and received this vaccine	that one can be an example that after receiving this is how I felt the effect I did not fall sick, or it was like this you can find people easily to join the study. But without doing that to tell a person to come for the study to test vaccine on them when a person is already suffering from TB or HIV people will be afraid that they will infect me with a certain disease.

Interviewer:	What message do you feel is important or helpful, help us to add other information or maybe what has not been mentioned that is important that if incorporated when giving information that can be helpful that perhaps understanding that is mentioned when informing people

Participant1:	Ok, I feel when you will be giving information about this study or don’t stress much on incentives or getting something. Let’s give an example how it is on blood donation people are asked to go to donate blood at the end they are given something it’s not like selling blood. So I feel emphasis should be on volunteering to help study that will help to come up with vaccine issue to do with incentives should come later but if you put it forward in the information that you will receive something therefore you have to join study I feel there will be misinterpretation you will have very few people that will be interested to participate in a study like this. Its’s what I was saying about level of understanding among villagers maybe you can target them now they cannot understand so I feel on that your messages have to be drafted properly for people to understand that it’s not selling samples but helping conducting research.

Interviewer:	Ok, other views

Participant 5:	The issue is making a person to understand to come to join study a person who’s healthy is already weak, I feel that can be a problem. Its work that if you leave it to one person cannot manage it if you can do as you have said chiefs, religious leaders and other groups have to be involved with these activities for them to inform people to join the study.

Participant 4:	To agree on what number one has said when we take people from the village when they hear vaccine they can think if they did not inject us during Covid it means they want to tell us lies, the issue is that they want to do a study they want to give us Covid vaccine. So, it just depends on the approach that you use to get people in the villages reaching out to chiefs that the vaccine that we are coming with, vaccine that we are coming with is not Covid vaccine this is what we need, we want to check healthy of these people. The approach that you go with on the ground it can help a lot considering that people can be attracted to participate when I see that after getting it how will I survive because people in the villages when they hear vaccine, they think its Covid vaccine it can happen like a lot of people will be afraid to participate.

Interviewer:	Ok, want to know what questions you have on this issue or maybe questions that you feel they can be there related to the study

Participant 4:	For people from the village which are being reached or we can answer the question right here.

Interviewer:	Here we are asking on your part questions that you can have it can be on behalf of the other side or can be both sides for you and the other side

Participant 6:	My question is going to you apart from informing community leaders what other ways you will follow to reach out to people to inform them about the plan to set up study.

Interviewer:	So, the other questions will answer them at the end because the other questions are part of the questions that am going to ask so I will be like

Participant:	Oo, you are answering

Interviewer:	We should do that, I will just record it ok there is the other part of questions that you can ask for the other side asking for those people that will want to join the study

Participant 3:	In this study the bacteria that they will be, let’s say it’s one person my immunity is low in my body we know that their body is sensitive when they are given the bacteria it will not affect other areas.

Interviewer:	Ok have recorded the question

Participant 5:	Maybe a person who has TB or HIV has joined has received these bacteria like study we take it for example the bacteria in the body as it has been said it can go in a very sensitive body that if something serious happens on their lives that it is difficult to control for a person like that how will you help them

Interviewer:	Ok, this one I can answer briefly as it was in the study which you participated, they explained when there is any problem you have to come to clinic for the study for them to see you, for this study it’s obvious it will be the same, a person has to be seen by the study from the time a person has joined, let’s start from the time he has joined the study the person will be looked after. By the study

Participant 7:	Maybe the other question can be previously when we were doing that study, they were conducting tests to confirm how the body is for one to qualify to participate. So, on these issues what level have you set for those people that are HIV or have TB what level have you set or condition where everyone is or there is a condition.

Interviewer:	On the issue that you have asked let me respond to it that there will be procedures in place checking on condition of people joining they will make sure those that are joining let’s take for example those people who have HIV their CD4 count they will say those having CD4 count limit is so when CD4 is higher or lower than that they will not be allowed to participate, that will be in the procedure to make sure that the people that are joining should not be put on high risk when its people that are within the range of someone who is not sick he is ok regardless of the condition that he is in but that he is not sick at that particular time, they have to do some other test will be conducted to follow up to make sure that they are not giving problems to people joining the study

Participant3:	The study that we were doing at the beginning let’s say a person was on medication, is taking any medication you were not allowed to participate so in this case for our friend who have HIV it is obvious that they are on medication this will not affect the study.

Interviewer:	That is obvious that doctors will look into that properly on the plan to ensure that there will not be conflict on medication that the person is taking during the time a person is participating in the study. Those are some of the things that are important that need to be looked into properly with professionals on that.

Participant 5:	Mainly I want to ask for a person who has joined the study you have given him bacteria when you see that the person, the bacteria entered his body and then you find out that sorry am missing out some points wait two minutes.

Participant 2:	A person joining the study how will they know if the vaccine they have received, have worked or not.

Interviewer:	Request we are like, instead of starting the discussion questions are coming to answer them instead of you responding to questions, we will be take more time, I want to ask the questions that we have can we keep them to the end of the discussion we want to hear your views on how you feel when we dwell much on the questions that we have it will be like what we wanted to discuss we have tackled them in between when the questions are coming the answers will come from what we have discussed.

Participant 1:	This time around the study is going to take place around rural areas for a village person some may ask because let’s not forget that we are administering vaccine there. This study for a person from the village can ask why leaving out people	from the urban or people from town coming here in the village you want to kill us, some questions will come like why choosing people like us some questions will come like that.

Interviewer:	Ok we can continue with questions will do at the end like that. Just want to know, help us enlighten, which another additional message that you feel is important that when conducting the research, we should incorporate it on the message that we will be giving out to people.

Participant 2:	The messages to go through radio, newspapers because it can be easier for those people that maybe don’t listen to radio, they can buy newspapers for the message to be easily accessed it can be like that.

Interviewer:	What messages should go to newspapers and radio	stations.

Participant:	About the study that there will be a research study to have clients, to have people who can understand this research to have more people joining.

Interviewer:	Mhi, another important message that we need to add when giving information to people

Participant1:	Research study for different diseases helps to come up with vaccines that can help more people in future people have to be prepared to support for vaccines for different diseases to be available just to emphasize on that I think it can be an important message to give to newspapers and other media mentioned.

Interviewer:	Ok

Participant 7:	Maybe on number 1 on the same point if there can be like history saying that sometime there was this and we did this so when you do this you will help other people in future for people to take it that what they are doing helps others some people did that, and we are enjoying it

Interviewer:	Mmhi,

Participant 3:	Aa first we need to explain to people about what our organization does, second you need to teach people that there is a research study that you want to conduct, and what the study is involved in. You also need to teach people about benefits of participating in this study.

Interviewer:	Benefit of participating in a study, any additional information that you can add on when reaching out to people or when explaining to people anything that you can add on

Participant:	The main things are what have been mentioned just explain clearly on benefits of the study in the benefit will be this maybe few little things that can come in their bodies if they can participate in the study, like those joining our study you were explaining that in other countries after receiving vaccine you mentioned swelling of tongue and the like small risks that people can face when taking the vaccine they should know both sides.

Interviewer:	Ok, they should know both sides.

Participant:	Yes

Interviewer:	So, lets come on it as it has been raised, what do you think will be the benefit of conducting this type of study. To start with benefits after we move on the other side.

Participant:	Silence

Interviewer:	Benefit can be their benefit will go to flat both sides

Participant 3:	The benefit that can be there one vaccine will help to aid development of vaccine faster and easier.

Participant 4:	Aa, it can be a good thing considering that if the people participating in the study if there can be any problems in between the study that can cause people conducting the study to find a faster way on how to solve the problem that is affecting people participating in the study at that time.

Interviewer:	Mmhi, another benefit

Participant:	Silence

Interviewer:	Another benefit that can be there

Participant 6:	The benefit just to say if the study goes well vaccine is available it will help saving many lives for people not to suffer from TB and HIV

Participant 5:	The other benefit is when a person get full information will have interest to join the study to support development of vaccine faster.

Interviewer:	Ok, there are other benefits to add on, let’s come on the part of risks that can be on this study, do you think there might be risks or problems.

Participant 1:	Aa in terms of risk on the part of people conducting the study there is a risk they can face hostile environment because the places they are going there is a lot that is said misconceptions, many misconceptions related to blood. Our study that we participated in, first study and this one it will mostly involve sample collections when people hear you are going for a study like this especially in a community that did not understand chances are high you can be attacked, because they can see that you are doing unethical things so, I feel that is the first risk for people conducting the study.

Interviewer:	Ok, another risk.

Participant 3:	Another risk that is there is for participants participating in the study they can face other complications like body reactions depending on the bacteria that they are given.

Interviewer:	Participants can face with reactions there is another risk that can be there

Participant 4:	Just want to add on the point number 1 has said it can be risky if there will not be a good approach reaching out the people in communities of course understanding is different how I can understand as one person who participated in the first study with whom you are just approaching now, how they can perceive there will be two different things for that one he will be taking the information for the first time will be thinking if these people are coming here what motive do they have, what reasons do they have coming here to conduct a study like this maybe they want to take something from us. Thinking of people in the villages, they can think instantly about something that the thing that is coming can have benefits after conducting study in their community that can help everyone young or older that can be helpful at that time. These people who joined study we have done they faced this and they come with solution to do that will go to all people.

Interviewer:	Ok still on the same issue let’s look at the benefit which benefit do you think can be there for those people with TB we will be separating because it’s going together but still, we will need information to be specific on the side of TB that is the benefit what benefit can be there

Participant:	Rephrase the question.

Interviewer:	I was asking on benefit that can be there conducting this study in people with TB, what benefit that can be there

Participant 1:	Ok benefit is on developing the vaccine you will have a clear picture about vaccine that has to be given to people with TB, what they should comprise of unlike just developing general vaccine fitting healthy people and those with low immunity that is different. This one you will have a picture that this one is special for these people.

Interviewer:	Another benefit

Participant:	Silence

Interviewer:	The risk that can be there for people with TB how could it be

Participant4:	Maybe I can differentiate depending on that maybe we take this this one has received TB vaccine how that one can react and how a healthy person can react it can be different because this one his body is on other drugs to survive considering has already TB attack just thinking maybe another way can be that.

Interviewer:	Ok, there another risk that can be there for TB before we go to HIV side

Participant:	Silent

Interviewer:	Let’s come to the benefit that can be there, the benefits of doing this research study in people with HIV

Participant3:	Benefit can be there is that it will facilitate to find vaccine for HIV disease.

Interviewer:	It will help to find vaccine, another benefit.

Participant 2:	Another benefit it that, when the vaccine is available for HIV these people will stop receiving tablets, they will be using vaccine.

Interviewer:	Potential risk that can be there

Participant 6:	These are the same issues that if people receive the vaccine in their body will it not cause other problems

Interviewer:	There is other risk that we can mention or benefit in general that anyone want to give in addition to what we have discussed.

Participant 4:	Just to add of course I heard that the drugs are strong when vaccine goes in the body it is also strong and they can tell you to stop taking any medication at the time of vaccination. So, I don’t know for people with HIV and then has received vaccine the time they take hospital	drugs can see there can be a risk considering his health of course there will be reaction vaccine to work and the drug they take on daily basis, just thinking I can see there can be some sort of reaction in the body.

Interviewer:	Ok, balancing the risks and benefits, which part is weighing more than the other when you look at the two sides.

Participant 6:	Where there are benefits outweighs if they can develop vaccine it will help to save lives of more people in future.

Interviewer:	Others, everyone to weigh on scale benefits and risk which side overweigh the other just looking at it.

Participant:	The side that I have seen outweigh is on benefits

Interviewer:	Its benefits your number

Participant 5:	It’s benefit that outweighs.

Participant 2:	It’s benefit.

Participant 7:	Benefits are more than risk.

Interviewer:	Other CHIM, research on this type have been conducted here in Malawi using pneumococcus in health people how can you differentiate that research and this research to be done in TB people how can you differentiate

Participant 2:	The difference is that most vaccines are given to healthy people and this vaccine that is coming is for people with low immunity in their body because if we give vaccine to people with TB or HIV how will it work for person who is healthy is not supposed to receive vaccine because the vaccine will not working in the body but there is no bacteria in the body.

Interviewer:	Other views on difference that will be there conducting research in healthy people and conducting research in TB and HIV on differences that can be there.

Participant 6:	The difference is that there will be more tests in HIV, yet this will not be case you will already know that these are already affected.

Interviewer:	There will be more tests, can you mention the tests.

Participant:	HIV, Covid 19 the other tests help me

Participant 4:	To support point raised by number 6 there was nasal wash, taking of blood samples, other samples that are obvious that can not be done on TB or HIV unlike those people who are healthy.

Interviewer:	Ok

Participant 5:	Maybe the difference can be these ones who were healthy they were not on any medication but these ones when they will be joining the study, they will be on medication already, they are on treatment that will be the difference.

Interviewer:	Ok there is another difference.

Participant 1:	When we weigh risk, we can say risk in healthy, I can say ones that are on high risk the ones with HIV or TB compared to the ones who were healthy and participated in the study. These ones are at the risk of having more complications unlike healthy as explained, it’s not that they take the drugs are the ones that are affected but here chances are very high that people will be affected.

Interviewer:	Let’s come on what we are explaining about risks in Chichewa we take them as risk we just want to help each other how we can explain the risks to people when giving the information.

Participant:	Risk that may come on their life.

Participant 5:	Ok, potential risks that can be there or.

Interviewer:	Just to say in general risk that can come this can come directly to individual or across everything that can be there anything bringing any threat.

Participant1:	Ok so, you mean information going to the people.

Interviewer:	Yes, which message when those people come.

Participant:	You are explaining to them.

Interviewer:	They have not joined

Participant:	When explaining to them

Interviewer:	In terms of helping each other how can we paraphrase risk information when informing people, how can we give the message, I what better way can help us to deliver the message about risk in order not to scare people with certain risks in order not to scare people much.

Participant 6:	Emphasis must be on benefits first explain about benefits at the end you should say however risks are inevitable you will, you will explain of minor risk at least that will carry weight.

Participant 2:	Benefits or risk, the benefit that you will give to people from the village maybe you should have drama group that they can give as example that among the drama group some can be doctors, laboratory demonstrating like they are taking your sample or like injecting vaccine.

Participant:	But maybe explaining to them risk/problems that can be there because you have done your research problems that can be there are these ones you will take all the responsibility will be on your side that if anything happens you will handle it you tell the people to understand

Interviewer:	Ok, there are other opinions/views that we can explain about risks that can be there. So, want to know your friend or relative if he tells you that he wants to join a study like this type of study that we are talking about HIV and TB how can you feel about your relative or friend joining this type of study how can you feel.

Participant 4:	On my part I can take it as a good thing considering if vaccines will be developed after conducting the study that can help other people that can just take the thing as something has just come in the community people have to take part that some people are testing something they should test on them. Because some people can take it as something that they have to participate they say they are giving money I can participate giving the picture we have not just come in the community the aim of this program, our aim depending on the people that are participating for them to get the right message to other people close to them to participate.

Participant 7:	If it was me someone telling me he wants to join this study with my understanding I can accept knowing that he has done a good thing. Maybe I should take it as someone who don’t know a person brought me the message, I will have many questions as I will need to understand what is the issue. The message should not only go to locations/areas that we go but if the message can go to all other areas that even when you get to the community they should be aware that these people we heard from radio, we saw on the screen, or we heard some other time announcing they should have idea when a friend tell them that am doing this they can understand that what am doing is this this is the benefit

Interviewer:	Ok, others what are we saying, or we have no side

Participant 6:	I can encourage him to come to the study I can give him example that I participated in the research study and finished well there are risks that people talk of but in the end, I finished without any problem.

Interviewer:	Why can you encourage him

Participant:	I can encourage him

Interviewer;	Number

Participant 6:	In future there will be benefits if the study can be successful, results of the study they can develop vaccine that will help a lot of people if you can explain that properly.

Interviewer:	Ok other views we are all giving positive responses maybe because we participated in the study that is the reason, we are giving positive there is no one with contrary opinion with others. How did you feel participating in this study?

Participant 7:	I think for me I was in the first group it was scarry because we did not have people to tell us that others participated that is scaring if something happens what can be done. I remember some other times you can feel like the body is something, but it was just thoughts because you are afraid for the understanding that you have been given something that is not part of you have the feeling that what if something happens. Sometimes I could think to the extent we were told that if something gets worse the door is open at Mwaiwathu I could think of reaching the status of going to Mwaiwathu could think of what conditions are they anticipating that a person can reach. So, I think we can support the people in case something goes wrong, I think our approach or response is what I think affected me, what affected me most was up to reaching the point of going to Mwaiwathu that means am critically sick to what level. I think if you can be giving people understanding that they should not reach the point that things will get very serious.

Interviewer:	Ok, other views.

Participant 1:	Yes, maybe just extension to what number 7 said, personally it was dangerous because I had to consult several people some of my friends who that time were about to start practicing medicine, one of them told me not to do that that’s childish what are you going to benefit, I was like hiding later on I said there is something. He told me don’t sell your life because of a small amount of money don’t sell your life as if here we take away life in search of money it was difficult of course complications (risk) as we go here and there were there so to consider our relative who is already sick, I feel that is risky to some extent.

Interviewer:	We want that risks to come out clearly here we are discussing to have views, views can be positive or negative all are useful.

Participant:	A person is sick we test on a person suffering from HIV Aids, TB the person is so desperate for permanent healing you go there that you want to do research on you are not giving a solution that he will get healed. It can be possible that when conducting this research, you can add other problems to existing one’s immunity is low you are introducing bacteria the person can die easily, maybe he could have survived long but simply because things are too much in the body, he can die sooner than how it could have been in the first place.

Interviewer:	Go on with concerns that can be there we take someone, lets picture someone be it with HIV or TB picture someone undergoing a study like this, what concerns can you have that our relative or friend concerns that we may have express them, what concerns dis we have put them the other side for us to hear.

Participant 6:	Worries can kill because I can remember after taking vaccine in the evening had a headache, I was saying will the problem I will not pass on. Considering someone who is already sick they may start blaming us at the end of it too many worries he collapses the issue being some health issues in the body, we can take that a person can collapse with BP thinking that have made a wrong decision joining research study.

Interviewer:	Ok,

Participant 7:	I think the other concern will be as raised by number one that after telling his friend he gave a response not to take part for someone already sick putting himself on a certain risk think of the people other people can put the blame that if something goes wrong on his sickness you will handle on your own so that is the problem that is a potential problem that can be there, they can be blaming you that we have been struggling with hospital bills we are the ones paying them you are doing this. When you start other problems when somethings go wrong who will support you, so I think on that there might be some sort of treatment.

Interviewer:	Ok before we move on, there are others with additional concerns.

Participant2:	Concern can be to me for a person who want to join the study if he faces risks in his body as said by my friends when he pass on I can be on risk that I have sacrificed my relative’s life the concern can between my relatives and me we can’t look each other in the eye with my relatives my security will be compromised that I have sacrificed my relative. Because he has faced a problem due to already compromised immunity has been given vaccine the person has passed on or person getting critically ill up to getting to hospital the one giving the information will be on a risk with people from the village my relatives saying you have sacrificed your relative

Interviewer:	Let’s come to an end here we want to hear comments let’s just start here everyone will share comments on everything that we have discussed your opinions/views on arrangement of the study. What comments would you have. Can you say this research can be done, that it’s acceptable to be done in these groups or not, the comments will go in line until we get here?

Participant 7:	My comment will be based on the experience I have had during this research I think it is something important for the study to be conducted, because what we are looking for is not for now, we are focusing on the future. We are eager that in future we will have people who have overcome many diseases, that is important that current problems are overcome or that they are overcome and that there is a suitable way vaccine. so, I feel without considering that there are many risks or other things but a study like this is important to conduct.

Interviewer:	Ok

Participant 3:	My comment is the study is important, the study should involve people who are HIV and those suffering from TB because they cannot give let’s say a person suffering headache LA which is malaria drug to find solution for HIV and TB people suffering from these are to be involved in participating. On time of participation these people need to have their witness in case something comes up

Participant 5:	My part I can say the study has to be conducted this will be useful like to the whole nation the nation will benefit especially if the research comes up with solution that people are lacking that will be good for the research to be conducted

Interviewer:	Ok

Participant 1:	Maybe just to conquer with what my friends have said already it is good for the study to be conducted the benefits are more compared to the risks that can be there. What I can request that can change or to add to Malawi Liverpool Wellcome Trust if you can consider it. I don’t know the place where you will be doing it, will you be going in the communities and start the study right there and complete or else people will be coming here like your office premises like we used to do. If it will be like we did few things that can be addressed especially when we go to Grace Bandawe you order food we don’t know how much it cost, a person is forced to finish the whole amount reaching the point of taking some packed meals that they don’t take simply because they want to complete the amount that has been paid for. I feel like if those costs are considered for people to take remaining amount of money, it will be better to add that to hard cash that they receive, food staffs you order at reasonable price the other money to give people to see what they can do because to be honest it was not good.

Interviewer:	Thank you very much, comments feel free we want to hear your views

Participant 6:	Ok many of our friends have commented already about the research, the research is good must be done just to prepare when reaching out to people you need the right message that’s my comment.

Participant 4:	The research has to be done considering that maybe other vaccines that we are getting now passed through the same process other people volunteered to take part (sacrificed to take part) by testing other things in order to find medicine to heal people in times like this. We can only ask the research to find people with side effects in their body to see how they react to the bacteria because at first you were taking people who were healthy in their body the main thing the research has to be conducted.

Participant 2:	To add on what my friends have commented the research has to be carried out urgently to find solutions. What I can request from Malawi Liverpool Wellcome Trust your studies mostly are conducted Monday – Friday most people who you invite you can call people to come in the morning 8 o’clock or 2 o’clock same time a person has to report at company for work, our request if you can consider Saturday and Sunday most people are home we take that like in the first study we were called while at work to get permission from our bosses people were at risk at their work places that every Wednesday he leaves at 2 o’clock. It’s a request if Saturday or Sunday everyone is free or working half day that’s our request.

Interviewer:	Ok, thank you other additions.

Participant 5:	Question we are discussing about bacteria in people with TB the solution that might come will be for bacteria or TB.

Interviewer:	Ok thank you for the discussion we had this afternoon that is important in our last part of questions, there was a question in terms you wanted to know like approach that will be used in the research, will the research be conducted in communities, or it will be done at Queens. There is another question about type of bacteria to be used the bacteria will be like the one used in TB vaccine, I believe everyone who is here heard that there is BCG vaccine given to children for TB.

Participants:	All yes

Interviewer:	That is the one that will be used, will be BCG which is like any other vaccine which we claim that anyone else received this type of vaccine, it’s a vaccine that leaves a scur here it’s the one that will be used, it’s a bacteria that is used to develop vaccine but it will be in form of vaccine, there is another question

Participant:	Yes, the question is the one I asked the bacteria is given to someone with TB coming in form of vaccine, vaccine will be for bacteria, or it will be for TB

Interviewer:	Here when I was responding on BCG, this is the vaccine that is available and is in use, they will use like the same form they are in a weakened form it’s in form of vaccine they will use the type of BCG vaccine that children are given when they go to under-five clinic, it is clear that all of us here received vaccines and this is one of the vaccine that we already received in the past

Participant:	Ok for me of course I don’t understand.

Participant 1:	Ok I feel like the question that he is asking is study has been conducted then you have developed vaccine will the vaccine heal TB, or it will not heal TB, but it will help protecting from TB or will help protecting pneumonia.

Interviewer:	In terms of TB every vaccine that we receive it protects from diseases the body is like has immunity that can fight infection when it attacks, meaning to say on the part of TB the body will be like prepared immune to TB that is clear

Participant:	Yes

Interviewer:	Before I close maybe there are others who want to share anything

Participant:	I asked a question, but you didn’t respond

Participant:	Maybe she has forgotten the question, I asked your experience friends

Participant:	That has been answered already on what we have discussed.

Participant:	That questioned has been answered.

Interviewer:	Thank you very much this the end of our discussion this afternoon. Thank you very much for your dedication to come at this place I don’t take it for granted. I appreciate for your dedication to be together to help us answering questions giving your views/ opinion on this plan.

Participants:	All thank you

Participant:	Ok after this will we come back later, or we are done

Interviewer:	On that I cannot say it that we are done if there will be anything like that, we need you, we have your contacts the same way we contacted you we can get in contact when need arise. There are other things that we cab be discussing as the signing sheet is moving on.

Participant:	Number 6 asked question that when you are joining the study you were ok HIV negative, no TB with passage of time reaching the year 2024 you are found to be HIV positive are you allowed to join the study

Interviewer:	That question I will not be able to answer as the protocol that will be used has not been developed, it can be difficult to answer that question. On the other hand, those who will make the plan it will be done for someone who already participated can join again as long recruitment criteria have been meant.

Participant:	Last thing for those people that will join the study those who are HIV some will be married it can happen that one is HIV the other is negative for someone who has been given vaccine they want to be at the same place as a family what protection will you give the other person after receiving the vaccine follow this as a family

Interviewer:	This one the same as the other as we are just preparing most of the arrangements will be done when all is in place this is what will be done, what we will be informing people, we are at the beginning its difficult to say we will follow this procedure this is what people should follow during the time of research.

Participant:	Maybe just to ask the time we were doing the study we were told that they will share results, I don’t know if this has been arranged already that last time study these are the results or

Interviewer:	About last time study, after a study has been done it takes time for reports to be drafted there are several steps when everything is ready the results are set, and final copy has been done there is what we call dissemination meetings to inform people about the results of the research. One of the ways that is used is engaging those people who participated in the research if they are still in the area people are called to tell them the results of the study, but it takes time to finalize everything. However, results of research study should be known to relevant people those that participated if they are within the same area where the research was conducted. If there are no other things to discuss thank you for coming this afternoon

Participants:	All thank you very much.
