# Supplementary material for: Acceptability of advanced Controlled Human Infection Models: The case of a pneumococcal challenge in people living with HIV and mycobacterial challenge with Bacillus Calmette Guerin in Malawi
Source: PLOS Glob Public Health. 2026 Aug 18;6(8):e0006236. doi: 10.1371/journal.pgph.0006236 (PMC13485124; doi:10.1371/journal.pgph.0006236)
Supplement: Supplementary file 1 [file pgph.0006236.s001.zip › Documents/ACHIMA_ IDI_01_TB SPE_CG_20.02.24.docx]

Interviewer 2:	So, I think we can re do this at the end because we were supposed to fill this space, I don’t know if I can give you another one, which one maybe you can do the last one rightly.

Interviewer 3:	So, this is SBC.

Participant:	Yes

Interviewer 2:	To fit here.

Participant:	That’s a big challenge for doctors

Interviewer 3:	The writing

Interviewer 2:	Maybe the surname can be down.

Participant:	Yes

Interviewer 3:	So, he will keep this one we get this one, sorry date is?

Interviewer 2:	20

Interviewer 3:	Participant ID

Interviewer 1:	Welcome to this interview this afternoon, in which we will be discussing controlled human infection model CHIM in TB as in TB challenge. We a re MARVELS group from Malawi Liverpool Research program conducting a controlled human infection study, we plan to extend our human challenge work to special agents such as Tuberculosis, which is TB, your views on the planned work will inform future steps regarding such studies. Have you ever heard, read or seen anything about controlled human infection study.

Participant:	Yes, we actually have been involved in one of the research projects I think it was directed by Dr our friend he came here he said he is conducting research within Blantyre, and he said some of the clients might be coming so we were prepared for the coming of the clients. So, he briefed us for some time on the study.

Interviewer 1:	This one was working for these CHIM model studies.

Participant:	Yes

Interviewer 1:	This one was working for CHIM model studies.

Participant:	Yes

Interviewer 1:	As he was a doctor.

Participant:	Yes

Interviewer 1:	So, time you met him did you work with him, or you were part of the people you were working with

Participant:	If you can come back on that one, I think he was from Wellcome trust, so he briefed out team he said in case the clients will develop some reactions he will be bringing them over, so I don’t remember seeing any clients.

Interviewer 1:	Ok, you will watch a small animation video that Is explaining more about this type of studies.

Video play:	It’s a given that vaccines prevent diseases and saves countless lives, but this takes ten years or more to develop new vaccines. We need to find a faster way, one way to speed up the process is by making better use of human infection studies, volunteers are given a sample of vaccine under development followed by a careful monitor infection medical care is always close to hand if volunteers fall ill and if they stay well, it means the vaccine works and can be made available much earlier. The idea is almost 300 years old but using todays advanced technology this type of study is not just scientifically sound but ethically responsible too and of course follows strict rules. Human infection studies show huge promise against fighting diseases like Typhoid and malaria but to develop vaccines most effective at the people at risk we need to test on those at most risk by expanding human infection studies and targeting them better the people most at risk could help develop vaccines of the future and save millions more lives.

Interviewer 1:	This video was explaining more about what happens in like a CHIM model study, can we move on from there.

Participant:	Yes, we can go ahead

Interviewer 3:	you want to listen again.

Participant:	No, its enough

Interviewer 1:	Having watched the video to what level would you agree or disagree to conduct a TB CHIM in a health adult volunteer, strongly agree, agree, neutral, disagree, strongly disagree don’t know or undecided.

Participant:	Agree

Interviewer 1:	You agree, in your view what could be the benefits of conducting a TB CHIM in health adult volunteer.

Participant:	Mm, that will be important especially with set of Malawi where TB rates are quite high. And we are the most affected especially local the poor local majority are the ones mostly affected especially the urban. But the only challenge will be how to care for reactions for those who will have some reactions of the TB that one might be a challenge.

Interviewer1:	In your view what are the risk of conducting a TB CHIM in health adults like what can be the risk.

Participant:	The risk would be I think I have already started talking about it the risk will be the care. Post affection care because our health facilities are not in good shape across the nation so that will be a big challenge. But if we have established facility that can care for those who are taking participating in the study that will be welcome idea.

Interviewer:	In your view what other concerns could arise conducting a TB CHIM in health adult volunteers

Participant:	The other challenge would be the serostatus of the patient because some people will not be able to disclose their serostatus and they can be challenge with TB and if their immunity is on the lower side possibly, they might have more serious replications that the rest. But knowing the HIV status of the participants will be a welcome idea to avoid some of the implications.

Interviewer 2:	So let’s say we are saying that in this study we will only recruit those who are HIV negative and let’s say one of our, on the screening day, the day we invite them before enrolling them in a study we are offering them an HIV test to confirm that they are negative

Participant:	Yes

Interviewer 2:	Do you still there will still be concerns to conduct a TB CHIM maybe we can say in HIV negative health volunteers. Will there be any concerns?

Participant:	I don’t think so not much now coming to my mind is the issue of Diabetes we know people with Diabetes can be at risk of getting TB easily but that will be a minor one, the main one will be the HIV status.

Interviewer 2:	Ok, having looked into those issues by excluding those who have long term diseases like Diabetes, Hypertension those who are HIV positive do you still there will be concerns if we would involve people who are health, we have ruled out other risk factors will there be any concerns.

Participant:	There shouldn’t be any concern

Interviewer 2:	Why

Participant:	Because I would say those are the conditions that can pose a bigger risk to the condition to the situation. People might be worried of allergy, but allergy cannot implicate the TB study, but those two things HIV, Diabetes are the main issues.

Interviewer 2:	Ok

Participant:	But the other issue would also be understanding of the participants, you know Malawi what we call education illiteracy level is on the higher side for them to understand the mechanism you need to do more explanation. I think people who are better uneducated will be able to take it in than possibly those with low education.

Interviewer 2:	Just commenting on the level education, what do you think could be the best way to tell someone doesn’t work at the hospital but we want to approach them to join the study. What do you think could be the best way to explain to them to understand the concept of TB CHIM.

Participant:	The best way will be to tell the participants how the vaccine should work like the Toxfree vaccine and the like that they are given some vaccine and these are the reactions and that’s it immune response towards that infection so you would get, I would say the child is immunized and they will be able to fight any other infections so if there no, I would say if there is no other concerns like HIV and Diabetes they shouldn’t be worried.

Interviewer 2:	Thank you.

Participant:	And apart from that there is also that vaccine BCG vaccine in a way it will be like similar they have had TB thing implanted in them, but they showed some resilience they hear that.

Interviewer 2:	Anthony, you wanted to say something.

Interviewer 3:	Yes, apart from illnesses are there any other concerns that would surround the Tuberculosis challenge. I think am happy not happy am interested that you have mentioned about the BCG. So, we will be giving more or less the same BCG but, in this case, we will use the BCG not as a vaccine but as challenge agent. Just like the examples I give you earlier on about the other challenge models.

Participant:	Yes

Interviewer 3:	So, you have mentioned about reactions and the likes are there any other concerns surrounding a BCG or a Tuberculosis challenge outside the clinical setting. Anything that will be concerning if we are to bring a BCG challenge.

Participant:	I don’t think so, the only thing you need to do a lot of health education because sometimes the people, they have a negative outlook for the vaccine research and the like. So, publicity to demystify what you are doing have to be the main way because they would say they are putting in something, somebody can take the message somewhere distort it that one will be very important.

Interviewer 1:	Ok thank you, so now I would like to ask questions about exploring sex difference between among participants. How would you feel about gender in a CHIM participation like a male or a female participating in these types of studies.

Participant:	I don’t think, of course I would suspect you may have more female than males it’s possible because sometimes males are more critical and they look a lot on the this and like but females are easy to understand and to take in the new ideas. But that is my thinking I don’t know there might be a change if you go into tertiary institutions where likely you are going to find more males than females.

Interviewer 1:	Like in our previous work for example we observed that more males than females had joined our studies, what do you think could be the reason that.

Participant:	I would say is likely due to the illiteracy level I have said that in the tertiary section likely there will be more males than females but in the other sectors more females than males, that’s my suspicion.

Interviewer 2:	Do you think that the level of education also, she mentioned that in our previous study we saw a lot of men than female do you think the level of education also contributed to this, is what you mean.

Participant:	Yes, it’s likely. I don’t know where you did this previous study was it within the urban or rural.

Interviewer:	It was within Blantyre, Queen Elizabeth Hospital so we were inviting people through different adverts others were telling their friends, we had seen that actually more males participated.

Participant:	Because you said you invited through invitations that is already screening process those who could

Interviewer 3:	What we did initially was college sensitization the college students were coming, and we went on a TV program radio, then we saw that we started getting people from the community and most of them were males. The males kept on telling like their fellow males.

Participant:	Ok, fine

Interviewer 1:	Let’s now go to our next question for female participants who are of childbearing age for those who will be willing to participate in future studies to be required to practice adequate birth control measures during the study like the time that they are in that study. What are you views on this?

Interviewer 3:	The females will be let’s say if we are able to do BCG challenge females who are willing to join will be required to practice contraceptives methods, what are your views on that.

Participant:	It’s a good idea but I will go back to what I said because it will also be to illiteracy level, or I would say education level. My thinking I think with the data we mostly assured that even use of contraceptive is linked to education level here especially in Malawi. For those people who will be sure they will be able to control use thoroughly usually most ladies are the ones that are educated most of the times that can screen out those with a low education level. But it is possible and of course it may also mean they should be a good relationship between if they are married the husband wife, the husband should be able to understand that that my wife is doing this because she is participating in a study. But for those with low education it will be very difficult to convince the husband to say we are doing this	otherwise it can be misinterpreted.

Interviewer 3:	Do you think it will be a big challenge.

Participant:	I don’t think so, the only thing is you might need to catch more females deliberately to avoid an imbalance because that can also screen others especially the females.

Interviewer 1:	Ok moving on we are going to go to, like the study procedure. As part of the study participants will be injected with BCG CHM which you already mentioned as a challenge agent this is the same BCG vaccine given to children in Malawi as part of vaccination policy but this will be given at different doses to be able to collect it by taking part of the tissue from the injection site (biopsy) and also to understand how the body’s immunity responds to BCG how do you feel about injecting BCG.

Participant:	Mm, can you come again

Interviewer 1:	As part of the study participants will be injected with BCG as a challenge agent, this is the same BCG vaccine given to children in Malawi as part of vaccination policy , but this will be given at different doses to be able to collect it by taking part of the tissue from the injection site, as taking biopsy and also to understand how the body’s immunity responds to BCG.

Participant:	Ok

Interviewer 1:	How do you feel about injecting BCG?

Participant:	I think, injecting BCG most of the people will 3 welcome the idea because they know BCG, we inject the children it’s not something strange so that should be quite acceptable. And BCG is almost given to every child if you can tell the people every child receives BCG, I don’t think that will cause worries it will be accepted.

Interviewer 1:	Mmhi,

Participant:	But am not sure on the explanation of taking a sample that one might need more explanation

Interviewer 2:	Anthony maybe you can come in sample collection after BCG.

Interviewer 3:	Yes, its exactly like that we will give an injection, then after around within 48 hours we will call them back so that we are able to take biopsy and culture in the lab to see if we are growing back the BCG or not. So, your views regarding injection, what are your views regarding biopsy. What are your thoughts?

Participant:	Yes, biopsy might be a challenge it will need more explanation, demonstration for the people to be assured that it’s not a big chank that will be taken from them and there will not be complication following the biopsy that’s my thinking . Sorry and as well it means even the BCG you want to merge it in a safer place so that they shouldn’t touch unless what.

Interviewer 3;	What would be your suggestion for the injection site. where would you prefer?

Participant:	The shoulder would be easier for accessibility, the shoulder

Interviewer 3:	Coming back to the biopsy issue what do you think, what would you suggest is the best way to explain to a lay person about the biopsy for someone to understand maybe from your experience, we want to get a biopsy what is the best way to get that understand even in our local language is ok.

Participant:	Am trying to find a better way biopsy (mu Chichewa we would day kutsina, kutsinapo) in Chichewa we would say a pinch it’s not like you are going to take a chink of fresh no but actually if it can be done by injection biopsy, I think that will be I deal I don’t think people will be concerned.

Interviewer 3:	Like a micro bio

Participant:	Yes, but it will need more explanation for people to

Interviewer 3:	Are there any other words you would use apart from a pinch, because essentially, we will be taking body tissue.

Participant:	Yes, body tissue

Interviewer 3:	What you have said is we have to minimize as much as possible tissue collect.

Participant:	Yes

Interviewer 3:	What will be the words that we would use to say we will do this.

Participant:	Taking a skin pinch (Kutengapo ka m’nofu\)

Interviewer:	So, there are several ways of introducing BCG in the body one of them is by putting BCG in the lungs this method uses a thin tube that passes through the nose or mouth down the throat to the lung’s bronchoscopy. This method is also used to collect samples from the lungs what are your views on this.

Participant:	On that one I would say you may need to limit the number of people who can participate in that because it’s an invasive procedure and you need somebody who understands what is going on. And also people need to be explained on the risks and the control measures like what the previous research were doing like to say if at all there is anything a complication you are going bring you to this hospital and we are already prepared what . And you can keep the people somewhere for a period of time in anticipation of anything if there is no any reaction within that window period then you release them home, because that is more invasive than the biopsy one.

Interviewer 2:	How do you feel about the procedure?

Participant:	It’s a bit complicated there will be fewer people who can accept than biopsy one

Interviewer 2:	Do you think there are other ways we can make them feel comfortable, those who have accepted to join the study do you think there are ways we can make the participants feel comfortable about the procedure.

Participant:	No, not sure

Interviewer 2:	There isn’t.

Interviewer 1:	Ok, as part of the study, the study arranges a residential where those people participants who are participating in the study are kept for quite some time to observe them. What do you think about the arrangement of sending participants to a study residential site?

Participant:	It’s a welcome idea it’s better to more protective to the clients because if you send them home you are being irresponsible, of course those people who will be able to go to that side I think those who have got a better education secondary school upwards they should be able to take part. But I don’t think those with primary education it will be a challenge for them to take it up to understand what is going on.

Interviewer 2:	Where would you suggest we, where do you think would be a comfortable place to keep these people in the hospital or somewhere maybe near a hospital.

Participant:	Near a hospital

Interviewer 2:	What do you think we should be able to provide for the people to make their stay comfortable.

Participant:	Oh, comfortable food, gym and then go ahead at least they shouldn’t feel excluded from home they can be able to do other facilities while they are there

Interviewer 2:	How many days do think you will be comfortable.

Participant:	Keeping them

Interviewer 2:	Yes

Participant:	But not more than a week, 5 days if possible but not beyond that it might disturb some of the things that they are doing, and acceptability might be affected.

Interviewer 2:	Ok

Interviewer 1:	So, in terms of these people who are coming to join this study who can be listed as a proxy of a study participant. Participant listing a proxy for them.

Interviewer 2:	This is someone when you are doing a study, we usually encourage our participants to talk to someone it can be a relative a wife or someone you are close to. So, the question is who you think will be suitable to be a proxy who can talk to and provide the details.

Participant:	Either for those who are married husband or wife, but we also need medical personnel close to them to alleviate their worries to explain to them that will be ideal

Interviewer 2:	In that way you are suggesting that apart from the clinical study team the participant they should know someone who is working in the hospital.

Participant:	If possible, yes because sometimes people might not trust what you are saying because you have a particular gender if they have concerns, they say this one you can also talk to these ones in case of anything you feel you have not been assisted and there are concerns let him explain.

Interviewer 1:	So, now we want to ask you about what your views in terms of people can be participating like if someone a close relative of someone whom you know is keen or has joined the study how would you feel like the study, we are talking about like has joined a CHIM TB MARVELS study how would you feel.

Participant:	It’s a welcome idea because in that way they are helping though they are not medical personnel they are also helping the community because whatever findings will be coming from that research will be able to help the masses not only in Malawi, I believe I other countries as well.

Interviewer 2:	Would you encourage someone to join a CHIM study.

Participant:	Yes

Interviewer 2:	Would you join a CHIM study given a chance.

Participant:	Laughing, that one now would be difficult, I will look at my program when you say would you join am looking now at my logistics, I go to work how do I freeze the things that I do

Interviewer 1:	So, in terms of the idea is a welcome idea.

Participant:	Yes

Interviewer 1:	That it will help like the nation and other things.

Participant:	Yes

Interviewer 1:	What would make you feel like that.

Participant:	What will make me feel like I have contributed

Interviewer 1:	Like the idea is welcome, the one participating in the study will help like to the nation so, what will make you feel lie in those lines.

Participant:	I would say the results will be, the results will have a bigger impact on a bigger population, the other population that you would likely not include pregnant women, the children you are not going to include them, but the results will partially affect them

Interviewer 1:	What will you be your worries, will you be worried about anything in this case when someone is participating in a TB CHIM

Participant:	Not much unless if the screening has gone on well, you will rule out HIV, you have ruled out diabetes there should be fewer or no worries

Interviewer 2:	What will be the few worries you will have.

Participant:	Well for now I can’t think of any

Interviewer 1:	What things could be done to make a TB CHIM safe in health adult volunteers.

Participant:	The one I have said about the screening, the other thing is the explanation to make sure that participants understand and consent, the consent should be really sought and make sure they know not that they are participating because there is money or anything but they understand the implications and they are willingly going in the research , yes I think basically that’s that. Of course, you need to compensate of their time because some of them will have good family and they will freeze on that and do the research.

Interviewer 2:	Apart from what you have said do you think there are other ways we can make the participants feel safe, apart from what you have mentioned. Remember we are saying that this is a new concept.

Participant:	Yes,

Interviewer 2:	Though we have already done streptococcus this is TB now looking at how TB affect people, do you think there are other ways we can make sure that these people are not affected they are safe through out the study and even after the study.

Participant:	I would say they should have access to full medical facilities, and you can do some screening for other conditions within that period to assure them and even to assure yourselves that you don’t have any other problem. And you can even give grace period after the process you say you have left the possibly for one week and you follow them possibly two weeks just to make sure everything is fine before you release them to say no the effects of the study can no longer be detected.

Interviewer 2:	In case things happen, in case they have gotten sick what would you suggest these people should be taken care of

Participant:	Mm, one I would say possibly you might need to offer them access to some well-functioning health facilities like private hospital like us or Blantyre Adventist or if they are in Lilongwe either MASM so that they should feel that they are well taken care of and they are not exposed to dangers. And they should have access to information they should be updated on whatever is happening by the study physician.

Interviewer 2:	Ok

Interviewer 1:	Any other comments that you may have, or you want to share with us.

Participant:	On

Interviewer 1:	On the questions that have asked or anything that you.

Participant:	I would want to ask what your target or sample size is, what are you aiming at

Interviewer 3:	So, we will start slow so the protocol is still, we are still developing the protocol, but from what we have written so far, we will recruit small numbers maybe in tens, it will not be in hundreds,

Participant:	Yes, in fifties

Interviewer 3:	It will be in tens, so it’s mostly because some of the views that I was hearing from you aa maybe these procedures are a bit risky, go for small numbers. So, we are developing the protocol, but the protocol will be a well concentrated protocol after this exercise. This exercise will inform what exactly we include in the protocol.

Interviewer 1:	As for you have been working here as part of TB specialist this hospital.

Participant:	Coordinator yes

Interviewer 1:	Coordinating about TB issue, what is your date of birth year and month.

Participant;	6 July 1976, of course I look younger than

Interviewer 3:	From the comments I think you asked a question are there any other comments apart from the question, like any comment.

Participant:	No

Interviewer:	You want to talk about the discussion we had, the planned studies anything

Participant:	It will be a welcome idea to have feedback of the research findings it will be better, some research studies some findings are involving you should be updating as soon as tangible results so that the people who were also involved you will assure them that you were not used but you participated and this is the information that is coming out. Yes, I think that will be better.

Interviewer 3;	We will share the results of this exercise and also the results of the actual future protocol.

Participant:	Ok

Interviewer 3:	In what like how you would want to access this information.

Participant:	I know you contact some dissemination like conference or seminar that you were involved if you can send some participants to attend so that you can hear the findings and a call.

Interviewer 3:	So, you want to be called.

Participant:	Yes

Interviewer 3:	To say this is what we find., got the date, backdating to year and date I have to go back, 1976.

Interviewer 1:	We are towards the end of the interview that we have this afternoon, is there anything that you would like to share with us before we close the session.

Participant:	No, nothing

Interviewer 1:	On behalf of the team I would like to appreciate for your timea and the contributions towards the interview that we have had this afternoon, this will really help us much you have provided a lot of information that we were looking for, thank you very much.

Participant:	Thank you

Interviewer 2:	Thank you very much doctor.
