# Supplementary material for: Acceptability of advanced Controlled Human Infection Models: The case of a pneumococcal challenge in people living with HIV and mycobacterial challenge with Bacillus Calmette Guerin in Malawi
Source: PLOS Glob Public Health. 2026 Aug 18;6(8):e0006236. doi: 10.1371/journal.pgph.0006236 (PMC13485124; doi:10.1371/journal.pgph.0006236)
Supplement: Supplementary file 1 [file pgph.0006236.s001.zip › Documents/ACHIMA_ IDI_02_PLHIV_CG_24.02.24.docx]

Interviewer 1:	I welcome your doctor to this interview this morning, where we are conducting an in-depth interview accessing the acceptability of a pneumococcal challenge in people with HIV and Tuberculosis challenge in health adult volunteers in Malawi.

Participant:	Mhi

Interviewer:	So, the whole discussion will be centred mainly on this issue, feel free to express yourself in either in English or Chichewa both languages are acceptable

Participant:	Ok

Interviewer:	Welcome, have you ever heard or seen anything about controlled human infection.

Participant:	I may have heard about it but I don’t know anything beyond that

Interviewer:	You don’t know anything beyond that.

Interviewer 2:	Ok, when you say controlled human infection model, when a person gets sick the bacteria, his/ her body cannot fight to kill the bacteria or the virus	the body is weak. So, when we want to make new vaccines, all the vaccines were tested on healthy participants. But they could not be tested without them having the disease. Yes so, I think it’s unethical to on a sick person who is looking for help to get healed. So, in controlled human infection studies we take some of these health we give them the bacteria and then we test if the vaccine is working. The plan is not to get them sick, but we want to see if they can carry, sustain it if after giving them in a controlled setting we will be able to catch it back. So, since 2020 we have been giving people bacteria’s streptococcus pneumonia 6B, we give them in the nostrils after that we follow. It’s a controlled environment at the clinic. We make sure that we have ruled out everything possible which can put them at a risk to get sick, so in that case we got to set so after we have done all sorts of tests and follow ups, we invite them to give them the bacteria. Then we follow them up for three weeks to see if we can catch or see if the bacteria have grown in their nose, but they are not getting sick, so that’s what happens in controlled human infection study model. It’s not just streptococcus others can do on malaria, others real TB, other Tetanus anything you can do but it should be in a controlled environment where you will be able to follow that person, but participant should not get sick or reaching the point of dyeing. So successfully we have done this since 2020 no one got sick we tested PCV13 against 6B we wanted to see if PCV 13 can stop the growth of bacteria in a person. We saw that there wasn’t much difference in those who received placebo and those who got the vaccine. So having had that confidence that we can do this in Malawi and that was the first study of that type in Malawi and in Southern Africa that was the first one. So, we successfully done that and ok lets look into HIV positives so we are really looking at someone who is HIV positive and has been on drugs ART for three years has not has any admission in the past two years, has not had anu severe pneumonia he is healthy but is HIV positive, CD4 count more that 350, viral load detectable or less than 100, so we will recruit because we started we are half way those are the people we are recruiting. So, having said this we want also to do a TB CHIM it’s like we are moving from step to step in TB CHIM.

Participant:	Sorry, what’s a TB CHIM?

Interviewer 2:	CHIM is controlled human infection model abbreviation is CHIM, or you can say human infection model I think those are the names we usually use human infection model studies. So, because we are new in Africa, in Malawi it’s still a concept which is still being built this is like a foundation here. We said we should hear from people who have been taking care of people who are HIV positive, who have had TB, we want to hear from them do they think this is acceptable. What are their views we want to hear from them and them whatever you will say we will not publish as Dr (name) said this no anonymously	that ok this is what was said by so and so. We listen to those views transcribe them and then we write a paper and then accept this is not acceptable ok what ever you say we want to take that into consideration that we hear from different people. In TB CHIM what we want is you know BCG vaccine we receive when we are babies, we would test the same BCG vaccine we give a person and then collect samples to se who it goes, it’s not necessarily the active TB so that’s what we want to do. It’s like slowly we are moving from the safest population, so we want to test new things. Generally we want to hear from you from the point of view doing HIV CHIM giving HIV people streptococcus pneumonia 6B which they are already on a risk what are your, we want to do this because we want in future to develop vaccines which will be working well in these kind of people so that’s basically a CHIM study that is all about. So, maybe you might have read heard but what ever you are saying is what is with you, Charity will take you through the questions, what ever you know is what you can say it’s what is useful sure.

Interviewer 1:	After explaining that there is a small animation video which you will watch about how controlled human infection model are being done.

Video play:	It’s a given that vaccines prevent diseases and saves countless lives, but this takes ten years or more to develop new vaccines. We need to find a faster way, one way to speed up the process is by making better use of human infection studies, volunteers are given a sample of vaccine under development followed by a careful monitor infection medical care is always close to hand if volunteers fall ill and if they stay well, it means the vaccine works and can be made available much earlier. The idea is almost 300 years old but using todays advanced technology this type of study is not just scientifically sound but ethically responsible too and of course follows strict rules. Human infection studies show huge promise against fighting diseases like Typhoid and malaria but to develop vaccines most effective at the people at risk we need to test on those at most risk by expanding human infection studies and targeting them better the people most at risk could help develop vaccines of the future and save millions more lives.

Interviewer:	So, I think like it further explains to what Edna has explained. So, we can continue.

Participant:	Yes

Interviewer:	Thank you, Malawi Liverpool Wellcome Trust through MARVELS consortium has demonstrated feasibility of CHIM studies that’s conducting this type of model studies here in Blantyre. Now the plans are to extend to people at risk groups such as the PLHIV and TB in health adults. Having watched the video to what level would you agree or disagree to conduct a pneumococcal CHIM in PLHIV

Participant:	Sorry, level of

Interviewer:	Level of agreement, are you agreeing, disagreeing, undecided

Participant:	Ok, thank you for the question, I would have a few reservations as you lightly put it that these people who are living with HIV have different control of their disease. Some of them might be very immune suppressed and by definition in our setting that will be a CD4 count of less than 200 and at an increased risk of all these optimistic infections. So, the pneumococcal vaccine that was mentioned which may be given to these are patients who are already immune suppressed. They might not be able to mount the response to that they are already immune suppressed and the BCG for example that was mentioned is a live attenuated vaccine so am not sure a live attenuated with any live vaccine will worsen or how a patient with immune suppressed can fair after they are given that. But on the other hand, I think it’s a good idea because then you will know what else you need these patients in terms of prophylaxis that’s my opinion. I think it’s a good idea but my caution and worry.

Interviewer:	It’s like you are somehow undecided on whether this should be done or not

Participant:	It should be done, with care

Interviewer:	So, like the level of agreeing, at which level are you like agreeing to do this

Participant:	If I put it on a scale of percentages, I would say 70% yes, but then with care

Interviewer:	Alright, thank you, in your views what are the risk that can be there in conducting a pneumococcal CHIM in people living with HIV. What can be the risks that you feel they can be there?

Participant:	Ok, so as I mentioned earlier that the risk would be maybe higher for patients that are living with HIVand are immune suppressed, different levels of immune suppression CD4 count cells, yes so I would say the risk will be this person is not able to mount response on their own already and now we are giving them how can I put it , we are giving them an insult in quates so you are not sure weather, or how they are going to respond to that. So, I would also be maybe accessing different groups within the HIV group to say how does it compare in those patients that are already immune suppressed to those that have HIV but they have good control so how does it be, HIV might not also be the only reason that is causing immune suppression in these patients there will be for instance auto immune disease on board and other comorbidities that might contribute to low level of immunity in these patients.

Interviewer 2:	All those things that you mentioned the CD4 count	is more than 350.

Participant:	Yes

Interviewer 2:	They have been on ART for more than 3 years, they haven’t been admitted for the past two years, is HIV positive but is health, what will be the risk?

Participant:	Someone who is healthy

Interviewer 2:	But is HIV positive adhering to ART, what will be the risk giving them a 6 B?

Participant:	A what

Interviewer 2:	A streptococcus pneumonias

Participant:	Aa 6 B

Interviewer 2:	What will be the risk having put all the other measures in control.

Participant:	Well unless it is something, it’s a condition that has been missed not diagnosed that can further put them at risk of immune suppression then I will be worried bit if everything is under control and you feel you have screened for every disease and they are ok, doing well at this point I cant think of any risk at this moment in time but if I do then I will get back to you.

Interviewer 1:	Thank you, in your views what are the other concerns that could arise conducting a pneumococcal CHIM in people living with HIV.

Participant:	From my point of view as a clinician

Interviewer 1:	Yes

Participant:	I think it depends on their acceptability of this TB CHIM pathway in these patients so am assuming yes you are going to get consent from the patient maybe from the guardian. But I think key will be if they understand what you will do with or on their patient without which I don’t think it will be acceptable because people will say they want to make me a little sicker than am already am and the result or the outcome is not going to be a good outcome. So, I think the acceptability will be a big issue that has to be addressed promptly and prior to the intervention.

Interviewer 2:	What if the TB CHIM will be done in healthy participants who are not admitted.

Participant:	In the outpatient department?

Interviewer 2:	Not as an outpatient but those people who are home, they are not sick, they are not at the hospital to seek any medical attention, they are in school they are in college, they are not sick in any way what will be your concerns. Someone who is healthy you and me we are at work we don’t have any medical issue what your concerns will be.

Participant:	I would still think that I think if a patient aperson is given a diagnosis they might be well they might not have any physical signs of being unhealth or un well but I think at the back of their mind they still think ok I have HIV I have to be careful of certain things that I can do or everything because well am at risk of getting anything even with a very good CD4 count . So, I think the participants might think I would rather be as I am right now nothing introduced in my body let me get my medication and I will be fine because this is what the hospital told me that you can live a healthy normal life for ten or 30 years with HIV. So, I think ok am not doing this because am not good as I am nothing coming in or nothing coming out.

Interviewer 1:	What things could be done to make a pneumococcal CHIM in people living with HIV safer from your point of view as a clinician.

Participant:	Safer

Interviewer 1:	Yes

Participant:	I think you are doing it all, almost of it as you explained at the start of the interview that you want to have a health adult in quates, and a health adult is not sick they haven’t been admitted CD4 count ok so that will be the major, the main thing that you need to address and especially maybe based on the family history if these people have any underlining conditions that a blood test or a scan can pick from that patient at that material time.

Interviewer 2:	Yes, I think apart from that do you have anything that we can do as a clinical team, as a study team what do you think are other things that we should put in place. You are saying that they are health they are not sick now, but it should be safe. What are the measures that we can put in place to ensure that they should feel safe?

Participant:	Mm, ok I would think that for conditions for instance am not sure if you would recruit a person living with HIV they are health but they also have Asthma ok, Asthma will come in in a chronic fashion and then they have episodes where they have exasperations where they get worse when they have an attack in quates so I think some of the environmental factors it’s very difficult to control. From when they come to the hospital fir interviews and what not on their way back there is these smells will trigger that give them an asthma attack. I think that would stand in the way a am just giving an example of all this Asthma there t are some environmental factors that it’s difficult for your team to control but the ones that you can control is the counts objectively like CD4 , how well is this person we know that the higher the level of immune suppression , the higher the CD4 count then the patient is at reduced risk of getting these optimistic infections. What else that’s for the environmental control that you can’t control on the patient side. From your clinical team side, I would think that I don’t know how you will do it but the participants need to have like more that 90 or 95% of understanding of what you really want to do and then you can seek consent and they can grant the consent.

Interviewer 2:	Doc, I want us to address them not as patients but as participants.

Participant:	Participants

Interviewer 2:	You should take away patient, a patient is sick lets address them as participants, we should look at the point that they are health, they are not even in hospital

Participant:	Yes

Interviewer 2:	We can continue.

Interviewer:	Apart from what you have explained, you have explained about other environmental health factors which cannot be controlled that can trigger problem to people like ones with Asthma. So, in this case what can you say about considering this group of participants, what can be done like to eliminate or to reduce.

Participant:	I think my opinion will be look at it from the perspective that there is things or factors that can be modified and those that cannot be modified. For example the environmental trigger that I talked about will be you can’t modify that you can maybe asking them wear a mask all the time avoid triggers and everything but some of them you cant. Or for instance lets take a person living with HIV who would be a participant in the study they have HIV, never have TB they are ok but they are living in the same house maybe with a wife or husband or a parent they have new positive Tuberculosis diagnosis that also is a it could be modified they can be in another room, split houses but I think that will be too much an ask

Interviewer 2:	In a Malawian set up.

Participant:	For the participant that is also one thing that will be a non-modifiable risk factor especially in our setting, but it could be also modified in other settings if we had the resources to separate or take this potential participant out of this risk at their home.

Interviewer 2:	Ok, you have reminded me of something, so when we have recruited these participants, we have given then the 6 B strepto we take them to Grace Bandawe for 3 days,

Participant:	Oh

Interviewer 2:	The first three days to ensure that maybe they are safe in case they are sick because they are at the risk that they can get sick those three days. What would you say about that, taking them to Grace Bandawe what will you say about that?

Participant:	I think the isolation is a good idea, I don’t know if it works for everybody because you said that these might be the college student or people that are working so, I don’t know how that gets to affect them if they are taking three days away. I assume they are residents of Blantyre.

Interviewer 2:	Yes, it’s strictly one hour using a minibus it should be a radius of one hour.

Participant:	I think the isolation in terms of wanting to observe them in case they develop an infection and they become sick is a good idea my only other concern will be, what’s the isolation place like, in view of the factor as I said these people might have all sorts of comorbidities but be held from an HIV point of view so what is happening in that kind of setting what kind of food are they taking, what are they drinking what’s the rooms like, but I believe like they are made well just to make it secure or a good isolation place. Am sure you looked into all that.

Interviewer 2:	So how many days would you recommend that someone be isolated or taken care of after giving the 6 B, what would you recommend how many days.

Participant:	You have mentioned that

Interviewer 2:	Three days

Participant:	Yes, 72-hour period but also if I have the knowledge of what this 6B really does, I know it’s a vaccine

Interviewer 2:	It’s a bacteria, it’s a live bacteria

Participant:	It’s a live bacteria.

Interviewer 2:	Yes, we inoculate them in the nose, so it’s developed from 6 B.

Participant:	Yes

Interviewer 2:	The one you know. that causes all sorts of infections that the real bacteria that we get.

Participant:	Am told that maybe 72-hour period of three days is enough that maybe they have responded to the back that was given. But I would want to know maybe I will do some reading that yes maybe within 72 hours maybe they would show but does it have maybe delayed on set or something. Delayed on set of action or a delayed reaction from the participant that has received it then would that warrant extending the time 3 days to 5 0r 7 days so that’s why am saying I really can’t comment because I don’t know the onset of action and reaction once this is given to participants

Interviewer 2:	Thank you.

Interviewer 1:	I think the next questions that I was supposed to ask they have been answered, this set on conversation that we have been having. So, we will be moving forward into the next set of questions. I would like to capture information about your self this is the part where I will ask you your personal information about, date of birth, year, and month.

Participant:	Ok,

Interviewer 1:	At this point, when were you born date, month, and year.

Participant:	11, May 1992

Interviewer:	1992

Participant:	Yes

Interviewer 1:	We are towards the end of the interview I would like to ask if you have any other information that you suggest to us, to discuss or comment on questions.

Participant:	I think most of the questions if not all have been answered and addressed through the course of the interview. My last comment I think it may be a repeat but I think, I would just say it’s a good initiative and it’s important for the participant to understand that I think if this intervention will be done and they participate it will help them in a way directly or indirectly because anyway we would establish patterns that would improve participant care or other people that are helped just like the participants are . Yes so, its’s important that they understand what you are trying to do because it has both direct and indirect results or effects on that study population. But I think it’d a good initiative and yes, one more thing it might be ok and everything CD4counts above 350 but remember that Tuberculosis or TB can occur at any CD4 count so I think that ought also to be given extra attention the TB screening in these health participants yes. And also establish weather they are receiving ant form of prophylaxis for instance against the Tuberculosis so you screen they have good HIV control but you screen for TB it doesn’t look like they have TB but maybe at home there is someone with smear positive and the like so that they can give them prophylaxis Isoniazid and everything so that can also help your study thorough TB screening put them on prophylaxis if you think they have risk and then you cab go ahead with the rest.

Interviewer 2:	Apart from the prophylaxis I remember there is a question if they are on prophylaxis what would you suggest is the best way to screen them maybe apart from getting the subject you know people lie, what can we do to do better on screening and making sure that they are not at risk of TB

Participant:	Once again, I will be biased towards me in patients like we do a thorough TB screening, Tb would either be pulmonary or extra pulmonary. Pulmonary we are going to look at chest X-Ray we are going to ask them to send sputum do a Gene Expert do a culture one and for the extra pulmonary we can see a fusion on chest x-ray you can go on and analyze that but remember this is a healthy participant, so we do not assume that they have a fusion. If they had a fusion maybe they can have a symptom no symptom, so you can do other scan you can do a chest x-ray, I recon on the tideline skin test is not usually sensitive in these patients that are living HIV and they have received the BCG vaccine but that might be another thing to look into and consider, so objectively you can do blood test , you can di imaging the sputum yes.

Interviewer 2:	Do you have a separate interview for TB.

Interviewer 1:	Yes, for TB it’s a separate and this one for HIV is a separate one only that the other questions are of TB.

Interviewer 1:	Ok

Participant:	Here I think it’s a good study to find out what it will yield

Interviewer 2:	e will share you the findings. Are we remaining with other sets of questions?

Interviewer 1:	No, we have concluded if there is nothing more, I think this marks the end of the interview that we had today this morning.

Participant:	Alright

Interviewer 1:	Thank you.

Interviewer 2:	Thank you very much for your time, we really value your input we will analyses transcribe that, you know Anthony is the PI in case we forget please remind me that we should share the paper when its published

Participant:	Is this Anthony

Interviewer 2:	Chirwa
