# Supplementary material for: Acceptability of advanced Controlled Human Infection Models: The case of a pneumococcal challenge in people living with HIV and mycobacterial challenge with Bacillus Calmette Guerin in Malawi
Source: PLOS Glob Public Health. 2026 Aug 18;6(8):e0006236. doi: 10.1371/journal.pgph.0006236 (PMC13485124; doi:10.1371/journal.pgph.0006236)
Supplement: Supplementary file 1 [file pgph.0006236.s001.zip › Documents/ACHIMA_ IDI_03_PLHIV_CG_21.02.24.docx]

Interviewer:	Welcome to this interview this morning where we are having a chart on accessing the acceptability of experimental pneumococcal challenge in people living HIV and Tuberculosis challenge here in Blantyre in health adults. The interview, this interview is 35 -45 minutes so I hope you will feel comfortable to express yourself in Chichewa or English feel free to express yourself in either language that you are comfortable with. The first question that I would like to ask is about knowledge attitude and perceptions about what you know. Have you previously heard, read or seen anything about controlled human infection model studies.

Participant:	I have heard about it, but I don’t know in detail

Interviewer:	You have heard

Participant:	Yes

Interviewer:	You heard this from where?

Participant:	So, we were attending research meetings, research dissemination meetings

Interviewer:	Ok, that was during research dissemination meetings. This was here at Queens.

Participant:	Virtue international virtue meetings

Participant:	Alright, thank you there is a small animation explaining about what controlled human infection model are

Video play:	It’s a given that vaccines prevent diseases and saves countless lives, but this takes ten years or more to develop new vaccines. We need to find a faster way, one way to speed up the process is by making better use of human infection studies, volunteers are given a sample of vaccine under development followed by a careful monitor infection medical care is always close to hand if volunteers fall ill and if they stay well, it means the vaccine works and can be made available much earlier. The idea is almost 300 years old but using todays advanced technology this type of study is not just scientifically sound but ethically responsible too and of course follows strict rules. Human infection studies show huge promise against fighting diseases like Typhoid and malaria but to develop vaccines most effective at the people at risk we need to test on those at most risk by expanding human infection studies and targeting them better the people most at risk could help develop vaccines better and could save millions more millions lives.

Interviewer:	This was just an explanation about what human infection model studies are, so we can continue

Participant:	Yes

Interviewer:	Having watched the video would you agree or disagree to conduct a pneumococcal CHIM in people living with HIV

Participants:	Yes, I would agree

Interviewer:	You agree, in your view what could be the benefit of conducting a pneumococcal CHIM in people living with HIV

Participant:	As it was shown in the video that it takes edges for them to find an effective vaccine so to shorten the process then we can conduct this type of study where we give the infection or the challenge to volunteers to quicken the process of doing the vaccine.

Interviewer:	In your view what are the risks of conducting a pneumococcal CHIM in people living with HIV just looking at it

Participant:	Yes, there are risks potential risks obviously HIV are immune compromised so maybe they may have complications developing after getting the challenge and am not sure it will be acceptable knowing how Malawians behave to things like vaccines and other things with experience from Covid. So, it will need a lot of explanation to convince them to participate.

Interviewer:	On the risks that are there if you are to group them to individuals who are like the clients to the health sector and other aspects how can you group the risks that can be there

Participant:	So, to the participants obviously they get sick, to the health care system when they get sick, they go to the health care system it means more work and use of resources. And they you said to the community.

Interviewer:	Yes

Participant:	To the community I think the community develops when people are well and are contributing to development. So, if people are sick the community will suffer because these people are sick, and they cannot contribute to development.

Interviewer:	It’s like health people are the ones that can work in development activities so in this case there will be issues to, it will affect development

Participant:	Yes, and if the elder being admitted the family will need more resources to look after them when they are very sick

Interviewer:	Thank you, what other concerns can arise in conducting pneumococcal CHIM in people living with HIV apart from the one that you have mentioned like burden to the health care workers, workload and need for more resources and from the community it will slow development. What other concerns will be there or that you feel might be there.

Participant:	Maybe that can contribute to development of new resistant organisms

Interviewer:	Development of new resistant organisms

Participant:	Yes, that’s a potential possibility

Interviewer:	Another concern that might be there

Participant:	Those are the main ones that I can think about

Interviewer:	Mhi, alright what could be done to make pneumococcal CHIM in people living with HIV safer. To start with safety for them, where they are coming from and consider everything like the hospital set up where they will.

Participant:	Yes, so first of all make sure that whatever you are challenging yourself with is safe and also make sure that selection of participants it’s very strict for example maybe only involve those that have achieved a good biological control of the HIV. And then also as you said the environment where they will be monitored make sure everything is there in case something emergency happens, they need to be cared for properly.

Interviewer:	In terms of the care that can be provided what can you suggest can be the basic standard care that can be readily available to them.

Participant:	Mm, I will answer this question based on a study that I don’t know it was not this type, but they were just trying out a malaria drug. Patients were admitted very well normal patients were admitted in a ward and the vital signs were monitored regularly up for new symptoms yes and then they were supposed to have emergency things in case something happens, the staff members have to be properly trained to manage the potential emergencies an be able to give proper and adequate first aid treatment in case patient develops a complication and be there 24 hours.

Interviewer:	So, like on a similar study conducted by MLW that is still running what they do is they have a residential area stay in space where participants

Participant:	Its community based

Interviewer:	It’s not community based for now the study that is there they use Grace Bandawe premises

Participants:	Ok, so I understand am differentiating from being at a hospital ok. It’s at Grace Bandawe

Interviewer:	So, in this case I would like to get your views on this approach of keeping the clients like the first few days that they have joined the study and they have been introduced to the challenge. How do look at the approach of providing a stay in space for clients

Participants:	So, if the stay in place like that that’s fine provided, they have proper equipment to take care of patients in case they develop something serious.

Interviewer:	In this case Grace Bandawe was selected because it’s close to a hospital like Mwaiwathu there they have study staff members who are available present right there if there is anything they can take participants to the hospital. Looking at that for how many days do you feel the study can keep participants who have joined the study at the stay in premises.

Participant:	Laughs

Interviewer:	On the number of days

Participant:	On the duration I have no idea, but I think keeping them for more than a week may affect most Malawians socio economic status because they live on hand to mouth.

Interviewer:	What could be the reasonable number of days to keep them that you feel it cannot affect them

Participant:	Three days

Interviewer:	What things can be done to ensure positive participants experience in CHIM model studies	.

Participant:	I think the key thing is education and awareness. There is a need for a lot of civic education and to make sure the public is aware of things that is happening. It’s very easy if something that is happening people have lots of myths and they think you are hiding you are using the study hiding behind other things issues to do with fertility they add everything to fertility saying you want to control how many children they want to have in future.

Interviewer:	To avoid misconceptions in communities because everything they connect it about the future.

Participant:	Yes

Interviewer:	Any other comments that you may want to add on to this about how best we

Participant:	Yes, and if there is any

Participant:	Yes, and if there is collection of blood, I think that also needs to be clear and properly explained because experience from previous studies volunteers may not be comfortable with a lot of blood samples taken on daily basis.

Interviewer:	Blood samples that will be involved

Participant:	They may not complain about taking a nasal swab whatever but as long as blood is concerned, is involved you need a lot of awareness, education and explanation.

Interviewer:	There is need for more awareness and education

Participant:	And also minimize blood sample collection

Interviewer:	For now I think if you have any other information anything that you want to add up on what we have discussed about CHIM model studies regarding the questions that I have asked before. If there is anything that you want to add

Participant:	No

Interviewer:	So, for now we are at the stage where I will need to get personal information about the date, month and year of birth. When were you born?

Participant:	2 March 1977

Interviewer:	You have been working as a senior doctor for how many years

Participant:	Since 2009, so that should be 14 years

Interviewer:	This is the only hospital that you have been working in

Participant:	Yes

Interviewer:	More like working here at a tertiary hospital what has been your experience like

Participant:	In terms of

Interviewer:	The work that you do and the type of patients whom you see

Participant:	At the beginning the majority of patients were people living with HIV over 70% but now the numbers have changed abit now we are seeing a lot of patients with non -communicable diseases, stroke, some lung disease and people are living longer now we are seeing a lot of older patients coming to the hospital. In terms of infections specifically pneumococcal infection that you have mentioned the numbers are coming up when I compare ten fifteen years ago patients coming in with what is invasive pneumococcal disease. But now very few people coming in.

Interviewer:	On this study like the one that we are discussing about the controlled human infection model, who would you encourage between males and females to join to take part in this study

Participant:	Laughs

Interviewer:	The people whom you have been seeing which group you can recommend

Participant:	Men are easier even though they are the ones who go out to make money and men are easier

Interviewer:	Why are you saying that men are easier

Participant:	Yes, because most women will have kids at home, they have kids to look after at home, so it will be challenging for them to leave their family for three days rather than men its easier for men to leave home for three days.

Interviewer:	So, you will opt, suggest men will be much easier in terms of responsibility because women have more responsibilities at home and other roles like the motherly role.

Participant:	Yes, unless if you are looking at for older women maybe older women who do not have kids at home

Interviewer:	We are at the end of the interview that we has this morning, anything that you would like to comment on discussion that we had this morning feel free.

Participant:	No

Interviewer:	Thank you, it has been a pleasure to have you this morning and I really appreciate for your time and your contributions that you have made towards this data collection, thank you.

Participant:	Thank you
