# Supplementary material for: Acceptability of advanced Controlled Human Infection Models: The case of a pneumococcal challenge in people living with HIV and mycobacterial challenge with Bacillus Calmette Guerin in Malawi
Source: PLOS Glob Public Health. 2026 Aug 18;6(8):e0006236. doi: 10.1371/journal.pgph.0006236 (PMC13485124; doi:10.1371/journal.pgph.0006236)
Supplement: Supplementary file 1 [file pgph.0006236.s001.zip › Documents/ACHIMA_ IDI_04_PLHIV_CG_23.02.24.docx]

Interviewer:	Welcome today as we are to start this interview. Welcome doctor this morning to this in-depth interview that we are about to start .As I have explained in the consenting process at Malawi Liverpool Wellcome there is the MARVELS group that is conducting these the Controlled Humna Infection Model studies and as part of that study we are interviewing senior doctors, clinicians and nurses in terms at how they look at extending Controlled Human infection Model to the group of people living with HIV and the other group to test TB challenge in health adults volunteers in Malawi. So, A part of the interview there are a set of questions some need explanation that is the reason why the interview will be audio recorded and the other component will be entered directly tablet based.

Have you previously heard or read anything about controlled human infection mode?

Participant:	Yes, I think I have, yes.

Interviewer:	What was the source of information.

Participant:	I think it was from the internet

Interviewer:	Ok, this was a science article or

Participant:	Yes, this was a science article I think it was from UK.

Interviewer:	So, to understand more about this there is a small animation that you are going to watch

Video play:	It’s a given that vaccines prevent diseases and saves countless lives, but this takes ten years or more to develop new vaccines. We need to find a faster way, one way to speed up the process is by making better use of human infection studies, volunteers are given a sample of vaccine under development followed by a careful monitor infection medical care is always close to hand if volunteers fall ill and if they stay well, it means the vaccine works and can be made available much earlier. The idea is almost 300 years old but using todays advanced technology this type of study is not just scientifically sound but ethically responsible too and of course follows strict rules. Human infection studies show huge promise against fighting diseases like Typhoid and malaria but to develop vaccines most effective at the people at risk we need to test on those at most risk by expanding human infection studies and targeting them better the people most at risk could help develop vaccines better and could save millions more millions live.

Participant:	Ok

Interviewer:	So, after watching this we can proceed?

Participant:	Yes, we can proceed

Interviewer:	Thank you, having watched the video to what level would you agree or disagree to conduct a pneumococcal CHIM in People Living With HIV, you agree, strongly agree, neutral, disagree, strongly disagree

Participant:	I agree not strongly agree but just agree.

Interviewer:	In your view what could be the benefits of conducting a pneumococcal CHIM in people living with HIV?

Participant:	I guess in my view it will help to fasten the process of vaccine development for these people and probably understanding the dynamics of the disease in these people. I think all in all it will be a good idea to fasten that because we have a large population in Malawi that are HIV relative that we see and come with Pneumococcal diseases so, if we can find a faster way of tackling that. And if this way where we have the model challenging humans that are not infected that are well, but they have HIV if that model helps to speed up the process it’s a stepping in the right direction.

Interviewer:	Ok, I think you are in support of the idea that it will help.

Participant:	Yes, it will help

Interviewer:	In your view what are the risks that could be there in conducting a Pneumococcal CHIM in people living with HIV.

Participant:	I mean the risk is that we know that people that are HIV reactive tend to suffer from more Pneumococcal infections so there is a higher risk than that, we know it’s a controlled environment, but the disease can become more severe in those patients. In some it can come to significant mobility, some may die from that so, those may be the risks that we need to look into

Interviewer:	So, as part of looking into those two areas that you have mentioned about develop like other active disease and leading to death, are there any other risks that might be there that you can think of.

Participant:	No, at the moment no I cannot think of any other risk.

Interviewer:	Ok, in your views what other concerns could arise in conducting a Pneumococcal CHIM in people living with HIV.

Participants:	I would say probably I would say for me the major concern probably will be the ethical approval for that. But from the patient/participant perspective the concern would be how they would understand or accept. How would they understand the fact that they have been given an infection to see how it develops. I would think probably majority of Malawians might not fully understand the science or the reason behind that, but I think with careful explanation that can be easily overcome. The other concern I find with this, probably the kind of compensation that is given to these patients, I know even in UK it was actually significant and probably I would think that might challenge Malawi where probably a lot of people would want to join that for the sake of the money or for the sake of the compensation that comes with it, yes I think other than that I cannot think of other concerns that I might have due to this.

Interviewer:	So, on the issue to do with compensation you have mentioned something like people might be interested like to join because of the compensations that is attached to it. How best can this be further reduced like to make sure that the people who are joining are really joining not because of the compensation but because they have understood the concept and the procedure that they are going to be involved in.

Participant:	Probably I don’t think I have a direct way of how to overcome that but probably I would say when getting the consent that should not be the first thing telling the clients or possible participants in this you get such amount of money probably that might be mentioned at the end after they have understood everything. And I would want to, I know there are already guidelines on how much you can actually give, so am not trying to invent the wheel but probably just giving enough money in order not to look like you are coercing the participant because of the amount of money that you are giving. Unfortunately the amount is subjective its different from people to people it’s difficult to strike a balance on amount of money, someone foe example from Namiwawa would think is enough, what about someone from Mbayani not to say much about people from Mbayani how much someone from Mbayani or different social statuses, how much would a CEO of a company and someone who is not a CEO how much would they think is enough for that. But I believe there are already guidelines of how much would be enough and how much would be more than enough.

Interviewer:	What things can be done to make a pneumococcal CHIM Iin people living with HIV safer.

Participant:	So, I would think of closer monitoring of those people that have been asked to participate and probably even though they are HIV reactive there those people that are not severely immune suppressed who can easily get an active infection, the infection becoming very active and probably difficult to control and then, I would think this will be probably done in a hospital setting where the medical team are close by if there will be any complications people can be around to easily deal with that .

Interviewer:	What things can be done to ensure positive participants experience in controlled human infection model studies.

Participant:	Can you repeat the question again

Interviewer:	Ok, what things can be done to ensure positive participation and experience in controlled human infection study model

Participant:	I think the first part is probably the most important thing is for the people to understand what the risk are and what are the benefits, right from the outset when they are joining, they have made the decision based on the benefit and they understand what the possible risks are for this. And I would probably be coming back to for I think having a team ready that will deal with any other complication that can develop and being readily available for the participants, probably that I would say for now.

Interviewer:	In terms of having the clinical team readily available for participants, in this case this should be in a hospital set up or they should be like in residential stay where like this is done.

Participant:	So, I would say thinking through it it’s difficult to have this all in a hospital set up probably looking at the numbers it might be difficult to have all the people. But probably even if it’s at home if we have the medical team that is available 24 hours for them, I think it can be safely done in a home environment. But probably I would prefer if it was done in a hospital environment, but it can still be done in a home environment.

Interviewer:	In terms of having, it done in a home environment how best could you explain how this can be like implemented.

Participant:	If for example you have introduced the bacteria, you have infected the people with it, I think the most important thing is explaining to them what the bacteria can do. For example, if they develop a cough, if they develop any adverse effects or anything unusual, they need to notify a clinician who will be available on a phone for them. And then having set up a place if they feel unwell can be easily assessed and probably that place could be open 24 hours in a day. And making sure that from where the patients are there should be easy access in terms of transportation so that they can reach the hospital, or they can reach a place where they could be easily accessed without any challenges in terms of transportation. But probably the most important thing is for them to understand the symptoms of the disease, anything unusual that they feel after that they should be able to notify the team.

Interviewer:	Ok

Participant:	Probably for the participants I would encourage that if there is provision of things like temperatures or thermometers so they should be able to measure their own, in case they are becoming unwell they should be able to notify the people responsible

Interviewer:	Ok, provision of thermometers to participants

Participant:	Yes

Interviewer:	Ok, any other comments that you would like to make on what we have discussed so far.

Participant:	Probably I would just want to say again that probably with a good explanation this could be acceptable in our setting as long as the clients understand what is tried to be achieved at the end of this. I think it’s feasible it could easily get acceptable in our setting, and for sure it’s a good thing.

Interviewer:	Ok, apart from that anything else that you would like to comment on

Participant:	Not really, I don’t have any comments

Interviewer:	Ok, in terms of when participants have been introduced with the bacteria they are sent to a residential stay, the study arranges to keep them for the first three days

Participant:	Ok

Interviewer:	How long you feel in terms of number of days when participants can be kept at the residential site of the study

Participant:	I think to be safe for me I would say probably five days, not less than five days, I wouldn’t keep them for less than five days

Interviewer:	So, five days will be like enough to keep them at the residential place

Participant:	Yes.

Interviewer:	What other things would you advise that have to be looked into at the residential stay where these participants are to be kept for the five days.

Participant:	Aa, probably I will need help with that what do you mean by that.

Interviewer:	In terms of any other things regarding the condition of these people they are in they have been introduced to a bacteria for the medical aspects what are the things that should be readily available at that place in case something happens to the participants. What are the first things that should be looked into as they are keeping these participants at the residential stay.

Participant:	Probably I would think the most important thing would be the monitoring, I mean they need to be monitored closely for the time that are kept in this residential place. Am not really sure how it is usually done probably I would think you might need to have bit of an isolation place as well in case someone becomes unwell weather you don’t want them to mix up if they are coughing for example you don’t want to mix them with other people that are well during that period of time, other than that I don’t think I have any other issues.

Interviewer:	So, for this part we are at the part that I want to gather information regarding the type of patients whom you take care of we are done. The questions that am about to ask I would like to get your personal information like date of birth, year, and month.

Participant:	27 June 1989

Interviewer:	Ok, any other comments that you would like to make

Participant:	Not really, I don’t have any other comments not at this point in time

Interviewer:	If there are no any other comments I think this marks the end of the interview that we were supposed to have this morning

Participant:	Aa, you said it was 45 minutes

Interviewer:	I think it can be 45 minutes or less than that

Participant:	Ok, alright thank you

Interviewer:	Thank you so much it has been a pleasure to have you for the short interview

Participant:	Thank you

Interviewer:	Thank you for the contributions that you have made will help us a lot as we are moving in the next level of data collection in this study, Thank you

Participant:	No, you are welcome.
