# Supplementary material for: Acceptability of advanced Controlled Human Infection Models: The case of a pneumococcal challenge in people living with HIV and mycobacterial challenge with Bacillus Calmette Guerin in Malawi
Source: PLOS Glob Public Health. 2026 Aug 18;6(8):e0006236. doi: 10.1371/journal.pgph.0006236 (PMC13485124; doi:10.1371/journal.pgph.0006236)
Supplement: Supplementary file 1 [file pgph.0006236.s001.zip › Documents/ACHIMA_ IDI_06_TB SPE_CG_28.02.24.docx]

Interviewer:	Ok, welcome to this interview this morning, as I have explained MLW intends to conduct a study that is aiming at accessing the acceptability of Pneumococcal challenge in people living with HIV and in health adult volunteers in Malawi.

Have you previously heard seen or read anything about controlled human infection model study?

Participant:	Yes, I have

Interviewer:	Ok, what was the source of information

Participant:	It was through research ethics research committee that I sit on

Interviewer:	Ethics research committee, this was a meeting.

Participant:	Yes.

Interviewer:	To understand more I will give you this video to watch it shows more about how

Video play:	It’s a given that vaccines prevent diseases and saves countless lives, but this takes ten years or more to develop new vaccines. We need to find a faster way, one way to speed up the process is by making better use of human infection studies, volunteers are given a sample of vaccine under development followed by a careful monitor infection medical care is always close to hand if volunteers fall ill and if they stay well, it means the vaccine works and can be made available much earlier. The idea is almost 300 years old but using todays advanced technology this type of study is not just scientifically sound but ethically responsible too and of course follows strict rules. Human infection studies show huge promise fighting against diseases like Typhoid and malaria but to develop vaccines most effective at the people at risk we need to test on those at most risk by expanding human infection studies and targeting them better the people most at risk could help develop vaccines better and could save millions more millions lives.

Interviewer:	Thank you, having watched this video to would you agree or disagree to conduct a TB challenge in healthy adult volunteers

Participant:	I would agree

Interviewer:	You, would agree, in your view what could be the benefit of conducting a TB challenge in healthy adult volunteers

Participant:	I think we still don’t understand much about how to prevent TB if we can have adult volunteers in case that can help to develop a vaccine in future for TB again that will be better off .

Interviewer:	What are the risks that can be there in conducting a TB CHIM in healthy adult volunteers

Participant:	I think the risk could be that we don’t know how that will behave in that out, maybe some can get sick from it and unfortunately for some other reasons on how their body react if that illness can lead in death, then it can become a scare to people in the community.

Interviewer:	Ok, other than that these are the two risks that you feel might be there

Participant:	Yes

Interviewer:	So, what are the concerns that could be there in conducting a TB challenge in healthy adult volunteers

Participant:	The concerns?

Interviewer:	Yes

Participant:	I think the concern is, could be like from what I see at the community from experience like Covid could be like there might be conspiracy theories against it to say maybe people can come up with certain rumors that they are putting something in you, something like that and that can scare people. So, that is a risk, and it can probably make if that goes it can make people to get scared of any other new vaccines that being tried on.

Interviewer:	Ok, in the previous study that MLW conducted where people were introduced like a bacteria in their body it was noted that, that study enrolled more males than females, why do you think could be the reason for having more males to participate

Participant:	I think by nature men are kind of loose status and are also women I think they are thinking about their children and taking care of their family and so they want to be certain about what they are going into. And if they are going into something that they are not sure or they don’t have much information about they don’t want to risk it, especially with the background of rumors	if you take this vaccine things will happen to you. I think more women are worried about their fertility, their children how to look after them if they fall sick from this or if they die. But I think we men they think they can take up the challenge.

Interviewer:	Ok, how would you feel about gender in conducting controlled human infection challenge to do with participants

Participant:	Mm, I think with good explanation gender should not be an issue good but if things are not explained properly then there can be a chance that there might be gender dispense.

Interviewer:	For the study that is intending to come for the females that are of the childbearing age who would want to participate in the study will be like encouraged to be on contraceptives for the period that they will be participating in the study, what are your views concerning this.

Participant:	My view there will be it will be dependent on the period that they have to be on contraceptives. In that regard I think for the younger ones who don’t have a family they are planning to have a family in the near future and are likely to be part of the study

Interviewer:	Yes

Participant:	But are the ones that have just started a family that have just got married probably they may be a bit reluctant if the period is long that they have to be on contraceptives and also maybe for those that they feel they are done giving children they may also be more willing to participate in the study, that’s just

Interviewer:	Your views, as part of the study TB challenge that will be conducted as part of that study participants will be injected with BCG, the usual BCG that we know that is in the EPI program this is the same BCG given to children in Malawi under vaccination policy but this one will be given at different doses to be able to collect it by taking part of the tissue from the injected site biopsy and also to understand how the body’s immunity responds to BCG. How do you feel about injecting the BCG

Participant:	I think this should not be a problem especially if people are told that it’s the same BCG that children are getting, I think on that part it may not be a problem.

Interviewer:	This should not be a problem if people are told that this is the same BCG that is being used in children

Participant:	Mm

Interviewer:	Where on the body would you prefer to be injected BCG

Participant:	On the arm

Interviewer:	On the arm, any other reasons for choosing the arm side

Participant:	Sorry

Interviewer:	Any other reasons for choosing the arm side as part of the area to inject BCG

Participant:	I think it’s easy to expose someone to inject me there and yes

Interviewer:	What is your view on biopsy on BCG injection site, conducting a biopsy on the part of where they have injected BCG

Participant:	It’s ok but then in that sense it will depend on when they take the biopsy what does it leave a big scar or a small scar that will be a concern and especially for women for cosmetic reasons in summer some women want to wear clothes that are showing the arm so then they might prefer another site because of this issue.

Interviewer:	Mmhi,

Participant:	Also, some men

Interviewer:	There are several ways of introducing BCG in the body one of them is by putting BCG in the lungs, this message uses a thin tube that passes through the mouth or nose down the throat to the lung’s bronchoscopy. This method is also used to collect samples from the lungs. What are your views on this?

Participant:	I think this one is more sounds like more invasive and I think it will be on the preferable for most people

Interviewer:	Mhi

Participant:	And the fact that one they say if it goes into your lung’s they are going to say they are going to put something in your lung’s I think that sounds a little bit more scarry

Interviewer:	So, part of the study which was done previously where they were introducing pneumococcus bacteria to adult health, there was an arrangement that the participants after they have been introduced to the bacteria they were sent to the study site, like area for monitoring and observation. What do you think about sending the participants to a residential site?

Participant:	Sending participants

Interviewer:	Yes, sending participants to a residential site where participants are kept for the first few days after they have been inoculated with the bacteria

Participant:	Well, it’s a little bit inconveniencing but if the volunteers are already aware of it are prepared are well taken care of in that residential site its ok because they are volunteers, so they have been told fully this information. But from the medical point of view, I think it’s a safe way of making sure that these patients are monitored.

Interviewer:	Mhi, for these participants who would like to join the study who should they give as someone who can be contacted in case of anything or in case like the study want to contact the participant, but they are failing to get in touch. Who can you recommend being introduced as a proxy who can be contacted on behalf of the participant?

Participant:	Their relatives I think

Interviewer:	Which relative because we have different groups of relatives

Participant:	Ok, if they are married their spouse if not then their parents

Interviewer:	For married people their spouse

Participants:	For these will be adults over 18 so either their parents or a sibling.

Interviewer:	Parents or sibling, in this case there are some who have parents their parents are staying somewhere and are living somewhere, who should be the one, should it be the one living close to them or

Participant:	The one living close to them

Interviewer:	Ok, these ones should be the ones living closer, the closer ones

Participant:	Yes

Interviewer:	Ok, I would like to ask like your personal point of view if a relative or friend comes to you and telling you that he or she intends to join a controlled human infection study, how would you feel about that.

Participant:	I will feel ok about it

Interviewer:	You will feel ok.

Participant:	I will just verify that they understand the information that they have been given, if they do, I will be fine

Interviewer:	What will make you feel like that, maybe because you are from the medical background

Participant:	Yes, certainly yes

Interviewer:	Ok, will you have any worries about a relative participating in these type of studies

Participant:	I will be worried for example maybe If I know that their health is not ok and if something goes wrong but not too worried.

Interviewer:	Mmhi, about their health if something goes wrong

Participant:	So, I mean that if I know that my relative health is already not good, and they want to participate in the study I will be worried.

Interviewer:	So, what are the things that could be done to make a TB challenge safe in healthy adult volunteers from your point of view

Participant:	I think to make sure that the volunteers are screened properly and are given information and are screened properly that they are in good health yes.

Interviewer;	Apart from that

Participant:	Nothing

Interviewer:	What things can be done to ensure positive participation in controlled human infection studies

Participant:	Giving all the information I think it’s making sure that participants are adequately informed. Having assurances on what will happen if they get sick how they will be supported I feel that I think those are the main things.

Interviewer:	Any other comments

Participant:	No

Interviewer:	So, for now I would like to ask for your personal information. I would like to ask date of birth, month, and year.

Participant:	12 July 1979

Interviewer:	Anything else that you would like to give as a comment as we wrap up this interview this morning.

Participant:	No

Interviewer:	I just must ask if you can help in giving information about taking a biopsy to participant, how best can this be communicated to a lay man in order not to scare them or to make them feel that it’s a certain process that is to be undertaken.

Participant:	I think if we explain to the patient that we are injecting, and then that injection as they will also know or as we explain that they might have seen it in children that after injecting the BCG there is that kind of swelling and then we explain that in that swelling it is now the body reacting to the vaccine and from there we want to se what the body is doing. So, we are taking this piece of tissue from there so that we se what the body is doing. I think with that explanation people can understand that this is what is happening because people want to understand what you are doing and what exactly you are looking for by doing what you are doing and if they understand that it will be easier for them to accept.

Interviewer:	Alright, on the part of sending participants to a study site for monitoring what will be the recommended number of days that the participants can be kept at the study site

Participant:	I would think not more than 5 days, because looking at the economy right now if you keep someone more than a week or more than 5 days they also are loosing out whatever income generating activities they are doing, mostly people is hand to mouth whatever they get and it will mean that now in those five days the people are loosing those things and keeping them there it means they have to be supported everything.

Interviewer:	Ok, alright I think for me this marks the end of the interview this morning. I would like to thank you for your participation and your contributions during this interview. Thank you.

Participant:	Thank you
