# Supplementary material for: Acceptability of advanced Controlled Human Infection Models: The case of a pneumococcal challenge in people living with HIV and mycobacterial challenge with Bacillus Calmette Guerin in Malawi
Source: PLOS Glob Public Health. 2026 Aug 18;6(8):e0006236. doi: 10.1371/journal.pgph.0006236 (PMC13485124; doi:10.1371/journal.pgph.0006236)
Supplement: Supplementary file 1 [file pgph.0006236.s001.zip › Documents/ACHIMA_ REVIEWER IDI_NCST_CG_24.04.24.docx]

**PLHIV CHIM INTERVIEW**

Interviewer 1:	Welcome to this interview this morning, as we have discussed we will have two sets of interviews that we will discuss we will start with HIV part, and we will continue after that we will go to the TB part.

Participant:	Ok

Interviewer 1:	Time the tablet cannot pass.

Interviewer 2:	Just still not worked.

Interviewer 1:	No, it has worked scanning the barcode.

Interviewer 2:	That’s good

Interviewer 1:	We are at the first section of our questions.

Participant:	Ok

Interviewer 1:	Where we want to know about attitude and perceptions on human challenge with streptococcus pneumococcus with people living with HIV. Have you previously heard, or seen anything about controlled human infection model?

Participant:	Aa no, I think we did is it from an insurance endorsement but not an actual study that am knowledgeable about it.

Interviewer 1:	But you just heard about it.

Participant:	Yes

Interviewer 1:	This was during like we can call it a meeting.

Participant:	Yes, we can call it a meeting. During a meeting yes, I attended I think one funders forum it was briefly mentioned so it was during a meeting.

Interviewer 1:	Ok, so to understand more about this there is a small animation video explaining more about it.

Participant:	Ok

Video play:	It’s a given vaccines prevent diseases and saves countless lives, but this takes ten years or more to develop new vaccine. We need to find a faster way one way to speed up the process is by making better use of human infection studies. Volunteers are given a sample of vaccine under development followed by a careful monitor infection medical care is always close to hand if volunteers fall ill and if they stay well, it means the vaccine works and can be made available much earlier. The idea is almost 300 years old but using todays advanced technology this type of study is not just scientifically sound but ethically responsible too and of course follows strict rules. Human infection studies show huge promise against fighting diseases like Typhoid and malaria but to develop vaccines most effective at the people at risk we need to test on those at most risk by expanding human infection studies and targeting them better the people most at risk could help develop vaccines of the future and save millions more lives.

Interviewer 1:	Ok, after watching this video would you accept conducting the controlled human infection studies.

Participant:	After watching the video?

Interviewer 1:	Yes, would you agree or disagree to conduct controlled human infection study models.

Participant:	After watching the video, I would

Interviewer 1:	You would agree.

Participant:	Yes, I would agree

Interviewer 1:	In your views what would be the benefits of conducting a pneumococcal CHIM in people living with HIV like a controlled human infection model in people living with HIV.

Participant:	I think from what I would say it has been usually said they are at risk of contracting so many infections not just pneumonia.

Interviewer 1:	Yes

Participant:	And I think recently there was a newspaper article that showed that infections are getting higher especially among young women and girls in universities it means we are still not yet there in controlling HIV. So, if we are not able to control HIV it means all associated factors are also there including the diseases that are out there, so if we are to save those people as the video said we want to protect those at most risk. We want you know getting vaccines faster then I think it would provide those benefits in good time and efficient time. Because if we are stuck with a younger population getting contracting HIV, we need to protect them for much longer years. That means we are also protecting them from any vulnerabilities’ that are out there. Because we do need the population the younger ones are most at risk, we are better of protecting them now so that they are also able to live longer. I would say those will be some of the benefits.

Interviewer 1:	So, like as part of the benefits that you have mention it’s like protecting the younger generation from future infections. What could be the risks that could be in conducting a controlled human infection study in people living with HIV.

Participant:	The risk would be there because they are usually people who have immune suppressed deficiency, immune system rather they are already on at risk. And I think because issues to do with immunity they are different for each type of person. We wouldn’t be able to know to any if we do this to such a person will they experience the same to someone else because the strength of the immune system is different for everybody. I think that will be the main risk factor that would come to my mind.

Interviewer 1:	Like risk of developing some other complications

Participant:	Yes, complications yes from the vaccine

Interviewer 1:	Any other risk that you would think of any other risks that could be there.

Participant:	Aa

Interviewer 1:	Just looking at it in general how the whole process can be.

Participant:	I think the other process maybe because I don’t know how it would also interact with the medication that they are taking for some of the people. If we do expose them to that, will they have any counter interactions with ARV’s that are there now those are some of the things that will be needed to think about. Would they be able to have the same type of protection since we have introduced something yes, they are on this type of medication will there be any type of interferences that will be I think my other thought?

Interviewer 2:	So that’s in the event that we are giving the vaccine but at the moment we are giving for example a bacteria.

Participant:	Ok

Interviewer 2:	And then the vaccine will come in future.

Participant:	It will come later

Interviewer 2:	So, what are the risks that you think that you think of giving them a bacteria which is infectious.

Participant:	I think for me then it goes back to the issue of immune system to say that we wouldn’t know how one’s immune system will act because the HIV virus it also supresses the immune system.

Interviewer 2:	Any other risk apart from complications disease due to immune compromise state, outside the body would you think of any other risk.

Participant:	Maybe to the other person depending on the living situation that they have will they be able to transfer it t someone else, outside themselves maybe it’s a viral then it means it can be transmitted to another person. So, depending on the living situation that can put another person contracting the same virus.

Interviewer 2:	Ok

Participant:	So, would we need maybe for them to be confined somewhere I don’t know maybe those are some of the things that we want to think about.

Interviewer 2:	So, I think we will explore some of those exactly you have mentioned in the discussion.

Participant:	Yes

Interviewer 1:	In your views what things can be done to make a pneumococcal CHIM in HIV safer.

Participant:	I think the trials themselves taking it from the video to say they would need to give them something which is really controlled that is safe. The trails must be in a state that they know that the risk of infection is minimized for any person. And another thing I think understanding would be very crucial part. Any potential participant has to be given that information in detail I think that will be the most important thing that will be successful, not in a very health perspective but it has to be something that is easily understandable. It’s not something that is going to be easy we want to give you, deliberately infect you with a virus so we can know. The benefits are there yes so, I think for someone to, potential participants will need to be given that information in detail well stipulated easily understood. To sat this is what we are going to be doing. And another thing I think there should be provision of insurance coverages in case of anything happens there should be covered during the trial and maybe after the trial has been finalized so I think those will be some of the success factors to the study.

Interviewer 2:	How best would you suggest we communicate that we will introduce a pathogen or an organism.

Participant:	The video for me was good, so maybe it will just need to be translated and then maybe also you should have those, there are some videos that they put real germs just showing how the whole thing is going to work. So, it will need to be straight forward communicated with that a person can just watch the video or they listen to the audio they can easily understand what is going to be happening. So, it means translations as you have done here you need to have someone else multi-disciplinary there should be social scientist there have communication specialist there, I think those will be some of the things that will make it a success.

Interviewer 1:	Ok, so you have talked about communication well detailed communication that has to be coming from specialist that are in the field providing like well detailed information to participants and provision of insurance during and after the study to participants participating in the study. Any other things in addition to that.

Participant:	I think I said that from the researcher’s point of view they should really make sure that those pathogens are really controlled, it should be at a stage where it should be completely safe to introduce the pathogen to the participant.

Interviewer 1:	Alright, what things can be done to ensure positive participant experience in Achim study.

Participant:	I would think, ok when they are enrolled it will need frequent checking up on them, I will use checking up on them but frequent monitoring, frequent charts with them a simple how are you doing. And like I said not just a very health thought kind way but a well communicated way how are you doing, how are you feeling are there you know any challenges that you are facing. I would say a psychological type of support will be much needed to help the participants.

Interviewer 2:	What psychological pressure would you think they would have?

Participant:	Fear you know potentially that if I wake up tomorrow, am I going to react badly to this or am I going to have pneumonia, am I going to have full borne pneumonia those I think would be the worst type of fear they will have. That fear to have that am I going to contract the disease maybe will I contract something else. Will I be ok maybe two years after trial that’s what I will think the most?

Interviewer 2:	How long would you suggest we follow them up to because the study for example will end within after a month or two.

Participant:	I would say maybe six months.

Interviewer 2:	Ok, anything that’s informing your choice of six month or just.

Participant:	I think I have calculated if it’s a one month then you at least follow them up for six months.

Interviewer 2:	So, what type of follow up would you suggest?

Participant:	You would call them see how they are doing, how they are faring anything give them that reassurance as well. Maybe you can tell them how far the trial how the study is going so far	that’s what I would suggest.

Interviewer 2:	Ok

Interviewer 1:	So, we are going to the next section where we would like to know background characteristics of survey participants. We are towards the end where we want to capture information about your background like demographic for you.

Participant:	Ok

Interviewer 1:	We want to ask the date, month, and year of birth for your details, would you be comfortable to share with us the information about yourself.

Participant:	Ok, no problem

Interviewer 2:	When were you born.

Participant:	August 20, 1990

Interviewer 1:	The year

Participant:	1990

Interviewer 1:	so, for how long have you been working like with NCST?

Participant:	I think this is my 5^Th^ year February 2018 yes, it’s 5 years now

Interviewer 1:	How far did you go with your education the level that you have attained.

Participant:	I have a masters

Interviewer 1:	Ok

Interviewer 2:	What’s your study, social science

Participant:	Yes

Interviewer 2:	Is there a specific field.

Participant:	Yes, social development

Interviewer 1;	Any other comments that you will share with us regarding controlled human infection in people living with HIV.

Participant:	Aa, I think it’s a good trial am excited as if am one of the potential participants. I would say it’s not going to be easy, but I think it’s dowable. It’s something that we should be able to explore new ways of developing vaccines and medical interventions, innovations. We saw how bad Covid it was how long too, how much fear we had because there was no vaccine at that time, and it took a while. And especially if we are doing it for our population here that time Covid was a global thing when they started making the vaccine they started the global north we had to wait until everything else was finalized. But if we can do our own things in good time and we can and we are looking at our own settings our own disease that affect us. For me that’s a good thing that’s a plus it’s going to be exciting that’s what I would say.

Interviewer 1:	Thank you.

Interviewer 2:	Would you suggest any other things that we could do to protect these people.

Participant:	I think maybe you could also engage with their should we say associations, the ambarella associations we know they are there we have Manet Plus we have NAPHAM I don’t know if NAPHAM is there I haven’t heard about it for a while, we have NAPHAM we have the department of nutrition so an engagement with those stake holders will also be crucial so that they also understand what is going on. Because with social media these days someone can just pick a small thing and blow it out of proportion. But if you have these people they already know what’s going to happen then that is also going to be a plus so that they can also already ask their questions they are already on their way that their members are going to be involved in the.. that’s also going to helpful for the study.

Interviewer 2;	For the actual people who are enrolled in the study you had mentioned about maybe putting them in a confinement which is already something which we already do. Is there anything else maybe the first question how long would you suggest we keep them we call it the residential stay, so they are free to move around during day go work whatever but, in the night, they must be with us the study team. Just monitoring them how are you feeling they go to sleep, how long would you suggest we keep them.

Participant:	I would suggest maybe you keep them for how long, what do you call that period I would say loosely say window period for the pathogen to develop into something that is full borne if that period is over, I would suggest then they should be ok.

Interviewer 2:	Ok, any other things that we do to protect them apart from engaging stake holders keeping them at close monitoring.

Participant:	I think the other thing I mentioned was about insurance make sure they are covered during the trial so, I think for me that’s what I would suggest.

Interviewer 2:	Alright, thank you.

Interviewer 1:	So, we are done the first part for people living with HIV like conducting a controlled human infection model for that component. So, now are going to ask questions basically for conducting TB CHIM. Some of the questions they will be like the other ones that I have asked already.

Participant:	Ok

**TB CHIM INTERVIEW**

Interviewer 1:	Have you previously heard or seen anything about controlled human infection study model, this was you heard about it this was from a meeting.

Interviewer 2:	So, some of the things will be similar like charity has pointed out but now think now giving a weakened form of Tuberculosis in this case we might also be referring it as BCG challenge, but this is the same BCG vaccine that children receive as part of immunization. But we take the same BCG because we know it is a form of TB, but it has been tampered with

Participant:	Yes

Interviewer 2:	So that it does not exactly, in our case we do not regard it as a vaccine we regard it now as a challenge that we are giving into someone and there is a potential that maybe it could develop into something else so, that’s the whole essence.

Participant:	This one is going to be given to

Interviewer 2:	Adults

Participant:	A healthy adults

Interviewer 2:	Healthy adults this one healthy adults HIV negative so we give them different doses of the BCG vaccine but using it as now a challenge agent in this case.

Participant:	Ok, would it not. Ok I know we have the vaccine so, would it not I don’t know how to put it. Since we already have like myself am already protected because I have the vaccine.

Interviewer 2:	Yes

Participant:	Would it mean introducing something much stronger than what’s already in my system or a different type of

Interviewer 2:	We might also be able to clarify but I think we will start with the basic questions. By then we plan to give different doses for a small number of participants just to see whether the model can work or not. Regarding protection whether the model can increase protection I would assume so because it’s a vaccine but just that in our setting we are regarding it as a challenge agent.

Interviewer 1:	Ok so we have the video will you need to watch it for the second time.

Interviewer 2:	It’s the same video.

Participant:	If it’s the same ok that’s alright

Interviewer 1:	You would agree to conduct human infection study model in a TB CHIM after watching the video. In your views what would be the benefits of conducting a TB CHIM in healthy adult volunteers.

Participant;	I think for me I would relate it, what comes to the top of my mind is the issue of resistance to antibiotics that we have experienced so far. So with that resistance that is there it means even the strains that are coming in are also stronger. So we also need to have that double protection, I will call it double protection in the future. And also TB has also been known some have developed they call it multi resistant type of Tuberculosis it’s very common so, it means we have those gaps that are existing there so if there is something that is going to come to strengthen those challenges I think that’s going to provide a big benefit to the TB community.

Interviewer 2:	So, just to get your thoughts if am following you, so what we are doing is to develop a model for TB with the hope if am following you that maybe we could develop something stronger that would provide more protection.

Participant:	Yes

Interviewer 2:	I think that’s the foundation of the model to say if wea are able to lay plat form for a TB CHIM we want to use the same model to bring in new vaccines so that we are able to select which ones are stronger.

Participant:	Yes, I know it’s still there because I know someone who has TB last two months. Someone from our community that thinking comes to my mind that yes TB is still there. She is a strong young lady, but she did develop Tuberculosis. She did have the challenges as well some of the medications becoming resistant but finally, they did manage to get her on something that has worked for her.

Interviewer 1:	In your views what are the risks of conducting a TB CHIM in healthy adult volunteers.

Participant:	I would look at it not so much as the HIV CHIM because it is a known factor that for TB there is some groundwork that has been there. For me I think the risk would be much less than they are for HIV because some of the groundwork is already there so we just be if my understanding is correct we just be there to advance what is already at the moment. So, to me I think the risks are there but not so much, they would outweigh the benefits that comes out from the study.

Interviewer 2:	At least you have in mind if we are to give someone a BCG challenge in their body.

Participant:	Maybe because the unknown we wouldn’t know how they would react I can say the groundwork is already there so, those risks I think they would not come as they would with HIV CHIM where it’s a totally unknown ground where we are going to break. TB, I think at least the foundation is already there so I don’t think there will be much more risks associated there outside the normal we don’t know what’s going to happen yes.

Interviewer 1:	The reactions that will

Participant:	Yes, but I would think the benefits would much outweigh the risks that will be there

Interviewer 1:	In terms of the benefits that you have mentioned that they will outweigh the risks that re there, what are some of the benefits that you think they are there in conducting a study like TB CHIM.

Participant:	Like I said even from personal experience TB is still there like I said I know someone like I said we live in the same neighbourhood she contracted TB. It means it’s still there like for me I would say I know like 5 people who I know they contracted TB before so it’s still a major challenge even in our communities. And even in our settings the way we live and everything for me TB is something that we need to attend it just as it is with the other diseases that are out there. And then it comes with complications because we have had Covid, those are also viral type of infections just as we have with Tuberculosis. So, if we are able to deal with Tuberculosis who knows that we could also come with counter runs with other type of viral infections that are there. I think that’s what I would say.

Interviewer 1:	What other concerns could arise conducting a TB CHIM in healthy adults.

Participant:	Yes, TB as a viral infection yes it spreads as well so we don’t know how, what risk would be there, but the good thing is with most of us we have the BCG vaccine. So even if we are out there the risk of transmitting to someone else will be lessened because at least most of us that’s my assumption is already protected from this virus. Just like that those who are contracting it’s coming in much stronger form as we know vaccines they tend to also should we say develop or grow they are also their makeup they become stronger as well.

Interviewer 1:	Ok, so now I would like to explore sex differences in participation. How would you feel about gender in a CHIM study participation. In our previous study we observed that more males than females had joined our study

Participant:	More males

Interviewer 1:	Yes

Participant:	Ok

Interviewer 1:	Why do you think this could have been the case just assuming to why more males	enrolled in that.

Participant:	From my assumptions maybe because of the fact that they mentioned that you have where you put them you call

Interviewer 2:	Residential stay

Participant:	Yes residential stay for more women they would find it a bit challenging because of their roles they have to plat at the house hold. They maybe think about the kids, the husband the home maybe those would be the things that would make them uncomfortable that I cant leave my house, I cant leave my kids to stay somewhere else to spend such a period of time that could be some of the factors that could contribute to that.

Interviewer 2:	Do you think there could be any other reasons, or maybe the first question would you expect to be differences in participation between males and females in human challenge .

Participant:	Yes, am actually surprised I would think I my thought I would think the numbers would be the other that more women would come up front that males.

Interviewer 2:	So, you would expect differences.

Participant:	Yes

Interviewer 2:	And expectation would be what.

Participant:	More of the females than the males that would be my expectation.

Interviewer 2:	Why

Participant:	I think for women it’s easy to I think we are more to should I say we understand things easily, we accept much easier and we would be able to say you are saying these are the risks but I would still go for it despite the risks because it’s going to be beneficial so I think women are more into that than men. I don’t know how it has played different Kikiki. But I think for me the separation that would contribute to that to say to be some where else. For the enrolment for me I would think much more women would be more up front than the males that’s what

Interviewer 2:	Because they could easily understand.

Participant:	Yes

Interviewer 2:	And they think more of the benefits.

Participant:	Yes, they think more of the benefits than the males, maybe that’s another research topic

Interviewer 2:	Maybe

Interviewer 1:	Alright so we continue for the TB CHIM that is intending to come female participants who are of childbearing age and those who will be willing to participate in the study will also be willing to participate in adequate birth control measures during the study. What are your views regarding this issue?

Participant:	Is it going to come in as an add on to the study or someone coming in they have to be on the birth control or is it that you provide the service as part of the study as well.

Interviewer 2:	So, when they come when we are giving information we say if you agree with everything, and you are willing to participate we would encourage you not to conceive.

Participant:	Ok

Interviewer 2:	So, that’s the phrase we use

Participant:	That’s during the

Interviewer 2:	Information, consenting

Participant:	I mean for how long

Interviewer 2:	Until they finish the study.

Participant:	They finish the study.

Interviewer 2:	A month to six weeks.

Participant:	Ok maybe have you done that with the other previous studies.

Interviewer 2:	Yes, and we would just say one of the requirements is we do not plan to be pregnant during the study period.

Participant:	Ok, could also be one of the contributing factors why less women are turning up for the study because the setting we are in you know issues to do with fertility they are regarded with a different type of eye. Maybe	that could be one of the factors contributing the number of women being less than those of males. But for me I would say from a lay man’s point of view I would understand it to say because if you don’t know the potential risks that will come with pregnancy, a foetus because you don’t know if there will be issues to do with transmission, so they are better off being safe not conceiving during the trial. But from another perspective maybe from them they would understand it that maybe the trial itself would have impact on their fertility issues that’s my understanding from a typical Malawian point of view. For me I think I would not have a problem like I said we don’t know, you want to reduce that risk of transmission during pregnancy from mum to child because we don’t know what’s going to happen so I would say. For me I would understand it as ok from the other part from participant’s point of view I think maybe they would not fully understand. Maybe a side I don’t know how the consent form goes but aside from telling them not to conceive I hope it also explained why you are advising them not to conceive.

Interviewer 2:	The explanation is exactly the letter you have given we do not want to put the baby at risk, but also potential transmission.

Participant:	Alright maybe you can also just to add to say

Interviewer 2:	And also, the risk to the mother that maybe she can develop an active disease.

Participant:	I think I can suggest you can also add to say it’s not really the trial is not going to have an impact on future plans to conceive if they have that so it just that you are just suggesting just during the trial but for after that it’s ok the trial is not going to have further impact on their fertility issues.

Interviewer 2:	I think I will buy that close in our next consent we will put it in it’s a good one.

Interviewer 1:	So, as part of the study, part of the study procedures participants will be injected with BCG as a challenge agent. This is the same BCG vaccine given to children in Malawi as part of vaccination policy. But this will be given at different doses to be able to collect it by taking part of the tissue from the injection site biopsy and to also understand how the body’s immunity responds to BCG. How do you feel about injecting BCG.

Participant:	Injecting it I think it’s ok my only concern will be to do with the biopsy issues. That’s also going to need complete explanation and understanding to say we are also going to collect part of a specimen from you. Not just the usual injection is ok but collection of the tissue itself is going to be another challenge. So, also need to be well explained to the potential participants and then what you are going to so, with that tissue, how you are going to store it and if you are going to dispose it how you are going to dispose the tissue. From our setting there just so many cultural beliefs simple things people would have that to say they took my meat they did this and that and so that’s also going to be a bit challenging but for the injection that’s ok. That’s part of how the trials is to be conducted but for collection of the tissues it self now an another explored area that you need to have the foundation.

Interviewer 2:	Why is it a challenge, is it the myths that you just mentioned.

Participant:	Yes

Interviewer 2:	What else

Participant:	Aa but also it means also you need to, I don’t know how big the site is going to be when you collect.

Interviewer 2:	So, it will be a few millimetres but less than a centimetre.

Participant:	Less than a centimetre

Interviewer 2:	Less than half a centimetre

Participant:	And then not so big because if it was something big there are also risk of infection and what, they would also need to run through how to take care of the site itself but for the last part I would think they are going to myths that are going to be there of collecting tissues.

Interviewer 2:	How would you propose we communicate the biopsy when we will be in your own understanding, it doesn’t be from a textbook or a medical explanation but in your own understanding how would you propose.

Participant:	In my own understanding it would be something like we will give an injection and we will collect part of small tissue from your body to test to check how it is going, my Chichewa is as typical to check how are things in the body. The body tissue we will use to just to check after we finish, we will dispose it that’s how I would put it, we will take small tissue part. I think it’s once.

Interviewer 2:	Yes

Participant:	We will take part of the tissue

Interviewer 2:	It could be multiple times.

Participant:	It could be multiple times so we will collect part of the tissue to test to see how things are going

Interviewer 1:	Where on the body would you prefer to be injected with BCG.

Participant:	On the arm, the upper arm where we usually have

Interviewer 1:	Why would you suggest the upper arm.

Participant:	It’s much more comfortable for me anyway and we are used for those sites’ injection maybe for medical type of vaccine it’s usually the upper part of the body. It will be as if you are doing things that are already being done, when you want to get a vaccine you get it on the upper arm, the upper part of the arm. I know it’s not going to be the BCG we get it the upper part Covid we got it on the upper part of the body that’s what for me, it will be easy to say we will inject you on the arm it's not really common. For me to feel secure I would feel it should also be done where other people have done it where vaccines are normally given, it would give me that sense of comfort I don’t know if am making sense.

Interviewer 1:	There are several ways of introducing BCG in the body one of the ways is by putting BCG in the lungs, this method uses a thin tube that passes through the nose or mouth down the throat to the lung’s bronchoscopy. This method is also used to collect samples from the lungs. What are your views on this.

Participant:	For me I think that’s a bit too advanced at the moment, it’s as if you are undergoing a complete medical procedure. So, it’s as if it’s a medical procedure in someone who is relatively already healthy so it is as if we are subjecting them to something that is a bit extreme for me, I would say.

Interviewer 2:	So, you think it’s too advanced too extreme a medical procedure for a normal study.

Participant:	Yes

Interviewer 2:	What are your worries.

Participant:	My worries will be you are going into the whole truck so It’s going to be the nose the throat a lot of things are going to be involved when that is being done all the way to the lungs, there are just so many things you have to go through and even the collection of the biopsy as well, I think.

Interviewer 2:	In the lungs we will be collecting fluid.

Participant:	You will be collecting fluid yes, for me I think that’s just it gives me that no it’s too advance it might be too effective but it’s just more advanced.

Interviewer 2:	Yes, what are your worries apart from involving too, I understand what you are saying is too invasive going to the lungs just to get to the sample.

Participant:	Mm

Interviewer 2:	What other worries apart from invasiveness.

Participant:	Invasiveness it means you are also increasing the risk it means you are also going through a lot because I know if you are scratching something else during the procedure	you are scratching the nose, that would also put the person more at risk of contracting other types of infections even bacterial type of infections.

Interviewer 2:	You also think there are injuries.

Participant:	Yes, there are injuries as well because for someone and still I don’t know if they are given an…..

Interviewer 2:	Yes, they are. So, invasiveness injury, infections.

Participant:	Yes

Interviewer 2:	Any other things you will be worried about

Participant:	I would think no, I think I because I remember with the Covid it was the same type of if they are getting a test they had to the back of the mind. But for this one it means going all the way to the lungs yes, we are increasing the risk as we go.

Interviewer 2:	So, what’s your take yes or no

Participant:	No, not now maybe after we have done the other options that are there then we can now move for now

Interviewer 2:	Ok, you are saying no bronchoscopy, but we can explore other ways.

Participant:	Yes

Interviewer 1:	Ok, the other set of questions that are following up are the ones that you discussed at length about residential part participants going for the study residential site, so we also have those similar questions here. What do you think about arrangement of sending participants to residential site.

Participant:	Yes, it’s much safer that way for a layman’s even for the immediate contact person yes, I mean it’s a good measure. Just as maybe it’s one factor that’s limiting the enrolment of women until we can certify that then it’s a good arrangement.

Interviewer 1:	Ok, so who can be listed as a proxy for the study participant. A proxy is somebody who the study participant has to give in to the study team as another person who can be contacted on their behalf. Who would you recommend being listed a participant proxy during a study like this one.

Participant:	Aa

Interviewer 2:	In case they are not reachable, or we want to find out if everything is fine discussions outside if the participant is not there.

Participant:	Mm, I think that one is a bit subjective it depends on how the person is living for someone who is married maybe they would prefer to have husband as their point of contact. For someone who is not it could be the most immediate person they are living with, that also depends on if they have disclosed that they are involved in this study. So, for me I would say let it be someone they have disclosed that they are part of the study so I don’t think it’s something that they will just disclose to anybody. So, the person they have felt safe to confide in let the same person be their proxy.

Interviewer 1:	Mmhi somebody they have disclosed about their participation.

Participant:	Yes

Interviewer 1:	Apart from that anyone else who they can also give.

Participant:	Then I will just be the next immediate family member I think it’s dependent on everybody’s type of setting if there is a husband there then they can make their husband if there is no husband for some that they can be living a lone maybe the parent if they are available. I think it’s just going to be dependent.

Interviewer 1:	Alright thank you, I think for these questions they are going for personal versus the general concerns in terms of what it means in participating in a TB CHIM. If someone close to you was keen or is taking part in a study like this, how would you feel.

Participant:	I think I would have fears like I said it’s an unknown but for the TB one am much more comfortable I can say because the ground work is already there as I mentioned so it will be ok as opposed to the HIV one but for this one I think that will be ok.

Interviewer 1:	A TB one it’s ok opposed to the other way.

Participant:	The groundwork is already there the vaccines are already there, there is at least a level of protection that is already existing for most of us yes, that’s what will make it less worry some I would say

Interviewer 1:	Less worrisome, what would you be worried about.

Participant:	Yes, potential risks for it to develop into something stronger or for them to contract the actual a strong version of the disease if I would say that will be my worry.

Interviewer 1:	Ok

Participant:	And then also the risk of transmission to other people.

Interviewer 1:	Risk of transmission, any other

Participant:	Nothing that’s it

Interviewer 1;	Ok, what things could be done to make a TB CHIM safer in healthy adult volunteers.

Participant:	I think for me is what I mentioned earlier before I knew that they are put somewhere else, so that alone guarantees it gives me much more thought to say that’s ok	they have to be kept some where else .

Interviewer 1:	Apart from putting them at a residential stay any other.

Participant:	Yes, because I think we are collecting biopsies so make sure they are also taken into the issues of wound care so that they are able to take care of whatever site is there so that they reduce their risk of infection.

Interviewer 1:	Ok, what things can be done to ensure positive participant experience in CHIM study.

Participant:	I think it also goes down to issues to do with consent, the information that’s going to be there, I said in the other one. Give out as much information as possible about the study what is going to be involved, the benefits that are going to be there that I think foe me would be one of the contributing factors.

Interviewer 1:	Ok, any other comments that you may have.

Participant:	Aa no I think that’s ok

Interviewer1:	Anything that you would like to share with us concerning the TB CHIM

Participant:	No, it’s ok

Interviewer 2;	Sorry to bring you back you have mentioned of biopsy having maybe being related to myths, would you have anything in mind, just anything that you think people would

Participant:	I think it’s the actual tissue itself that will be a problem people will say maybe they will sell it to witch doctors or maybe they will use it for other things for rituals and other things ,that would be the first thing that would come to my mind

Interviewer 2:	Mm

Participant:	Where will they go with my tissue, what are they going to do with it so maybe they are going to sell it or they will make something so those will be some of the myth that will surround that.

Interviewer 2:	That’s all for me

Interviewer 1:	Other than that, I would like to thank you for your time and contributions that you have make to this interview this morning it has been a pleasure to have you. This marks the end of the interview, thank you.

Participant:	Thank you very much.
