# Supplementary material for: Acceptability of advanced Controlled Human Infection Models: The case of a pneumococcal challenge in people living with HIV and mycobacterial challenge with Bacillus Calmette Guerin in Malawi
Source: PLOS Glob Public Health. 2026 Aug 18;6(8):e0006236. doi: 10.1371/journal.pgph.0006236 (PMC13485124; doi:10.1371/journal.pgph.0006236)
Supplement: Supplementary file 1 [file pgph.0006236.s001.zip › Documents/ACHIMA_CAG FEMALE FGD_08_22.02.2024_English.docx]

Interviewer:	Alright I welcome you all CAG members here Ndirande Kachere CCAP this afternoon as explained earlier the main issue we want to discuss with you about a research study that we intend to conduct. Beforewe start anything will show you animation video about these types of studies and how they are conducted.

Video play:	It’s a given that vaccines prevent diseases and saves countless lives, but this takes ten years or more to develop new vaccines. We need to find a faster way, one way to speed up the process is by making better use of human infection studies, volunteers are given a sample of vaccine under development followed by a careful monitor infection medical care is always close to hand if volunteers fall ill and if they stay well, it means the vaccine works and can be made available much earlier. The idea is almost 300 years old but using todays advanced technology this type of study is not just scientifically sound but ethically responsible too and of course follows strict rules. Human infection studies show huge promise against fighting diseases like Typhoid and malaria but to develop vaccines most effective at the people at risk we need to test on those at most risk by expanding human infection studies and targeting them better the people most at risk could help develop vaccines of the future and save millions more lives.

Interviewer 1:	Alright in short, we can say in the video they explained it is known that it takes a long time to develop vaccine. They have identified a way which is shorter that can help to develop vaccine faster. This way is through what we were talking like giving people bacteria causing diseases like pneumonia and see how body immunity	reacts to the bacteria in the body. This is a way under investigation as a shorter way that can help to come up with vaccines. So, there are other vaccines that we can say have been developed like Malaria and Typhoid using a similar way. In short, I can say that is what the video that was shown is explaining the process how it goes. For people volunteering to participate in this type of study to try on them. Participants are looked after to see if there is anything they have to be taken care of in order for the participants to be well taken care of in short that is what the video is saying reaching that point we can start the discussion this afternoon.

Participants:	Yes

Interviewer 1:	Let’s start alright, as explained our discussion this afternoon mainly we want to hear your views as people living in the community on your views on different research studies on how they are being conducted. If we can do this type of study where we give bacteria that cause pneumonia to people with HIV what are your views on this type of study

Participant 2:	When I heard about the study, I was thinking considering that this research study is not easy as opposed to other studies conducted. If we looked at TB the way a person suffers from it for a person to accept to be given bacteria that causes TB because here in Malawi people die of TB. Maybe it’s due to lack of equipments and other things in hospitals daily people at Queens are dieing at TB ward. Even when one has been attacked by pneumonia a person who is HIV positive you find that he cannot survive considering his body immunity you find they have died so as an organization I don’t know how you considered to do that. We can call it what strategy will you use for people not to loose lives after giving them this bacteria, and how sure are you that they will survive 100% after giving people TB or pneumonia bacteria because for me I can say am fit enough to be given TB bacteria later on I start suffering from TB and I need to take TB drugs they way they are you get thin from . I don’t know the strategies that you have put in place	in order that it will not affect participant in case of death what compensation are you planning.

Interviewer 1:	Alright, other views

Participant 6:	My question is on a person who HIV you does not have will give them TB injection you give the other person how many days will you keep him/her like at quarantine, what will be your arrangement

Interviewer 1:	So, your question is part of our discussion here we can say we want you and us to discuss.

Participant:	Let’s help each other

Interviewer 1:	Help in guiding us on how we can conduct the study on the procedure to follow to make sure that people are safe, there are no any risk to the people here we can say we want to hear ways that can be put in place in making sure that when conducting a study of this type people are safe there are standard procedures that will be followed during the time of conducting this study,the other questions we should be asking.

Participant:	We should be answering ourselves.

Interviewer 1:	At the end we will give you our responses we cannot start answering questions if we start answering questions it means what we wanted to discuss it will be like we have given information. First we want to hear your views you give us first your views, she has given her views that she has concerns, she has given her concern saying for a healthy person has been given TB bacteria has been diagnosed with TB the body changes you start taking drugs that is the concern. The other things will be addressed at the end is that so SS (referring to interviewer 2)

Participant 1:	I want to conquer with number 2 on the point of enlighten on the risk I feel the issue is difficult. This person is coming from a family maybe these people during the time they were sick they passed through a lot the same person decides that I want to try the vaccine. I feel there will be a battle with family members to accept them to participate. Because these people maybe have suffered during the time they were suffering from TB now they want to try on them to see when they give the bacteria how it will be. We see the risk can be there maybe she may have a heart that I will do this but there might be other hinderances the family might say that we struggled with how you suffered the decision that you are saying might not be viable, just enlightening on the risk.

Interviewer:	Other views

Participant 4:	As it has been said the risk can be there one a lot of studies have been conducted but we haven’t heard they are taking this we put in you to see how it goes this has never happened so, for people to hear this people may have many fears.

Participant 2:	I want to comment my opinion on what she said putting aperson on quarantine, I feel when you start the study just to enlighten on that we have different immunity levels in our bodies, I don’t know am not from a medical perspective but we hear that there are people with blood group O they don’t get sick or 5-6 years moving yet others like blood group B malaria every year. So, body immunity is different so I don’t know on that when conducting the study, you will check a person’s blood group because you can give TB bacteria to a person who is well of blood group O who has strong immunity you can keep them for 2 -3 days that you anticipated to have signs for TB to show. Or maybe it may happen the person does not get sick maybe after 6 months or one year after the study has phased out, the project has phased out a person getting sick I don’t know on that that must be investigated when starting this study. At least a person you have picked up don’t be with them for a short period of time but take them for a long period of time until you make sure that TB bacteria did not survive in his	body’s immunity for them not to blame you.

Interviewer 2:	How long do you think we can keep this person.

Participant 2:	I don’t know medical personnel you can help us you can take example of a person with blood group O can contract HIV virus can stay with it in his body for years without getting sick, yet the virus is in his body yet other blood groups after contracting it 2 -3 months going to hospital, they can get it. So, it is the same as experts according to how you look at blood groups of people, I think maybe a person we can be with them for a year or two years I think we can trace TB or pneumonia, or it has not been traced that he has survived I think so.

Interviewer 2:	Thank you very much.

Interviewer 1:	Alright we can continue.

Participants:	Yes

Interviewer 1:	Just want to know on the information that we have provided at the beginning I want you to share with us the message that you have captured specifically.

Participant 2:	Alright let me start I have got that it’s a study that yoy want to see how bacterias react in the body, you have two groups of people those with good health status and a person who is HIV positive. Those that are HIV positive have heard that you will test them with pneumonia bacteria those that are healthy you will test them with TB bacteria. The aim is to see when we were children we were vaccinated with TB vaccine to protect us not suffer from TB but because TB isa air borne disease after growing up we find when you are healthy like this we find you are suffering from from TB, you want to see how it goes in your research study as study aim maybe that as a person grows we boost TB vaccine. Or for those with HIV you give them pneumonia injection as they give to reduce pneumonia cases, for my part I have heard that.

Interviewer 1:	Alright, others let go she has explained her part how we understood, what message have we got.

Participant 4:	I have got research information ever since I have not heard of this research heard about it today it’s all about learning.

Interviewer 1:	Alright, others or we repeat the first information.

Participant:	The information is clear

Participant 3:	And for me what I got mainly like you doctors after considering a child after getting TB vaccine before they are discgarged from hospital. After seeing that as we grow, we find a person suffers from TB when health personel see that they tried their best to try maybe to find a study to find out what is causing that.

Interviewer 1:	Alright, anything in addition on the information that we have got.

Participant 1:	Yes, I just want to add on what my friend number 2 enlightening we heard that participants are from 18 years and above. To enlighten what we already said when a child is born is given vaccine it shows with time the vaccine is no longer effective that need to be looked into from 18 years maybe to say that you health personnel you have seen that TB is still affecting these ages mostly going upwards that have given you insights if there can be a vaccine that can help for those that are 18 and above when you consider the vaccine if you can get evidence after conducting the study that maybe there should be a fixed vaccine to save lives in my understanding from what I have got

Interviewer 1:	Alright thank you very much for the views that you are sharing with us on this issue that we are discussing this afternoon. We can continue just want to which message/information do you feel is the most important in this type of study.

Participant 2:	Before a person joins the study there is need to enlighten clearly on benefits and risks of the study because the benefit I see as a person for this to take place it means somebody took a risk. Even to be at peace John Chilembwe was killed, he was beaten up he fought for freedom of this country. Meaning to say a person participating must accept that he is fighting freedom for people. Resultstaht will come on my life be it I get sick, or I will be ok it will help other people to get vaccine that will protect people. If results come out that people need to boost vaccine the message has to inform people should be told clearly about benefits and risks of the study before they join

Interviewer 1:	Mmhi, another important message she has started another important message.

Participant 3:	Another important message I feel when people come to the clinic, they must be told about these studies like teaching them so that when they grow, they must know that there are these vaccines that have been introduced whereby they put bacteria in the body the aim is for people to learn. Why am I saying that because people these days they think too much. Maybe us as well we haven’t heard about it, we heard it from here saying that disease causing organism in the body at the hospital they teach about different things to educate people that sometimes when we want to fing drugs, we have to do this, sometimes to come up with vaccines we have to do this, this is very important. Maybe our friends from outside countries they treach them they can read books yet us here we just stay even at school we don’t learn science there that there are other diseases they put disease causing organisms to come up with medication, we put bacterias or we remove this to come up drugs this is important.

Intervieer 1:	Ok alright another important message

Participant 6:	Just want to add	not at the hospital or even in secondary schools the message can get there even in churches the message can go there.

Interviewer 1:	Ok alright, we are discussing about important message that can be given or the message that you feel is important about this study, there is any message that that can be given as part of the study or the message that you have been given from the today’s message.

Participant 2:	Yes, telling people the purpose of the study as one way of informing people to join the study because when people know the aim of the study this can help developing our nation in the health sector. Another message will be informing them about aim of the study what is it that you want to achieve after the study.

Interviewer 1:	Ok thank you very much for your views, other views or we can proceed.

Participant 3:	Another message on the radio we saw MLW coming on radio they can give information that other studies we do this, other studies are like this, people should not be afraid because all this promotes our health.

Participant:	Or mugha phone announcing about this

Interviewer 2:	Your number, raise your voice.

Participant 6:	On community leaders we can have meetings enlighting on these things.

Participant 1:	To add on that sometimes messages on phones on health issues apart from that flyers/paphlets distributing to people for people to read because if two or three know something if they know the right information and someone is not sure they can share with that one real information the other person knows through a friend.

Interviewer 2:	Thank you very much for all your views it seems we are moving together, we can continue do you have questions that have not been answered that you want to be answered from us, I know there are question aunt asked have kept it will respond it if there are other questions that we want know other things related to the study

Participant 6:	My question I want to ask how many people you need in this study.

Interviewer 2:	Ok how many people will we need in the study (could not hear what participant was saying was speaking in lov voice)

Participant 3:	When a person participates you will be looking after him for how many years or how many months will you be looking after them in the study.

Interviewer 2:	Ok alright.

Participant 3:	You mentioned that after a person join this study you will be looking after them like admission or at home or he will be the one following you on that I didn’t understand. Does it mean everyone who is not sick will not come to hospital like me I will go to there to sleep for three days I want to know that?

Interviewer 2:	Ok thank you that question our friend will help us let me address other questions that you asked. First question was to do with we are giving bacteria to a person who is healthy, we are giving TB bacteria how sure are we that the person will not get sick or die if a person dies will there be any compansetion. First when conducting a study, I want you to know that the bacteria that we use to give people are not exactly as the ones we know that makes people sick. This means that the bacteria that we can give for TB if we test it will not be the same as the one with a patient who is in hospital the one, we use is not as strong as other one the reason we should not have risks that we can think of like death and other things. That is the reason I said we have been conducting these research for many years in healthy people, but in the people we conducted the study there is no one who got sick that they went to hospital my collegue is the one who looks after participants when they go to residential site she can explain better maybe if people got sick on what has been reported so far are flue maybe sore throat but not serious illness for a person to be admitted that has not happened. That is because we give a weaker bacteria different with the other. In terms of death compensation that will be a bit difficult to respobd now because we haven’t met with a situation like this that someone dies what we put in place is a person can get sick how can we do that people are safe not that making people sick so that one we will take it as an important point to discuss when we go back. There was a question about residential site how long people stay at the residential site, a question was asked how many people will be needed in the study how long the study will take. On this point we are still on planning we have not started the study because we are just thinking we can so far, we want 100 or we will do the study for one year (participant phone ringing). We were thinking of doing it for one year like if we can start this year and finish like next year it will depend on what you are going to tell us now if the study can be safe conduct, it or do not do it have, I answered your question.

Participant:	All yes

Interviewer 2:	How long do we look after the people in a study for them to be safe for a year or years as already said that the bacteria that we give people is weak, when we are getting people to join a study we conduct test to check level of his immunity if he is ok if the body immunity can manage to fight the bacteria in the body. When we see the immunity is ok we give the bacteria when the bacteria has been in the body for a while in short when body immunity have failed to fight back the bacteria on the last day when we are exiting them from the study we test them if the are still carrying the bacteria we give them drugs to take you join the study healthy you should go back healthy as well. We follow them up for 3 days later after giving them drugs we know by this time the bacteria is dead, we never had a situation that we give a person drugs and the bacteria still remain it will be the first time this has never happened that is reason we are here to discuss if it can be like that but according to the experience that we have had in all the previous study. When people join a study, we send them back in good condition with what you are saying we will continue looking after them to add other few months or a year we have recorded that that is important we will discuss about it. I give ny collegue a chance to explain about residential site on how long they stay there thank you very much.

Participant 1:	Before she explains I just want to know at the residential site do you allow guardians to be there apart from people who look after them, guardian lets take example of a person who has joined study you have recruited him do you allow guardian to stay with him or how is it, I wish to know about that.

Interviewer 2:	Ok thank you very much you can start.

Interviewer 3:	We are through with questions about residential site where we keep people that have joined study, I can start answering.

Participants:	All yes

Interviewer 3:	Our studies I believe you have heard there is astudy where people are sleeping at Grace Bandawe, you’ve nener heard about it.

Participants:	Mm

Interviewer 3:	You never heard that there is a study people are sleepinga at Grace Bandawe, we have been doing the study from 2020,2021,2023 and 2024 count how many years. Where they sleep is at Grace Bandawe do we know Grace Bandawe hotel they sleep there for 3 days. The three days it can be I will go in deatil about (33:16-18 audio inaudible) but I will explain. The time they introduce bacteria the people have been tested if they have any disease, they test all that to check if the person is eligible after checking all the other things when the results come out it’s when a person is given the bacteria/ Let’s say a person has been given bacteria on Monday, on Monday is when a person goes to Grace Bandawe. At Grace Bandawe as the nurse has said am the one looking after participants. When they go there Monday evening, they go home during the day whether they work or they go to school we have school students, working class businesspeople all of them they come only in the evening spend a night at Grace Bandawe. The reason is let’s say we have enrolled 4 or 10 people whom we have give the bacteria the other one stay in Ndirande, Kachere the other one stays in Chichiri I should say they stay in different places within Blantyre. Let’s take example we have everyone to be at his home as I have said that sometimes people can get sick, it can be illness related to bacteria it can be to do with other diseases it will be difficult for us to go to Kachere and other places that is the reason they thought of keeping people at one place for three days. That means Monday, Tuesday and Wednesday they will go back on Thursday morning like ten o’clock is the time they check out from Grace Bandawe.When they looked at it they saw that the bacteria remain active the first three days everything related to the bacteria that maybe a person has headache, flue the illnesses are headache, flue maybe itching of throat, loss of voice that can happen within three days after that I have never heard something happened to someone as explained the illnesses are the same. For headache the doctor recommends giving Panadol when we suffer from headache, we take Panadol, Brufen what ever it is we give participant he gets healed it does not continue. As explained, we never had an incident participant getting sick at Grace Bandawe the reason, they go there is because of Mwaiwathu hospital the aim is if they get sick, we take them to Mwaiwathu or even in the middle of the night we take all of them to Mwaiwathu. Another question that was asked is do they have gaurdians from home, home guardians are not allowed.

Participant:	Alright

Interviewer 3:	We don’t allow guardian from home I am the guardian.

Participant:	It’s like a hostel

Interviewer 3:	Not like a hostel because everyone has his/her room, everyone has his room.

Participant:	People you don’t know each other.

Interviewer 3:	We make friendships there, because we don’t know the other person you can’t sleep with him that the reason everyone has his/her room. They stay eat and bath right there everything is provided right there. In the morning one can leave at 5 o’clock if he goes to work early.

Participant:	When it’s past four you are at work.

Interviewer 3:	Yes that is ok they work during day as it easier lets take during day am sick while at work I can make a phone call it can happen quickly unlike at night some places are far even for a car to reach there that is the reason we say during the day a person has to call doctor am not ok, the doctor will see what to do but during night it can be difficult I believe I have answered the question.

Participants:	All yes.

Participant:	Can they stay for whole day.

Interviewer 3:	If they want, they can stay.

Participant:	Not restricted on the day you are off.

Interviewer 3:	All the three days we book rooms the room is for participant he can stay for all the three days he can go home to do some other work in the evening 5 o’clock like now from here am going there.

Participant:	All at the hostel they should stay one week that is not strange.

Interviewer 1:	Alright we can continue.

Participants:	Yes

Interviewer 1:	I believe questions have been answered as we go we will be moving with discussion, welcome to our discussionfeel free when you want to give your views/opinions mention the number I gave you and give your opinion on the message that been given we want you to guide us on which message that we need to on the message that we will be explaining to people about the study

Participantt 2:	The message the nurse was answering saying that the bacteria is not strong enough as the one we know in patients because at first I was scared that even if it was me I cannot accept. The way I see a TB patient you can fel sad she has explained bacteria is weakened in the study they have to be told so that they are not afraid.

Interviewer 1:	Other additional information that we can add on the information.

Participant 2:	This one had a question the other time she was told to wait.

Participant:	Have been answered

Interviewer 1:	Alright we can continue additional message that they have given us they have said bacteria is weakened that has to be incorporated as part of the information that we are explaining to participants not so

Particiupants:	Yes and telling them this is not risky and encouraging them.

Interviewer 1:	Anything

Participant 3:	I just want to add on what you said during Covid time people were saying when you receive Covid vaccine you get weak, you swell that people take that as real but after people started receiving the vaccine it was found out there was nothing that was happening. People were saying on social media someone collapsed this happened to someone, but it was a lie. So, when giving the message/information as it has been said by ny friend that the bacteria is not strong it can not cause harm in a person that has not happened.

Interviewer 1:	Mm alright, thank you very much.

Participant 1:	To add not so as it has been said by the guardian, she gave a positive encouragement shehas cleared that a person can go to work that message if you tell people they can see that it is good unlike to hide the message that things go like this. People will say bacteria they weaken it as you have said you keep the person just in case to be close to hospital so that we may be enlightening them on anything that can happen with experience that you have had nothing has happened. That message must be there for example people that have been having there is no other problems that you together this can make people that this is not scarry.

Interviewer 1:	Alright what do you think can be the benefit of conducting pneumococcal study in people living with HIV.

Participant 2:	Benefit to these people I feel people with HIV they face different problems because of state their immunity the benefit can be making sure that, because you are keeping them and you have put bacteria in them that can compromise immunity if you can after them giving them enough food during the time they joined the study they have to be in your hands in terms of food.

Interviewer 1:	Alright other views.

Participant 3:	Other thoughts I feel like people who are HIV positive they have the chance not to suffer from pneumonia because you will be looking after them how is the person what type of treatment they can receive.but I can say he is luck for them not to suffer from pneumonia or they can get healed faster.

Interviewer 1:	Alright another benefi that can be there.

Participant 5:	Like giving example the way, they did with Thyphoid the time they were giving children vaccine when they go to hospital they don’t go on queue as a benefit because a person will say I will not long to be on queue.

Interviewer 1:	Alright, that side.

Participant 7:	I feel like the benefit that can be there for these people is that in future there might come vaccine to protect them not to suffer from pneumonia.

Interviewer 1:	Alright

Participant 6:	I see it as a potential benefit for a person who does not have HIV is given vaccine, one who has HIV will be given TB vaccine you said when conducting a test, you will carry other tests he can benefit like to protect his life.

Interviewer 1:	Alright for she has gone on TB side I started asking in terms of HIV lets go on benefit in terms of those people that will be given TB bacteria what benefit can be there.

Participant 4:	Maybe the person ever since has never been tested he doesn’t know his status, but he looks healthy when he goes thereafter testing him they find him with other disease he can know from there

Participants:	All ok

Interviewer 1:	Saying benefit that when they know other problems they have they did not know.

Participants;	All yes

Interviewer 1:	Another benefit that can be there for a person who is given TB bacteria, the benefit that can be there.

Participant:	They have never slept at a good place, they will sleep at a good place eat good food there.

Participant:	When they go to full board there.

Participant:	They will sleep laughing when they go to a place, they did not know but now they will see another benefit of sleeping somewhere else.

Interviewer 3:	You did not hear?

Interviewer 1:	Alright that is the benefit.

Participant:	Yes

Interviewer 3:	When you join study, you go there that is what they say benefit.

Interviewer 1:	Alright

Participant 3:	Another benefitis that in your life you will have history saying that I participated in research where they were giving bacteria, they find me ok at Queens hospital and I participated in saving other people.

Participant 6:	It will be as if you are doing campaign to other people encouraging them on future coming studies in communities, they should not be afraid they have to be brave participating in studies.

Interviewer 1:	Alright

Participant 1:	Another benefit is that if the study can be successful it means that it will help a lot of people, to them they will be proud that I participated if I did not go there my friends could not have this chance just because I sacrificed to take part no matter what will be there I have accepted to take part the success that comes when it has been accepted he will feel proud that a lot of people will be saved because am one of the people

Interviewer 1:	Thank you very much we have talked of different benefits that can be there to conduct study in people with HIV and TB. Let us come to what you see as risks that can be there or what risks can be there conducting this research, we will start with a person living with HIV. Risks that can be there, she already started first risks that we see that can be there.

Participant:	Silent

Interviewer 1:	Or with what has been explained risks that we can think of as explained or there are no any risks.

Participant 1:	Risks are there first I think I mentioned saying that for example myself am in a family I have made a decision to take part in the study automatically I have a wife be it my parents lets say my husband there will be misunderstanding saying you know how you are you want they do that to you that is risky there will be still a war that cannot be avoided where there are two or three to pull you back on the decision that you have made that is one of the threats that will there that we can face

Interviewer 1:	Alright in terms of risks when a person has been given this bacteriafor pneumonia a person who is HIV positive when you look at a person who has HIV what risks can be there following being given the bacteria.

Participant 2:	The risk that can be first if a person get sick with already low body immunity on that how can the survival be.

Interviewer 1:	Ok, the first risk if he gets sick what can be the result another risk, here let’s think of what we think we can face that can be risk.

Participant 3:	Another serious risk is that people we don’t understand about vaccines or research studies. For example, a person who has HIV Aids you give the bacteria another risk can be relatives when a person dies, he was already sick, but they will not look at that a person was sick they will look at as the drugs have killed that will be the main risk.

Interviewer 1:	Alright

Participant:	Let’s move

Interviewer 1:	In terms of TB a healthy person who has been given TB bacteria the risk can be there, what risk do you think can be there.

Participant:	Ok I have heard

Interviewer 1:	Your number

Participant 5:	I heard that a person suffering from TB has to be isolated I heard that so when he has been given bacteria he has to be isolated or he has to be with friends he cannot transmit TB.

Interviewer 2:	Ok what you have said is true because a person with TB, TB can be transmitted through air that is the reason we said the first three days we look after them we take them as crucial that is the reason we take people to research study residential site.As we said that the bacteria that we give is not very strong that is the reason we allow people that they can go to their work and come back that they can stay for a month at their home we feel like the risk is minimized unlike with real TB that is in hospital we just keep them for few days after we release them to do their things

Interviewer 1:	Alright let’s move on

Participant:	To addthese days I see like for TB those that are critically ill are the ones admitted these days when you are feeling better with TB you can walk, they just give the drugs.

Participant 2:	Yes, I have seen most people these days are just given drugs to take at home maybe if they are admitted it’s just some days maybe 2 days.

Interviewer 1:	Alright let’s move on

Participants:	Mm

Interviewer 1:	So, I want you to look at risks and benefits that can be there conducting a study of this type what are your views reviewing if the study can be conducted or not.

Participant 4:	The study can be conducted others may agree others do not agree the study can be conducted.

Interviewer 1:	Alright now I was saying in your agreeing you should consider that there is a risk and benefits looking at the two things which side overweighs the other between benefits and risks for this study to be conducted.

Participant 7:	I feel the risk can be there the study can be conducted but for people fear can be the issue considering that am healthy they take a bacteria give me maybe I can get sick that can be the problem, they will doubt to accept if there can be strong teaching that is possible.

Interviewer 1:	It’s possible now am asking how you look at it as you have said that benefit is there you have explained that maybe people who have participated maybe in future this may help a lot of people to get vaccine or drugs through conducting this type of study. In terms of risks, you have mentioned that maybe a person can fall sick so, I want you to look between benefits and risks what over weighs the other that this house can approve the study despite having this, but this type of study can be conducted.

Participant 2:	It shows that risks are few benefits is more considering results of the study to be conducted, you were afraid from how I understand the study is not scary with past experience as explained people don’t get sick. The study is not risky there are more benefits in terms of health it will bring results that will bring vaccine that will benefit everyone more than the risk. Benefits are more in terms of bringing positive results to bring vaccine that can help.

Participant 3:	Am also saying there are benefits first my friend said that even John Chilembwe died for his country we are people who will have successful stories saying that we did that.

Interviewer 1:	To show that you are giving encouragement that the study can continue.

Participants:	All permission to proceed.

Interviewer 1:	Alright in other studies of this type have ben conducted in Malawi using pneumococcus in healthy people, how can you differentiate this study with this one in HIV people. The study has been conducted in healthy people that did not have any disease now we want to differentiate with a study like this and this study that we intend to do in people with HIV the difference that can be there how can you differenceate it.

Participant:	Silent

Interviewer 1:	The question is not clear.

Participant:	Understood

Participant 1:	I will just try.

Interviewer 1:	Not trying in our disxussion there is no right or wrong answer everyone is giving her views so views can be different from that one that is an important point.

Participant:	I was enlightening that this one has HIV maybe there might be a minimal risk because his body is compromised already while this one is healthy the bacteria has gone but it will not be as the same as this one whose health is already compromised that’s my opinion

Interviewer 1:	Alright

Participant 2:	I see that it can be the same because people these days people who are on ARV’s when they are adhering to taking drugs I hear that their viral load get low, their viral load is ok because their immunity is strong it can be similar because they are healthy these days are different even a person who is positive you cannot see him with eyes the same as HIV negative person can do he can do everything that HIV negative person can do so, it is the same that maybe you want to see the reaction in a HIV positive person and those who are HIV negative

Interviewer 1:	Alright before we move on any opinion.

Participant 7:	I see that this study those who are healthy are the ones who can struggle with the bacteria because his body is like healthy, and you have introduced something in the body yet the other one maybe his body struggles at some point in time because he is taking drugs, and his body will not get sick it can fight back.

Interviewer 1:	Alright let’s move on I want you to share with us on the issue to do with risk that can be there in this study, how can we explain these risks that can be there, how can we explain the risks.

Participant 2:	I believe the time we were starting we were afraid because we did not understand you asked about how we can deal with the risk we answered saying that when telling people about the study content in the consent form that you give them to sign add to the information that the bacteria is not weak, it will not be strong as you can think of that to reduce the risk to everyone. All the important information must be on the consent form so that there is no risk for people participating in the study the study is not scary.

Interviewer 1:	Mmh alright another way how we can explain about the risk, or it is like that.

Participant 7:	I feel like when explaining don’t put too much scary words because that can give more risk that if we do this this will happen, don’t put scary words just give only words of encouragement.

Interviewer 1:	Alright

Participant 7:	It never fails there are other people if you explain to them, you ask questions that you can explain everything but make sure that explanation should have limits don’t mention everything about risks no because they have given you bactreria they have to look after you for anything that can happen. They want to check on what can happen but mainly there is nothing that happens.

Participant1:	Maybe to add on that as guardian has said that is one part of encouraging them that we were once in this group this is good there are not any problem, but people can feel free to go to work and come back. Coming here we just want to be close to them we can’t rule out if there can be a problem so that we can help them but doing this we found that we are done with themthey are still healthy they are walking maybe just to help.

Participant 6:	I feel like for a person who participated like when doing sensitization meetings those ones take them, they must testify to encourage people to remove fear that I joined they should be like encouraging them to remove fear.

Participant 2:	Meaning role models not so.

Participant:	Mm

Participant 2:	It can promote for many people to join.

Interviewer 1:	Alright let’s move to the next section where we want to discuss differenciating on interest in taking part in this type of study between men and women. How can you feel a woman or a man deciding to take part in this type of study where they give bacteria that cause diseases.

Participant 3:	Like if it was me, I can feel good like us CAG members we are one of the people who encourage, enlighten what we said encouraging people to participate in a study be it a woman or a man.

Interviewer 1:	Between women and men who should participate in this type of study.

Participants:	All everyone.

Interviewer 1:	There is nothing that make that for this one is male or female.

Participant 2:	It’s for everyone just that men sometimes are difficult even if you can take statistics of people who have gone for HIV testing for men and women, for men to make a decision to go for HIV testing it means he is ill he has been carried to Queens he is on bed admitted for them to walk on their own it is difficult. So, just take it that men their numbers can be low women can be high, it’s not that women are easily taken no but we understand more than men.

Participant 1:	And the evidence is that statistics in hospitals we hear that in hospitals there are more women, men mostly they get sick, but they don’t go to hospital. The issue is that women can feel free when they want to make a decision a the husband can hinder or he can scare her but the study is for the both sides.

Interviewer 1:	Alright meaning that everyone male or female can take part.

Participant 2:	I want to add I see that maybe if it’s a child joining the study who is staying as dependant consent has to go to the guardian who is keeping him regardless that he is 20 years old, because one can be 22 years but is a dependant when you say you can make accept , the person who is keeping you is not aware you go to Grace Bandawe what is there, am doing this as parents they cannot understand. So, consent should not take that a person is 18 years if he has agreed to join if he is a dependant, I live with my uncle, uncle must get the consent he has to accept as explained like my child has decided I have accepted because he is a dependant.

Interviewer 1:	Alright let’s move on, let’s come on the issue that we were discussin for, in our previous study that we conducted of this type of study for example you it showed that we registered more males who joined the study than females. Why do you think this was so?

Participant 2:	Women are the ones who takes care of the home so they can consider that I have a child for example myself I have a one year and months child, because am used to leave the child I work I can leave the child, but think of a house wife has 2,3 children to leave home going to Grace Bandawe for 3 days has left children at home she will be thinking about that. Because this study women did not take part because they will be thinking of leaving the family child. Women are so loving with children unless if there is another way that they should not sleep there or if this cannot happen because they are on quarantine maybe they can transmit to children so, on that men can participate.

Interviewer 1:	Mmhi, why

Participant 3:	Another reason the money that you give helps, I saw it during Covid 19 time instead of having 5 people we find that 20 men come to join study to indicate that money attracts people to join study.

Participant 2:	Transport allowance

Interviewer 1:	Something else that you feel can make more men to show interest in joining the study, here we are just enlighting maybe is this one more thing.

Participant 1:	I see the addition is money that is all that is what men were thinking if I stay here, I will leave something for my wife if I go with something to buy relish because if are to say the truth a man is difficult person when it comes to medical issues.

Participant:	For example, with circumcision most people were asking what benefit we are going to get most men were asking are you still giving that

Participant 2:	We have been tested HIV to get 2000 (laughing)

Interviewer 1:	Alright let’s move on in the next research there is hope that women of childbearing age those that will be willing to join this study are expected to practicefamily planning during the time they are in this study, what are your views on this arrangement.

Participant 2:	Oki have a question to you, a person who is on childbearing age should practice family planning when joining the study or they have the right to practice family planning if they already do that.

Interviewer 1:	Ok on that a person who is on childbearing the time she has made decision to join study they have to make a decision to follow family planning it can be they already practice but during the time they are in the study the aim is not to get pregnant during the time she is in the study what are your views on this arrangement

Participant 2:	Ok that is understandable because a person who is pregnant somehow, I see like body immunity is not ok you can be suffering from other things if they get pregnant somehow it can be difficult. She can be suffering from something you will see it’s the study. So, it is necessary that a woman who has accepted that I want to take part in the study if they are not on family planning, they must be on family planning should not be expecting.

Participant 6:	Just to ask a person has delivered can you join this study.

Participant 2:	After giving birth.

Interviewer 2:	In	our previous studies that we conducted a person who has just delivered or has a child who has not reached 5 years we don’t allow them to join the study.

Interviewer 1:	Alright let’s continue issue of sending people to residential study site after joining study we just want to know on this idea what is your point on this arrangement is this feasible or not.

Participant 7:	It is a good idea because as she explained if something happens during the night maybe transport is an issue for the person, we can find that it’s taking long time to get treatment unlike keeping them if something happens, they will receive treatment immediately.

Participant 2:	Want to answer one by one you will be following a person even during the day it means they will call to follow them up, you find a person does not have a phone is sick is failing to get in touch with people that is the problem.

Interviewer 1:	Still on the same in your views people who will participate we start with TB study at residential site the people should be there for how many days after joing study.

Participant 6:	We can not force you you are the ones conducting research.

Interviewer 1:	We want just your views; we should start with those people that have joined TB study we can keep them for how long.

Participant 3:	My thoughts I think those that are HIV positive you can keep them for 5 days maybe healthy people whom you have tested that you we did not find you with any disease we keep them for three days.

Interviewer 1:	Ok

Participant 6:	Me too I feel like those that are healthy will be at risk because you are putting a bacteria in his body which was healthy you are like putting something in them that one you can give 5 days, the one with HIV you give vaccine for pneumonia that pne can stay 3 days as his body is like already used laughs vise versa

Interviewer 1:	Other views how we look at it.

Participant 2:	I feel like the days have to the same regaedless how one’s status is maybe instead of three days you can add that they stay 5 days you already convised us saying that on the 3 days you see the person did not get sick.

Participant 4:	I feel like you did not start this you have done it I feel like when you were setting 3 days it was not difficult it is better to go by the way you used to go in the past.

Interviewer 1:	Alright any additional views or different views, we want to go on stusy activities. The main issue before we continue, I want to know when a person has joined a study who can be his proxy during the time he is in the study, another person I have joined a study who can they give to the study team as proxy that I have joined study. Another person who I can give who can be contacted in case of anything has happened the study team want me, but they are failing to get in touch with me, they can contact the other person as proxy that this will be my proxy who can this be.

Participant 5:	I feel a person must feel it in his heart, must name the person.

Interviewer 1:	We want for you like these people whom they can give as their proxy should be people not just any other person or there should be specific people and reasons to give them as proxy and for what reasons.

Participant 2:	Aa I was thinking for people if they are married if it’s a woman must give her husband, if it’s a man the wife can be. When a person gets married when the husband is sick the wife is a guardian his relatives will hear about it later, when the wife gets sick the husband is the one who runs maybe they can ask to come. If it’s a child who is a dependant his uncle, aunt if he has his mother in the village but the one who has brought him in town not the mother who is in the village. When consenting make sure you are the mother who must consent, he is my husband. You are a child/dependant a student who must give consent is father, mother or guardian must consent.

Interviewer 1:	Let us move we go to study activities as one way of the study participants taking part will be given BCG injection as away of introducing the bacteria, here we are talking of group of TB people. This BCG is the same as the one child get in Maalawi as part of vaccination policy, nut this will be given at different doses to be able to collect different samples like collecting tissue to test from injection site to check if BCG is present on the injected site. How can you feel injecting BCG?

Participant 2:	Injecting is easy but taking part of the tissue to have a wound that is a sacrifice because the wound will remain with you.

Interviewer 1:	You said injection.

Participant 2:	Injection there is no problem but on taking tissue sample that maybe painful because the wound will still be on the skin bodies react differently.

Participant:	Others are slow to heal, others some their wounds are slow to heal

Interviewer 1:	Alright on this procedure of collecting tissue for testing on your part how do you look at that.

Participant:	There is no other way to avoid taking sample tissue.

Interviewer 1:	We should start one by one mentioning the number.

Participabt 2:	There is none maybe you should consider if there can be another option not taking part of the tissue. Maybe you should take like grands your medical profession you know other ways of testing.

Interviewer 1:	Alright

Participant 7:	Wanted to remind the same apart from taking part of the tissue.

Participant 3:	I feel like taking tissue part is not strange he hears after delivery they take part of placenta for cancer and other things. If a person is well informed, they cannot complain because even when removing a tooth, they inject anathersia we don’t feel pain you find that the tooth has been remove, I don’t know how you will collect the tissue. Will you inject anethersia or you will use spirit only, if you can explain to people in detail, it will be easy because most times, they collect tissue sample for testing.

Participant 6:	Before you continue some people whom you interacted with after 3 days where you collected the tissue was healed.

Interviewer 2:	Maybe let me respond in this way a study to do with TB this will be the first time we have never done it, where we collect body tissue this is the first time.

Interviewer 1:	She has answered the question not so

Participants:	Mm

Interviewer 1:	The procedure of collecting body tissue to test to check if BCG is present how can we explain that to potential participants in Chichewa help us to structrure clear Chichewa that we can use when explaining about this procedure, how can we explain it.

Participant 1:	I believe the Chichewa you have already explained it the issue is the same with our study we need to collect part of the body tissue to test for our study I believe if you can explain that to aperson he can understand during the study he/she can see that as strange no. They will know that it is part of the study as explained by number 3 that collecting body tissue is not an issue in the past it was strange but because of increase in diseases that are coming taking part of the body tissue these days most people hear about it, we hear they are collecting body tissue at Mwaiwathu to test at Mwaiwathu because this news is common this will not be strange to people.

Participant 3:	We have heard that this person is suffering from TB.

Interviewer 1:	No, for TB they are healthy.

Participant 3:	Aa that’s just it.

Participant:	Number 3 you were saying those that they will collect body tissue are healthy

Interviewer 1:	Yes, those that are given BCG like for TB.

Participant3:	However just add to it in the explanation saying that in the past we saw that most people were getting vaccine, but still more people suffer from TB as they grow, we want to check what is causing this yet a person already received TB vaccine

Participant 6:	I will go back a little bit you asked another question you asked our views on how many days, for these ones you can say 3 days as you did with the other one that did not involve collecting body tissue, my opinion is that if you can add 2 days to make it 5 days to see where you collect the tissue to check how it will go.

Interviewer 1:	Oho

Participant 7:	I just want to, maybe if I did not get you like saying when the study is conducted you will inject BCG, they stay 5 days at residential site to check how they respond when they will be going is when they collect body tissue not so, or body tissue will be collected in between the 5 days.

Interviewer 2:	No, after staying for some time because we want to see after some time if BCG will be present.

Participant:	Ok

Participant 1:	Ok after receiving BCG you will give a space for how long for them to come to collect body tissue to check if BCG is present.

Participant 7:	I feel like another way for people not to be threatened on the body tissue that will be collected. Just telling them the time we were injecting you the vaccine we introduced TB bacteria so, we want maybe to get, it can bring.

Participant:	No

Participant 2:	My opinion I believe in the truth this is medical tell people the truth it must be his choice saying he will join study or not without hiding the risk or benefit of the study. Because when you are going to delivery, they don’t tell you that when you push the same time the baby will come out, we are told the truth.

Participant 4:	Everything said here the person will be told in advance if he agrees to everything after knowing about it there will be no problem, but not that doing it and then he goes it will be difficult but must start enlighting everything else.

Participant:	Everything you mean has to be told that we will do this, at this point we will collect body tissue.

Participant 1:	Has to accept when joining he is knowing what he has signed for.

Participant:	That am remaining with one option.

Participant 1:	When calling them he is aware.

Participant:	When collecting the tissue, they will give anaethersia to kill pain the same way as they do ceaser

Interviewer 1:	Alright, where do you think BCG can be injected.

Participant 2:	Do you have to inject BCG on the fresh, for children you inject.

Interviewer 2:	Yes, we inject here because there is more fresh.

Participant 2:	Better you inject on the butt that when you remove the tissue how will one sit laughing.

Participant:	Better on the arm.

Participant 2:	I wonger how they will sit, the arm when you collect the tissue it will be atleast exposed it will not delay.

Interviewer:	Injection site we have said the same arm, that side you wanted to say something

Participant:	You asked where to inject.

Interviewer 1:	Was asking about site to inject, where to inject.

Participant:	Understood

Interviewer 1:	About what happens in the study we have discussed when they are participating they will collect body tissue to test, are there any concerns related to taking this tissue body the purpose is to conduct other tests to check if BCG is present and they will collect other samples that will go together in the study checking presence of BCG, there are other things you would like to add

Participant:	No, it’s ok

Interviewer 1:	There are different ways of introducing BCG in the body, one of the ways is introducing BCG in the lungs. The method uses a thin tube that passes the nose or mouth through the throat down reaching the lungs. The method is used to collect samples from the lungs what are your views on this type of procedure.

Participant 2:	I think the method is more complicated than just giving BCG because there are other things that can make people not to participate in the study. Like for me I have not been tested for ulcers, but people say when the pipe is going it irritates. So, when too many things are involved yet the injection you just inject people can came easily the other way is difficult.

Participant:	If this way is there, it should not be included laughing, to insert pipe

Interviewer 1:	Chabwino maganizo ena ndiotani panjira imeneyi yoikira BCG mapapo maganizo anu owonjezera enafe tikutipo bwanji.

Participant 1:	I feel like this method can scare people because he will think of taking a pipe and put it.

Interviewer 1:	The pipe is small.

Participant:	Will the pipe not miss it doesn’t miss and scratch elsewhere that can give a person many questions but will I come back alive it can give questions and that can scare yet just doing it here is simple he will know I have been injected that’s all they collect what they want but the pipe a person will be undecided am sure or even if you can put these options foe people to choose, the option people can choose is injection but the pipe one can be difficult.

Participant 3:	Just to give a testimony saying the pipe one is difficult considering time of Covid they were saying we insert something most people were not accepting, I don’t know how people were tested but just receiving vaccinepeople were receiving, but the pipe people were refusing.

Interviewer 1:	Alright I want to hear your views in terms of someone or your relative or a friend tells you that he is participating in a study or is keen to participate in a study like this how can you feel.

Participant 7:	I would welcome it, I can encourage him to take part

Interviewer 1:	Why

Participant 7:	It’s good because let’s take example like he did not know anything about his body, at the beginning before they do anythingthey will be tested everything that is an advantage for them to know about his health, because at the time of conducting tests if there is any problem that he did not know he will know about it.

Interviewer 1:	Others’ views how can we feel, I have come your child telling you.

Participant 2:	I can encourage him because even here I can think twice or even joining it’s good.

Participant 6:	I can also encourage I will tell him that am also in the group I have take part.

Interviewer 1:	Or before you take part.

Participant 6:	I will tell him what am saying I will not tell a lie, we go together let’s go we sign someone will join

Participant 3:	I will welcome him will explain the benefis of a study like this one, I will explain the benefits that can be there and how the study can help the nation.

Participant 5:	The good thing we are CAG in thr community we are the eyes of Wellcome trust.

Interviewer 1:	Ok, all of you are saying you can encourage him, what concerns can be there that a person has participated in a study like this, what concerns can you have, about encouragement we will encourage them still more concerns that you can have as a person.

Participant 3:	Concerns can be there because it’s not all people who can accept this there will be other people who will say it’s satanic up to taking body tissue, they put infection in the body the person will not refuse they will look like you belong to satanic.

Interviewer 1:	Alright, here the concern is that this is for pneumonia be it a friend or a relative has told us that he is participating in the study concerns that you can have. The concern that am asking about this one who has come on the open that am taking part in TB study at 3A the concern that you can have after encouraging him.

Participant 1:	I have encouraged him still more as a human being my concern will be when he goes there as long as he does not encounter something difficult, like sudden illness sick his eyes will be on me. I will have concerns if something comes up because am the one who encouraged him. Automatically when he encounters something	he will say you were telling me as a good thing see it has affected me my concern can be fear if what they are doing is not successful or is has risks. That will be my main concern just thinking let things go smoothly there should be no problem.

Interviewer 1:	Alright any other concerns

Participant:	Aa that’s all

Interviewer 1:	What are your comments on the discussion that we had today where we have discussed about this type of study. Comments we will start from here.

Participant:	The comment I have is this

Interviewer 1:	Number

Participant 7:	That it is a good idea that before implementing the study you wanted to seek for other people’s views. How can this go to look for other views that this can happen it’s what you have answered?

Interviewer 1:	Alright

Participant 2:	It’s good that you reached to us to have our views on how it can go in a study giving people’s views on how it can go amending other areas before you take it to people, check it and make necessary changes.

Participant 3:	My last comment is that we want to thank you these days medical professionals before starting your work in an area, before anything you come to people in the community explain to them what you intend to do the aim if for people in the area to know what you are doing, and they understand. Because in the past you were just coming to clinics that you want this vaccine most people did not accept that because you first reach out to some people when you do that the message is understood continue doing that.

Participant 1:	I am grateful mainly because of the work Liverpool follow because of these measures it has helped many communities to know about your work unlike at the beginning. Reaching maybe like community engagement, which was not there you are using the community, the community is aware of what is happening people are not surprised because they already know when you go in the community, they know what you are doing. And community leader as other stakeholders who have been called, they are like testifying these people came when they are coming in the community, we know about it it was like this. For this reason, ours is to appreciate for this arrangement that you are following.

Participant 6:	I want to thank you for inviting us to tell us about this study a plea where there was a mistake, or you feel it is not good you adjust.

Interviewer 2:	Thank you very much.

Participant 4:	First am thankful to the Lord he is the one who made it possible so that we can meet here, thank you for the lessons today we will take it, we are grateful for what you have taught us, we will keep it in our life up to the time you want to start this may the Lord guide you always.

Interviewer 2:	Thank you very much.

Participant 5:	I’m just thankful that you invited us.

Interviewer 1:	Speak louder because you are far.

Participant 5:	I am grateful too because of your presence we have shared one or two things, when we are in the community when this study is coming, we will not be surprised.

Interviewer 2:	Thank you very much last words is that when we invited you you took it to be present at the discussion, we are grateful thank you very much for coming next time when you are invited come.

Participant 1:	When you will be our guardian, we are ready we will come, thank you.

Participant:	But you lied to us that it will be less an hour, but it has been 2 hours.

Interviewer 2;	We are very sorry.

Participant:	We started a bit late.

Interviewer 2:	We are very sorry.

Participant:	Guys am going

Interviewer 2:	Thank you very much.
