# Supplementary material for: Acceptability of advanced Controlled Human Infection Models: The case of a pneumococcal challenge in people living with HIV and mycobacterial challenge with Bacillus Calmette Guerin in Malawi
Source: PLOS Glob Public Health. 2026 Aug 18;6(8):e0006236. doi: 10.1371/journal.pgph.0006236 (PMC13485124; doi:10.1371/journal.pgph.0006236)
Supplement: Supplementary file 1 [file pgph.0006236.s001.zip › Documents/ACHIMA_CAG MALE FGD_09_27.02.2024_English.docx]

Interviewer:	We welcome you to our discussion this afternoon in the field of research that we re doing that we are talking to people especially in the various groups representing local people in the field of research that the wellcome Trust want to do at this time we want you to watch a short videothat explains the type of research we are talking about, the video is in englist but I will explain it in chichewa

Video play:	It’s a given that vaccines prevent diseases and saves countless lives, but this takes ten years or more to develop new vaccines. We need to find a faster way, one way to speed up the process is by making better use of human infection studies, volunteers are given a sample of vaccine under development followed by a careful monitor infection medical care is always close to hand if volunteers fall ill and if they stay well, it means the vaccine works and can be made available much earlier. The idea is almost 300 years old but using todays advanced technology this type of study is not just scientifically sound but ethically responsible too and of course follows strict rules. Human infection studies show huge promise against fighting diseases like Typhoid and malaria but to develop vaccines most effective at the people at risk we need to test on those at most risk by expanding human infection studies and targeting them better the people most at risk could help develop vaccines of the future and save millions more lives.

Interviwer:	The message in the video is that it is known that it takes a long time to make different vaccines. One way to find a shorter way is to make different vaccines that are used for different pathogens. If you are in our discussion this afternoon on the side of making vaccines related to TB and also on the other side of your opinion on the side of giving them to people who are protected by HIV, the bacteria that causes pneumonia, in a nutshell the message in our video shows us how it depends on the time when this is happening that people they are like watching them and we want to see if they are normal and that shows that the medicine that was given to them or that the vaccine that is being made has worked well but if it is possible that the person is sick then they should give care so that the person can recover depending on the same research unit when we watch the video is there any comment you can give before we start our discussion this afternoon on the message that has been given?

Participant 1:	I understood the message very well and that’s how they talk to us Africans from our white countries that maybe the blood is different depending on where we come from. I think vaccines can be very useful regardless of whether they worked or what happened, but they should really try it for us, the people who have system. For the one who has planned for us to find vaccines that can protect us from these diseases.

Interviewer:	Any thoughts? Or we can start there? Do we understand?

Participants:	Yah.

Interviewer:	Okay. We welcome you again to our discussion section this afternoon. So, I just to know what information you have gathered from the message that I have explained that you have watched, have you picked a message about what.

Participant 5:	I have received a message saying that there is a need for research and the research will use bacteria to give them to people who are HIV positive and those who have tuberculosis and the aim is that they can be able to find vaccine that works faster or find another vaccine that takes a little time to support what we have said we saw in the video that according to the change of seasons that we ar in we need to find another vaccine that they use when the season passes.

Interviewer:	Well, thank you very much. I would like to add that on the TB side, we are giving people who are healthy that they are not sick, they are normal, and we are giving them this BCJ which stands for Tuberculosis. What is the other message that we have gathered from the message that we have explained this afternoon?

Participant 4:	This study wants to investigate the ways of the other body’s defenses for people who have HIV and tuberculosis however, I am a little worried for those with TB and any other healthy person who is HIV-positive maybe he might be on another treatment so his immunity happens that it’s low, it’s made to boost with ARV, so my concern is this part of people with HIV, maybe these bacteria, their immune system won’t cause them to get TB disease again?

Interviewer:	Well, their opinion, the rest of us our opinion? We are looking at our discussions from all angles, we say positive, we say negative, talk to all sides when it is necessary to decide, show us that this is the way it is, that is the goal of today’s meeting.

Participant 2:	I have heard how you have explained the people who are taking part in TB and the HIV virus and then the people who are taking part in the TB side, especially the people who say they are healthy, so my concern is that you will get bacteria and spread it for that person who says they don’t have TB, they are not sick. How do you know that this is the disease? Those bacteria will work well that his immune system will be good I don’t know how you will work with them when you take the bacteria and give it to them. Number two, you will give these bacteria to the person who is HIV positive. How is it compatible since a person who is HIV positive, the HIV positive virus is caused by a bacterial virus in addition to a virus.

Interviewer:	Okay so, we are talking about the message that we have gathered from the information that we have been given for today is there any other message we can add that we have gathered

Participant 1:	Understand what you said that the research is being done in two stages, one is being done on people who are healthy, the other is being done on people who are HIV positive, the research is being done on those who are healthy, with the research which is giving out BCG bacteria to healthy people to see how TB can work for healthy people when the are positive are giving for pneumonia bacteria? Maybe I should say that.

Interviewer:	What message have we heard? Or should we continue with the message that we have all heard and explained?

Participant 2:	Then I just want to add or perhaps ask a question, they say that a person who is HIV positive is sometimes say that they may also be suffering from TB, how will you recognize those people? Because person with HIV is found to have TB so there is probably most three quarters.

Participant 1:	I want to argue with them when they say that for people who are very positive about pneumonia, when health people are looking at the issue related to TB, they should give bacteria to these people in order to get results for sure.

Participant 2:	There are two, right?

Participant 1:	Yes, for the healthy people, they will take the bacteria from TB, while for those of us who are positive, we will take the bacteria from pneumonia.

Participants:	Laughing

Participant 1:	Absolutely

Interviewer:	Well then, we can continue right?

Participants:	Yes

Interviewer:	Which message do you think is the most important of the message that has been explained to you?

Participant 1:	The most important message that I have heard is the message of BCG, the bacteria that we are giving to people who are healthy, will reduce the effects of the spread of TB in this country.

Participant 4:	Another very important message that I have heard is to say that the only way to give this virus is probably more summary to get results faster than other ways of taking it and they explain the change in climate but the difference in areas is a way that I really felt was important.

Participant 3:	Real research from what I have heard can take a long time, up to ten years this method, perhaps as my friend have said, it is seen as a summaryand beneficial to many people in future.

Interviewer:	Good, good.

Participant 5:	I think it is a very important message to say that this research will help find a vaccine for pneumonia. When we need it most, we should have vaccine against pneumonia. We will have dealt with this disease of pneumonia.

Interviewer:	Okay. I just want to know what the additional message is that people should be told before they make the decision to participate in this kind of research.

Participant 3:	My thoughts were that maybe there would be a way or another language that we could use to replace the idea that we might be injecting bacteria into the human body. Cause the understanding is quite difficult so I was thinking that if we can replace this presentation of putting the bacteria that causes pneumonia, TB. So that at least threatened that this coming is something that can cause us problems.

Interviewer:	So, we need to enlighten each other to say that we can give this message, how can I phrase it, how can we get rid of putting of bacteria in the body, what is a good language that can be used as a light to explain to people

Participant 5:	In agreement with the three who have finished speaking, we have not reached the point of reframing by rephrasing but for a person who says he is normal, when you reach the ground, we will give you the TB virus as a test, but we will also remind you that it requires some courage from the person you are approaching. Of which you reach is found to get to them directly that way is that you can’t find many people who can maybe only one says but its to slow down those who can agree the best way we can maybe is to approach someone and tell him that we want to see your security system we are wabut we want to measure your security code to see how it can attack the war or whether it can be solved when I attack it, how can I think like that, maybe because we are paying for the group

Participant 1:	I will argue with them saying that there should be some hidden language because you are a participant, the rights defenders should know that before they do the research because they will see if there was something there were some benefits if the organization finds it, we hide the purpose, they will only get results in the person. This person may also have the right to claim that people lied to me in the beginning, but the issue of research is not unusual. So, let’s not hide it when we say the truth that we want to make it but there should be good policies to explain to the person the risk that a person may face, just to explain to him that he will be under constant supervision by the medical staff and that he will be helped from time to time for sure.

Participant 2:	I also agree with the point number 1, at first, we agree with them, but there is a problem because if the client is found to have a problem, he can file a complaint but should be told in justice.

Interviewer:	Alright.

Participant 4:	I think that all these messages are true that a person somewhere can be threatened that we want to put virus in your body, and it is a scary thing that the person should be explained is that it is clear to him that he will take part or not take part because it is his right to agree or refuse yes but after being told justice

Interviewer:	Alright

Participant:	In addition to this, there needs to be a sufficient ransom so that we can encourage that person to join.

Participant 5:	The only way to agree is to say that if you find a person, when you find that person, we want to do something that is fair, as our number four said, whether it is one yes if you explain to him that we are prepared in this way, then you will be like you are contemplating on whatever you are offering. I will do this, I am sure my benefit is this, but for you to come to me in general, we want to put some kind of virus. So, if there’s something that is like other okay, we will do that but by the end we will do that or maybe not before that we will do that we will do it in this way again. When a person knows that this is the case, he tells the people who live with him, whether it is his parents or his husband.

Participant 3:	I strongly disagree with five regarding the ransom, the participant should not be enticed by the incentive and the way they are talking about the ransom should be explained and they should make a personal decision that they are joining after realizing the benefits of what they are doing not thinking about how they will change the issue of the money or any other support.

Interviewer:	Good, when we do research, when we explain to people, we show them all the information about the research process, the details, when we explain it to them, we give them time to decide. When they make that decision, they are told the benefits, the risks or the negatives that may exist during the participation. How will these be resolved? On the side of threats that may exist that if the part of the research will be resolved, when it is explained they are given a chance to decide whether they want to do a consultation and make it that way.

Patricipants:	Yes

Interviewer:	Yeah

Participant:	I ust want to ask if those people have been diagnosed with the bacteria and you have been diagnosed with a problem, what is your plan? These are the problems that can be found with them.

Interviewer:	Okay, your question is another part of the conversation that we want to hear your opinion in the future. What should they do in the research field? Or how will they do it? I will return it to your side so that you can give us your opinion and suggestion as to how you see it when there is such and such a system so that it will help us because here, we want people’s opinion.

Participant 1:	The way you explained that there should be a doctor or a nurse to explain some aspects of this research, so I don’t know where to take these problems? Either the disease itself continues or it is death. A death has occured

Interviewer:	Let’s look at all sides to see if the person is sick or if it has continued to happen. What kind of plan will there be? Let’s hear from here.

Participant 6:	My opinion is that at the same time of the investigation you believe that we should say that it has started.

Interviewer: We have not started talking to the people to hear opinions

Participant 6:	What is needed is to say that at the time we are starting our research before it is misleading to say that we as members (16:53 unclear) are being educated, we are being informed about this research and we are also giving our opinion and that is that the chiefs should also be educated especially in the areas where this research is being carried out. Everyone should know for sure that we are in queens, here at the college of medicine where it is being taken, there is a research centre. It ust happens but it doesn’t happen, there are some things that don’t go well, they do it sometimes, but it doesn’t happen, but this research doesn’t happen.

Participant 3:	In my opinion, I have heard that during this research period, this person has been monitored to show that the monitoring is always right whether in difficult times or good times, the one who is in charge of the research is responsible for being careful, I feel like that.

Participant 1:	The biggest issue is that you are sure that a person will be taken care of by the medical staff from time to time when they have a problem, they will be taken care of, but there are other things that are technical, like the officeor the owner of the research, they should not disclose it to public because of over things, for example if a person dies, we will give three million examples. Three million is the person who takes it and shows it to my family. Maybe there will be other things and maybe it will leave you at risk depending on the village where we are coming from. I think the biggest thing is that a person will be monitored all the time they ask you to investigate. What is that enough but to say that there will be compensation when this happens? I think this would be technical and you cannot see that if has happened you can see that has happened. I think you can see that.

Interviewer:	Okay, well, that’s the story in the same matter that you brought out, you said that it should be a technical one, on the compensation side because here we are in a sticky situation on your side. How do you see it happening? On the compensation side that can be given, do you think that these compensations can be given? How will it go? Let’s take it from a person who has ust been found to be sick, or from a person who has just been found to be dead, perhaps following the investigation of the house we have met. Just give us some light

Participants:	Laughing.

Participant 6:	Especialy if he is a man, his wife and children should be taken care of as a monetary apology, whether its about the box or even if they pay for school. it is a good thing that maybe he was taking care of his children its necessary to take part by taking care of his children in terms of education and other good things.

Interviewer:	Well, those are the thoughts of the rest of us.

Participant 2:	As we said that you will take this persons part and if this person is found, you will take care of him in any problem, especially the part of the disease until it reaches a certain point he will die unexpectedly that means you will follow on anything important till then as he said if he had kids he was helping they are suppose to take part

Inerviewer:	Well thanks, we can move on here, right? Well, let’s come to our next section where we will talk about the benefits or the brnrfits and skills that may exist in doing research on pneumococci that have been infected with HIV?

Participant 1:	There is a great benefit because pneumonia is a very dangerous disease, so for our friends who are positive and find a vaccine that will help them against this disease, we will make a big difference because our friends are very vulnerable when it comes to pneumonia.

Interviewer:	Another benefit that may exist, lets look at it from the individual’s side, from the community’s side, from the country’s side, lets look it from both sides. That the profit that can be made

Participant 5:	I once said that we will have a great benefit, we will be sure if we vaccinate someone in our health life, it will be the first time to explore the pneumonia vaccine that will help those who are positive, not only for those who are doing this assessment, but it could be for everyone in the future. Would be a great advantage for us as a country.

Interviewer:	Are there ant additional benefits?

Participant:	Another benefit is that when this vaccine is made, it has been approved, it has worked, so we will help our committee, maybe we will help other countries in Africa so that those who have problems like this, so that the world will be able to have forex

Interviewer:	Okay, fine

Participant 2:	For a person who is HIV positive, I don’t see the benefit of this vaccine, but the benefit of this vaccine can be for the person who doesn’t have this disease, it is a special benefit that can exist safely in the community and in the world, we will change a lot, but for a person who is HIV positive, I do not see the real benefit.

Interviewer:	Well, the other point of view is that here we must look at all the sides and let everyone say what they think. It will help us to have thoughts that have deep wisdom so that when the time comes to do this research, it will help things go smoothly thinking of how all points were given.

Participant 5:	I would like to add five as I am not sure if the competition of this research will be written as a report stating the names of those who will do it, okay if it is so its okay, because I thought that maybe there will be compiling a record that has names of people that those people who helped to find this vaccine and these names will be like future history but since the names are not mentioned its okay, I was off-side

Interviewer: Okay.

Participant 4:	I also agree with the opinion of number five, saying that from many researchs that are being done on participants, I think that in this research, if a person reaches the point where he has decided to participate, then we can say that he is a hero because he has solved the problems of the world. So, the person who makes the plan whether in office if he has planned well, as a participant can benefit from something.

Interviewer:	Okay

Participant 2:	To add number four, with the way he has explained I think that this research it will be difficult for us because of the big risk it has. Typhoid was very difficult for people to accept him well, so better that one that this one.

Interviewer:	Since you have said that there is work, to make this easier what do you think should happen on the plan of preparing of starting this research

Participant 2:	Its plan is to call the leaders of the churches as we did about Typhoid, we called of the churches markets we were taking two and sitting at one place and talking to them and explaining because they had most difficult questions.

Interviewer:	Where do you meet?

Participant 3:	I think in your first explanation, you explained that you have been meeting with different various of groups, we are not the first, the best way is the one you have already started after continuing to inform people, research on how to inform people, I think it’s a good thing when reaching everyone on the ground the danger will be less because many people will be waiting to be reached and explained to so what my friend number two is saying is that you should reachout to all churches and stay together I think its something you have already started that groups should be reached out

Participant 1:	The way they explained this research may seem difficult but the issue is the approach, the beginning that you have met with the churches and met this various others, you have met us as CAG but we want to increase in other parts because I have seen some parts before the research, whether the messages you have shown us here in the community they will see for themselves. In other ways, only those people will be able to hear from you. Maybe it will help that all of us together inform the community what is happening and that others are talking about it and that we are there to guide them that it is like this it is like you join the research based on the fact that we know the benefits of the research we will go ineven if they say it is gangerous but only after some who can join I am writing names here, we will participate in this research because we know its benefits. There are chances that the investigation will be successful.

Participant :	In the worst case is taking the bacteria and putting it in the body that’s the worst case and there would have ben another language to cover it up though I would have said people should come straight and I should tell them the truth but this is where its dangerous maybe there would have been another way to say so and according to the words that say so and give them as if we are testing their safety

Interviewer:	Okay, okay, Let’s come to the dangers that can exist when a person with HIV is given pneumococci bacteria and see what dangers can be found

Participant 5:	I think that the dangers of a person who is already affected by HIV are that you are giving him another burden because he says that his immunity is weak and boosted by the ARVs that he is receiving inorder for him to fight against HIV, his protection is also posted, so it already has pneumococci so if we are giving him a big burden, some people may not be strong, may weaken quickly or leave us because we are like adding another wound to them

Interviewer: Okay

Participant 2:	At the beginning I said that the person that I don’t see the benefit of it because of what he said, the person is already sick, he has said the person is already paid for treatment so we should give him something else I feel like we are not doing well.

Interviewer:	Okay, fine, Let’s come to the part of giving the BCG to someone who is healthy and has no other health problem, we have given the person BCG that we are talking about, do you think there is going to be any danger, lets start with the benefit that the healthy person will have after being given BCG of TB

Participant 5:	I think that the benefit is difficult to find because the person does not have a problem we are the ones who are looking for a vaccine so that we can formulate the benefit that he can get in general, maybe that can be found will help others in a special way from him, I don’t think that there is any real advantage that we can do

Interviewer:	Well, some thoughts on the benefits of doing this research on people who are healthy. BCGs research do you think will there be any problem this one has given us idea that he thinks there can be a great benefit what are our contributions.

Participant 1:	I had a little question because I don’t know what methods you can use to find out that these people are healthy depending on where they live, there are people who live near factories. They are living in places where they can get TB at any time so I wish I knew what procedures you should follow to decide if this person is healthy or just tested?

Interviewer:	Okay, fine the best part of wanting to see a person who is healthy is that he is a person who is being testd to see if he is free of any other problems or diseases.if the person shows that he doesn’t have any disease at that particular time and has gone to take part in the research, this person can be asked to take part in the research but it will depend on the fact that the owner volunteered himself because hes curious to do so then the next section if there is anything to see if this person is healthy that will be done based on the tests that will be done on him in order to see when they make a screen, if they make a screen the person seems to be healthy so before this person enters they should be tested for various things to make sure that there is a guarantee that the person is a person who is in the group of healthy people

Participant 1:	Theres a great benefit to the community because when the results come it will help to reduce the spread of tuberculosis which is a very dangerous disease that affects people with HIV.

Interviewer:	Okay

Participant 1:	I want that the other benefit is that although there is TB vaccine, people are getting sickand dying so if they test someone who is healthy there will be a result that will be found it will be acceptable result, and it can be used perhaps on the side of what the platesays that death can be reduced.

Participant 2:	I don’t think there is any benefit in taking a vaccine to give it to someone who says he is healthy so that you can know how it works, I think theres no benefit because its hard to know if it has worked because the person is normal so youre giving that person something else unless if it was that the person is affected.

Interviewer:	Okay

Participant 3:	The benefit is there and it is great, I will give an example in my body just eating something that is contrary to what I like to eat creates the same reaction taking this vaccine and putting it in someone who is healthy will take a long time to show that the vaccine is working health is the benefit, it is possible that it may shorten the time to get the real results of this research, I am not sure if it is clear, lets discuss it maybe we will listen carefully

Interviewer:	I asked what you think would be the benefit of doing researchon pneumococci and I asked about the BCG study that is given in healthy people and what is the benefit of TB bacteria that is given in healthy people

Participant:	In this case, the benefit can only exist if the person is healthy, so the person is infected with the virus, when the person is injected with the virus that means he or she will start suffering then he can show signs. He has been given the vaccine to protect against germs and the germs have started to work if it is found that he has recovered in time, it will be found that many people will have trust. I believe that this vaccine has been made to prove that it is suitable it can help people that’s what I think but if the result is different there is no benefit that means the chances are 50/50.

Participant 5:	Maybe you are confused because what they are being given is not vaccine BCG its not vaccine, they are germs that its result should formulate vaccine.

Participant:	So, what if they put germs on the healthy person?

Participant:	So, if they get sick with the BCG virus or if they don’t react in any way, it will help the vaccine, the results will be found quickly

Participant 5:	Let’s prepare the vaccine in this way according to the information that can be obtained from that person, so let’s assume that the germs are not injected, then the research is ongoing, and the monitor is made regularly I don’t know want or what can happen to the person at the end.

Interviewer:	No, what we are looking at is not the benefit that goes to the individual, but we are looking at the benefit of this vaccine to see if there will be a benefit or not.

Participant:	There is a benefit and a vey big one that is helping that in Malawi as our country should find vaccine or find a solution.

Participant: The benefit is there and is the great great benefit, this will help Malawi as a country to find a solution to TB as a disease.

Participant: Number six wants to understand more since he has just joined us, he wants to understand what will happen at the end when the body starts reacting yes, we have given administered germs to the participants and the research is being monitored, what will happen next?

Interviewer: Okay, as a part of the research, when a participant’s body is reacting because of what he or she has been given by the research team, the participant will be attended on until he or she gets better.

Participant:	Participant will be helped and protected by the research team.

Interviewer: Yes, as part of care the participant will be helped by our research team, and when we talk about the benefits of the study we are not just talking about individual benefits, you should also consider general benefits.

Participant:	Which means those who are involved in the study and those who are not involved in the study.

Interviewer:	The benefits will be for both the participants and those who are not participating in the study.

Participant: Yes, there will be benefits.

Interviewer:	Okay, in terms of challenges, what challenges do you think will be there because of this type of study?

Participant:	The challenges that will be there is that some people might join the study and withdraw from the study while the study is still going on.

Interviewer:	People might join and not be there Up to the end of the study.

Participant 1:	Why are you saying so?

Participant:	One, when we take someone who is not sick, a person who is not sick do not take things seriously, so they might start the study and not be able to finish it.

Interviewer:	Okay fine, a challenge?

Participant 2:	The challenge that I see is that the participant might get a problem because of what they have been given, maybe the participant was feeling well and started getting sick. This might make him withdraw from the study.

Interviewer:	Can you give examples of the challenges they may encounter for participating in this study?

Participant: They might get sick.

Interviewer:	What type of disease to be specific or just any sickness?

Participant:	I cannot foretell the type of the disease.

Participant:	You are just scared?

Participants:	Laughing

Participant:	Maybe TB

Participant 3:	The challenge that I see will be from other religious people, they will take it as a chance to raise against the idea of putting germs in people’s bodies.

Interviewer:	Okay, that is a good idea.

Participant 3:	Sure, sure.

Interviewer: We should continue looking into the benefits that might come because of doing this study. What can you say in support of this study?

Participant 3:	I think there are more benefits than challenges so it will be a great thing to have this study happening.

Interviewer:	Okay, fine, others?

Participant 5: Just to agree with number three that the benefits are high, considering the moments we are going through and the technology that we have. All the stakeholders should join hands in encouraging other people to join the study. The benefits are high.

Interviewer:	Okay.

Participant 1:	For a long time, we have had studies that developed from another countries like Kenya. Now things have changed so Malawi as a country should take this as a good thing because the results from this study will be of benefit to our country.

Interviewer: Let us move forward, some studies have occurred using pneumococci or we should say the bacteria that causes pneumonia in the bodies of health people, how can you differentiate those studies from this study which is using people living with HIV as subjects?

Participant:	Can you come again?

Interviewer:	Some studies in Malawi have used pneumococci before or we should say bacteria that causes pneumonia in people that are health how can you differentiate those studies from this study that is using persons with HIV as participants?

Participant 1:	The study that used health people is different from the one that use someone who is sick, because the other one is well, and the other one has a virus. So, when we talk about HIV, we are not talking about those are sick but those with the virus that might cause a lot of diseases, so giving them the vaccine will help to understand the way their bodies can be treated.

Interviewer:	Okay, is there another comparison that can be given when doing research on a healthy body and the other one which is infected by HIV?

Participant:	I do not really see the difference because the one with HIV is already on ART which means their body is functioning well and the same with the other person who is healthy, that means there cannot be a significant difference.

Interviewer:	Okay, fine let us talk about the challenges how can we present these challenges to the people so that they should not be scared and should turn the participants away from participating in the study.

Participant:	Can you come again to the question?

Interviewer:	How can we present these challenges to the people so that they should not be scared and should turn the participants away from participating in the study.

Participant 5:	The challenges will be there yes, but we need to let the participants know that even though the challenges will be there, but we will be there for them throughout the study, this will help them understand that they are in good hands. Especially this one with HIV and AIDS we need to tell him that after giving him pneumococci they will be fighting two things but has to be told that we will be there for them monitoring their health, this will assure the participants that you will be there for them, unlike letting them be at home and should be visiting the hospital from home.

Participant 1:	When we talk about care it should not just be for the participant but rather for all their family, for instance if you give the participant BCG that means the participant will be coughing out and that puts the people around them in danger. So, this care must be extended to the who family because they sleep in the same roof.

Participant 5:	Yes, they eat and stay together.

Participant 1:	Yes, if its me that means my wife as well.

Participants**:**	Yes, the same.

Participant:	I think that idea is great.

Interviewer:	Okay, let’s continue. I want us to discuss about who you think can participate in this study between men and women.

Participant 5:	I think both sides can participate, we do not have to generalize that we should only have women or men. Let us make it open to everyone so people could accept depending on how we are presenting it.

Interviewer:	Okay, so it must be for everyone right?

Participant 1: The idea is the same as my colleague, but I just have something to add. I think we should not generalize it or make it open for everyone that will make us have a lot of people participating in the study. We should instead have a figure of people we want to have in this study which will help us not to have more women than men since women are always interested in issues that are to do with research and women often visit the hospitals. So, if they can have an actual number of people they want to be involved in the study, that will help to have good results that will benefit both men and women. Apart from that if there will be a time when someone will want to find out how the BCG worked on men and women this study will be of help to them.

Participant: When it comes to health issues, women are always willing to take part, but men are often reluctant to be involved in health matters. So, we should not focus on one part as my friend suggested, it should be open to everyone who shows interest.

Interviewer:	Okay, fine.

Participant 2:	I am agreeing to what my friends are suggesting, you should have an exact number of participants to be involved, but I could love if you can involve more men than women since men are the one who are more prone to TB.

Participant 6: I just want to comment on the age limit, you have indicated that those who have reached 18 years of age can participate in the study this shows that you are an expert in this because I could just guess about the age without being specific which would make things not any better.

Interviewer:	That means we are going to include both men and women in this study. I just wanted to know if there will be any difference in involving both men and women in this study.

Participant 2:	We should consider having more men because men are close to suffer from this disease, because a good number of men do smoke, and some men work in factories so they can easily have this disease.

Interviewer:	So, about the study that I was talking about that they used the bacteria. It showed that men were the ones who showed more interest in participating than women, what do you think could be the reason to that?

Participant 6:	I think women the reason why women are given more information about health is because they often meet with HSA’s, so they are trained about health issues and other various vaccines, so this makes them easier to follow health matters.

Participant:	You should know that men were the ones who were more than women.

Interviewer: Men were more than women, let him finish and see how he will conclude.

Participant: But men have not been active in such matters for the past years it was only women being ahead of us, I think men have now started realizing the importance of this.

Participant 4:	It is possible that the channel which was used to disseminate the information was more comprehensive to men than women.

Interviewer:	Okay, other ideas?

Participant 1: Women usually take part in studies that are involving them or children, but looking at this study is for men.

Participants:	Laughing

Interviewer:	So, you are saying it is for men?

Participant:	This one is for the ones who know what they are doing, you can not just join this study for the sake of joining, this one is for the die harders.

Interviewer:	Why do you say this one need strong hearted individuals?

Participant:	We are saying so because we are talking about someone who is okay and is going to be given germs that might lead to diseases so it is not easy it needs men who do not usually think about what they will leave behind when they die, but for women they usually think about their children. So, men are courageous enough.

Participant 1:	Yes, very, this needs decision makers, women cannot just join any study without letting their husband know. They might say my husband I want to join this, so when the husband thinks about his wifey getting the germs, he might stop her considering that she is the one who takes care of the family.

Interviewer:	Game might be difficult okay so on the same research we should now talk about the one that is coming in future which we are saying of BCG, there is hope that women who are on reproductive age who also want to joim in the research they have to follow methods of birth control for the mean time they are in this research, what are your opinions about this issue of women of reproductive age to follow mwthods of birth control in time of doing research

Participant 1:	It is very necessary because we are saying this research will take bacteria inserting them in the body so if it happens that they are pregnant in the process it will be complicated so the problems of getting sick frequently will arise so they are suppose to be given because maybe they might get pregnant in the process of research which it can affect what is expected not the owner expecting

Participant 4:	The way number one has explained, when a woman is pregnant a lot happens for example, she has said she has BP she will not be part of the research but when the woman is pregnant a lot of women happen to have BP which they are not qualifying to join the study.

Interviewer:	Alright

Participant 3:	Just a thought sometimes the method of birth control can be distracted finding that a person took the method of birth control by accident is found I believe if its like that maybe the one taking part can withdraw to take part I just wanted to know if she is not part of the research anymore because of what is happening that does not happen frequently but its possible to happen that means they will be still looked after for or it is going to end there

Interviewer:	I cant answer your question at the end because that thought that is there is to make sure that onlt those people that want to take part in the section of the research should not be found at some point that a woman is pregnant because which is a big reason so for that mean time that she is taking part in the section of research there shouldn’t be any woman who is suddenly pregnant for other reasons some of the reason is that you have already mentioned that according to how she lives when pregnant and to to be given bacterias it can be somehow difficult to manage it so the thought that is there of examining well that she is not pregnant also for the time she is taking part in the research should show that she is not pregnant if it happens then it will be that it has happen accidentally I believe in the research you will find that maybe it can happen since we are only human

Participant:	Sorry we are together, but we are left behind somewhere so let me ask from at beginning of insterting the germs from there maybe I can be able to follow

Participant:	My question is that what is the main reason of taking germs and inserting them in a person’s body who is not affected with HIV or affected with TB whats the main reason in this research I wanted to know how it would be in this research I can read im a bit behind

Interviewer:	When I was starting I explained the reason of this research is to search some other ways that can be used to help the search some other vaccines that can be used in future to help in terms of other diseases so the bacteria that is put in peoples body to see the defense of their immune in the body how is it going to deal with these virus with the aim of the results that are going to come out that is what can give an idea of making a vaccine or not

Participant:	Okay, that’s where it is explained that there will be a person with the help of those working in hospital meaning that after the person has bacterias in the body if it happens to be any problem they can help us

Interviewer:	Yes

Participant 1:	Okay we should go back I think this question you have answered it in the issue that we said it should be a must that every woman who is taking part in this research should take the methods of birth control and also for all those women in reproductive age should be taking the methods of birth control I wanted to go back to that you have to show properly about the other method of birth control that a person should be following time and time while at home like pills happens that some women stops taking them in order I don’t know what ways are you going to use to make sure that they have really taken a proper trusted way of method of birth control

Interviewer:	Can i answer your question at the very end? Because right now is with time it will be like we are against it. We wanted to reach to the other part that we wanted to discuss things that will happens in this part of research that we are making of TB which is of BCG as one way of this research the one taking part is going to be injected Jackson of BCG as a way of giving out bacteria BCG this is the same as the one that children receive in Malawi on the plan of vaccine but this will be given on different measures this vaccine will be given out on different measures like taking the fresh where the injection has been injected to test if BCG is still there also to test how the defence of the immune in the body to BCG is like. How do you hear about this BCG injection

Participant 5:	In my hearing im heating that where the vaccine is put bacause it has been accepted you will have been to already have research on the people that they have now been given consent that i will also take part in the research so BCG is coming is inected in me that I have already gave the consent I would feel good because the process of research is starting from my body

Interviewer:	Okay, fine, other opinions

Participant 2: Right there there is no other problem because it’s the same things that you have already discussed so it is going to start.

Interviewer:	Fine

Participant 1:	Did I hear, or I didn’t hear well fine ive heard that you will be taking the fresh

Participants:	Laughing.

Interviewer:	On the place where they have injected to test that BCG is there on the place that is injected

Participant 5:	After somedays eti?

Participant 5:	After somedays, right?

Participant 1:	There I have heard I just wanted to ask here, when you say you will be taking the fresh on the place injected that means it will be in the body?

Interviewer:	Here we want to start looking at its place where it has been injected that that place is the BCG going to be found? But on the testing, they will take a small fresh on the place where they have injected. Also, some other test in the body that they are going to test to see if BCG is still in the body, then there are two types of tests, on the place of injection which is sample of body tissue and testing blood which they are going to test to see if there is BCG

Participant 5:	If it is not going to be found that means it is defeated right its not strong anymore for someone who has strong immune system which has defeated BCG.

Interviewer:	They want to see whether it is found or not to know how ist is working and on these steps, what are your opinions of taking the fresh

Participant: It is well, but my thoughts with the way I have heard is very small but to mention it that a fresh is sounding big, the thing is that we are going to test where we have injected that’s okay, so telling them that the fresh

Participant:	They should know yes not that it is going to be a big piece no it is ust going to be same part of testing like becterias of BCG are found

Participant 2:	Is there no other way apart from the fresh to find out if there are bacteria or not.

Interviewer:	On of the way of doing this is through this way the other one for now I should stop here because there are uestions coming so if I go there, it will be that I have entered another section so we should finish this first then do the sample taking on the injected place

Participant:	We have heard

Interviewer:	You have heard it is accepted

Participant:	It is accepted but it will depend how it is going to grow.

interviewer:	It is not big it can’t even be seen or putting it on the figure it ust looks like

Participant:	The language of testing not taking fresh so maybe you are also injecting to check it’s the same as taking the sample like of blood you take a few but the way it presents itself to others its like litres that you have benefited in a certain way it’s the same here to just say fresh it will be a big deal

Participant: We will also test where we injected

Interviewer:	Meaning that you are saying that we should just be saying where is tested not where it is injected

Participant:	Not fresh if you say that you will increase the risk to those taking part that my body is being used and they are also taking the fresh

Interviewer:	Fine so you have already answered the question that was coming that you have given some blood so how you can present to others on the part instead of taking fresh

Participant:	We will test to see if there is the BCG where we injected or not on the first place you will come again to cut off if there is or not

Interviewer:	I should ask on which place of injection do you think BCG won’t be hard to inject

Participant 5:	If it can be on the butt.

Participant 1:	Like that people to volunteer themselves in this research if it can be somewhere that won’t be an obstacle where it will not make them overthink otherwise if it can be on the shoulder where we are used to or we say on the arm.

Interviewer:	Fine so the place should be on the arm number five said on the butt

Participant 5:	I wanted it to be on the butt to take the fresh so taking on the butt the fresh one can’t see how you are cutting.

Participants: Laughing.

Interviewer: Fine lets continue

Participant 2:	Before we continue, I think can’t you ask the person whether to inject here on the the butt?

Interviewer:	We want you the first people we are meeting to give the opinion

Participant 2:	Giving the person a choice.

Interviewer:	No that’s why we are saying choice, meeting you we want you to help shed light on some things that the place of injection should be the one that we have heard from different groups

Participant 6:	I think it doesn’t matter where the person is injected.

Interviewer: Number one you wanted.

Participant 1:	I just wanted to give an example that one time I went with a patient to the hospital, after I went to the place where Jackson had written for them, he asked me that there is fuel in the car so we should go back home and prepare first.

Participants:	Laughing.

Interviewer:	So that what you are thinking about?

Participant 1:	This will be a barrier to be on a butt don’t just be seing a lot of people walking im town.

Interviewer:	We should continue there are different ways of giving BCG in the body, one of the ways is to put BCG in the lungs this way is used with a pipe which passes through the nose which is inserted in the nose or passing the mouth, neck to the lungs this way is used to take test or sample of the lungs what are your opinion

Participant 1:	Donors in that are not available.

Interviewer: Let’s explain

Participant 1:	We should be talking about possibility this is not possible because there are many things that we these people have seen when testing covid, how difficult is it for people, this is this but it’s the same.

Interviewer: The injecting one

Participant 2: Just to add a point, this method is not good, better the injection.

Interviewer:	I believe that the answer to your other question has come with thiese methods

Participant 1:	But not this one

Participant 5:	We have already given that person a risk and we have failed to use our words to convince them to come into the system.so we should also tell him that we want to insert this through the nose we will do like this I remember I was put in a cafeter to put in the urinal for the first time when he diagnosed me with stomach problems and I slept with it for the whole night and it was very difficult for me, if of the nose reaches to the lungs we should take the one that inects the normal one but that one you wont find it even if it is found

Interviewer:	Let’s come to our other part I explained at the beginning that if oe part of the research when the patricipants join the research bacteria will be kept or they will be kept or they will be monitored. They should be close especially in the early days when we give them bacteria for testing what are your thoughts on keeping research participants at the research site?

Participant 1:	It is good because it is not that person we are Talking about that we are going to take pathogens and give that person, there needs to be man marking so that that person is monitored from time to time very good things but since there are many years in the family I think that it can be a barrier to say that like a father to say that my wife is not sick but joins the research to get results that will help the world but it is a good thing that’s when a good approach is needed for people to be well, otherwise you will be surprised. People are enetering the survey but not completing it.

Interviewer:	okay

Participant 4:	I think its really good that they will be monitored during these days because they will also have the courage to say that if something happens the doctors and nurses who are nearby will help me but maybe they don’t know where the storage will be whether its at hospital or at the lodge?

Participant 5:	I think that if there is another way for him to be monitored whether he will go frequently he will be given transport to see him in the right place to see him the difference is to admit him there for some time and that would not be good.

Participant 1:	I will argue with them before they go too far because if they are kept, that person will be kept in a good place. There they may not be increasing the number of bacterias that have already been given because people come from different places as I explained at the beginning that we come from places that are very dusty, they will give him the bacteria that cause TB and keep him safe, but if they leave him again there is a risk that he can increase the number of insects because it is not safe place the person we take to pay for treatment we take him because he is sick

Participant:	I believe at first that it is not good to keep him for two or three days. I believe you said that we are treating him as an outpatient.

Participant 5:	As an outpatient I feel that there is a risk because this person is trial and error. So, when we are doing trial and error, it is 50/50. If I give him the BCG, the order should be made so that the closeness should be certain that when we drop him home at night maybe this disease might come again at night so lets assume a place like chavalas in chikwawa lack of transports, motorcycle reaching there with bumpy roads so from there to rush to the hospital one can use motorcycle for three to four days either the doctors will find a way to stay with him sleep here I should see through my equipments and stay with him for three to four days monitoring him just for that matter I think so but if we just throw the bacteria at him he will stay there.

Interviewer:	Let’s give it to each other

Participant 1:	Thoughts on the part of this research saying that if we want to choose the participants, lets sit the hospital maybe on the package of the things that we say to be given, maybe there is a phone saying that you should call at night, three there should definitely be a good response, the doctors should be alert so that he car is ready as soon as they come being in a place that is accessible will be difficult

Participant 3:	Short stay is a good idea and a very good way but when the challenge is there, I find that they will take part because many of us depend on each other to stay in the house moving someone for one or two days is a big challenge on the other side, until today in our culture there are still some who believe that, I as am man cannot cook for myself so the children will be hungry whether you the father, because of feeling oneself you will fail to prepare food because someone else has moved

Participant 2:	I think that there may be a problem and it is a good way, but he should not be like a patient, and we should not take care of him because let’s say you have taken a man who has a responsibility with a guardian that doesn’t mean that he is sick hes not strong well we should leave him home.

Interviewer:	So, before you proceed. I would like to explain in a previous study that people to go to Grace Bandawe where they stay for the first days in these days, they go in the evening only because of stress of which a person has been given a bacteria in his body which is new and thinking that diseases most of the time things can start at night. People are given chance to do their work in each and every day they should go there in the evening with the aim that if something happenone they should be near the place where it wont be difficult to take them to the hospital so my thought was that these people in the first days should be monitored inorder that something that can happen they should be able to take them to the hospital without any obstacle

Participants:	This method is good don’t put another one

Interviewer:	So, let me ask you another question this group has talked about two different people so do you think how many days that these people can be kept in these kinds of places like Grace Bandawe that how many days can they be monitored in the evening

Participant 1:	First of all, we are not told how long your research is taking.

Interviewer:	I said that the research was not done because this is the first stage where we are meeting the stakeholders to talk to you. We can’t say that this was supposed to be like this these methods will come because of these meetings all the opinions that are given are the ones that are going to make the plan of how things will go.

Participant 5:	I think they should be five days.

Interviewer: Five days fine this one has given us his thoughts

Participant 4**:**	I thought if it can be the whole week depending that bodies react differently but on one-week that means everything will be settled.

Interviewer: Fine

Participant 3:	I think its difficult to suggest days because we don’t know what the reaction period will be.

Interviewer:	Fine, fine

Participant 1:	It is important to sit with the doctor since this has reached to the doctors that maybe after some days it will be that the person has shown the symptoms depending on what they are seeing but if it is the way the have suggested one week or five days it is possible to keep them for those days.

Interviewer:	Okay, fine

Participant 4:	I agree with you that you need experts to investigate properly.

Interviewer:	Okay, fine I want to know for sure from the person who we are calling a participant, who is the person who is supposed to give him or her if it is his or her case that if something has happened after the investigation they can call him or her if someone wants him or her but he or she cannot find someone else who you think can reach him or give him or her message related to the research who do you think it could be?

Participant 5**:**	The question says that if I have participated in close contact, she should be my wife, if she is a woman, her husband should be the parents if she has children.

Participant 5:	Because of the closeness its like theres not a day before im togather with my wife maybe in the afternoon we eat together or evening so im going they have called me at Liverpool, they know that im here so after an hour or two I will be going back simple like that like now they know that I have left so calling each other on the phone it will not be difficult

Particant 3:	Privacy to be someone outside or who is present is that the privacy inside will be disrturbed.

Interviewer:	Fine

Participant 3:	Of course,

Participant 2:	I agree with the previous issue but its too near just like he has explained another thing to add there I wanted to take part that if CAG member is not allowed to take part because there can be misunderstandings.

Interviewer:	Fine let’s come to our last topic, I want to know what you would do if your friend or relative tells you that he is going to take part in the research of this type how would you feel

Participant 3:	I can feel better because I realize the benefit of this research.

Interviewer:	Fine anything else you can do?

Participant:	I will encourage him because I know the benefits of this research

Interviewer:	Fine, other thoughts?

Participant 1:	Sometimes is difficult but we as CAG members in the area often, whe n an organization like MLW is found there is a study that says that we as CAG members can join them and make us heard because we will be set good examples in the area most of the time when vaccine come in we only charge for them because most of the time when people talk about research they say that we are only like posters and we usually don’t participate

Interviewer:	Fine, you have talked about encouraging others you are like posters its not supposed to be like you are taking part is there also some things that you can say? Anxiety or what you have after being told.

Participant:	Theres no need to worry because we know that the research is done in accordance with good policies inorder not to find a problem because the purpose of the research is to find the results that will be useful to the world for different diseases

Participant:	As you have agreed with number four, I agree that it is true that there can be no concern because the one who is conducting the research is this large and stable organization MLW

Participant 2:	Right, there is no need to worry if you do research here in Malawi as it has already been done in other countries.

Interviewer:	Fine

Participant:	There is no need to worry because we as CAGs have given good advice on how research can be done to be useful to us

Interviewer:	Thank you lets come to the conclusion that you can give this afternoon about the discussion that we had. What is the summary?

Participant 1:	All these researchs that we have discussed in todays day are good in my opinion I say that please these researchs need to be done to advertise properly but this research I don’t know how to say it in other language but maybe you have made me understand to say that people should have a good approach what people should do if theres an opportunity I will make a poster important in the communitynso that when something comes up people will understand it easily

Participant 4:	I think that this research is for the participants I think that maybe I am asking for incentives of course the part of research we don’t need much but we need to help the world but as a human being I ask if there is going to be special thoughts in part of incentives different from what others think. This one should be a practical incentive.

Interviewer:	Fine

Participant 2:	In my research my conclusion was that I will do a good research and Liverpool has a very good plan my worry is the key to tell the person that the next statement you should plish up should be better and not to be threatening if someones statement softens I see that they will take part, there may be many but if it is like that there will be a shadow

interviewer:	Alright

Participant 2:	The time we were doing things about typhoid it happened that some people were asking that why not doing campaign to make people join this village they will want to inect us things that we don’t know about that’s why im scared.

Interviewer:	Is there also another comments to add,I thank you because of your important and helpfull opinion that we have shared this afternoon in the part of our conversation I don’t take it for granted, im thankinf you for your volunteering and finding your own opinions,your strength and wisdom that is important and it will help to improve good plan to carry out the research that is expected to happen in future, thank you so much this marks the end of our discussion

Participants:	Thank you
