# Supplementary material for: Acceptability of advanced Controlled Human Infection Models: The case of a pneumococcal challenge in people living with HIV and mycobacterial challenge with Bacillus Calmette Guerin in Malawi
Source: PLOS Glob Public Health. 2026 Aug 18;6(8):e0006236. doi: 10.1371/journal.pgph.0006236 (PMC13485124; doi:10.1371/journal.pgph.0006236)
Supplement: Supplementary file 1 [file pgph.0006236.s001.zip › Documents/ACHIMA_COMMUNITY LEADERS_MALE 07_16.02.24 240216_1057.docx]

**NAME:	FGD**

**SEX:	MALE/FEMALE**

**PROFESSION:	COMMUNITY LEADERS**

**LOCATION:	NOT MENTIONED**

**Video:**	Vaccines prevent diseases and save countless lives. But will it take him 10 years or more to develop new vaccines? We need to find a faster way. One way to speed up the process is by making better use of human infection studies. Volunteers are given a sample of a vaccine under development, followed by a carefully monitored infection. Medical care is always close to hand if volunteers fall ill. But if they stay well, it means the vaccine works and can be made available much earlier. The idea is almost 300 years old, but using today's advanced technology, this type of study is not just scientifically sound, but ethically responsible children, and of course, follow strict rules.

Human infection sites show huge promise in the fight against diseases like typhoid and malaria. But to develop vaccines most effective for people at risk of disease, we need to test them on those most at risk. By expanding human infection sites and targeting them better, the people most at risk of diseases could help develop the vaccines of the future and save millions more lives. It's a given that vaccines prevent diseases and save countless lives. But will it take him 10 years or more to develop new vaccines? We need to find a faster way.

One way to speed up the process is by making better use of human infection studies. Volunteers are given a sample of a vaccine under development, followed by a carefully monitored infection. Medical care is always close at hand if volunteers fall ill. But if they stay well, it means the vaccine works and can be made available much earlier. The idea is almost 300 years old. But using today's advanced technology, this type of study is not just scientifically sound but ethically responsible too. And of course, follows strict rules. Human infection states show huge promise in the fight against diseases like typhoid and malaria. But to develop vaccines most effective for people at risk of disease, we need to test them on those most at risk. By expanding human infection states and targeting them better, the people most at risk of diseases could help develop the vaccines of the future and save millions more lives.

**Interviewer:**	in short, this video shows us part of these methods in the trial….(**PI** interrupts)

**PI:**	CHIM studies are types of research that involve the deliberate infection of volunteers… (0:01:59-0:02:03)

**Interviewer:**	alright, welcome to this section of our research study this morning. This research study involves discussions about controlled human infection model in HIV positive individuals. We are the MARVELS team from Malawi-Liverpool-Wellcome program conducting a research study bearing the abbreviation: “CHIM” which stands for Controlled Human Infection Model in full. We are planning to expand our work on pneumococcal challenge, to help reach out people living with HIV. Your views in this study will help us learn the procedures followed in this type of research in future. I would like to learn from you about the information you feel to be very important from what I have explained about this study?

**PM01:**	where are you getting the bacteria you are inoculating into the bodies of Malawians?

**Interviewer:**	as regard to such bacteria (0:03:31.1-0:03:35.4) please say out your number.

**PM01:**	number 1, in my opinion, all that information is important because this will help us reduce the occurrence of diseases. For example the disease that is under study now, oppresses Malawians.

**Interviewer:**	let us proceed with the responses, in this case we have opened the floor for discussions. Which information from what we are planning to disseminate during the progress of the study do you find is important or should be expanded or make some amendments?

**PM03:**	the information we have from you is important. In addition, there is need to have this information disseminated to the public or community awareness meetings, so that the public should be aware because the aim of the information is for an individual to fight for their own wellbeing because no man wishes themselves dead, that is impossible. Individuals must be protected during such activities. It is therefore important for such information to be disseminated to the public.

**Interviewer:**	Alright

**PM02:**	the information we have from this meeting is so important, but we wish to have good ways of approaching people in communities once the project is approved because I remember during the COVID era, so many things were said among people. That is just exactly what will happen in the process of waiting for the vaccine under development. We will ask you to have time and explain this to the public as you go round communities to meet with chiefs to make it possible for such information to reach out to individuals.

**Interviewer:**	what do others say?

**PF04:**	I agree with this information because it has already been stated in the first place that this is not a forcing matter, but rather something that an individual should accept voluntarily, just as I have already said that: in different families, some men are reserved while their wives are naughty, to the point that they sneak from their houses just to attend research meetings without their husbands being aware. I would like to learn from you: how do you follow up this kind of issue, because whenever we are conducting our study, we see people moving around the village, looking for a particular individual who died after a certain study phased out or maybe something went wrong within the progress of the study which hindered them from participating? I would like to know about the measures you have in place to follow up on people who don’t attend study meetings due to lack of interest, deaths or maybe something just went wrong with them. Should we say this is the time when you go to meet with the chief and in the process discovers that a certain woman got involved in a study without her husband being aware? In addition, the issue of transport reimbursement sometimes contaminates the field, in the sense that the woman got involved in the study because of money and decided to walk home just to serve the money. Once the husband sees the money, he asks the wife where she got the money and this results in fights. In the long run, the chief takes this situation as having something to do with this woman cheating on her husband, or it could be that the husband is cheating on the wife, I don’t know what measures you have in place to resolve such cases?

**Interviewer:**	let us keep that question for the last session, because I feel it will fall in the comments section for the same topic. Which information have you got from the discussions that you feel is important and should be shared with someone?

**PM06:**	from this information, I have learnt that we can encourage people in the community to take part in this study, with an aim of making the diseases that oppress our history. Therefore, once we encourage people to participate, it means our lives will be improved. Therefore, I urge the community to participate in this study.

**Interviewer:**	what else have we picked from this information?

**PM04:**	the information I have got here is that everyone should join the study voluntarily, to assist the communities with research findings. When people participate in research, research findings will help other people suffering from the same disease to improve their health. Therefore, chiefs must play a great role to explain to the community since they are the centre of influence for the community. When the chiefs don’t get involved in information dissemination, false information will come out instead. For example the community will say: “the research team should be drawing blood from people without their consent, or maybe they are getting paid for doing whatever they are doing” but when the chiefs understand the whole purpose of conducting the research, they will save us from being perceived wrongly by the community.

**Interviewer:**	alright, what other information do you feel should be included in the information that has been explained about this study?

**PM02:**	I feel the information you have given here is well understood, but we should wait for this program to be approved. Once this is established, we can then think of measures to follow, in so doing: things would work out for us

**Interviewer:**	can you explain the measures we should follow?

**PM02:**	for example, the good measures we can ask you to put in place may include: visiting the group village headmen, just to let us know about your visit in our village on a set day and request us to gather the community members together for you to hold a meeting with them and explain in details for clear understanding and welcome the project, because as chiefs, it is hard to make the community understand the project without allowing you to have it explained in details. As custodians of the project you should give an overall explanation, basing on the skills you have you are in a position to explain this clearly to the community for a clear understanding.

**Interviewer:**	what else can you add on this?

**PM06:**	what I can say here is that: in most cases, issues regarding illnesses are disseminated and decoded in different ways, more especially by people working in media houses (the journalists), therefore, I would request you as an organization and the government to ensure that this information is well disseminated for the public to capture the right information, because once the information is disseminated wrongly, the information decoded will be different from the original one. In so doing, this will make people shun from joining the study. Therefore this information is important because of the fact that some diseases are easily cured when correct measures are put in place and practiced, although some situations at hand makes it difficult to have the disease cured. When the research study is in good progress, this may cause disease occurrence to reduce which may result in the existence of a health community.

**PM04:**	I feel there is need for the community to be given special training because it is difficult for the community to agree with something because there are usually misconceptions and negative news spreading around. For example, people will be speculating news like: these research teams are deliberately injecting bacteria in people’s bodies just to make them sick. Civic education will help the public to understand what is going on, because the public will be told that the inoculation of bacteria in participants’ bodies will be carefully monitored to prevent them from developing adverse events, in so doing misconceptions towards the whole project will be discouraged. How long does it take for the bacteria to begin working once inoculated in the body?

**Interviewer:**	there are different types of bacteria bearing different incubation periods, some possess an incubation period of three days while others have an incubation of five, seven days. Therefore, it is the lab team that understands how the bacteria works once inoculated in the body. As of now we cannot justify the length of the period but it will all depend on when everything will be set, that is when we will tell because everything will be in place then and people will have an idea on how long a procedure will take for a participant to show the outcome of the procedure

**PM01:**	in the first place, you said something like: “this all requires the government to approve this procedure”?

**Interviewer:**	yes

**PM01:**	and now you are saying: we should disseminate all the information to the community, what if we take this information to the village, what responses should we give to the community whenever we are given questions on the matter?

**Interviewer:**	I did not say: you should disseminate that information, but I said: “we would like to learn from the views of you people as community leaders” I would like to know what will happen once this procedure is set, since you are representing the community, therefore your opinions will represent the whole community. I did not say you should go out there to disseminate the information because it is not yet time to disseminate this information.

**PM03:**	I agree with all what has been explained here, more especially on the issue regarding looking for participants, that should be given a clear explanation of the whole procedure because, to be honest on this matter, none of us here can manage to explain the whole project to the community, this is the reason why we would urge one of the research team members to explain the whole project to the community so that if there may be any questions, they will be in a better position to give correct answers. I feel this can be a better way to do it. Additionally, whenever you inform us about your visit, there is need for you to have a vehicle and a megaphone to sensitize the community about your visit on a specified date and place and then we will be there to remind the community about your visit, so that whenever you come and give an overall explanation of the project, we will be there to agree with you but where questions are concerned, you should be the one to answer.

**Interviewer:**	the main issue here is based on the consensus that: before this project commences, the team members should be there to inform the community about the project so that they should get the right information from the staff members for the study. I think that is the main concern not so?

**ALL:**	that’s it.

**Interviewer:**	how else should this project be presented?

**PM03:**	on what basis?

**Interviewer:**	in this case, you have told me that in terms of what should happen, we should approach the community leaders first to inform the community about what is going on. Is there anything you feel should take place aside conducting community meetings?

**PM06:**	the other thing is that: since community meetings are usually difficult to conduct, the other way to do it would be: taking this whole thing from the perspective that: the project is being conducted by a medical team, therefore, an individual should be informed about this in a special way whenever they go to the hospital to seek medical care, in so doing, the information can easily be disseminated. The information taken from the hospital is easily trusted to be a fact since a hospital acts as a centre for true medical information. Therefore, if information can be disseminated this way, most people will agree to participate. Or in other ways, allowing the medical team to explain the information to chiefs, would allow the information to be disseminated easily because the chiefs are few in number compared to the villages they operate in, as a result, we can easily ask the group village headman to disseminate the information to his community through his team.

**Interviewer:**	in this case you mean breaking the community meetings into smaller sections to meet with sub-chiefs?

**PM06:**	yes, let us take for example: chief Gomani has 20 sub-chiefs, this means to say: if the rest of the chiefs can do the same, it will be easier to have the information disseminated to several people in different villages in a short period, instead of disseminating the information by going village by village at a time, which may take ages to reach out to the community. Information disseminated in 8 villages at one go can go a mile in a short time.

**Interviewer:**	let us proceed, in your own opinion, what kind of benefit do you feel can be there when one participates in the pneumococcal CHIM study among people living with HIV, which involves inoculating a pneumococcus bacteria in their bodies?

**PM06:**	in this case, are you implying to a germ that causes HIV or TB not so?

**Interviewer:**	in this case….(PM06 interrupts)

**PM06:**	in this case, I also have another question here before you answer the question you have given

**Interviewer:**	yes, go ahead

**PM06:**	should we say: an HIV positive individual rarely develops pneumonia, because the way I understand your question: it like you are implying: “inoculating a pneumococcus bacteria in an HIV participant?”

**Interviewer:**	that is not what I mean in the question, what I mean is: every individual has pneumonia

**PM06:**	but why have you opted to inoculate the pneumococcus bacteria in an HIV participant?

**PM03:**	it is because this person is already at risk of suffering from pneumonia in order to do a trial on this person so that once a fellow HIV positive individual suffers from pneumonia in the future, they should be able to be treated using the same vaccine.

**Interviewer:**	that is her opinion, what do others say?

**PM02:**	in my own opinion, I feel that it is a good idea to try this bacteria on an HIV-positive individual because this person already has the disease and the research is being done to find something to prevent other individuals of the same condition from suffering from pneumonia so that when the worse comes to worst, they should be in a position to get their special pneumococcal vaccine, therefore, I feel this is a very good way to go.

**Interviewer:**	alright, what do others say?

**PM04:**	my question is: since HIV is described as an infection that attacks the body with different diseases and once you inoculate this bacteria in the participant’s body, this person will be found suffering from TB as well, in this case, how will the reaction be like; since this person is already on ARV treatment and is required to take the pneumococcal vaccine. I feel it would be a good idea to inoculate this bacteria in people who are HIV-negative so that once someone gets infected with the disease, they should easily get treated with the vaccine that is in the process of being developed for this study.

**Interviewer:**	Alright, let me clarify this, I don’t mean that an HIV positive person should get a TB vaccine as well but a pneumococcus bacteria should be inoculated in their body. Therefore, in this question, I am referring to an HIV positive individual inoculated with the bacteria in their body. In terms of the TB vaccine, BCG vaccines are given to health people. Let us not mix up situations here, because these are different. Of course it begins as a single thing but later own develops branches. My question is: do you think it will be beneficial to have this pneumococcus bacteria inoculated in the bodies of people with HIV? If you feel there is no benefit in doing that, let us give our opinions and the reasons for giving such opinions.

**PM03:**	for something to be known to the public, for example: for the public to know that this drug cures such-such a disease, it means a research should be done on a health person for it to prove its effectiveness. If this experiment is done on an HIV negative individual, then the effectiveness of the vaccine will easily be seen, but if the experiment is done on an HIV positive individual, it will be hard to see the effectiveness of the drug

**Interviewer:**	which other opinions can you give, what risks do you feel can come as a result of inoculating the pneumococcus bacteria in an HIV positive individual?

**PM04:**	the risks that I feel can exist in such case may include the fact that: the body has been put on pressure since it is already infected, and once we inoculate this bacteria in the body of HIV positive individuals, it will be difficult for the person to react to this other infection since they already have a low immune system, that is my opinion.

**Interviewer:**	alright, what do others say, what other risks can come as a result of inoculating the pneumococcus bacteria in the bodies of HIV positive individuals? Feel free to speak in either English or Chichewa.

**PM06:**	if this bacteria is inoculated in the body of an individual who is already sick it means that we are pressurizing her body in the same way it happens when we overload a vehicle. In short, we need to give an HIV positive individual some space and chose the right things to put in her body since they are already at risk of other diseases

**PM04:**	a trial is supposed to be done on people who are health so as to see the actual results of the study, because if we try something on individuals who are sick, it will be difficult to see the effectiveness of the study

**Interviewer:**	in this case, we are having a hint in terms of the benefits and risks that exist due to the inoculation of the pneumococcus bacteria in HIV positive individual. If you put this on scale: which of the two variables would outweighs the other?

**PM05:**	the variable that outweighs the other is inoculating the pneumococcus bacteria in the body of an HIV negative individual

**Interviewer:**	alright, you mean inoculating the pneumococcus bacteria in an HIV positive individual?

**PM05:**	yes

**Interviewer:**	in this case, I mean: inoculating the pneumococcus bacterial in an HIV positive individual, therefore, I would like to know the benefits and risks of doing so. On a scale, which one of the two variables outweighs the other?

**PM02:**	it seems that people gathered here are campaigning against the inoculation of a pneumococcus bacteria in the bodies of HIV positive people, it would rather be a good idea to inoculate the pneumococcus bacteria in the bodies of health people with an aim of seeing the effectiveness of the trial. Let us take the scenario of a malaria patient, if a trial was to be done on her and then she gets tested for malaria, she will be given a drug that will cure her

**PM06:**	let me conquer with the point that number 06 has given. In this case: he means to say: a person carrying a 50 kg bag is said to be struggling already to bear with the weight and once we stop him over to add an additional 20 kg, I feel this will not be realistic. This is why we are saying: an HIV positive individual is usually loaded with a number of condition, by inoculating the pneumococcus bacteria in this person, we will be jeopardizing his health, making it hard to treat the pneumonia, unless this bacterial is tried on a health individual for effective results.

**PM0:**	it is a fact that an HIV positive individual already has pneumonia, it therefore depends on the strength of their immune system to have the signs and symptoms seen in the individual. So if the pneumococcus bacteria is inoculated in this person’s body, it is likely that the impact of the bacteria will be seen in this person since this person already has a number of conditions developing in their bodies

**Interviewer:**	alright, while still on the same topic, let us come to the issue regarding how we can describe the risks and benefits, how should information regarding this be explained to people?

**PM03:**	I feel that the information should be explained to the chiefs who should disseminate it to the community because chiefs have their own ways of disseminating information to the community, meaning to say: you will come to the village on a specified date to conduct the meeting. After we the chiefs are done with welcoming the community to the meeting we will then give you the stage to give the information about the study to the people since they will be ready to hear from you

**Interviewer:**	let us move to our next topic which is about what difference you feel is there to have a female and male person participate in a research study like this one. Do you feel there can be any kind of difference to have a female or male person participate in a study like this one?

**PM04:**	the difference can be seen when a different vaccine is given to these people but if they are given the same vaccine, no difference will be shown because there is no indication on the drug that states: “this drug is meant for women or men only”

**PM06:**	there is no difference between the two because both the woman and man could be infected with this disease.

**PM05:**	the difference can be there if an individual is inoculated with such a bacteria because people have different genetic makeup, hence a difference in how their bodies respond.

**Interviewer:**	in this case, I would like to differentiate this in terms of: a male and female person participating in a research study like CHIM, where they have a pneumococcus bacteria inoculated in their bodies, the difference can be shown in terms of how the body of each individual responds to the bacteria?

**PM03:**	I feel there will be no difference in the way the bodies of both a woman and man respond to the pneumococcus bacteria because the drug they will use will be the same

**PM02:**	I would like to comment on that. I feel there will be no difference between the two people because each person will suffer from the disease in the process

**Interviewer:**	alright, regarding the work of the other study that took place previously, for instance: it was noted that the population of male people who showed interest to participate in the study was higher compared to the population of women, what do you think could have been the reason for such turn around in the study?

**PM02:**	in most cases, women are afraid of taking risks. This takes us to the scenario we had during the COVID era, the turnaround of women for vaccination was low compared to that of men. In most cases, women tend to develop fear.

**Interviewer:**	what do others say?

**PM06:**	women are usually afraid of taking risks, we men are always bold when doing things. Women are unpredictable, they can go first now and later on change their mind, and this is usually a drawback. Men usually give in and are always ready to take up risks. This is the reason why we agreed to do that.

**PM04:**	even though it was not stipulated on what was going to be involved in the study, most men felt that this disease had affected them most, this remains the reason why men were in forefront to participate in the study.

**Interviewer:**	alright, was the study they participated in, the same as this one; where a pneumococcus bacteria was inoculated in the bodies of health people, and the number of men who participated was high. Additionally, you have said that women rarely take part, we only agree to take part easily change our mind after sometime…..is there any other reason…(interruption)…

**PM06:**	on the other hand, women will first ask their husband’s opinion before they participated as they take it to say: “a man is the head of the house” and by the time the study team is within the community, the man would have gone for work. In so doing, women miss out such opportunities. On the other hand, it all depends on the responses that their husbands give them, either discouraging them from or encouraging them to take part.

**Interviewer:**	in regard to the future research study, there is a plan set for women wishing to participate in this study who are in the child bearing age. Before participating in the study, these women are required to take birth controls. Let me hear your opinions about this issue.

**PM01:**	birth controls should be discussed in a family before their usage. When this is taken without the consent of a partner, it turns to be a very serious issue.

**Interviewer:**	that is his opinion, what do some of us say? May I hear from your opinion, for the sake of planning an activity for women of childbearing age who may show interest to participate in the study so as to be given contraceptives during their participation in the study?

**PM01:**	what measures have you put in place to choose women of child bearing age to take birth controls before participating in the study?

**Interviewer:**	let me answer your question at the end of this discussion, because it seems to appear on the discussion ahead

**PM04:**	as researchers, you are supposed to put in place conditions that are in line with the categories of people who are required to participate in the study, just as I have already said it that: you are not supposed to inoculate a pneumococcus bacteria in an already infected person, a pregnant woman is not supposed to participate in the study, people who are not pregnant should not do this and that, all people allowed to participate in the study should possess the conditions stipulated in the inclusion criteria because different people are living with different conditions; this is to say: “some people are epileptic, therefore a need for an inclusion criterial. The inclusion criteria should be developed by the research team. This gives us an example of a scenario in the dental clinic, where a patient is asked to tell whether they took alcohol or not, so as to ensure that the effectiveness of the drug given during a tooth handling procedure is not compromised.

**PM06:**	I think the inclusion criteria has already been stipulated and contains the following: Asthma patients, diabetic patients and BP patients should not participate in this study.

**PM04:**	meaning to say: if someone is in the child bearing age, they should take birth controls before participating in the study?

**PM06:**	yes, because you cannot tell whether an individual is interested to take birth controls or not.

**Interviewer:**	alright,

**PM01:**	it is good to have women of child bearing age take birth controls before participating in the study, this carries a significant benefit because there is a possibility that during participation, they can easily fall pregnant if birth controls are not taken. This is the reason why I have said: the decision should be a voluntary one made by an individual participant. Some churches don’t allow their members to take birth controls, therefore this will act as a control measure for the study. In this case, only people willing to do what the study requires of them will be allowed to participate in the study. The study does not force participants to take birth controls when they don’t wish to.

**PM04:**	is it good for girls in the child bearing age to be given birth controls, and if so, will this not bring complications to the girl when they wish to give birth in future? I feel this will bring about a lot of complications in the girl in future.

**PM01:**	this is the reason why I have said: birth controls should be considered on voluntary basis before participants begin their participation in the study. Once an individual agrees with what is stipulated in the inclusion criteria, they are fit to participate in the study and if they disagree will definitely not be allowed to participate since some churches don’t allow their members to take birth controls. Issues regarding birth controls should not be something where a woman makes decisions after getting her partner’s consent. Because someone can have about six children where the doctor can request the woman to take birth controls failure to which she can put her health at risk accompanied by negative results. In so doing when a woman of child bearing age is given knowledge on the dangers of bearing many children without giving herself space through birth controls. In the long run, the participating woman ends up asking for birth controls and later on informs her partner about the birth control measure she has adopted. Before a participant is given chance to choose a birth control measure, the doctor asks her to invite her husband to the study site in order to avoid inconveniences in the process.

**Interviewer:**	alright, let me say something on the issue regarding birth controls, I believe we all know that there are different methods of birth control, it is not a thing that people will be forced to take, instead: before participating in the study, people will be briefed on that and it will all be up to them to choose the type of birth control they feel would be convenient for them. Some people use traditional birth controls while others use modern ones depending on their preference. The aim of doing this is to ensure that all participants are safe from conception, this is to prevent them from falling pregnant during the 6^th^ or 4^th^ week of participation in the study. If they were to fall pregnant after having already received BCG, this could be fatal to the unborn child. As we are aware that participants in this study will either be married or single, this may stand them the chance of falling pregnant in the process since they are in the child bearing age.

**PM01:**	I asked the reason why this was being done, and the reason has now been given.

**Interviewer:**	let us continue with discussions. Our next section has partly been tackled. Remember I have already said it that: participants of this study are required to stay in a place where they are closely monitored. As part of this study, we will request study participants to stay in a place set by the study for a few days, in order to monitor them regularly, more especially during the first days of the study after having the bacteria inoculated in their bodies. Now, my question is: What are your views on the issue regarding keeping participants in a place set by the study for close monitoring?

**PM01:**	how long are you going to keep these participants for?

**Interviewer:**	I have already said it that: these participants will be kept for a few days. Previously, the duration of keeping the participants in that manner was: three days and we are now talking about two days. The whole plan allows participants to people to carry out their routine work and later on go to the place set for them. I would like to hear your views on this kind of system. Do you think this system should be used in this study or not, we wish to learn from your views on how you feel this system should go?

**PM04:**	when we have a student participant, someone above 18 years old and in the child bearing age, they should be given a consent form to be signed by their parents, to help the parents know the whereabouts of their girl child on the specified days. Additionally, if the participants are married, their husbands should sign the consent form to act as a witness.

**PM01:**	it has been said that, participants will be allowed to only spend a night there and not the whole day, since nights are the most tempting for couples.

**PM04:**	can you repeat the question.

**Interviewer:**	okay, my question goes like: what are your views on the issue of keeping the participants in a place set by the study; “Grace Bandawe”?

**PM02:**	I feel it is good to keep these participants in a place set for them by the study for a specified period of time, be it three days or whatever days, they will need to stay there for the specified period of time, because whenever they will be leaving their respective homes, everyone will be aware of the situation at hand. It will be bad for an individual to participate in the study without informing their family members first. They should inform their family members about a study they have enrolled in so that whenever they are leaving the house, the family members should know about the program.

**PM06:**	it is good because this will help their care givers, especially the doctors, so that follow ups on their participants should be effective since these participants will be closely monitored

**Interviewer:**	do you have any other opinion on the matter?

**PM06:**	none

**PM02:**	this is something different from what researchers who conduct door to door interviews do. In this case: an interview can be set for a specified date but participants may not be available in their respective homes. However, if participants are gathered in one place, it will be easy to monitor them at once. Let us take for instance: researchers who conduct door to door visits may plan to visit participants on a Tuesday and come Tuesday, they don’t make it for interviews, this becomes difficult to proceed with the interviews. On the other hand, let us take a scenario whereby a participant being handled from her home, she falls sick in the night, it will be difficult for the doctor to follow up on her unlike if she stayed close to the doctor for monitoring and care.

**Interviewer:**	this shows that we have a general consensus on the matter; this shows that the system is helpful and should be practiced?

**All:**	of course yes

**Interviewer:**	I would like to learn from you. Who should be the guardian of the participant? Because whenever a participant is enrolled in the study, she must have a name of a trust wealth person to act as her guardian in case something happens to me, this person should be approached in case I am not reachable.

**PM02:**	let us take a scenario whereby a man like me marries a woman, my wife should be the guardian when I fall ill and when my wife gets enrolled in a study, I will definitely become her guardian and if a child falls ill, her parents should be her guardian to approve such a situation, my opinion though.

**Interviewer:**	what do others say, we have heard our fellow team member mention that a married couple should be each other’s guardian.

**PM01:**	it all depends on the family member you have around, someone who can be there for you, it maybe your mother, father or uncle, any person you feel can be there for you in times of need.

**Interviewer:**	is there anything you would like to add on this discussion before we proceed? Our next discussion will be based on what the study will involve. As part of this study, participants will be given a BCG injection as a way of inoculating a bacteria into their body. This BCG is just the same as any other BCG that children are given as a normal vaccine in Malawi. However, this vaccine will be given in different doses, this is the only difference that will be there between these vaccines. The normal BCG is given once, while the BCG that is going to be given in this study will be given in different doses in order to carryout different tests including: collecting a sample of flesh from the injection site to see if there is any presence of BCG and have it tested for its immune response. What is your perception on the issue regarding BCG into the body?

**PM04:**	this is a recommended measure because I don’t see any other way to have the bacteria inoculated into the body, unless there is an aural method to have the bacteria in the body.

**PM03:**	when an individual agrees to have this bacteria inoculated in their body, they need to have it injected in their body. If there is another way of placing the BCG in the body aside the injection one, an individual participant should choose a method of their preference.

**Interviewer:**	alright, on this same issue regarding an injection, I have read it from the information sheet that a sample of flesh will be taken from the injection site to test for the presence of BCG in the body, what have you got to say on such a process?

**PM04:**	how long will it take you to collect a sample of flesh after a bacteria is inoculated into the body?

**Interviewer:**	in that case, I feel this is something that will be done in the future, something that has not been established yet. I cannot tell when the research team will come to collect the sample from the injection site to do some tests to see the presence of BCG. Therefore, I am unable to tell the days when the research team will come to collect this sample.

**PM03:**	meaning to say: after the participant is given the injection, it will take some days before the research team comes to collect a sample of flesh from the injection site. The sample collection will not happen the same day, this is to ensure the BCG settles in the body, this is why it is planned to have the sample collection after a few days of BCG injection, I believe the whole aim of sample collection is to test for the presence of BCG?

**Interviewer:**	yes. What do others say?

**PM06:**	it is possible because this is one of the measures that can facilitate the whole process of establishing the study, because if this doesn’t happen, there will be some gaps in the study. Therefore, it is important to have this sample taken.

**PM03:**	I would also like to know whether the BCG remains on the injection site after the injection, or this will be filled throughout the body, meaning to say: the sample should only be taken from the injection site or elsewhere? Because what I know is that: when an individual has an infection, it means the infection will be spread in the blood stream and if the sample is taken through a finger prick, injection site or elsewhere, the sample taken will bear the same thing present in the blood stream. I would like to know whether the BCG only stays on the injection site after injection or maybe it also spreads throughout the body.

**Interviewer:**	let me answer this way: when the BCG is injected in the body it spreads all over the bloodstream. Therefore, when I say sample collection: I mean both the flesh and blood. This means: the presence of BCG will be tested from both the flesh and blood to find out where exactly the BCG can be found.

**PM03:**	now, the fact that the sample of flesh has been taken, how will it be stored?

**Interviewer:**	the sample of flesh is collected by skilled health care workers and the collected sample is very small and can hardly be seen. Usually the sample is collected in small portion and is stored in the tubes given for such kind of sample and then the sample is taken to the lab for testing.

**PM03:**	does this mean each sample will have a barcode?

**Interviewer:**	of course yes, each sample will have an identification code for better identification of the participants.

**PM03:**	alright

**Interviewer:**	what is your opinion or concern on the collection of a sample of flesh from the injection site?

**PM04:**	people have different perceptions of things. There are some people who find it hard to understand things, because the thing that I can easily understand will not be understood by someone else, instead they can view it in another way, but it will be wise to get some training, because whenever an individual is enrolled in the study, they may already have agreed with all what will be involved in the study, as you have already said it that: an individual has the right to withdraw from the study at any time. Some of the reasons why they may wish to withdraw from the study can be: lack of understanding what the research team is going to do with the sample of the flesh taken from them

**Interviewer:**	alright, let us proceed. There are different ways of administering the BCG vaccine, one of them is by inoculating BCG in the lungs. We have talked about the first one being the injection one, the other way is by inoculating the BCG in the lungs using a pipe that is inserted in the nose or mouth through the throat to the lungs. This method is also used to collect samples from the lungs, what is your concern on this kind of method?

**PM05:**	I feel most people are afraid of using this method of passing the pipes to the lungs because this method causes most people to develop different infections to the point that some individuals don’t get cured from their conditions. This is the reason why most people don’t agree with this method.

**Interviewer:**	what do others say?

**PM02:**	there should have been another way and not placing a pipe through the nose or mouth to the lungs

**PM03:**	most people did not agree with the method of placing a pipe through the mouth to the lungs. It would be ideal to find another way to get the lungs in order to collect some samples from a living person, this method of placing a tube in the mouth or nose of an individual through the lungs can be ideal for a dead person. If another way of collecting these samples could exist, things may work better.

**Interviewer:**	alright, if there can be another way of doing that, aside the current method?

**PM03:**	most people disagree with this kind of method.

**Interviewer:**	is there anything else you wish to add on this?

**PM06:**	this is the only method, most people disagree with it.

**Interviewer:**	most people disagree with this kind of method?

**PM06:**	most people disagree with this kind of method

**Interviewer:**	what is the difference between the injection method and this kind of method, which method between the two can people opt for?

**PM06:**	the injection one

**Interviewer:**	why do people disagree with the injection method?

**PM06:**	it is because when the injection is given, the drug spreads throughout the body.

**Interviewer:**	alright, is there another reason or maybe you feel this is the only main method that has been used all along. Therefore, it would be easy to accept this kind of method?

**PM03:**	you have said: a TB bacteria will be inoculated in the body, what type of TB is that, because we have different types of TB? We have TB of the bones and TB that affects the lungs and the blood. The most common TB is the one spread through air, I am not sure whether this TB will be passed to the lungs if an individual was to be injected the BCG. Because the most common TB is associated with coughing. Which type of TB are you going to try on the participant, is it the TB that affects the respiratory system, the blood or the lungs? If it is the TB associated with the respiratory system, then this same method of placing a pipe in the mouth or nose to the lungs will be required. I don’t know whether it will be possible for the TB to be inoculate in the lungs?

**Interviewer:**	well, that is another opinion. Is there any other opinion on the matter, while still on the same topic of TB, anything that you have remembered and wish to add on what has already been said?

**All:**	no

**Interviewer:**	I would like to learn from you, how can you feel if you were to be told by a friend or relative that they have been enrolled in this study?

**PM03:**	if a friend or relative comes to inform me about their involvement in the study, I will not discourage them because it is not possible that someone can discourage an individual from going to the hospital when they are unwell, but I would encourage them instead of building fear in them. That is my opinion.

**Interviewer:**	alright, this person has given us his opinion saying: he cannot discourage or build fear in a person wishing to participate, what can we tell a friend or relative that may wish to participate in such a study, how can we feel?

**PM06:**	it is a good thing to see someone volunteer to participate in a research study, because everyone is said to have a right to do so. An individual will be encouraged to participate in the study because this is another way of promoting development in the nation as this will create a way of combating this disease through a research study. However, we will encourage an individual to participate in the study instead.

**PM02:**	if I was to come across a friend or relative enrolled in this study, it would be wise for us; the leaders to encourage such people, because discouraging them will cause them to move backwards. It would be good to allow people to enroll in the study on their own, we will only be there to congratulate them when we meet with them on the way. We would again encourage them to participate in the study.

**PM05:**	I would of course encourage such people to participate, but what if they asked us this question: “what are we going to benefit from the study,” will there be any kind of insurance or compensation?”

**Interviewer:**	I get the question

**PM04:**	it all depends on the explanation we give to the participants, as you have already said it that: sometimes you will be visiting people in the community to explain to the community about the benefits of the study, study results so that whenever an individual decides to participate in the study. Participants should know that they are not only doing this for themselves but the community as a whole. If someone reaches the point of taking part in the study, it means they have agreed to sacrifice their lives meaning to say: when something good happens, it would be as a result of an individual participant having sacrificed their lives to save many. In addition, the training sessions should give a hint to the participant about what the study benefits are.

**PM06:**	we are indeed supposed to encourage these people depending on what we have learnt from this meeting. From what we have learnt here, it shows that this research study has no harm. We are not supposed to discourage these participants but should be one of those people encouraging them. This is why I have said: we are part of the people who are entitled to taking part in the study.

**Interviewer:**	alright,	what comment do you have on the discussions we just had?

**PM04:**	what we have learnt here is good, only that the information has reached out to a few people. I would request you to let people know the benefits of and results of the study. People should be told about what they will be required to do when taking part in the study and what the study results are once they come out. When there is picture about what the study is all about, people will not see any benefit of participating in the study if what they only see happening in the study is having samples taken from their bodies, instead, there should be a good strategy used for the study, leaders should also take the responsibility of encouraging community members to participate in the study with the mindset that: study results will benefit our country, village as well as our community.

**Interviewer:**	thank you very much, do you have any comment?

**PM05:**	this research study is really important although someone has said it that: the study should be explained clearly to the public but it would not be a good idea to have it explained to the public, instead chiefs should also be inviting their subordinates to such meetings so as to get involved in disseminating the study information to the community. Therefore, by involving chief’s subordinates in such meetings, information will be disseminated to the public fast.

**Interviewer:**	alright, thank you. Are there any other comments you would wish to give?

**PM03:**	I think we should be holding meetings where this issue should be presented, because if we only take our subordinates to the meeting, it will not carry weight, but if these meetings can be conducted in an open place like a playground to allow the information to reach out to many people. If we only invite our subordinates, we will not manage disseminating the information correctly, because when we are asked questions about the study, we will not manage to give answers, but you are the right people to give us the answers. I would request you to be informing us about the days you will be holding meetings with us, so that I remind you to come with a mega phone and go around the community to inform people about a meeting you would like to hold on a specified date and place. It would be good if this was to be done that way, because everyone including doctors need to meet with people, because there are some questions that we cannot manage to answer and you would be in a better position to answer them

**PM04:**	in addition to that, all the information should be taken to the hospital because that is where many people are found and it would be easier for them to access the information. For instance when women attend antenatal care, they get a health talk before the begin accessing antenatal care services. If people were to be given such information during this time, it would be a good idea.

**Interviewer:**	thank you, is there another comment you would like to give?

**PM06:**	this research study is good, and it will depend on the study findings. When the results of the study are good, this will lead to the reduction of diseases that are fatal in this country. On the other hand, these results will lead to a meaningful meeting.

**Interviewer:**	thank you, if there is no any other comment, let’s end our discussions here. I would like to thank you all for your commitment to attend this study meeting, on behalf of all my colleagues conducting this study, may I thank you all for taking your time to participate in this study. Thank you very much.

**The End!**!!
