# Supplementary material for: Acceptability of advanced Controlled Human Infection Models: The case of a pneumococcal challenge in people living with HIV and mycobacterial challenge with Bacillus Calmette Guerin in Malawi
Source: PLOS Glob Public Health. 2026 Aug 18;6(8):e0006236. doi: 10.1371/journal.pgph.0006236 (PMC13485124; doi:10.1371/journal.pgph.0006236)
Supplement: Supplementary file 1 [file pgph.0006236.s001.zip › Documents/ACHIMA_FORMER TB PATIENTS_FGD_20_14.03.24 240314_1428.docx]

**NAME:	FGD**

**SEX:	MALE**

**PROFESSION:	FORMER TB PATIENT**

**LOCATION:	NOT MENTIONED**

**Video:**	Vaccines prevent diseases and save countless lives. But will it take him 10 years or more to develop new vaccines? We need to find a faster way. One way to speed up the process is by making better use of human infection studies. Volunteers are given a sample of a vaccine under development, followed by a carefully monitored infection. Medical care is always close to hand if volunteers fall ill. But if they stay well, it means the vaccine works and can be made available much earlier. The idea is almost 300 years old, but using today's advanced technology, this type of study is not just scientifically sound, but ethically responsible children, and of course, follow strict rules.

Human infection sites show huge promise in the fight against diseases like typhoid and malaria. But to develop vaccines most effective for people at risk of disease, we need to test them on those most at risk. By expanding human infection sites and targeting them better, the people most at risk of diseases could help develop the vaccines of the future and save millions more lives. It's a given that vaccines prevent diseases and save countless lives. But will it take him 10 years or more to develop new vaccines? We need to find a faster way.

One way to speed up the process is by making better use of human infection studies. Volunteers are given a sample of a vaccine under development, followed by a carefully monitored infection. Medical care is always close at hand if volunteers fall ill. But if they stay well, it means the vaccine works and can be made available much earlier. The idea is almost 300 years old. But using today's advanced technology, this type of study is not just scientifically sound but ethically responsible too. And of course, follows strict rules. Human infection states show huge promise in the fight against diseases like typhoid and malaria. But to develop vaccines most effective for people at risk of disease, we need to test them on those most at risk. By expanding human infection states and targeting them better, the people most at risk of diseases could help develop the vaccines of the future and save millions more lives.

**Interviewer:**	the video explains that it has been noted that vaccines protects people from infections. It takes a very long period of time for a vaccine to be made available. The easiest way to make vaccines available is through conducting clinical trials in people who are already at risk of diseases to check for their body immune response. We will dwell much on a study about BCG. BCG is a vaccine against tuberculosis in health people to check how their bodies respond to a vaccine. Just as stated in the video, once people volunteer to participate in a study, they remain under monitoring throughout the study period, once the research team observes that a participant doesn’t develop any health problems after taking BCG, it will mean that all is working effectively. However, if an adverse event occurs, the research team comes in to take care of the participant by giving them appropriate medication to ensure they get better. That is the issue I have brought here for discussion. Welcome people over there, you came in when we were done with the other part of the discussions. I will give each one of you an identification numbers that you will be required to use before giving individual ideas during our discussions. First and foremost, I would like to learn from you. What have you grasped from the information you got this afternoon?

**PF02:**	the information that I have grasped from the afternoon discussions is: the vaccine has been existing from long time ago. I have received this type of vaccine before. Due to no improvement to prove the vaccine is effective, people had to be vaccinated again. However, people are growing older and still keep suffering from the same disease that the vaccine fights against, despite their previous jabs.

**Interviewer:**	what do others say about the information you got from the information you were given in the afternoon?

**PF05:**	what I got from the information is that: we will be given a bacteria and be monitored…I have forgotten the other part.

**Interviewer:**	you have forgotten the other part?

**PF02:**	yes

**PM06:**	what I grasped from the information I got this afternoon is that: whenever we are joining a study, there are measures we are required to follow in order to keep health. For example: we need to eat well; take fruits and do.

**PF04:**	the information I got this afternoon is that: people with HIV should get a pneumococcal vaccine

**Interviewer:**	according to the information you got this afternoon, do you feel this type of research study will be successful in our country?

**PM01:**	it is all possible to have this research study done successfully in our country provided people are approached in a good way, as it is difficult for Malawians to understand issues regarding drugs due to cultural beliefs. Therefore, I would say: it is all possible to have this study conducted in Malawi. However, there is need to put more effort on civic education on the aims for conducting the study. People have negative attitudes towards clinical research, for instance: previously we had issues with the COVID vaccine. Most people in Malawi did not get the vaccine because of vague reasons. In addition, most of the reasons for not taking the vaccine were due to perceptions rather than facts. Therefore, there will be need to put much emphasis on civic education so that people understand the whole concept of the study that states: “most vaccines we have today have fought against a number of diseases that have previously destroyed people’s lives.” These vaccines were established through the same clinical trials. Clinical trials have been there for long. We will continue with clinical trials, but most importantly, people need more civic education on this.

**Interviewer:**	what do others say?

**PF03:**	on the same issue regarding vaccines, it is possible for the trial to be conducted in this country. People who got vaccinated before should be supported in their respective communities to make the rest of the community understand the whole concept about vaccines without any problem since vaccines react differently in different people.

**Interviewer:**	what do others say, number 06, you seem to have something to say?

**PM06:**	most of the things have already been said

**PF04:**	the vaccine will be used in HIV positive people. The aim is to find out how the vaccine functions in the body. Pneumonia attacks everyone, it can be HIV positive or HIV negative people. Won’t it be possible to try this on the two categories of people to find out how it functions?

**Interviewer:**	let me answer your question this way: we have the other section of the study where a pneumococcal trial was conducted among a small sample of health people who did not have any underlying diseases. Currently, we wish to conduct this trial in people with low immune system and are already on treatment to see what is going to come out from the results. The first trial was done in health people with no any underlying diseases because pneumonia just like TB can attack anybody. The first trial was done on health people to find a step to move forward. From the information about the study that you got today, which information do you feel is very important?

**PM03:**	the information is very important. This is the information that needs civic education to ensure people understand it better. If the information was to be disseminated anyhow, there will be a lot of misconception about the information which will be contrary to the facts about the whole information. For example there will be misconceptions like: “the aim of the study is just to finish you up,” this is why there is need for more civic education. Once this study becomes successful, it will benefit more lives

**Interviewer:**	which procedures should be followed in order to get more people to participate in the study?

**PF02:**	just as already stated: people should be given civic education on the matter to give them an understanding of what is exactly happening to assure them there will safety after getting vaccinated. Participants should be assured that the vaccine will not bring them any adverse events. This will make every participant sure of the benefits and risks involved after vaccination. This will make them understand they are putting their lives at risk not only for their benefit but the benefit of the rest of the population

**PF05:**	I believe we have come here to learn. The other good way to take in order to get more people participate in the study is: we should take the role of finding people who have been on TB treatment before and people who are HIV positive. These people should be approached and be given information about the study so that when you come to conduct the study in their community, you should have more people enrolling in the study depending on how they accept the study

**PM06:**	the other way can be: doing it the way the health sector does it through door to door visits. You should as well employ people who will conduct door to door visits. When the community sees a health worker disseminating information regarding health, they feel safer compared to having a mere community member disseminating that. In this case, the people you will employ will go door by door to explain more about the vaccine. There will be people in the community that will accept the study while some people will not, just the way it was during COVID. Some people understood the whole concept about COVID while others did not. This will be an effective way; the door to door visit would be effective in this case. The other way is to give the best measures to follow, for instance: if I fall ill, how would the health care team reach out to me, because after enrolling in a study I need to be followed up to have my wellbeing checked during the progress of the study.

**PM01:**	just to add on what has already been said, for those of us that are participating in the study, remember we are talking about measures to be followed to ease the progress of the study. Participants should be given enough civic education about the study to ensure we have enough knowledge so that whenever we approach people out there, we should be in a position to answer some questions that we may be asked. We have different people with different knowledge about clinical information; some may have more knowledge on this while others don’t. Therefore, if we can go out there with little information on this, we will not go far. Additionally, it has already been stated by this fellow group member that once people realize they are dealing with someone from the health sector, people develop trust in them. When informing people about this, there is need to put more emphasis on civic education so that we should be confident about what we are doing. For example: we need more knowledge on this issue regarding vaccination. How far have people handling this gone. These questions will be expected in the process. The other thing I would like to say is: we are discussing the pneumococcal vaccine here and are also talking about tuberculosis. Will this tuberculosis vaccine be tried on people who have ever suffered from TB before or what? Is it your first time to administer the pneumococcal vaccine in Malawi or maybe it is only given in communities where pneumonia is an issue? I wish to have clear understanding on such areas before the study resumes.

**Interviewer:**	let me respond this way: in terms of pneumonia, there is a type of vaccine that children are given known as PCV which is given to children. But now, we are looking at the pneumococcal vaccine being given to people with underlying diseases and are already on treatment. This is the category of people that will be approached; they will not be approached with a vaccine but rather be given a sample of the pneumococcal bacteria to check for their immune response. The other question you asked is about TB; the BCG vaccine

**PM01:**	before you proceed, does it mean the issue to do with pneumonia is not about vaccine, but is about doing a trial on people?

**Interviewer:**	yes, to check for immune response

**PM01:**	when you are talking about TB, you are referring to a vaccine to check how people can be prevented from contracting TB. My question is: when these people are given the bacteria, will they be placed in an environment that will put them at risk of contracting tuberculosis like the way we are trying it here?

**Interviewer:**	no, in this case, I will just say: “no,” because this study is just at the initial stage and the ideal measure that is required to be followed is not yet known, since we are just in the phase of learning from your ideas at the moment. Once these ideas are approved, all people who have been approached have given their ideas and a report has been written proving that the reviewers are satisfied that this is a very important thing, that is when decisions will be made, to develop measures to follow when conducting this study

**PM01:**	meaning that some of us are asking about things that are going to take place in future?

**Interviewer:**	yes…(all laugh)

**PF05:**	the first thing to do is to conduct a survey to prove whether this should proceed or not, or maybe you have fears about it and feels it should not proceed. That is the whole concept about it. Of course we have a number of questions to ask, we will find a forum to ask those questions

**PF04:**	you have said: pneumococcal is not a vaccine but rather a bacteria established to be tried on a person, what is the benefit there; whenever the bacteria is tried in a person, what if the person dies or whatsoever happens to her, I don’t see any benefit with that, because all you want to know with this experiment is to check if people will survive or not. You will not even give them any medication?

**Interviewer:**	the fact is that: as these trials are being done, they will help decide whether to establish a vaccines in future or not. In short, all they wish to do in this case is to find out how things will work

**PF05:**	I think what she is trying to mean here is: the health care team will be present when you are given the bacteria so that in case of any adverse event, you will be handled to the health care team to put you on treatment, to fight against the bacteria so that you should not fall sick because of that bacteria

**PM01:**	just to add on this; as individuals volunteer to have a bacteria put in their body, they need to understand that they are there to fight for other people in the future. Therefore, whatever will happen in the process, it will be taken care of.

**PF05:**	just as it has already been said: this procedure will be carried out by the health care team. It has already been stated that this procedure will be done in people who are at risk of diseases and not health people. However, the research study aims at working with people at risk of diseases in order to check for their immune response to see whether these people can get better after being given some medication or maybe end up dying. This will cause the research team decide whether to proceed with this type of research study or not.

**Interviewer:**	I think she is trying to refer to two issues here, but let us focus on one. Let us focus on the issue about TB. We will focus much on the TB vaccine that will be administered. I think the confusion is coming in because of the two things that have been mentioned here, making some people responding in terms of this part while others are responding in terms of the other part. I will urge you to stick on the BCG topic. Now that I have talked about the section regarding the research study focusing on TB in terms of BCG vaccine that will be administered to participants. How will this research study help in coming up with an effective BCG vaccine?

**PF04:**	after participants are done with getting a BCG vaccine, they go out there to inform the rest of the community that they also got the BCG vaccine and once people out there find that this person is well, they get encouraged and wish to get the BCG vaccine as well.

**PF03:**	I don’t work at the AETC. Whenever HIV positive individuals go to seek for ART, this is the time when participants recruited in a study. This category of people encourages people to enroll in the study. People with HIV assures the rest of the community that once they got tested and were diagnosed with HIV, they will live longer provided they adhered to treatment, in so doing, they will remain looking health like them. In short you are required to move with people who have once had such experience, they will help you motivate the rest to enroll in the study. Meaning to say: people who once got vaccinated will have their word taken seriously compared to your word because you will not have evidence to show them about your experience.

**PM06:**	have you already done a survey on the vaccine and found out how long it takes for the vaccine to show its side effects after an individual gets vaccinated?

**Interviewer:**	this vaccine has not yet been examined, I will keep repeating myself that: we are just in the planning phase and would like to hear from people. I don’t have answers for the questions at the moment.

**PM01:**	let us take a scenario whereby an individual goes to buy Panadol. They understand that once they take Panadol, they get relieved from headache within two hours, the same with a trial vaccine, an individual participant will be given the vaccine and expected to show side effects within a certain period of time aside being under close monitoring by the research team?

**Interviewer:**	let me respond this way: since the vaccine has not yet been established, it will be hard to tell about the length of time it will take to see its side effects in people once they got vaccinated. It seems I am bringing confusions among you whenever I respond to those other questions you keep asking me, because I am referring to a pneumococcal vaccine that was administered some time back, this shows how confusing this is becoming for you

**PM01:**	what this question is referring to here is: what will happen when the vaccine begins to be administered, when people begin getting enrolled in the study, I think this should be when the question will be answered

**Interviewer:**	for the research studies that took place sometime back, each and every study has had a period it was required to run to reach its completion. For instance: let’s take this study will run for 6 months, it means: an individual will be told about how long the study will run immediately they enroll in the study, be it 6 months or whatsoever period. I think the question you missed here is: when people enroll in a study, they are monitored closely during the first days, I think you did not get this part? In the first place, I said something like: when someone enrolls in the study, they are expected to be under close monitoring by the doctors or nurses and once the study phases out, the research team doesn’t abandon them. When a participant experiences some adverse events while at home, they are encouraged to contact the research team to inform them about whatever they are experiencing. The aim for doing that is for the research team to take full responsibility of taking care of this individual participant throughout the time, until they get better.

**All:**	I think this question was asked before and has already been answered, I think our fellow member did not get it right. This participant will be under close monitoring

**Interviewer:**	let us proceed. Regarding this type of study; BCG, who do you think is the right person to participate, between men and women?

**PM01:**	I think anybody should participate in the study, regardless their gender. It should only depend on how they feel about enrolling in the study. This is all about volunteering to participate and there should be information given to people before they decide to participate, because a survey has shown that this type of disease is disturbing people, more especially those with suppressed immune system. The study aims at helping such people to get relieved from this disease burden. I think both men and women should participate in the study, depending on their preferences.

**PF05:**	I agree with what this person has said: everyone is allowed to participate in the study

**PF01:**	I also agree with her

**Interviewer:**	but that is not your number, you have taken someone’s number

**PM02:**	sorry, I am number two, I agree with my fellow member, because diseases don’t choose the type of person to attack. Anybody can contract an infection, be it male of female both of them can suffer from TB or pneumonia

**Interviewer:**	this shows there is no difference between a man and woman?

**All:**	there is no difference, both a man and woman can contract infections

**Interviewer:**	regarding a pneumococcal study that took place previously, it shows that most men enrolled in the study compared to women. What do you think could have been the reason for most men to participate in the study?

**PM06:**	most of the times a woman gets discouraged because of a number of things, for instance: when a woman is breastfeeding, they would not enroll in the study considering the fact that the baby is still breastfeeding, participating in the study would lead to weaning the baby before the right time. In the long run, this would create room for more women to participate instead.

**PF02:**	my comment here is that: men and women are different creatures, the biological make up of women allows them not to hold pathogens in their bodies compared to that of men. Men are mostly at risk of holding several pathogens in their bodies, this is why I feel most of them are quick to deciding to participate in different studies in fear of holding infections in their bodies that can lead them to poor health, in so doing, this makes them seek medical care.

**PF03:**	the reason why most HIV positive men enrolled in this kind of study was because their immune system was suppressed. In addition, most men show up at the hospital to seek treatment because of being shy to get there individually but rather enroll in different clinical studies to access their medical care easily. I think this should be the reason why most of them enrolled in this type of study compared to women.

**PF04:**	I think the reason why most men enrolled in this type of study is that: information about the study did not reach out to both sides equally, most women get involved in several community activities. They are usually the first to participate in community activities compared to men. Therefore, even when it comes to going to a clinic to medical care, most women are present compared to men. Men usually get shy to show up at the clinic for medical care. This should be the reason why most men enrolled in the study compared to women.

**PM01:**	it is a surprise to me, because women mostly get more information about things compared to men because they go for antenatal care, undergo various tests including HIV. In most homes, women are the first people to undergo an HIV test due to pregnancy to ensure the unborn baby is safe throughout pregnancy. In this case, it sounds strange to find most men enrolling in this type of study compared to women. By the way, was this type of study done within Malawi or it took place outside Malawi?

**Interviewer:**	this took place right here in Blantyre, Malawi

**PM01:**	maybe information about this study reached out to most men and that the means used to disseminate the study information only favored men. Therefore, if this information was available in hospitals, most women would have been aware of the study and would have enrolled. All in all, most women usually become bold to participate in research studies. The other thing is: due to a lot of things said by people, it shows that most women take hearsays seriously, this brings them negative attitudes towards things. Whenever I am out in public, it is usually women I find saying negative things about research studies. However, men usually take things positively, this case it could have been that women took negative hearsays about the study seriously.

**Interviewer:**	let me respond on the means that were used to disseminate information about the study. The means included: going to health centres and giving awareness to people about the study. When people came to the study site, they found most men available compared to women. Otherwise information was disseminated in health centres as it normally does. Let us proceed. While still on the same issue about participating in a study, let us take the plan set about conducting a BCG challenge in women of child bearing age; 18 years and above. Women who wish to enroll in the study will be required to take contraceptives. What are your views on this?

**PF03:**	let us take a scenario that: someone would like to participate in the study but is not married, what will happen to such people then?

**Interviewer:**	my question is: according to the plan of the study, a woman of child bearing age is required to take contraceptives before participating. At this point of time, I would like to hear from your views, having come up with a concern about what will happen to a woman of child bearing age who wishes to enroll in the study but is single, which category of these women should be told about contraceptives in this case? I will return this question to you as I wish to hear from your views on this part.

**PF03:**	I think people who are required to participate in this study should be women on contraceptives to ensure they do not fall pregnant in the process of the study, although you will still find some single women wishing to participate but are not on contraceptives. But since you have asked me this question, I think the question should have been: we have come with a research study and those of you that are single should take contraceptives to participate in the study, to ensure that no one is found pregnant in the process of the study,” since the aim of the study is to work with women of child bearing age but are not pregnant.

**PM01:**	just to add on this: your target group seem to be those women on contraceptives as those who may not be on contraceptives are likely to be found in a certain condition in the long run. The issue here is about safety because whenever a woman is pregnant, her immune system definitely weakens. This is due to their biological make up and sometimes we may think that the immune system is low because of what we are doing. Therefore, every participant should be well geared to ensure no adverse events develops in the process just as she has mentioned it. As such, this is the reason why the research requires all participants to take contraceptives to ensure that no participant falls pregnant in the course of the study. That is my opinion.

**Interviewer:**	which other views do you have regarding women volunteering to participate in the study, so that during their participation, they should not experience anything negative?

**PM06:**	just to add on that, there may be some women who are not married and do not take contraceptives. They think they cannot take any advice regarding that. Some women don’t take contraceptives, they can stay up to three years without taking contraceptives. Therefore, there should be a measure to take for people willing to participate in the study. It will be up to those women not taking contraceptives to decide whether to participate in the study and follow the required procedures when participating in the study.

**Interviewer:**	in this case, the contraceptives being mentioned here will depend on the different types of contraceptives people take. Some people use traditional contraceptives while others use the modern ones. Thus, all participants will be required to use contraceptives of their preferences to ensure no one falls pregnant in the course of the study. During pregnancy, participating women are likely to develop different adverse events that may put their unborn babies at risk. The aim of wanting all participants to take contraceptives is to ensure they are safe from any risk outside the study.

**PF03:**	so is this only meant for people who do not expect to fall pregnant?

**Interviewer:**	in this case, let us not only target those who cannot fall pregnant, because by doing so we will block other women from participating.

**PF04:**	in terms of contraceptives, should we say you will require women to take contraceptives by compulsory?

**Interviewer:**	no, the contraceptives will not be given to women by compulsory but rather asking them to use contraceptive of their preference, as you know that people are divided into different categories. There are some people who are sexually active while others are not. Henceforth, this study only requires participants who are at risk of getting pregnant during the course of the study. The study is therefore targeting Women from 18 to 45 years of age, these are women of child bearing age. Therefore, my question is based on having participants take contraceptives to ensure they don’t fall pregnant in the course of the study

**PF04:**	should we say this question is going to those monitoring participants, if so, I urge those people to give enough civic education to the participating women on how they should manage things

**Interviewer:**	alright, we talked about something regarding; referring study participants to the study sites for close monitoring, do you think this is an important issue or not?

**PF02:**	this is an important issue because an individual will not know the side effects of the vaccine, but whenever an individual is in the hands of health care workers, you will understand how your condition was like before you came to the hospital and after receiving the vaccine. Therefore, it would be wise to stay close to the research team for close monitoring so that in case adverse events occur, you will easily communicate to health care workers in time, for treatment.

**Interviewer:**	how long should the participants be at the research site for, after being given the BCG vaccine?

**PF04:**	I think it will all depend on when the side effects show up in an individual for instance in three days’ time. Some individuals can experience side effects of a vaccine and be asked to stay for observations, while others can be released right away due to not showing side effects immediately but be followed up to check for their wellbeing

**Interviewer:**	which other views do you have?

**PM01:**	just to add on this; it will all depend on the available results. If the results show the presence of adverse events, obviously the individual will spend some time at the observation site. However, whether an individual has side effects or not, she still needs to have someone to monitor her health, because you may not know when she may experience some adverse events. This the reason why a participant is required to have their health monitored, to ensure they do not experience problems in the course of the study. There should really be close monitoring, in case of adverse events, the responsible people in the health sector should follow up on a client. For instance: we have an ART regimen which may cause a client to experience some problems whenever they take the medication but once they are given a certain treatment, they feel better. For example in South Africa, whenever an individual is on ART, she is also given some vitamins to keep them health. This is the reason why most people in South Africa who are on ART do not experience any health problems compared to most Malawians on ART. In Malawi people are given ARVs alongside Bactrim without vitamin supplements. Our leaders should therefore look into this matter, because those people on ARVs are human beings too. These leaders should ensure there is care for such people too

**PM06:**	just to add on this, may I know something regarding people who undergo HIV testing. Of course, it is a fact that people have some underlying conditions, what if this study participant experiences a health problem, what should they do to get help because it may happen that this person can fall ill for two weeks and then fights for herself to get treatment, what have you done to make it easier for such people to access medical help in time?

**Interviewer:**	can we tackle that question at the end of the discussion, because this is another section we wish to discuss. So allow me to answer this question at the end of the discussion. Let me now asked this question: who should represent a participant’s guardian during the study, a person that the research team will contact in order to reach me?

**PM06:**	I think this will all depend on who volunteers to be the participant’s guardian; someone the research team should contact to reach the participants, because there are some people who are married and are in good terms with their spouses, these people should automatically be the participants’ guardians while there are some people who have a very good relationship with their older siblings, those can take their older siblings as their guardians. Some people can ask their parents to be their guardians during the study

**PF05:**	I had the same response

**Interviewer:**	alright. You have said that this will all depend on the participants’ choice, for those participants that may wish their older siblings, spouses or parents to be their guardians, should we take it to say a guardian should only be someone staying close by or should be anyone even those living far away?

**PF04:**	this person should either be staying close by or far away, it will all depend on who the participant chooses to be their guardian whenever they enroll in a study. It should not be something a participant should hold for herself, but they should have a confidant who they should ask to be their guardians, whether they live far or not, someone who can allow you to give out their contact numbers for you to be reached

**PF05:**	the best person can be someone you live close to, because this person differs from someone living far from you. The person who lives far from you has disadvantages including: having phones off due to not having them fully charged and only have them charged after 3 days or so. As a participant you could experience an emergency and may need a guardian to be close by. In the case of a guardian living far away, it will be difficult for the participant to be reached. Therefore, the ideal guardian could be one who staying close by. This guardian should either be a participant’s spouse or a child; people you can tell about your enrollment in a study and they will be called by a research team to ask about my well being

**Interviewer:**	alright,	what advantages can be there when we conduct this TB challenge research study in health people?

**PM01:**	I think the main advantage that can be there is that: we can save more lives. Because I never thought of getting infected with TB in my whole life, all I thought was that: TB only infected people who were reckless with their lives; people who smoked cigarettes. When I went to the hospital, the doctors told me they had diagnosed me with TB and I was required to start treatment. At first I protested a lot and even went as far as protesting more when I was asked to undergo a TB test, because I did not expect to get TB positive result. But after this, I realized that every person was vulnerable and at risk of suffering from TB. However, in most cases, whenever TB was being discussed, people thought this was something that only affected a specific group of people just like leprosy. People look at such victims as people who applied to be in such conditions which is not the fact. By conducting research studies like this one, you would be helping a lot of people. We have children, women and other people who suffer a lot because of such diseases. Therefore, this will help us participate to save more lives. People should understand the importance of going to the hospital for medical checkup, because pneumonia is very dangerous. I have ever suffered from pneumonia which made me difficult for me to lay down or sit up. I was always on oxygen. Therefore, my conclusion from my experience with pneumonia is that: this is a very serious condition and if people are assisted, most of the existing health conditions will be reduced, as I believe that most people who die of pneumonia die as a result of a compromised immune system. So this will be a very good step because we will participate to fight this whole issue

**Interviewer:**	alright, which other benefit can come as a result of conducting a TB challenge study?

**PF02:**	this means: the prevalence rate of this germ will be reduced compared to how it is today. Because if a vaccine is established and effective, then most lives will be saved because once people get vaccinated their lives will be safe from infections. However, people will be eager to join the study when they get proof of the vaccine being effective

**Interviewer:**	which other benefit will you get from the existence of this research study?

**PF03:**	just to add on what has been explained: this will help in preventing the spread of the disease because if someone survives the disease, definitely other people will be saved from contracting the disease. In the past, TB had signs and symptoms and people could easily tell that a person had TB just by looking at the signs someone showed which not the case is today. Someone can look health and may be asked to get a TB test after experiencing a prolonged cough, the individual would refuse to undergo the test just because they look health. The other issue is that: the injection may not be effective because after undergoing a test, the individual would be diagnosed with two conditions including TB and HIV. This is common nowadays, whereby a person can be diagnosed with HIV today and the next day is diagnosed with TB

**PM06:**	this shows that TB attacks someone with low immune system.

**All:**	it is during the suppression of the immune system when the body is attacked by a number of diseases. TB is a very big concern globally, because once a member of a household contracts TB, definitely all the members of the household will be at risk of contracting the disease. This study will help the disease to stop spreading because once an individual receives treatment they will protect the rest from contracting TB.

**PF04:**	I have heard that every person has TB but it all depends on how strong their immune system is for the TB infection to come out. Additionally, whenever the BCG is being given to someone already having the TB bug, how will this work, will this make the bug to be less active?

**Interviewer:**	the way I have said it here, TB vaccine is given to health people and not people who are already TB positive because this is basically given to people who are health and not in people who already have TB. Trying the vaccine in a health person will help in the effective process of checking for the presence of the BCG in the body.

**PF03:**	the question is: a person who has not taken BCG has a suppressed immune system, therefore how will BCG help an individual once they get vaccinated?

**PM06:**	it means it will protect people

**PF04:**	I don’t mean this will protect them. It is just unfortunate that in Malawi we only know that vaccines are only given to children,

**PF05:**	I have also heard from the hospital that every person has TB, it just depends on how strong the immune system is, before the bug presents in the body. I think that should be the question we should be answering

**Interviewer:**	what I can say on this matter: do you remember I said something about giving someone bacteria in order to perform a trial. Two people can be given the bacteria, the other person can show some signs like fever, cough and flu while the other person can show no signs at all and may seem health. The same thing happens to our bodies as people have deferent ways their immune system responds to vaccines. Someone can be sick while the other person cannot fall sick at all. In this case, every participant is taken to be health and what the research team wants to know is: what will happen when people are given BCG, for those that are going to fall sick, their signs and symptoms will be recorded. For those that are going to be health, they will be screened to check for the presence of BCG.

**PM06:**	just to add on this: diseases come when an individual’s immune system is compromised. For example: you can have a partner who may be HIV positive while their spouse may test negative. A man can be HIV negative while the wife can be HIV positive. In true sense, this being a couple, they are involved in intimate activities that may expose the other person to HIV. Once the body of a positive partner becomes weak, disease attack their body this may indicate that their immune system is suppressed. It is a fact that every person has TB but if the bacteria doesn’t bring any problem to the body, it means: they cannot be sick. For instance: you can be bitten by a mosquito but cannot be diagnosed with malaria, this doesn’t mean you should not take antimalarial drugs. The same with the vaccine, it doesn’t mean the bug just enters the body anyhow but rather through a controlled measure. The bacteria is given as a vaccine and doesn’t enter the body anyhow

**PF05:**	such bacteria has its own way of treatment whenever an adverse event occurs in a participant.

**Interviewer:**	alright, what do you think can be the risks of conducting this type of study; the TB challenge study we are talking about here, in health people?

**PF03:**	we cannot instantly tell of the risks that can be present when conducting the TB challenge study because this vaccine has not yet started being administered. Therefore, we cannot conclude what risks will be there when conducting this type of study

**PM06:**	just to add on that: every vaccine has its own side effects, but all these side effects should be controlled in order to work effectively. If we take it with a negative perspective that says: “vaccines are not good,” by today, we would still be having smallpox and polio, but researchers worked hard to get a solution. Risks will be there since drugs remain to be drugs. However, the risk can be minimal compared to the risks of TB, because once this attacks the body, the effects will be so risky.

**PF05:**	risks can be there, but the severity will all depend on how an individual’s body responds to treatment, because some drugs have side effects when consumed while others don’t even show any side effects when consumed. In your case, you have said that participants will be monitored, this is when you can observe the side effects like: Redding of eyes or foam. As of now, we cannot tell of the risks of conducting this type of study because we don’t know the side effects of the vaccine. This is just the same experience we had during COVID, people said: “once someone got vaccinated,” they will have paralyzed limbs. Some people complained of headache, I guess that is how this vaccine will work. The side effects will be observed during the study, maybe all participants will develop sores after getting the vaccine, and all this will be experienced as the study progresses

**PM06:**	in addition to that: you need to specify who will be affected by those risks; be it the people who will get vaccinated or?

**Interviewer:**	in this case, I mean people who will be vaccinated; people who will be in the trial

**PM01:**	of course the side effects will be there but we don’t know them yet. However, we just need to expect risks in this study. The issue here is all about the immune response. The immune response depends on the blood group of an individual. Some people respond well to vaccines while others don’t. Therefore, the risks will be there.

**Interviewer:**	let us now come to the part of the study that talks about activities in the TB challenge study where participants are given BCG. As part of the study, participants will get a BCG vaccine as a way of giving them a bacteria. This BCG is the same vaccine given to children in Malawi in the vaccine program. However, this one will be given in different doses, meaning to say: it will be given in different doses. After the vaccine is administered, a sample of flesh will be taken from the injection site to check for the presence of BCG. Additionally, other samples will be collected to check for other things or check for the presence of BCG in the body. What is your opinion on this idea of administering this BCG vaccine as a way of giving bacteria to participants?

**PF04:**	before we get to the end of the discussions, will the sample of flesh from the injection site be taken immediately after the injection or how long will they wait for, to come and collect the sample?

**Interviewer:**	it will take a specific period of time after the injection, before the sample is collected from the injection site. It will not be done right away. I asked a question on your opinion about administering BCG, as a way of giving bacteria to participants in form of BCG?

**PF05:**	the BCG being administered is given as a trial because from my understanding, every vaccine means putting bugs in one’s body. Because you cannot just say: ‘you are suffering from malaria’ but researchers should catch the mosquitoes and check for the presence of plasmodium

**PM06:**	once they use the present vaccine; the BCG is not new to the public, it will be easy for the public to accept it because it is not new. This will make things easier compared to using a vaccine that is completely new to the public. However, the fact that this vaccine is already common doesn’t matter but what matters is: “is it possible to have an adult vaccinated and be protected from infections?” because with time; if vaccines are given to children and adults or people who we feel are at risk of infections, we can give them that to help them get safe from contracting infections. I feel this is a good way to go. On the other hand, the name of the vaccine will matter because if we were to explain to people a new vaccine name, it would take us time to have the people understand it. Additionally, let me request this to be discussed at the end of the interview

**Interviewer:**	alright, which part of the body do you think the BCG should be injected?

**PF05:**	you mean on the body of a person or?

**Interviewer:**	of course on a person’s body; in this case I mean the place on an individual’s body that would be ideal to have the BCG vaccine injected?

**PM06:**	I think the ideal place to have the BCG injected should be the upper arm just the way we do it when giving the BCG to a baby

**Interviewer:**	right on the upper arm, what do others say?

**PF02:**	I think the best site would be the thigh to make it easier when collecting a sample of flesh, since the thigh normally has more flesh and will not take time to heal.

**PM06:**	The thigh and buttocks would be ideal because it has more flesh

**PF05:**	you said that after injecting the site, they will collect a sample of flesh?

**Interviewer:**	yes, the sample of the flesh will be very small

**PF05:**	will this sample be collected from the injection site or?

**Interviewer:**	the sample of flesh is required to be collected from the injection site

**PF05:**	if that is the case, then the buttocks would be an ideal place to inject because this part has more flesh compared to any other part

**PF04:**	I think it would be ideal to inject the upper arm, because the upper arm is the best place because after the sample of flesh is collect, it will heal fast since fresh air passes through and there is more movement around this part which restricts rheumatism from occurring compared to the buttocks site which may be bruised whenever you sit down. Therefore, I suggest the site should be the upper arm, everyone should have a preference of where they feel would be the ideal site for the injection. The researchers will give us both the advantages and disadvantages of injecting those sites

**Interviewer:**	what is your view on the issue regarding collecting a sample of flesh from the BCG injection site?

**PF04:**	I think someone should be told that a sample of flesh will be collected from the injection site in time instead of letting one know about that when they want to collect the sample

**Interviewer:**	alright, what do others say?

**PM01:**	we need to be very careful on that part because nowadays, most people believe that some people do business from collecting samples from individuals’ bodies. Before this process, participants should be told about the procedure earlier. This should not just be done anyhow, there is need for an explanation of the procedure in advance, and the procedure will require an expert to inform a patient. Because I remember during my experience, I it was hard to have TB diagnosed in me but I had to undergo a lumber puncture procedure. I resisted to have a lumber puncture because of the negative things I heard about lumber puncture. With this issue of taking a sample of flesh, I feel people will also bring in negative ideas like: ‘people are taking flesh sample for their unknown reasons.’ Therefore, this should be handled with care because we cannot avoid negative speculations about this

**PF05:**	let me ask something: kindly repeat the question that the rest of the participants are responding to

**Interviewer:**	my question was: “what is your view on the issue of taking a sample of flesh from the injection site and have it tested?”

**PM06:**	I think if we compare all the vaccines that have been administered before, a sample is not taken immediately after giving a person a vaccine. I think the whole procedure should be explained before the participant is involved in the study. This applies to this issue of taking a sample of flesh from the injection site, there will be some conflicts if proper explanation is not given on the matter. People should be informed about what the study will involve earlier. Participants should be told that: samples will be tested and the reason for taking a sample of flesh should be explained in detail

**PM01:**	it will be hard for an ordinary person to understand

**Interviewer:**	when giving an explanation in the first place; I said that a sample of flesh will be taken from the injection site to check for the presence of BCG. On the other hand, other samples will be collected; this shows that the blood sample that is being mentioned here will be collected to be tested separately to check for the presence of BCG. This is where types of samples are being mentioned; “samples taken from the injection site.” In this case, what can be the best explanation for the sample of flesh that will collected as mentioned here, to the participants?

**PM06:**	in my opinion, what people really need to understand more is: the sample will be taken from the injection site and not anywhere else. The explanation to participants should come from there; both flesh and blood samples will come from the injection site that is what the participant should be told

**PF04:**	will the samples be taken from only those taking part in the study or they will be taken from anyone who decides to take the BCG?

**Interviewer:**	this will only concern people participating in the study

**PF05:**	this will involve those who consent to participate

**Interviewer:**	this will involve only those who consent to participate

**PF05:**	it will involve those who consent to participate in the study, because when someone consents to participate, it means they have understood the purpose of the study and they have volunteered to participate in the study

**Interviewer:**	there are different ways of administering BCG vaccine; one of these ways is putting the BCG in the lungs. This method uses a pipe that is placed in the nose or mouth through the throat to the lungs. This method is also used to collect samples from the lungs. What are your views on this type of method?

**PF04:**	this method may be good if there is no pain involved because I think pain is involved because it is not easy for a pipe to be passed through the nose and mouth to the lungs.

**PF02:**	most of us don’t know that lungs also get infections. Additionally, we don’t even know that some people have pipes inserted in their lungs to collect samples, this is a dangerous procedure to experience, but it may help us understand whether our lungs are infected by certain diseases….

**PM06:	…….**(number **PF:02** interrupts)

**PF02:**	do you understand my statement?

**PM06:**	please repeat

**PF02:**	I am saying: this kind of sample collection can be good because most people don’t know that lungs also get infections because we are only used to having samples taken from us for malaria screening. Lungs also get infections, that procedure is good but the concern is on how the procedure is done; pipes are inserted in the mouth through the throat to the lungs, but this is however a good procedure because it aims at taking samples that are screened to check for the presence of infections

**PM06:**	in most cases, such procedures seem bad because we just discuss them but if for instance we have a relative who has undergone this procedure before, at Queens. This procedure may seem dangerous if presented by word of mouth but for someone who is sick, they have no option but to undergo the procedure in order to get assistance. This procedure involves having a pipe inserted in the mouth through the throat to the lungs, the patient just agrees to have the procedure done on them without hesitation provided there are no other risks aligned to this procedure, as long as the procedure helps the patient, because this is what patients basically go through whenever they go to the hospital with different conditions

**Interviewer:**	alright, if this procedure is explained to people, people agree to undergo such procedure as part of this study?

**PF05:**	I think people cannot agree to undergo such a procedure, because this sounds to be a very dangerous one, this is more like the procedure used to collect COVID samples, with this type of procedure, the swab is inserted at the back of the throat, from my experience, this was painful. If this was painful, how about inserting a pipe in the mouth or nose through the throat to the lungs? To be honest with you, I feel people will not agree with this type of procedure.

**PM06:**	once they hear that the procedure is about inserting a pipe in the nose and goes through the throat to the lungs, they will not agree to undergo such a procedure,

**PF05:**	unless otherwise.

**PM01:**	do we have other means of collecting samples from the lungs?

**PF05:**	maybe they will give the participant anesthesia to make them insensitive to pain

**Interviewer:**	I have mentioned two methods of giving BCG; the injection one and the other one that involves pipes inserted in the mouth or nose through the throat to the lungs. I would like to hear your views on this method of inserting a pipe in the nose or mouth through the throat to the lungs?

**PF05:**	maybe the injection one would be ideal

**PM01:**	the one that inserts the bacteria directly into the lungs

**PF03:**	the best one is the injection one, because most people even shunned from undergoing a COVID procedure.

**PM01:**	the one using the pipes is a surgical process and requires one to have it done at a central hospital by experts because if done carelessly, the lungs can be damaged…(laughs)

**Interviewer:**	let us come to another issue. How can we feel or what can we tell a friend or relative who tells us they have enrolled in a study like this one?

**PF03:**	I can have nothing to say because they have already made their decision to participate, that is their right to decision making. The only thing I would do is encourage the person

**Interviewer:**	alright, what do others say?

**PF02:**	the answer is the same. This individual has already made a decision. The only thing I can do is to tell them the types of methods used to put bacteria in the body so that she should have a decision of which method to use, whether to continue with the procedure or not. But if they may wish to participate, they are free to do so

**PF04:**	let me add on what she has said: this is all a decision they have made. You can advise them not to participate, but once they make a decision to participate in the study, they will proceed. For example: when I got a phone call informing me of having been diagnosed with TB and should start treatment, I had a sister close by, she told me not to start the treatment, she told me that this was a lie; “I was okay,” according to how health I looked then. However, on my own, I decided to leave my home to the hospital without informing anyone to the point whereby I got my treatment. My relatives thought that maybe something was going to happen to me if I got treatment then I decided not to inform them earlier

**PM06:**	unfortunately, people are afraid of something they already have despite having people around to help them

**Interviewer:**	what concerns can we have after being told by a friend or relative that they got enrolled in a study?

**PM01:**	I think when answering the question, we should answer on behalf of people that side, people who can understand this, because we are better off now because we have an idea of all this. Then, the question should be: how will those people feel…(laughs)

**Interviewer:**	I am asking you this question; how can you feel if a friend or relative told you they were participating in this study?

**PF05:**	concerns can still be there because this is just a trial and we don’t know what we may experience in the process of being given the bacteria. Let us take the scenario of being given a vaccine and we develop an adverse event in the process, if I participate with a younger sister, it means we will both experience whatsoever, that is a risk on its own because we will both experience the problem at the same time, in this case we can develop fear. To avoid this, I may decide to go there as an individual first and see what will follow after consenting to be in the study

**Interviewer:**	let us come on the issue of comments. From the discussions we just had, do you think the study will be successful among people?

**PF04:**	it is all possible provided people are well informed about it, because the way we have understood this thing here, we are able to agree to participate because of the knowledge we have about it. Therefore, people participating in the study should have a clear explanation for them to understand the whole thing. In so doing: the study will be successful.

**Interviewer:**	what comments do we have from the discussions we had this afternoon, I would like to wind up the discussions?

**PF02:**	it is good to have the vaccine because it will keep us health. We will be free from some of these infectious diseases

**Interviewer:**	any other comment?

**PF03:**	I feel this is good because life nowadays relies on vaccines. A research study like this one will ensure that vaccines are developed to combat diseases we have been experiencing in the past. In addition, most people are dying of pneumonia and it would therefore be wise for people to given such bacteria to allow the body develop strong immunity to fight diseases like pneumonia

**Interviewer:**	any other comments, or maybe you have no more comments?

**PF03:**	let us proceed

**Interviewer:**	when we reach the comment stage, it means we are at the end of our discussions this afternoon. Do you have anything you wish to say regarding what we have discussed this afternoon, just anything to comment or disregard from the discussions we just had?

**PF06:**	I think you have said a lot, I would like to understand: for those who are going to participate in the study, will they participate as people employed in the study or the study will work with anyone who shows interest to participate. What measures have you put in place in terms of what will be involved for people who are going to participate. What measures have you used in the selection criteria?

**Interviewer:**	alright, are there any other comments or questions?

**PM01:**	for those of us that are gathered here, I believe there are many people out there who have a condition as we have. What criteria did you use to select us that is my first question? Secondly, for those people that are going to participate in the trial and those of us that will be involved in the main study, what difference is there between us. Does the participation involve us getting the bacteria or we are required to be involved in civic educating people, I would like to have a clear understanding on this part.

**PM06:**	she already responded to that question

**Interviewer:**	I have already responded to that

**PM06:**	the participation will require us to get the bacteria

**PM01:**	you mean we will be the first people to get the bacteria?

**Interviewer:**	your participation will only be: when you get involved in the discussions we just had here

**PM01:**	alright

**Interviewer:**	our discussions are done here and people out there don’t know about this, it doesn’t mean this information should be disseminated to people out there

**All:**	no

**Interviewer:**	the discussion was just to get insight from you

**PM01:**	just the first phase of the whole project?

**Interviewer:**	yes. The participation here is your involvement in the discussions we just had here and nothing else. Your next participation will be in the study once everything about the study is set and approved

**PM01:**	alright

**Interviewer:**	let me wind up so that we go to the other section

**PM01:**	alright

**Interviewer:**	if there is nothing else to say regarding this discussion, may I thank you all for volunteering to participate in the discussions, where you gave in your views. This will be very important and helpful in planning for the study in future once everything is approved and set. Thank you for coming after getting our invitation to attend these discussions. Thank you very much.

The end!!
