# Supplementary material for: Acceptability of advanced Controlled Human Infection Models: The case of a pneumococcal challenge in people living with HIV and mycobacterial challenge with Bacillus Calmette Guerin in Malawi
Source: PLOS Glob Public Health. 2026 Aug 18;6(8):e0006236. doi: 10.1371/journal.pgph.0006236 (PMC13485124; doi:10.1371/journal.pgph.0006236)
Supplement: Supplementary file 1 [file pgph.0006236.s001.zip › Documents/ACHIMA_HEALTH ADULT MALE_11_06.03.24 240306_1424.docx]

**NAME:	FGD**

**SEX:	MALE/FEMALE**

**PROFESSION:	HEALTH ADULT MALE**

**LOCATION:	NOT MENTIONED**

**Video:**	Vaccines prevent diseases and save countless lives. But will it take him 10 years or more to develop new vaccines? We need to find a faster way. One way to speed up the process is by making better use of human infection studies. Volunteers are given a sample of a vaccine under development, followed by a carefully monitored infection. Medical care is always close to hand if volunteers fall ill. But if they stay well, it means the vaccine works and can be made available much earlier. The idea is almost 300 years old, but using today's advanced technology, this type of study is not just scientifically sound, but ethically responsible children, and of course, follow strict rules.

Human infection sites show huge promise in the fight against diseases like typhoid and malaria. But to develop vaccines most effective for people at risk of disease, we need to test them on those most at risk. By expanding human infection sites and targeting them better, the people most at risk of diseases could help develop the vaccines of the future and save millions more lives. It's a given that vaccines prevent diseases and save countless lives. But will it take him 10 years or more to develop new vaccines? We need to find a faster way.

One way to speed up the process is by making better use of human infection studies. Volunteers are given a sample of a vaccine under development, followed by a carefully monitored infection. Medical care is always close at hand if volunteers fall ill. But if they stay well, it means the vaccine works and can be made available much earlier. The idea is almost 300 years old. But using today's advanced technology, this type of study is not just scientifically sound but ethically responsible too. And of course, follows strict rules. Human infection states show huge promise in the fight against diseases like typhoid and malaria. But to develop vaccines most effective for people at risk of disease, we need to test them on those most at risk. By expanding human infection states and targeting them better, the people most at risk of diseases could help develop the vaccines of the future and save millions more lives.

**Interviewer:**	this video shows us that vaccines saves lives and it takes a long time to develop a vaccine. Therefore, as one way of developing vaccines fast, a process like the one in our discussion here has been put in place. Bugs that cause diseases are put in an individual’s body to see how it works. This is one of the methods that can help in discovering a drug or vaccines, in a short period of time rather than taking years just to have a vaccine developed. Our discussions today will be based on your views about this idea, and whether you feel this will be helpful or not, depending on how you understand the idea. I have given each one of you a number to use as your identification code in the interview. You are required to say your number first before giving your views. Feel free to give your views as you are here representing people in your community. Do you feel this research will be effective in our country or this will not be possible?

**PM04:**	I feel this kind of research study can indeed be effective in this country. However, it will need more community sensitization to make people understand it well. I am saying this because I am part of the information dissemination team in my community; I am a social media clerk on both Facebook and WhatsApp. In addition to this, I usually hold other face to face meetings in the community and feels that the community needs to understand this issue very well. I understand this issue very well, such that, I wish to find a way of getting a strong vaccine to help fight diseases mentioned within the discussions. The vaccine in discussion hasn’t yet been developed. In order to develop the vaccine fast, there is need for people to participate in the research study. We will take the information there as it is, but in order to have a positive response from the community, there should be a strong sensitization to clear out the common doubts about research. When part of the sensitization does not come from research studies like this one, the community ends up with negative perceptions towards the study which are spread throughout the community. This results in creating a bad image like: “this research aims at killing a lot of people”. I feel sensitization on this will be essential to make the whole process work effectively.

**Interviewer:**	what do others say?

**PM06:**	I have understood this information very well and what is required of the study. However, looking at the challenges that the country is facing, it will all depend on the people we are going to explain this study to, once they are sensitized on this, they will be confident enough to participate in the study and agree with what they will be required to do in the study, accompanied by a compensation fee. This is due to the economic status of the country that has lead people to consider the financial benefits of participating in the study first. This compensation will encourage most people to enroll in the study.

**Interviewer:**	alright, what do others say?

**PM05:**	let me conquer with my colleague here, it is all possible to have this accomplished, provided there is enough safety verification about the study. The fact that there has been sensitization on this and understood the whole information regarding the study, it will be possible to participate or agree to go ahead with this plan. The challenge is: many people are watching out there. Families and relations interact with individuals that decide to participate in the study, it is easy for these parties to come in and discourage individuals with a willing heart to participate. Though people are sensitized on this matter and have an understanding of the study, most people would be willing to participate. The most important things to be done first, is to give civic education to the public about this. When people understand the main purpose of doing this whole thing, it will be easy to have this program proceed and get supported by a huge number of people.

**Interviewer:**	what do others say?

**PM02:**	let me comment on what my two colleagues have said on the need for giving civic education to the public. If we approach an individual with no clear information about the study, it will be hard to make them understand what the research team is trying to do. There will be need to use the means that will make an individual understand the research team. Of course I have understood what the study is all about and that there will be a relief once the vaccine is established, as it will help the elderly to survive diseases that can be controlled. The most important thing here is civic educate people about the whole study.

**Interviewer:**	the main issue here is offering enough civic education to the public. I would like you to give me your opinions about procedures to follow when approaching the community with the research study information. What do you think would be the better ways you would use to disseminate information to the public, in this case I am referring to ways that would be effective enough and give positive results as expected?

**PM01:**	in this case, we are gathered here to represent the whole village. On the other hand, you can as well request the whole community to attend a meeting you will hold to civic educate people on the research study information. After the community is done with attending the meeting, these individuals will take the information they got from the meeting and share with others. When the research team comes in with the same information later on, they will just be there to give a deep explanation about it. We are only here to represent you in the community and at least give the community a bit of understanding of the whole study to ease your way in.

**Interviewer:**	the study is just at its initial stage. Once everything is set, we would be done with writing and submission of study reports then. When the research study begins you are required to use the methods of conducting study meetings through other people you may have met with to have the study explained to the rest of the people. The research team will come in later to explain clearly about the whole study. Apart from this, which other way are you going to use?

**PM03:**	let me take you to the scenario of my experience on my way to this place: I came across a school that was busy distributing deworming drugs, I understand some parents asked their children not to take the deworming drugs. In this case: if a child was asked to shun from getting vaccination, will she agree with having a bacteria put in her body, how will that be possible?....**(they all laugh),** definitely the parent will not allow their child to participate in that, which proves the whole need to give civic education to the public. If civic education is not given to the public, the whole work on this will be in vain. Civic education will ease the whole process because an individual cannot agree to have a bacteria put in their bodies, they know this is very risky.

**Interviewer:**	alright, what other views do people have?

**PM06:**	the other thing I can add on this is that: when we go back to our respective offices, we should have a targeted audience. This information should not be disseminated anyhow because the encoded information will hardly be decoded correctly due to the audience’s misconception of things and the like. Some people did not go far with education this makes it hard for them to understand things. If we can have technical advisory group (TAG) in communities, comprising people who have a positive view about things, there will be an effective result. I have a technical advisory group (TAG) on my Facebook page and WhatsApp forum, and I am able to manage controlling the whole community. We have a community WhatsApp forum, comprising counsellors, community leaders, religious leaders and research team members. These groups easily understand us fast and can help us easily disseminate information, unlike just disseminating the information anyhow. If information was to be disseminated anyhow, the community will view us as “Satanists”… (all laughing)…. Nowadays everything is said to be satanic when it does not have satisfying information. However, people forget that even the vaccines we are using now, were established after some people sacrificed their time to participate in different research studies that resulted in the development of the vaccines that are currently being used. It is not easy for some people to understand all that. For a research study to work effectively, there is need to have a targeted number of people to participate. I don’t know how many people would volunteer to participate in the study. As a research study from Malawi Liverpool-Wellcome-trust, do you need up to 100 participants? I don’t know how many participants you wish to enroll in the study?

**Interviewer:**	we have not yet reached the stage where I can tell how many participants will be needed to enroll in the study. We are just in the planning stage of conducting this kind of study. This is just the foundation stage where we are trying to find out whether the study will be successful once conducted or not.

**PM02:**	this is all possible, but what is needed most is civic education, as already stated earlier on. For this information to reach out to the community, there is need to inform religious leaders to disseminate the information in their churches, community leaders to disseminate the information to their community members. This applies to the MPs. But the main parties to involve here should be religious and community leaders. These parties pass on the information to the public easily. Religious leaders and community leaders easily understand things, such that: they are in a position to clearly explain the information to the community for a better understanding.

**Interviewer:**	what information have you grasped from the discussions we have just had here?

**PM04:**	the information I feel I have grasped from our discussions is: as an organization, Malawi-Liverpool-Wellcome-program is an organization that is planning to conduct a research study that needs our support. That is what I have grasped from the discussion.

**Interviewer:**	alright. What information have some of us grasped from this discussion?

**PM01:**	the information I have grasped from the discussion is that: Malawi-Liverpool-Wellcome-trust is planning to develop a new vaccine or drug through research.

**Interviewer:**	alright, which information do you find very important from the information you have been given today?

**PM01:**	the information that I find most important here is that: there is a plan to establish a vaccine to be used against this infection, after the study is completed.

**Interviewer:**	what do others say, should we proceed?

**PM05:**	the most important information here is that: for this research study to be successful, there is need for people to volunteer to participate without being forced. Failure to people’s voluntary participation, the study will not be successful

**Interviewer:**	at the look of things, which information do you feel should be included when approaching people, to let them know about this research study or anything regarding this kind of research?

**PM01:**	the information that needs to be added here is: informing the public about the importance of the vaccine and how it is going to be working in the body, because when an individual is suffering from an infection, they get cured from the infection and live longer when the vaccine works effectively in the body. People should be told about the advantage of something new that has been developed. That is the information I feel should be added on that, because people usually look forward to good things unlike bad things. Therefore, telling people about the advantage of something will boost their eagerness to decide participating fast

**Interviewer:**	is there anything else you would like to add on this?

**PM04:**	I feel the idea that a group member mentioned in the first place about safety is true, because 100% of the process you have mentioned to take place in the study when putting the bacteria in the body is so terrifying. Therefore, if there is an assurance for enough safety in the study, for those participating in the study, there will be a high turn up of participants in the study.

**Interviewer:**	what benefit do you expect to have from conducting a pneumococcal research study, which is commonly known as pneumococcal CHIM in people who are HIV positive?

**PM04:**	I feel there is benefit in conducting this type of study because people who are HIV positive are mostly prone to low immune system, and the most common disease they are at risk of is pneumonia. Therefore, once a research study is conducted on these people, I feel it will give an answer to the reason why these people are at risk of suffering from pneumonia and then develop a vaccine for them to fight against pneumonia alongside ART.

**PM02:**	the other benefit is that: when this study becomes successful, people with HIV will be relieved from other infections, leading to a decrease of this population suffering from pneumonia.

**Interviewer:**	which other benefit do you feel will be there from conducting a pneumonia study in people with HIV? Let us go to the issue about risks. What risks do you feel can be present in people with HIV when a pneumonia study is done on them?

**PM04:**	I will begin with pausing a question before proceeding with my response. What measures have been put in place, since people with HIV are categorized in terms of viral load, there are some who have just been diagnosed with HIV while others have lived with HIV for some time. In this case, these two categories have different units of viral load copies. I would like to know what the research team targets in this case or maybe they will consider taking any of the two category?

**Interviewer:**	let me respond to your question this way: because most of the things have not yet been looked into, but people who will be requested to enroll in this study will be health people, this means that their viral load is good, meaning to say: they adhere to ART and everything about their health is in order. That is the procedure I feel is expected to be followed. What kind of risks do you feel will be there when such individuals participate in the study? As I have already highlighted that: the person required to participate in the study is someone who is already on ART and adheres to treatment and their viral load copies are normal and they are not sick?

**PM03:**	the risk that I see in this case is that: volunteer’s response can really be poor because these people are filled with so many anxieties, therefore these people are usually strict with their life styles to avoid contracting bacterial infections. Now that this study requires them to have a bacteria put in their bodies, it will really be hard to convince such people, but with enough information, most people can be willing to participate. Without the information, most people will shun from participating in the study

**Interviewer:**	which other views would you like to give?

**PM05:**	this issue can be hard for people to understand, follow and accept because I feel they will not be in a position to have enough knowledge about it. Previously, I remember that this was something hard for people to come in public and say that they were HIV positive. This was not an easy thing to do. It all took the presence of civic education where people were given knowledge and assurance that: it was not a death sentence to be found HIV positive. People have now become courageous to come in an open and reveal their HIV positive status. All this happened because those people had enough civic education and all the fear they had, is now gone and they trusted the information they were given through civic education. This applies to this same issue we have at hand. We need to be very strict with giving civic education to the public and once the public understand this whole thing, it will be easy for people to come in the open and request to enroll in the study, just because they had enough civic education. Civic education should be given through musicians, a means that will attract the attention of many people to participate. For example: what TNM company does as a company: TNM sometimes takes popular musicians who go round communities with their music on, and once they reach a place of their target, they disseminate the information they have by introducing themselves and their aim for their visit, little by little the public becomes aware of what is happening on the ground.

**PM02:**	let me ask you a question: does this risk go to someone who has received the vaccine or the whole community?

**Interviewer:**	in this case, the risks are viewed on both sides, be it you bringing a risk to someone who has received the bacteria or the other party, but it is both sides, the project as a whole, specifically on how this is happening.

**PM06:**	alright, the other risk is that: after an individual dies mysteriously, after having received a vaccine, the community can go on demonstrations.

**Interviewer:**	are there any other additional risks to that?

**PM03:**	just as this fellow group member has already said, for example I find myself in a situation whereby I get into trouble and you are the person who was present during the civic education, people’s eyes will be set on you when something happens. You will be responsible for whatsoever has transpired because we live in the same community and do a lot of things together and once you are found in a place where such an event is taking place, the community members would associate you with this, and yet this kind of problem just came up coincidentally, as you know that death can occur as a result of different circumstances and this is not the only way. This is the reason why we are saying: if there could be enough civic education, people would know: of course this has happened but even if the deceased did not participate in the study, she would still have died.

**PM02:**	whenever you could appear in public, everyone would look at you and say: you are satanic…(all laugh)

**Interviewer:**	looking at the benefits and risks of the study, what are your views on the commencement of the study?

**PM01:**	in that case, I feel there is nothing that can happen, let us not focus on the risks of something before it happens but we should just accept things the way they are because once this thing is successful, it will assist us all

**Interviewer:**	what else?

**PM04:**	I feel this is a program with good aims. In most cases when something like this comes from organizations like this one, we trust that it is safe.

**Interviewer:**	what else?

**PM05:**	we don’t expect everything to happen in a positive way in every project, people should not put the outcomes first but should think of proceeding further with the projects that an organization has for us, with an aim of benefiting from the project after its completion by having a vaccine established.

**Interviewer:**	some research studies in the CHIM study have taken place in Malawi using a pneumococcus bacteria that causes pneumonia in health people. How can you differentiate those other research studies from this one, in people with HIV?

**PM05:**	can you repeat the question?

**Interviewer:**	I have said that: some of these CHIM studies have happened in very few health people in Malawi, people who are HIV negative. What are your views about this research study being conducted in people who are HIV positive?

**PM04:**	I feel research studies should be done a lot in people with HIV, because people who are health have a strong immune system. In most cases, their response to a drug is usually fast. For example when they have a headache, they just take water, Panadol or some aspirin and then they feel better in a short period of time. But this research study should mostly take place in people who are HIV positive because their immune system is already low. Diseases often attack these people and there is need to develop something to suppress the virus so that they should not be attacked by pneumonia. This research study should mostly be done in people living with HIV.

**Interviewer:**	what do other people say, what is your take on having this type of study take place in people who are HIV positive?

**PM05:**	this research study should also take place in people who are HIV positive because we also take these people as human beings. Of course it is a 50/50 thing, between those people who are HIV positive and those that are HIV negative. When a person is diagnosed with HIV, it doesn’t mean they are no longer humans that is not how it happens, those people are just like any other human being. Just as it has already been explained that sometimes, pneumonia or tuberculosis, both the diseases attacks them with force. For them to be protected against infections, they are supposed to participate in research studies like these ones for this individual to accept the research study as well. There is need for these individuals to be given enough information too

**Interviewer:**	how can we describe the risks of this study to the community?

**PM04:**	the risks about this study is that people are not given enough safety to participate in the study. What I mean by safety here is that: for an individual to participate, this person should volunteer to do so, a volunteer is someone who does something voluntarily, because even when an adverse event occurs, they are there to accept the situation. Whenever, this type of research is taking place, there should be someone to give people information about the study, this part should have enough backup. The volunteer is sacrificing her life to save other people’s lives, because even if this person dies, it will give a hint to people about which way they should take to prevent more deaths from occurring. In so doing many lives would be saved. Therefore, that is where the dangers of participating in the study is coming in, to avoid this participant to be a mere volunteer. The participant should be given enough safety so that even when adverse events occur, the bereaved family should at least have something to comfort them.

**PM05:**	the other risk about this research study is that: three quarters of most Malawians did not go far with education, most people in Malawi did not get enough education, and whenever someone could be telling them things like these, it becomes hard for them to understand. By the way, how many people will this type of research need to participate, because it shows that this needs to go beyond, you may find that the news has spread widely and only to have some people disappointed, for example if only a few participants maybe trained and only select a small number of people to use for trials. I believe the trial doesn’t take place among a 100 people. If this can go a mile, it may be followed by risks or disappointment. This part gives you a picture of how many people you may wish to have enrolled in the study, people who may have understood the whole thing. Additionally, if people can volunteer fast, it would be good to approach the participant’s relatives and civic educate them on some things. The research can be effective because people will know the results about the study they participated in.

**Interviewer:**	just as I have already responded: at the moment we haven’t yet known about how many people will be needed to participate in the study, because this is just the first phase of the study. But all your ideas you are giving are clear and helpful.

**PM03:**	in a research study like this one, for example the six of us are given this type of bacteria, all of us cannot be health at the same time, one of us should be found with a problem. People take the highest percentage, for example if 4 people are found with a problem, it means things are not well.

**Interviewer:**	let us proceed. I would like us to discuss issues regarding participant’s interest to participate in the research study among children, women and men. How can you feel to see a man taking part in this type of research study?

**PM03:**	I don’t see any problem to see a man or woman taking part in the study, because the infection that is going to be under study can infect both a man and woman, therefore, I don’t see any problem with that. Therefore, there is no need to choose a person to participate in the study.

**PM04:**	additionally, I see no problem regarding participants in the study. Let me just give a view of what it can be like if a man and woman from the same house was to decide to participate in the study, looking at who is the bread winner between the man and woman, in case something happens within 30 days, as you know that Malawians’ earnings is from hand to mouth. They eat whatever they find available, this means that, if one of the bread winners can go out of the house for three days, there can be a problem.

**Interviewer:**	do you feel there can be any problem between men and women participating in this kind of research study?

**PM02:**	I feel there is no any difference between the two. They are both equal just as it has already been stated by fellow group member because any person can be infected by a disease. For example: HIV can infect any person, TB can infect any person. It doesn’t matter whether they are male or female, they are free to participate regardless their gender.

**Interviewer:**	in our last phase of the research study we conducted previously, it has shown that most men showed interest in participating in this research study compared to women. Why do you feel things happened this way?

**PM04:**	in most cases, the communities we are living in and Malawi as a whole, people have different beliefs. Most of the women that are in the age range suitable to participate in the study are married while the rest of them have specific controls. These are the things that act as a drawback, for instance: let us say this individual refused to participate in the study due to absence of consent from both sides. The husband may agree with her to participate in the study while relatives from her side can disagree with the idea due to misconception that the woman might be involved in a certain research study that is risky for her. This is a challenge in most women compared to men. Men are usually free to make decisions on their own without third party.

**PM03:**	worse still, if the community finds out that the study is going to give the participant a bacteria, this will definitely bring a negative response to the request…(All laugh)

**Interviewer:**	what do others say on the issue regarding having more men participating in a research study like this one?

**PM01:**	it takes a very long time for a woman to reach the stage of decision making compared to men. In addition, a man is a strong creature by nature compared a woman. That is what I can say.

**Interviewer:**	let us proceed. Regarding the phase of the study that is expected to come in future, this research study will work with people aged 18 and above, this will be mostly women and girls who wish to participate in the study. This study will involve women of child bearing age, as part of this research study, women required to participate in this research study are expected to take contraceptives. What are your views regarding this idea?

**PM04:**	in addition to the question we have just answered in regard to women, this is another issue. Let me go back to issues regarding beliefs or religion, you will find that some churches don’t accept their members to go to the hospital to take contraceptives, in this case, this category of women automatically get excluded from participating in the study, there are some women who have no issues with traditional beliefs and religion but mostly issues come in when their husbands don’t give them consent to participate in the study, in the long run, this category of women automatically gets excluded from the study.

**PM01:**	you said that women are required to take contraceptives, because of the perception that: these participant’s wombs gets affected by the activities of the research, therefore, this is why they are asked to take contraceptives to prevent the womb from getting damaged. That is what I had to say.

**Interviewer:**	what do others say? Let me come to the issue about beliefs and religion. People have different types of contraceptives depending on their community’s beliefs, we also have other types of contraceptives that women use. In this case, the study requires women to take contraceptives they normally use. The aim of doing this is to ensure that the participating woman should not fall pregnant during the progress of the study as this study will be dealing with women of child bearing age; 18 years and above. Some of the participating women are married while others are not, the aim of requesting the participating women to take contraceptives during the study is to ensure the study is effective. Are there any more ideas aside what has already been said regarding women participating in the study?

**PM03:**	if you feel there is a problem with having a pregnant woman participate in this study, you have decided that it would be a good idea for all participating women in the study to take contraceptives and if you feel there is no problem with that, there will be no problem with allowing them to participate anyhow.

**Interviewer:**	let us proceed with the other phase. I said something on people participating in the study saying: they are requested to stay within premises of the research site in order to be monitored well, I would like to know what your views are, regarding people who have agreed to take part in this study at a specified place for monitoring purposes?

**PM04:**	I feel there is no problem with that, because when an individual reaches the stage of volunteering to participate in something, it means they have understood the whole idea about the thing. Therefore, it is a part of the study activities to allow participants to stay at the study site. This shows there is no problem with that

**PM06:**	I feel there is a problem with hosting participants at the study site for three days, because you have said: these participants will be allowed to leave the camp and visit their respective homes at some point of time which may bring about some challenges because the participants can face different problems while at home which may hinder them from going back to the camp site. In so doing, this may affect their participation in the study. It would be good if you kept them in the camp for the whole three days without allowing them to leave the camp, up until the study is over.

**Interviewer:**	so you mean the participants should stay in the camp throughout the study period without leaving the study site?

**PM06:**	yes

**Interviewer:**	alright, what do others say in regard to this whole study plan? Do you think this study plan is worth enough to be followed by other future research studies similar to this one?

**PM04:**	I feel this is just a good procedure to take. Going further with the whole procedure will all depend on the length of the research study period. We will have some study evaluation. Of course there will be short falls, but I feel this procedure is just good and there is need to put back in shape the areas that need shaping. All in all this is something good to take.

**Interviewer:**	for someone planning to enroll in the study, who do you think they should chose for a voluntary guardian?

**PM02:**	I feel anyone can be a voluntary guardian, for example the chief can chose to volunteer to stand up as a participant’s guardian. On the other hand, the participant’s parents or spouse can as well stand up as a guardian.

**Interviewer:**	you have said: a guardian should be any person: a spouse, parents and other people. From what you have mentioned here, note that: we live in different communities. For instance: I can be staying here with my parents living somewhere else. In this case: who will I approach to stand up as a guardian for me? Because the research study team may wish to communicate something to me and I may not be available, therefore, the research team can contact this guardian to reach out to me as a participant of the study.

**PM04:**	this research study is in two categories. We have a category of health people who are HIV negative and we have another category of health people who are HIV positive, but now when we take health people who are HIV positive, these people normally have a confidant; a person they usually share sensitive information with. This confidant can act as a guardian for this category of participants while the category of health participants; participants who are HIV negative can choose anyone they feel comfortable with, to act as their guardian. Such people can either be their friend or relatives. In the case of spouses; these usually choose their partners to stand up as guardians for each other if they feel comfortable to do so.

**Interviewer:**	do you have any additional comment before we go any further? on the same issue regarding who the participant’s guardian should be, should it be someone from the same house as them or someone they usually socialize with?

**PM06:**	it is just unfortunate that this seems that everyone here came in town to work and relatives are in the village, but the plan should be: a relative should by all means be there as a guardian, because this will be an issue to do with sickness and therefore should not involve someone from outside the family.

**Interviewer:**	should this relative be someone we live close with or it should be someone who lives far away from us, who should we take for a guardian between these two categories of relatives?

**PM06:**	if she is close by or someone we live in the same house with, then she will be there to stand up as a guardian and if we don’t have any relative close by we will just take anyone we trust. For example, there may be some people who may have a very close friend, last week I was in Chilomoni to attend a funeral and the guardian of the deceased was just a mere friend who stood for her just because her relatives had not shown up during her illness. In such circumstances, such people can represent a guardian.

**Interviewer:**	let us move to a section where we will be discussing issues regarding activities in the study. In this case, we will discuss health people who are HIV negative and are being given a TB bacteria as part of the study. Participants will be given BCG as a way of giving them a bacteria. This BCG is just the same as the normal vaccine children receive in a vaccine program in Malawi. This vaccine will be given in different dosses and samples will be taken to check for the presence of BCG. Other samples that will be collected will include flesh that will be tested to check for the presence of BCG. In addition to this, other samples will be taken including blood to check for the presence of BCG in the body. What are your views on the issue regarding putting BCG in the body?

**PM05:**	you have said that a sample of flesh will be taken, does this mean that the skin will be cut?

**Interviewer:**	yes, they will take a piece of flesh for testing. This is usually a small sample of flesh that can hardly be seen by the naked eyes. I would like to hear from your views regarding the issue of injecting the BCG into the body, let us begin from there?

**PM03:**	I feel that the person who is getting the BCG has already agreed to get the BCG, therefore I don’t see any reason for not participating in this thing.

**Interviewer:**	what other views do you have regarding this?

**PM03:**	I feel there is no problem with this, because as an individual, you have accepted that. In addition, you have already said it that: this is not a forcing matter, it is all up to you to withdraw from the study or continue. Therefore, I feel this is good because you have agreed to participate.

**Interviewer:**	let us come to the concern that this one gave regarding taking a sample of flesh for testing. What is your opinion regarding this kind of procedure; where a sample for testing is taken for testing, from the injection site?

**PM02:**	let me just ask a question, what tests will be carried out once this sample of flesh is collected?

**Interviewer:**	it will be taken to the lab for BCG testing from the injection site. Just to check for the presence of BCG on the skin from the collected sample

**PM02:**	let me ask the question this way, this participant has had a sample collected from them, the first thing is that BCG is going to be injected in their body and then after some days, the research team will come back for sample collection, my understanding here is: what will be the first thing to be done in that process?

**Interviewer:**	the first thing will be the BCG injection and after a few days the research team will come for sample collection to check for the presence of BCG. The other samples to be taken will include blood and other samples to check for the presence of BCG. What are your views regarding this study procedure?

**PM05:**	it is really worrisome somehow, for this sample of flesh to be taken because of the rumors that people spread. We have heard rumors about circumcision, there have been a lot of rumors about this. Some people said: after circumcision is done, the foreskin is given to fish. A lot of things have been said about this, I don’t know what measures have been put in place when conducting this kind of study to prevent misconceptions about the study? Why did they suggest taking a sample of flesh?

**Interviewer:**	I would like to hear from your views about what you feel about the misconceptions that are out there?

**PM05:**	my concern is on the issue regarding taking a sample of flesh from the injection site.

**Interviewer:**	which other concern do you have about the misconceptions that are out there?

**PM04:**	let me just comment on this. When volunteers are found to participate in a study, there should be a special sensitization, to avoid participants being taken by surprise when they are enrolled in the study and are given information about what the study will involve. In this case, someone would be assured about how good the progress of the study is. In so doing, the participant will know saying: “I will be in the system up until the study is completed.” Meaning to say: once the research team requests for urine from the participant, they will just give out the sample to the research team without compromise. This should be so to avoid misconceptions. I think the next time when the last person to participate in the study is asked to give samples of something, there will be no problem with doing so, as this will be part of the whole study process.

**Interviewer:**	which part of the body should the BCG be put into the body?

**PM01:**	this depends on what the doctors decide, either to have the BCG placed in the body through the veins in the arm or wherever. We cannot decide which part of the body to place the BCG into the body but doctors have the right to decide since they are professionals….(All laugh)

**PM03:**	it will be done according to the recommended procedure by the health sector. For instance when we go to the hospital: we can give the doctor the signs and symptoms of what we are suffering from but instead, the doctor may request that we get an injection to cure that. Therefore, we will go by the doctor’s decisions as health practitioners.

**Interviewer:**	I just wanted to hear from your opinions about which part of the body you feel is ideal to be pricked in order to get vaccinated using an injection.

**PM04:**	if there can be a choice, I would choose the upper arm instead.

**Interviewer:**	let us proceed. We are still on the issue regarding study activities; activities concerning the BCG vaccine against tuberculosis. We have different ways of administering BCG including: putting the BCG in the lungs. For this method, a pipe is passed from the nose to the mouth through the throat to the lungs. This method is used to take samples from the lungs. What are your views on this type of method?

**PM04:**	can you repeat the question please?

**Interviewer:**	We have different ways of administering BCG including: putting the BCG in the lungs. For this method, a pipe is passed from the nose to the mouth through the throat to the lungs. This method is used to take samples from the lungs. What are your views on this type of method?

**PM05:**	I feel this method is risky. When people go to the hospital, they see different procedures using pipes being done on different people, in the long run, they become frightened because they associate these pipis with with oxygen tubes. Of course the method sounds risky but just as my colleague has already said it that: it all depends on how an individual volunteer accepts this type of method. In this case, as an individual, you may have volunteered to sacrifice your health just to get a solution for a certain health problem

**Interviewer:**	what do others say regarding this type of method?

**PM02:**	aside agreeing with this type of method, will this method not affect those participating in the study? Because there could be a possibility that when this process is used on someone, they may end up being affected somehow. But if this procedure cannot affect an individual participant, then it is all good to take.

**Interviewer:**	number 04, you seem to have something to say?

**PM04:**	I wish to comment on the issue regarding the risky part of this type of method, because just by looking at how this method is done, it shows how risky the whole process is. Apart from the fact that this process is carried out by professionals, this process remains risky. This idea of inserting something in the throat is so risky, we have come across emergencies experienced by health workers at a health facility where we heard that an adverse event occurred due to a clinical apparatus that was forgotten inside patient’s tummy during caesarian section. All in all this is risky. Secondly, even though the procedure is carried out by medical experts, it remains a risk, of course we understand that medical equipment is usually sterilized but due to different handling of these equipment, we cannot be 100% sure that they are fully sterilized, this is where the risk comes about.

**Interviewer:**	which other views do you have to add on what has already been said?

**PM05:**	an individual who has once undergone a COVID test will not agree with this procedure, because we have relatives in the community who have undergone a COVID test before. They still complain up to date of experiencing some abnormalities in the throat ever since they had the procedure. In this case, if such an individual was to be approached to participate in a study requiring such a procedure, they will definitely agree with the procedure now.

**Interviewer:**	what do others say regarding this type of procedure? I would like to learn from you, how would you feel if you came across a relative or friend who enrolled in this type of study?

**PM04:**	it may not be difficult to understand if there is enough civic education regarding this procedure. This would still be based on the same issue regarding sensitization to the people so as to be aware of what the study requires of the participants to prevent them from facing problems. So this will all depend on whether the participant has knowledge about this whole procedure. However, it would be difficult to perform this procedure on someone who doesn’t have any knowledge about the procedure.

**Interviewer:**	what do others say: “a relative informs us about having enrolled in a study like this one”?

**PM01:**	it will all depend on the amount of knowledge you have about that. In addition to this, you may have already been sensitized about it and understood how it works. In this case, you will not be faced with any problem about it. The main thing here will be encouraging this person that’s all.

**Interviewer:**	what do others say: an individual informs us about their enrolment in a study, and are given a bacteria?

**PM01:**	they just need to accept the whole procedure because they will be involved in the study as individuals.

**Interviewer:**	we have come to the end of our discussions, what comment do you have towards the discussions we just had today?

**PM04:**	before we come to the comment section, I have a question.

**Interviewer:**	you can ask the question at the end, let us have the comments first

**PM04:**	let me thank you for conducting this type of study and having sensitization done in the first place. I believe the main aim of the study is to help people and not any other benefits. I believe that the end of all this will bring us a good thing.

**Interviewer:**	are there any other comments or concerns that the group has, please bring them out for us to wind up the discussion.

**PM06:**	this type of study is good and helpful. By conducting the study in the future, we will be done with diseases like tuberculosis. All we need now is to support the whole study process to achieve the predefined goal. On the other hand, we have learnt about things we never knew about.

**Interviewer:**	any other comment you wish to share?

**PM05:**	this research study is good but will require the research team to give enough civic education to the community for better understanding of the whole project. When the community understands the study, it will be easy for participants to enroll in the study without compromise. Therefore, it will all depend on how the rest of the people understand it for the study to be successful.

**PM01:**	this will be a very good project if Malawians accept it. More over the drug that is going to be developed from the study will be a drug that will help the whole nation. If Malawians can unite, support this project, the Malawian population will benefit.

**Interviewer:**	any other comment that the group wish to give?

**PM02:**	this is a good research study and once people understand it, a number of people will participate. The other thing I wish to comment on this is that: I wish the community to be sensitized about who will participate in the study, once the volunteers are found, they should be given civic education about the study. This will ease the whole progress of the study because there are some things that should not be talked about on the stage to prevent them from developing fear

**Interviewer:**	any other comment you wish to give?

**PM04:**	let me agree with number 02, there is some information that needs to be excluded from the study information

**Interviewer:**	what information should be excluded from the study information?

**PM04:**	there is some information that should not be included when study information is disseminated on stage, because some information shuns people from accepting the study

**Interviewer:**	what else?

**PM04:**	that is all

**Interviewer:**	we have come to the end of our discussion, before we end, this team member would like to ask a question, can you go ahead with your question.

**PM04:**	is it allowed for volunteers from the same household to participate in the study; for example a child, mother and father?

**Interviewer:**	participating in the study will depend on how every individual volunteers to participate. We respect individuals’ consent to participate in a study. In this case; once an individual has civic education on the study, they will understand what the study requires of participants. After an individual participant volunteers to participate, they may have shown interest to enroll in the study, in this case, I don’t believe the volunteer will be stopped from participating in the study when they have requirements for the study inclusion criteria. The first thing to consider is to ensure that a participant volunteers to participate and understands all that is supposed to be done and have agreed to participate in the study on voluntary basis.

**PM04:**	the other important question I forgot to ask is: when an individual participates in the study and the whole process is completed; for instance the participant was given a TB bacteria, what will happen when the participant develops tuberculosis a few months of study completion? is there any safety for this particular person in terms of care by the study team or how will this person be assisted in case of an adverse event following receipt of pneumococcal vaccine?

**Interviewer:**	let me answer this way: it doesn’t mean that participants are left out when the study completes, there is always room to accommodate them in case of adverse events

**PM04:**	meaning to say: their data will still be available in the system then?

**Interviewer:**	of course yes

**PM04:**	how long will it take for someone working or running a business to get to the study site?

**Interviewer:**	in this case, I will not be able to tell how many hours this will take, just as it has already been mentioned that: we are in the first phase of the study, where we are just discussing the study with people and write reports. The rest of the study stages will depend on the ideas that we get from study discussions. Once these ideas from different group interviews are combined, we will put all this together and analyse the data and adopt the procedure that will be set to be followed later on. It is hard for me to answer that question because we have not yet reached this other part of the study.

**PM02:**	for those who will volunteer to participate in the study, what have you planned for them? As you understand that people expect something after a study is completed.

**Interviewer:**	when conducting a research study like this one, participants will not have direct benefits because the study results will benefit a lot of people; meaning to say: this will benefit this generation and the forth coming generation. In terms of direct benefits to the participants, this is normally not mentioned. What actually happens is that: during participation in the study, participants are given transport reimbursement from their home to the study site plus a compensation for their time. In short all this is not taken as a benefit, but the main benefit here will come after the study results are out, from the tests that are going to be carried out during the study. Let me wind up, because you seem to have many questions that will require more time. This is the end of the interview, thank you all for your participation. Your ideas are very important in establishing procedures to follow when conducting the type of research study that is expected to take place in future. Thank you so much for participating in this interview.

**The End!!!**
