# Supplementary material for: Acceptability of advanced Controlled Human Infection Models: The case of a pneumococcal challenge in people living with HIV and mycobacterial challenge with Bacillus Calmette Guerin in Malawi
Source: PLOS Glob Public Health. 2026 Aug 18;6(8):e0006236. doi: 10.1371/journal.pgph.0006236 (PMC13485124; doi:10.1371/journal.pgph.0006236)
Supplement: Supplementary file 1 [file pgph.0006236.s001.zip › Documents/ACHIMA_HIV_Provider's_FGD_01_English_14.12.23.docx]

Interviewer:	We have invited you should we really give the bacteria to people from your experiences you have had in the ward, finding people struggling with bacteria the way you look at it because we want to have a vaccine in future. Do you think we should take that risk because we want to hear your views?

Participant 7:	Yes, you can go ahead, maybe there should be a consideration in terms of CD4.

Interviewer:	What do you mean on CD4 in terms of CD4 count?

Participant 7:	Maybe for HIV positives if you could set CD4 cut off point, if CD4 should not go beyond this point in our study, the lowest should be this, and we take like that so that we do not get too many risks.

Interviewer:	Other views? He has started giving his views on what others are saying.

Participant:	Maybe, number 9, I want to add on what he has said in terms of CD4, maybe what he should say people with CD4 their immunity is already low (compromised) so like giving them the bacteria it will be like putting them at risk If we take that these people have high CD4 if we give the bacteria, they will be ok. But I think we should consider viral load of a person is it low or high have added on CD4.

Participant 1:	To add on that part low viral load, I think maybe for viral load we consider this range because for a person who has been o ART for a longtime, they have suppressed load for a longtime and chances to have high viral load mostly it’s very low so maybe if we consider that there might be a good possibility.

Participant 2:	I agree with it, it’s ok if participant has agreed to be inoculated with the bacteria to see how their immunity is going to respond. Because as you said earlier it will be like a contribution to science and we are going to see how they respond and we are also going to reduce other diseases, deaths coming because of the bacteria, this is my opinion/view.

Interviewer:	Ok, another way?

Participant 3:	I would say it is ok, it will be good that participant. I would agree that it will be overall a long term, it will be a good thing but then I would suggest if there can be another way if participants fall sick, access to care should be fast as we know our system sometimes takes long for somebody to access care. I see that there should be a way to track that when they fall sick, we should be able to pick it up. If indeed an adverse event happens due to vaccine, we wouldn’t want that, it will be very bad we should bear the cost.

Participant 4:	I think if they have thought of conducting research, a research will be conducted on risks and benefits on the things that will be taking place in the research corresponding to this research as a matter of looking at risks and benefits analysis. The analysis will look if the risks are more than benefits what will be more between the two will determine whether the research should proceed or not that is what I think.

Interviewer:	There are other additional views.

Participant 6:	Frequency for participants monitoring it should not be participants coming once a month, after introducing the bacteria to them monitoring should be on regular basis maybe weekly because others live far, they can feel they can stay even if they are feeling something due to vaccine they cannot know. They report that when they come that they were feeling this when I was home if they are coming regularly, we will be able to know/pick that this is due to that.

Participant:	To comment on number 7 on what he has said, number 3 to say that we cannot consider CD4 everyone but for those we feel they have very low CD4 we emphasize to them about everything they are going to feel/come across or experience they should report because these are prone to infections if they can get sick with the infection itself so with low CD4 there should be readily available care for them not to deteriorate the case. Readily available care should even include mode of transport for the person to reach the hospital as an emergency.

Interviewer:	This study should be provided by the study or by themselves

Participant:	From the study.

Interviewer:	From the study.

Participant:	Especially those that seem to be on high risk of very low CD4 in my opinion it’s yes.

Interviewer:	Ok, we have shared views/opinions on this issue of conducting this type of research and on how things can turn out in HIV positive people let us continue. I just want to know on information that you have been given in the video and all the other information given related to this type of research. What can you say you have understood or learned.

Participant 3:	Aa mostly that this approach is important to help developing vaccine, so it is a helpful way and in the long run it helps.

Interviewer:	Ok, other thoughts

Participant 5:	It is a thing that will be happening in our country it will help us so it is like, it is taking place in indigenous people it will help indignant people because sometimes we adopt other things different with our nature and other people. When it takes place here it will be acceptable to help others because it can help others

Participant 2:	It will reduce deaths caused by this disease so when the vaccine is introduced will make it possible to say that people who are affected by this disease have well protected immunity when they get sick, they should not be on the risk of premature deaths like preventable deaths.

Participant 6:	It will help maybe to reduce congestion in the hospital, many people will not be admitted as it is the case currently people will have received vaccine people will not get sick as they used.

Interviewer:	Ok, there is other opinions before we move

Participant 5:	Maybe number 5, we are saying that other vaccines are there, but they are donations because government cannot afford to buy. After the research, vaccine will be available easily for government to afford. I don’t know if it will be given for free or what maybe it will be cheaper compared to getting it from abroad as it will be readily available. As it has been said that most people will be able to get that to prevented diseases and to avoid causing other problems

Interviewer:	Ok, of all the information that has been discussed, which message do you feel is the important

Participant 7:	The information/message that is most important the study will mostly help in infections which HIV positive patients suffer most before ART, ART has helped success of the study as said by my friends that infections will be controlled due to the success of the study. Infections will be controlled because of the success of the study so it can be the importance of the study for me I see that as an important point which I have captured.

Participant 8:	The importance that I have noted is after developing vaccine many people can access not only under-five, but all age groups can access the vaccine. When we conduct this study, it will not take long time to develop vaccine it will depend on the success of this study.

Interviewer:	Are there additional thoughts. Just want to know what questions do you have? Do you have questions that have not been answered on what has been discussed on how to conduct this type of study?

Participant1:	Unless if I have missed something after the study has been approved, are we going to have it locally made or it will be manufactured elsewhere.

Interviewer:	So let me explain in this way for vaccine to be developed there is need to have data, to back up on why you need the vaccine. Like for ant malarial to be introduced or Covid vaccine to be introduced there were a lot of people who had Covid, and people never recovered, that was true that there was Covid.

Participant:	All yes

Interviewer:	Ok, what we are doing is we already know that people who are HIV positive are mostly affected with streptococcus, they get very sick from pneumonia maybe you can agree with me, how many people have pneumonia in the ward roughly out of 100 those with streptococcus infections. Streptococcus does not only cause pneumonia, sepsis, meningitis are caused by the same, out of ten how many are HIV positive

Participant:	9 of them

Interviewer:	That means they have been hit hard so we want to have data to back up why we need vaccine, this is the data that we want to collect. This study that we want to do now, we are not giving people vaccine you should understand, we will give them a bacteria the same bacteria causing a lot of people to be in 3B ward. We will give in selected individuals but those HIV positive. You understand without vaccine, wait let me finish our aim is not to get people sick but to see if they can carry the bacteria in their nose and not get sick, why their CD4 is good, viral load is good so it is not everyone who is HIV positive there is a strict criteria that we will use so the question is. You have seen people who are suffering in the ward, do you think it is ok that we should do the study. You understand people are suffering because of the bacteria for the future to develop vaccine do you think it is ok to do this study, what are your views from HIV provider point of view you know the experiences.

Participant 5:	Sorry to come in number 5, maybe I did not get you he was asking will it be manufactured here.

Interviewer:	Here in Malawi the Vaccine

Participant:	Majority yes

Interviewer:	Currently we don’t have the capacity

Participant 8:	The way I understand it, it’s not a matter of vaccine, we are not working on vaccine we are just infecting them with a bacteria.

Interviewer:	Why?

Participant 8:	To see if they can survive or not get sick.

Interviewer:	It’s not about seeing if they can survive or not, we want to get data immunologically on how the body reacts, when we have the bacteria you understand? That is where everything starts, it is like we are building a foundation. If you have a strong foundation for something that goes a long way you understand, are we together number 2 you wanted to say something.

Participant 2:	Yes, you have tackled my question along the way.

Interviewer:	Ok

Participant 3:	I just wanted to ask you, you said this was done outside what was the outcome.

Interviewer:	The outcome was, what was the outcome of CHIM for HIV negative, just throw it at you, abroad not many studies have take place but here we did interviewed some people that friend sitting there dis this in 2019 he invited different people research ethics committee, clinicians, community members asking them if we can conduct a research to give bacteria to people in their nose. The results showed that it is acceptable we did that. So, the main difference now is that we are moving from the group which we can call is not at risk going to the group which we can call they are at a high risk like people living with HIV

Participant 1:	You did not answer, I did not get you answering the question, he asked about the results of the study which was conducted.

Interviewer:	Outside Malawi

Participant:	The one you conducted in Malawi 2019 for HIV negative people, what were the results

Interviewer:	Oho not one like the one we are conducting today.

Participant 1:	Ok I thought you said you did this study in HIV negative now you have to try in HIV positives we want to see the results what the conclusion was.

Interviewer:	(name) you want to add something, no I think it’s an issue after acceptability the main study started, now they want to know is it. Ok we are together now. A similar study to the one we are conducting now was conducted and the results are what I was explaining that people saw that it is acceptable to do that. From there it’s when we started conducting a study where we were giving people a bacteria at the clinic. We invited 24 people infected with the bacteria to observe the bacteria that we gave them after two days we get a sample from the same nose to test at laboratory. The results we were able to get the sample, at the laboratory we were growing the bacteria, and checking body immunity how it is changing. But mainly what we were looking at safety to conduct this study. No one got sick up to the point of admission or serious illness for days, most of the time people were just saying headache or flue for these people medication that was needed was like panado but no other types of drugs because they are sick.

So, they have explained other things related to the research that has been conducted and the study results, we can continue with the discussion. Just want to know on the explanations given, which other message/information do you feel is important that must be explained for you to decide to participate in CHIM type of studies.

Participant 9:	To reach the point of conducting a research it shows you went on the ground you got data that people are suffering from this disease, so I have two questions lets take for example in 100 people how many people suffered from the disease, how many were positive and how many can suffer from the disease overall, the other question will come along the way, I have three questions.

Interviewer:	Let’s say let us reserve the questions to the end of the discussion because we will be preempting what we want to discuss because when they explain it will be like having responses, if we can keep some of the questions towards the end. First, we want to hear your views on the way you look at this system, considering the care that you provide people with HIV. We can continue, I asked a question on what additional message/information do you feel is missing that you need to have it to decide joining the study, here we are talking of decision to join this type of study. What additional information that we need to provide of all the information/message that has been provided related to this research, no response.

Participant 9:	If we are all quiet it means all important information/message has been provided.

Participants:	All participants laughed

Interviewer:	Number 6 you wanted to say something.

Participant 2:	The other message/information that I will want to know if I accept to participate in the study, they explained clearly saying that people were not getting really sick they were complaining about headache and flu. The other question that I want to know is that apart from suffering from headache and flu there are any other risks that I can suffer from any other illnesses apart from headache and flue.

Interviewer:	The question has been quoted, number 7 you raised a hand

Participant 7:	Yes, the other thing I want to know is for these clients that we want to test on them have you considered other conditions like diabetes or other conditions not to be suffering from other infections. For a person joining the study should not be suffering from these diseases, I want to know on that.

Interviewer:	Ok, the question has been quoted you will be answered. We continue to the other section where we want to discuss about benefits or risks that can be there, first thing I want to know on what has been discussed do you feel there is any benefit that can be there in conducting this type of research study giving bacteria to people who are HIV positive, what can be the benefit.

Participant:	No response

Interviewer:	The benefit that can be there doing this type of research study

Participant 4:	I think the benefit is that health providers and scientists will know better what happens with the bacteria when it gets into the body of a person who is HIV positive on what happens. If he/she did not get sick this happens, if he gets sick this happened the benefit that will be there is knowledge, we will know what happens.

Interviewer:	Ok, another benefit

Participant:	The point is the same maybe I should explain in another way, we will have data and we will know when we introduce an infection maybe a lot of people, they have CD4 of this range we will have data to support when vaccine will be developed, we will have basic information for vaccine

Interviewer:	Mmhi, other views on the benefit that can be there, this is the only benefit that can be there

Participant:	Other benefits will follow success of this study

Interviewer:	Benefits like what?

Participant:	Vaccine development, when vaccine is introduced, it will help a lot to stabilize condition of streptococcus pneumonia infections

Interviewer:	Another benefit, here he has mentioned benefit of vaccine when it’s introduced, it will help stabilize conditions, others what we can add on, on what we see as benefit what we see as the benefit that can be there in future

Participant 1:	When vaccine is introduced, it will reduce mortality rate due to streptococcus infection.

Participant 2:	Apart from reducing the mortality rate others will reduce the number of infections and hospitalization that will be taking place in people of this group, and it will reduce congestion in our hospitals due to such conditions.

Participant:	An addition number 7, as we know people who are not sick, they can contribute to development of their life and helping the nation. I believe when vaccine is introduced will help in national development.

Participant:	This will help us to achieve sustainable millennium development on infections as we want to go towards zero infections

Interviewer:	ok, let us come on how you have looked at this issue, what are the risks or challenges that can be there when conducting this research study, giving HIV positive participants bacteria. What are the risks/challenges that can be there?

Participant 8:	It is obvious that these people who will be given these bacteria they can fall sick because it’s people of this group who are very commonly found with pneumonia so if we have introduced the bacteria they can fall sick.

Participant 3:	I think there is no difference with number 8, already this is a risk group so if we introduce to them something that there might be a completely opposite outcome to what we found with HIV negative ones, we can have more serious adverse events that is the biggest risk.

Participant 2:	Am also looking at acceptability to a lay man/ common man someone with low education status I see there can be resistance, I can see the possibility of negative attitude and superstitions why giving them, even giving an example of Covid 19 vaccine few people went to receive the vaccine so I feel with this that is a possible problem that can be there.

Participant 1:	To add on what has just been said there is high chance of mis information that will happen because of giving live bacteria so on that we need to prepare how you are going to manage that to avoid a lot of misconceptions.

Participant 8:	Without prompt and effective treatment after introducing the bacteria it can lead to death that is the risk.

Participant 9:	Just to add others will get sick but the sickness will depend on if it’s due to the vaccine or it is a normal illness/sickness because when you introduce the bacteria they can think beyond, one can get sick yet it is not in line with vaccine so this will happen.

Participant 3:	I think it will also get difficult for someone to separate/isolate for a patient to get sick the confounding factor, I don’t know if they will be able to say for this it happened because it was just natural process or that this is already a risk group that could have even happened without the bacteria, they were supposed to get sick.

Participant:	Am also expecting clients to be taken individual requirements like you want to introduce the infection to me it’s difficult to get food, are you giving supplementary food to me for to get this vaccine, others will demand supplementary foods.

Interviewer:	That side

Participant:	It has already been mentioned

Interviewer:	Ok, let’s continue you have raised your concerns that can be there on the issue of introducing these bacteria in people with HIV. Just want to know from what we have discussed do you feel there is something that can be done to make sure that this type of research to be successful considering the concerns/risks that you have raised here. What should happen to reduce/ minimize or make sure that there is a systematic way for the research to be successful?

Participant 3:	I think that it will be good that the research team delegates particular health care workers who will be there in the event that people fall sick health workers who can be readily available to help them. Because like I said our system takes time so sometimes, we could end up losing people due to delays that happen in the system. If there can be some people who are designated not that when they need help, they should come to the hospital not just coming to the hospital there should be designated care workers to attend to them, I think that will be good if an adverse event has happened.

Participant 6:	I think when recruiting participants, we should be giving them enough information to make sure that they have really accepted to join they should not took their benefits like being provided transport, have they really understood the importance of their participation, for example when they meet with risks getting sick they should be aware they have been informed. They have made the decision voluntarily and they are doing it because they have understood the staff.

Participant 7:	Indeed, to avoid delays in reporting problems that they are facing to extend from number 3, there should be daily phone call, calling them checking their wellbeing from the research team side to identify problems/symptoms early.

Participant 6:	Again

Participant 4:	Just want to emphasize on what number 7 has said phone calls should be from the research team calling participants not participants calling research team today am feeling this no, because you know human beings are human beings one can say am ok had it been I was not feeling well I could have called them. The research team should be calling them how are you if they did not pick a call follow them.

Participant 9:	In addition to what number 2 has said, there is a type of illness that will require participant to get to hospital immediately when recruiting ask them where they are coming from because others come from far areas for them to get to the stage it’s a long distance, others may use 4 motorcycles. You should know where which area they are coming from and is it easy in terms of access to get to the hospital.

Interviewer:	Number 6

Participant:	What I wanted to say is we should look at how we can access participant if is at his/her home, how can we get him, how long can it take to reach out to him

Participant:	I just want to add, I don’t know on the issue of phone calls, me as a female I should be calling females, there are gender-based violence issues. I don’t know if males will be calling females, if it can be females calling fellow females, males calling males it will do good because some people don’t understand others can say you are lying they cannot call you for that that can cause conflicts in the house, as we are saying calling daily so we need make sure a female should get the phone not a man calling a female.

Interviewer:	Ok let’s continue, as explained that this type of research studies has been conducted but in HIV negative people now, we have plans to conduct in people living with HIV to give them these bacteria in their body. How can you differentiate conducting this type of study in HIV positive and HIV negative people, here we want your views on the difference in the two groups of people.

Participant 8:	The difference can be there, maybe conducting a test at the time of starting the study, for people who are HIV negative we can just conduct HIV test and other tests it ends there yet for HIV positive it will require us to do CD4 count, Ram tests and many other tests to determine if it’s safe to introduce bacteria in the person

Participant 2:	The main difference that can I see between these people, people living positively and HIV negative ones, is that HIV positives are on high risk of optimistic infections because their immunity is low compared to HIV negative one that is the main difference is on issue of immunity that others might suffer from other illnesses that come due to low immunity unlike the other group.

Interviewer:	There is another difference

Participant:	No, just to extend from there another difference in this study, should I use the word we should expect more risks than the first study in terms of infections. WE can expect more infections on this in this type of patients that the first of HIV negative, yes, there can be more infections

Interviewer:	Ok, any additional views on what has been said about risks that can be there on these two groups, any additional before we move on. Let’s continue I just want to know in terms of communicating with the people, when explaining about the type of study. What information do you feel we can use to explain to people or inform them about the research process of giving them bacteria?

Participant 8:	Maybe the research team can go to places where HIV positive people are found let’s say we can go to light house, when people gather there and have like a health talk informing them about the research and where they can find you if interested.

Interviewer:	Ok, another way

Participant 4:	To add on point that number 8 has said, I think on where to get you after telling them where they can find you if it can be like same Light house premises because it can be like something else for them to move from light house to go like Lepra side, I don’t think many people can go there unlike the research should be conducted right there.

Interviewer:	Ok, he has talked about one issue on the place where the study is conducted should be at the place where people of this group are present, another on how we can reach out these people. Another way is through which way.

Participant:	Silent

Interviewer:	Your views on how you feel

Participant 4:	In our wards we have people who are reactive we can visit them in our ward one by one as we do daily explain to them that there is this research if you can be interested to join or not.

Interviewer:	Ok.

Participant 6:	Mm, I was thinking of places where they conduct HIV tests let’s take example a person has been tested and found positive despite that the person is anxious, afraid you can explain to him that there is research if he/she can be willing to join study if he can decide.

Interviewer:	Ok.

Participant 2:	I see that they can announce on radio that there this research those interested can join, these days with social media Facebook, twitter and other social groups.

Interviewer:	Ok, still on the same on approach how do we approach people with information, just want to know information on risks about this study how can we explain to people or inform them about risks that can be there participating in this type of study

Participant 5:	The message/ information that we are giving is we are conducting a research study. About risks we must tell them when they come to access to join. We must tell them about risks if we are to talk of risks at the beginning, I tell you few people will come. We should give them information those willing to join will join but to tell them about risks, the risks and the benefits that might be there when they join the study. On benefit I think we can tell them about risks we don’t have to mention unless it’s not difficult. It’s easy for a person to understand but if we scare them, they cannot come.

Participant 1:	I want to differ with this one, I feel like it’s good to inform them about the risk and benefits so that when coming they have made a final decision because if we tell them of benefits you find that from 10, 20 come on the day you are starting when you tell them of the risk you find all gone one remaining so it will be like time and resources waisted

Participant:	Ok, was thinking that because we know the risk when giving information, we should give it along with ways that have been put in place to deal with risks that can happen. Measures that we have put in place that when there is this problem, we will do this we should define these measures that will encourage them. Still more on the other part minor risk we should let them know of the other risk.

Interviewer:	Ok, anyone with additional views.

For those saying there is need to inform people about risks that can be there, people can have questions, which can be the best way to inform people so that they can be comfortable we can say like that.

Participant 7:	Considering that, other risks may happen like maybe suffering from an illness number 7, participant is sick we as research team what should we do when they get sick, how can we quickly help them. So, I want to explain on ways that the research team has put in place when they start getting sick, I was thinking that

Interviewer:	Ok

Participant 1:	I can see that misconceptions happens because maybe we don’t have comprehensive information about the study in the end things just happen in a similar way, but the problem is because of the vaccine. If they already know, they will know that I was informed that this cannot happen because of medication that way we will reduce mis information.

Participant 5:	Maybe

Interviewer:	This one we are not giving vaccine, it’s only the bacteria, I was talking of vaccine or bacteria

Participant:	Oo sorry, yes about bacteria

Interviewer 5:	Ok, the way I was responding it was when giving information I thought we are communicating with them and that we are supposed to tell them about the risk. We are just giving information of course am buying number 7 idea what he was saying that we can tell them about few risks not severe ones if they are there but for the severe ones we have to tell them when they at facility, when they come at facility they come in my room where I will be explaining to people, I have to tell participants everything he/she has to decide not hiding some information but to the public we don’t have to disclose serios risks because people will not come for the study

Interviewer:	Our next question will be going to us on the part of decision making in participating or not participating in this type of research study. If your friend or family member wants to participate in a research study like this, how would you feel about it?

Participant 6:	If he has made the decision, I cannot control what he wants, I cannot tell him not to participate, or take him to so this no. It depends on him, if the owner is willing to participate or he does not want to participate if he has received enough information sometimes people don’t want to participate if we have given them few information, so the issue is does he have enough information about the study and he has accepted to participate.

Participant 7:	Extension of my friend he has the information he is on neutral he asks me should I join the study I can tell him yes, join this is good because he is on neutral, he has understood I can tell him it’s good join.

Interviewer:	Why would you tell him to join?

Participant:	Because I know him taking part in the study at the end, he will benefit in saving lives of many people after the vaccine has been implemented.

Participant 5:	If your family member asks me I can explain my part if I know the staff on my part with what I know because we are saying it’s a study we enroll people from 18 that one is independent he can make a decision on his own not be told, not helping to make a decision or no don’t take part but let him make the decision

Interviewer:	Ok others what are we saying, here we want your views if someone approached you has explained to you about this issue what can you say

Participant 2:	I agreed with the views that others have said, I would respect the decision that a person, my relation has made if he has received all the information, and is well informed of benefits and risk about that

Interviewer:	Mmhi, others

Participant 4:	I agree with the rest of my friends what they have said because a person has reached the point of signing a consent form that I want to join this study it means they have explained the information, what I can ask is if he has understood, yes, I have understood that one must continue I cannot say anything.

Interviewer:	Why would you ask that if he has understood

Participant:	I don't know if he really understood the information, maybe he just signed because of pressure when he saw other people signing it.

Interviewer:	Ok

Participant:	Ok, if a relative approached me that he wants to join a study I will ask, first what he has heard about the study to see if he has understood or not. Will he explain clear information or wrong information. When he explained the information if there is something that I can add or subtract on the information I would do that but if he has decided, I have given him right information, he has to make the decision himself to join or not to join I don’t have a say to join or not.

Interviewer:	So here it shows that the opinion that is being given is that a person should make a decision himself, if you are to take part you will just help with giving other information, there is anything that you can do on the decision that he has made when he reaches out to you

Participant:	Supporting him

Interviewer:	Supporting in what way?

Participant:	He

Interviewer:	In what way?

Participant:	Encouraging him if he has said no will also support that, when he says yes will help him to come on the days he has been told to come, you must come for review, when you have a problem come in good time to the hospital.

Interviewer:	The first time the question was asked people laughed meaning there is other thoughts/views, here we have heard about supporting them on the decision a person has made, we want to hear the other side people were laughing that there might be something

Participant:	Fear

Participant:	Which side of it

Interviewer:	Laughing

Participant:	Laughing can just mean that a person has decided, what should we say

Interviewer:	No, the thought of the decision that he has already made there is a possibility that the first response it’s like it has directed all our responses to go to one side, but I believe there are other people who might have a different opinion/views

Participant:	It is not that people laughed that there are different views no, it was just that we did not expect that you can ask that

Participant:	When he is your relation

Participant 3:	All laughed.

Interviewer:	Number 8, you look like you want to speak

Participant:	No

Participant:	If someone has different opinion /views on that question he/she is free to speak it’s not like someone has been directed because the first one responded in this way all, we must be on that point no

Interviewer:	Ok, what can be your comment on this type of research study, your comment that you may have

Participant 2:	My comment is the main thing it’s very good to explain to the person what is happening, its benefits and the risk it should be to the person to make the decision on his own not coerse or pushing to make the decision. A person has to take it from how he has understood what has been explained.

Interviewer:	Another comment number 1

Participant 1:	just want to support what has said most of the time when doing the procedures, some procedures it’s like we health professionals are forcing them, we already know that there is need for someone to do this, so we give information in a way that we are forcing them. So, when we give information in such away, we are like forcing and then they say they are forcing like they want to benefit something, lets try to provide information which can show like we are forcing people but they should take it as something important or not

Participant 9:	My comment is that. The face that you show client when joining the study should be the same face that you will show them throughout the study, what do I mean, I mean when we are giving the information to join the study, we can show them good face (good behavior) the aim is to recruit them, we give them good information. He has joined the study client is telling you about his problems through phone, not picking calls, cutting the calls as long as he has been recruited. You have to make sure the care which was there at the beginning of the study has to be maintained until the end of the study.

Participant:	Ok another comment is that

Interviewer:	What number?

Participant 7:	Ok, lets take for example most infection vaccine they are attenuated but this one is not attenuated the way I know and how I have understood it. This is real infection, but the infection is not attenuated and there will be infections there should be good planning, quick management of patients when infections happen. On that you have to prepare very well because this one is not attenuated it’s really serious

Interviewer:	Number 6

Participant 6:	What he is saying is what I was thinking that we have to plan/prepare because it might back fire maybe out of 10 people whom we have inoculated them with the bacteria 8 get seriously sick we need to prepare for that .

Interviewer:	Another comment?

Participant:	On the same preparation our patients when they come, they reach AETC not so, at AETC people are on long waiting queue for those who joined study they get sick because of live bacteria that we gave them, when they take long to be assisted that will be a drawback to the patients, if the study team can be doing

Participant:	Special triage

Participant 1:	Yes, for them not to get bored.

Interviewer:	Let’s conclude the discussion that we have had here, any additional comments that you want to share, what are your views on the type of research study

Participant:	The research is good it will not help only one person, it will help many people perhaps the whole country of Malawi however we should not just aim at recruiting people we have to consider the people because the focus will be on these people whom we are conducting the research on, when something happens we will be pointing at them, let’s not just do it as long as it’s possible , but we should consider them as well when they face problem how can we help them

Interviewer:	Another comment

Participant:	Silent

Interviewer:	Silent meaning, we have concluded in Chichewa, ok this is the end of our discussion this afternoon, thank you very much for your dedication to take part in the discussion today. Thank you.

Participant:	Thank you.

Interviewer:	Those who had questions
