# Supplementary material for: Acceptability of advanced Controlled Human Infection Models: The case of a pneumococcal challenge in people living with HIV and mycobacterial challenge with Bacillus Calmette Guerin in Malawi
Source: PLOS Glob Public Health. 2026 Aug 18;6(8):e0006236. doi: 10.1371/journal.pgph.0006236 (PMC13485124; doi:10.1371/journal.pgph.0006236)
Supplement: Supplementary file 1 [file pgph.0006236.s001.zip › Documents/ACHIMA_HSA FGD_03_English_01.02.24.docx]

Interviewer 1:	Welcome this afternoon to our discussion where we are discussing about what is happening in our study. Was explaining that CHIM study or controlled human infection model is a study that gives participants bacteria that cause pneumonia to see how the body reacts. CHIM study is investigating on how the body reacts when a person has been given bacteria, we want your views on how vaccine works controlled human infection model in people with HIV, TB and healthy people in Malawi. We want to know what people know about CHIM, risks that can be there and how people can be affected conducting these types of studies using TB in healthy people those without TB. We want how to know how we can conduct the study safe, your views/opinions that you share will help how we can manage TB CHIM studies and pneumococcus bacteria in people with HIV. What is conducting research, the research is conducted by Malawi Liverpool Wellcome Trust as one part of Kamuzu university of Health Sciences, Wellcome Trust United Kingdom is providing financial support for the research. Kamuzu university of Health Sciences ethics committee is the one who reviewed and approved the study. Who can participate in the study, we want adults from 18 years and above healthy and energetic you are eligible if you speak Chichewa or English, participation is voluntary the information sheet is explaining about the study if you make decision to join the study you will be asked to consent by signing. What can happen when you join study, when you join there will be a discussion interview that will be conducted by nurse or doctor from research, the discussion will be conducted at your convenient time at 3A clinic room. We will record the discussion in a recorder to be able to write we discussed. Benefit of participate in the study there is no direct benefit that can be realised in participating in research apart from being given a chance to give your views about CHIM study for people with HIV and TB in healthy people your views will help CHIM studies that are underway and in future on how they should be conducted here in Malawi.

Risk that can be there, there are no major risks in participating in the study apart from time that can be lost in the discussion that will take about half an hour or an hour. The information that I will share will be confidential everything that you are going to say during the discussion will be kept confidential, what you tell us and what we find out will be kept separately, we will collect personal information about yourself like age, gender and how we can contact you, your name and anything that can identify you will not be used when reporting results. Only the group involved in the study and working at MLW and ethics committee reviewers of research at KUREC they are the ones that can have access on collected information. We will keep information that we have collected in secured computer in Blantyre for not more than 5 Years, written information will be kept in lockable cabinets. How will results of the study be shared, results without personal history will be shared to all people at the end of the study. Results will be shared published during research dissemination meeting in Malawi and other countries, results will be shared to Ministry of health,human rights committee here in Malawi and Malawi Liverpool Wellcome trust. On the part that are you supposed to take part or not in the research it’s your decision if you choose to participate you will be given consent form to keep and we will ask you to sign consent form if you choose not to take part there will be no any changes for the care that you receive at hospital. You are not forced to give any reason for not taking part in the research study. You have the right to change your mind and choose not to participate at any time, if you make decision not to take part, we will continue using information that has been collected unless you tell us not to. Will you be compensated for taking part, you will be compensated for your out-of-pocket expenses for transport you will be given 10,000 if you will be asked to provide additional information on the research you will be compensated with 7,000. If there are problems or concerns you can contact us or KUREC Private Bag 360 check their contact below, who can you contact if you want additional information you can contact us in person or through phone you can talk to Dr Anthony Chirwa he is from Malawi Liverpool Wellcome trust they have written us the contact details and email address, college of medicine Research Ethics their contact details they have given. When we turn page all that we have read has been summarized in 7 points we want to know if you are consenting to take part in the discussion this afternoon.

Participant F:	Have you already started recording, the research has already started.

Interviewer 2:	Ok, different research has been conducted but the one that we intend to conduct is extension to the one that started earlier. We were conducting study where we were giving bacteria causing pneumonia to healthy adults who is not at risk of suffering from any disease. Now we want to extend to people who have HIV we want to see how they react after giving them that bacteria we want to see how their body immunity can protect them. In the end the study this will help us to develop other vaccines in answering your question other research studies have been conducted but this one will be the first of this kind because the group that we will be dealing with is a group that we have not dealt with before, it’s a group that is already at risk they have HIV and other conditions.

Participant F:	Alright

Interviewer 1:	Questions if there are questions or concerns.

Participant F:	Ok, what considerations you say a person should have TB or HIV so which criteria will be used to screen people, some people look to be sick, or this one is at least better, you will base on condition of a person.

Interviewer 2:	Our consideration for this gathering we want you and us to help discussing what are your views on the research to go to these people but before going to them we look at other things.

Participant F:	I feel like you conducted it before in few healthy people, maybe the way it was asked if you can highlight more that its people who are sick to what extent, if they can be categorized in groups those that are sick to what condition or a person who is HIV positive or has TB but is ok he looks physically fit or they are in this condition how are the results coming out or will be in this condition how will the results come out that can help

Interviewer 2:	Thank you very much for our views.

Participant F:	Ok I can add to that saying if you want people who are HIV positive or they are TB related on these people we find there are other people they go for TB test they are found with TB, he is being tested for HIV you find they are HIV positive they have not started treatment, he has started TB treatment what expertise will you use that they will be starting medication will they start with TB medication or both TB and HIV at once or it will be like they will take TB treatment for two weeks after to continue with HIV

Interviewer 2:	Ok, thank you your question will be tackled let me cut you mainly we want you to consent first to have this discussion, we have not started the purpose of this meeting my colleague will help you to consent later we start the discussion.

Interviewer 1:	Alright for those who have black pens these are the ones that we have them for those that don’t have we can use them to write after using we collect them because we are using the same when signing consent. The first bullet when we turn the last page of consent form, we want you to give us consent if you have agreed to participate in the discussion this afternoon are we all together.

Participants:	All yes

Interviewer 1:	At the beginning they are saying I have read information sheet about the study which explains what the study is about and what am expected to do, there is a box at the end if you have agreed to participate you will put your initials for me Charity Gunda in the box I will put CG to show that I agree and have read the information sheet you put initial for your name

Participant F:	I thought we are using numbering.

Interviewer 1:	No numbering is when answering questions when discussing but now it’s signing consent that you agree to participate in the discussion there must be signed evidence that a person agreed to participate the first box you put initials and all the other boxes below you put initials everyone to put initial for his name.

Participant:	Ok, where do we put here?

Interviewer 1:	The last box, we write in all the boxes down first second initials only, will exchange that one.

Participant F:	On number 2 they say have asked all questions that I wanted to ask am happy with the answers given will not sign the other before completing.

Interviewer 1:	Ok, questions what questions you have, that one has asked her question we will answer it at the end because it’s part of the discussion the question that he asked is part of the discussion that we want to do.

Participant M:	Let’s discuss.

Participant F:	You have not answered me, am happy.

Interviewer 2:	Ok.

Participant F:	Just asking maybe you can expand we are short of words of explanations, or we want you to help to take part to see if there is restrictions when we go to the community whether we go to patients what can happen

Interviewer 1:	The main issue we want to hear from you the main issue we need to conduct study this study the procedure is giving bacteria’s we can say bacteria as research procedure in this type of study where people are given bacteria to cause pneumonia our discussion will focus in two parts. WE will ask on TB we will also ask in terms of people with HIV how do you look at it, can you say you are accepting this can help or it will put people at risk.

Participant F:	To cut it short a person.

Video:	It’s a given that vaccines prevent diseases and saves countless lives, but this takes ten years or more to develop new vaccines. We need to find a faster way, one way to speed up the process is by making better use of human infection studies, volunteers are given a sample of vaccine under development followed by a careful monitor infection medical care is always close to hand if volunteers fall ill and if they stay well, it means the vaccine works and can be made available much earlier. The idea is almost 300 years old but using todays advanced technology this type of study is not just scientifically sound but ethically responsible too and of course follows strict rules. Human infection studies show huge promise against fighting diseases like Typhoid and malaria but to develop vaccines most effective at the people at risk we need to test on those at most risk by expanding human infection studies and targeting them better the people most at risk could help develop vaccines better and could save millions more millions of lives.

In the video it’s explaining that it is known that vaccine protect against disease and save lives, but it takes long for vaccine to be developed. As one way to develop vaccine this way of conducting studies giving people bacteria causing different diseases as one way that can help to come up with vaccine faster without taking long without taking many years as it used to be when developing vaccine. This way of giving bacteria causing different diseases is one way that can help to come up with different vaccines faster to start using them among us. That is what the video was saying on what happens for people participating in a study giving bacteria in their body as one way to find a faster way to come up with vaccine. Before we continue there are questions before going into the main discussion we want to start

Participant M:	One question is this happening only in Malawi in Africa let me not say in Malawi maybe it can be taking place in other areas in African. About the research that people are given bacteria so that it helps them to identify vaccine faster is the trial just taking place here only or its worldwide or in other areas it is happening also.

Interviewer 2:	Ok, thank you very much for the question, these studies have been conducted mostly in other countries in UK as explained in the information sheet in Liverpool this has been happening, so we thought our friends have been conducting these studies coming with vaccines without huge risk in people. We thought this can also be done here in Malawi, the thought was that everything we have to wait for our friends the whites on our own we cannot do	because our genetic make-up and them is different, whey they develop it , it works on them it cannot work on us here in Malawi, let us try here in Malawi will our bodies respond well. Answering the question here this is not the first time it has happened in different countries we are just imitating what our friends did for us to see with our genes can it be possible or not.

Interviewer 1:	Another question

Participant M:	Ee I want to ask my question is that, here it shows that this research that is taking place the results we want to come up with vaccine is that so. Now we know vaccine is developed from weakened antigens from another disease. What are we doing with the people are we giving people sample of vaccine or we are just giving people bacteria or the vaccine?

Interviewer 2:	Ok thank you very much CHIM studies involve two things giving people vaccine after giving them bacteria or not giving people vaccine just giving them bacteria to see how their immunity can work in their body without giving vaccine. We had a study where we were testing PCV 13 vaccine, this vaccine most of us we know we give young children we were trying it in adults as we know pneumonia can attack anyone young or old. What we were doing we are take sample of trial vaccine that we are trying out we give a person later the person goes home stay for a month when he come again, we give bacteria we have two groups. The other group we give them real vaccine the other group we give them water as a control we give all of them bacteria to see to see those that receive vaccine will they get sick and those that did not receive. This help us to know if PCV 13 can work in adults because people who got vaccine and received bacteria were not getting sick or showing any signs/symptoms yet those that received only water were showing symptoms of getting sick like flue this helped to know that two things can happen. Sometimes we were giving vaccine, some we just give bacteria that this group receive bacteria let’s say group of HIV negative should receive bacteria HIV people to receive bacteria so that we compare immunity of people with high immunity. How can we do that on those with low immunity the vaccine to compare the difference of immunity of the two groups. I have answered your question.

Participant M:	Ee

Interviewer 2:	Thank you very much.

Participant M:	In case those people that have been given bacteria they have started reacting they are getting sick what plans have you put in place for people to be protected I don’t know what I can say, they are bacteria not so the other people their immunity is already compromised and you introduce bacteria in their body after sometime the body starts reacting to the bacteria he will start getting sick I don’t know what plans have you put in place for people to get healed or for them to be safe or I don’t know how to put it

Interviewer 2:	Ok, thinking of the things that we have introduced there are several plans that we were thinking before recruiting them into the study we first conduct several tests for these we collect blood samples which shows their immunity this one can receive bacteria and not get seriously sick. For example, we have said we enroll people with HIV we can’t just take bacteria and give them, we were thinking maybe we check their CD4 or	how low their viral load is or how high is the viral load, when we look at these things we can continue and give them. After giving them bacteria we watch them closely for example they have received bacteria today we don’t allow them to go home experience in the study that we have been conducting we put them at one place they spent night at Grace Bandawe the first three days we take these days are very critical to us. The aim of putting them up at Grace Bandawe is that if there will be any problems like getting sick during the night there is health providers who can take them to Mwaiwathu we gave them card whey they get sick our staff will take them you to nearest hospital Mwaiwathu where they receive treatment since we started this research no one got seriously ill up to being taken to Mwaiwathu these are the plans we put forward in our discussion you can have other opinions to help us to do it like this

Participant F:	There is another question you have said they spend nights at one place when they go home how long you take following them, communicating with them with the bacteria that you gave them if there can be a problem because of the study.

Interviewer 2:	Ok it depends on the protocol of the study what it is saying like in the other study participant was coming 7 visits others they come up to 9 visits after the 3 days that we are talking about they can come after one week, some after two weeks after finishing his visits the person did not suffer from any illness we can allow him to go. Maybe after a month if they face anything within that month we check if this is due to the study or not, we give help according what has been presented as any other person can suffering from anything like malaria , flue same as anyone else joining a study can suffer from malaria so we don’t rule out that this one is sick due participating in a study unless the symptoms or signs that are shown are like coughing due to bacteria introduced on them recently. Before a person is sent home, we check them in the test of samples that we collect to check on the bacteria given to them to check if the bacteria is still alive in their nose or it has died. If they are going while bacteria have died, we cannot say this one is getting sick due to bacteria that we gave them no, we test on them before they go, we can continue.

Participants:	Yes

Interviewer 1:	Alright as explained in our discussion we want to know what information you have got from the message given related to the study that we want to conduct.

Interviewer 2:	Maybe before tackling the question, we explained that our meeting today we want you to help us on the research study that that we want to conduct, we have conducted research in healthy people. As I have said we want to extend it to another group that we see can be at risk. So, we want to give bacteria to people with HIV to see how protected they will be, how will his immunity fight the bacteria first. Second, we want as you all know TB is a disease that is troubling here in Malawi. TB affects people with HIV we want to see if we can find vaccine that can be given to adults to protect people from TB, in doing this we reduce strength of bacteria give to a person who does not have TB and does not have HIV the one referring to as healthy in all aspects. We give bacteria to this person and see how his immunity responds, how the body fights with the bacteria as I have said when we do that, we follow them up on how the person is responding when there is any problem, we deal with it immediately. Coming to the question what message you have got from the information given. Do you feel this can be done to conduct these two studies that have been mentioned that we can give TB bacteria to a person who does not have any illness can this be done here. Can we take the bacteria give it to people with HIV in short what message have you got from information given to you, is the question clear. In the interest of time, we can start if we delay, we will get late anyone who can open the floor.

Interviewer 1:	When answering the numbers distributed you mention the number 7,2,10 and give your opinion let’s do so.

Interviewer 2:	In general, just say as you have explained I have heard this about the study that you want to conduct depending on your understanding and what we have shared.

Participant 7F:	I feel like trying it in healthy people who don’t have TB can be good and practical a small risk can be there for those with HIV because we don’t know condition of people who you want to have CD4 count of what range, because if he can be with very low CD4 count and give that one we can increase chances of more risk thank you

Interviewer 1:	Thank you, other views.

Participant 9F:	My view I feel testing in a person by giving bacteria to a person who is HIV negative and does not have TB can help and is possible this can help in future to have vaccine to protect from TB to reduce TB cases in future.

Participant 4M:	It will depend on severity/ condition of these people because one can have just contracted it it’s in early stages it’s not yet severe. That one can receive it without problems as it has been said but for those who are critically ill it can be difficult but it’s possible to do this study

Participant 3 F:	For me I don’t know it if a question or comment for a person who will be does not have TB and is HIV negative, we will give them TB bacteria maybe on that time he is HIV negative on window period. I don’t know person has been given bacteria has started to get sick it will not cause other problems like a person getting sick earlier because the person was negative, but he was on window period that he did not know maybe in between receiving bacteria can that cause a person to start getting sick.

Interviewer 2:	Ok so we will take that maybe the questions we address them at the end, will it be possible to do that.

Participants:	Yes

Interviewer 1:	Other views on what you have gathered from information that we provided, others are concerns we are combining all it will go together because we are looking in terms of HIV and TB. Other views on information that you gave collected/gathered information that you have collected on what has been outlined as process for this type of study.

Participant F:	My opinion I will tackle on TB everyone is born with it not, so it depends on strength of body immunity, when immunity is low is when a person get sick. There are other people they were not vaccinated we vaccinate against children at birth against TB not so for a person to be protected they are given BCG. Others we consider them adults maybe some we will find that they did not receive BCG, how will you know if a person is not protected at first and then you continue the other part because first you have to check if a person was vaccinated when he was a child for BCG everyone has a scar so, we have to know for those people who don’t have we should know that these maybe they were not injected or vaccine did not work in their body as intended. In terms of HIV, it will be like a question will you start with testing to check if they are HIV positive or negative or after testing when a person is negative it’s when you continue that the person can accept to join the study. A person can continue with just word of mouth that he has not been tested or you will start testing after testing confirming that he is HIV negative and you are satisfied that a person is negative you don’t know how he as been in the past you have to probe more find out how many people he had sex with and when probe if he has been on PEP or has never been on PEP you need to know how to help him

Interviewer 1:	Alright thank you any other views before we move on information from what we have heard on the information that has been provided.

Participant 3F:	I want to comment, another comment doing the study may be worth it for the reason for other vaccines that were introduced they tried them somewhere they introduce them that is the reason we have many side effects, if vaccines are tested here, it can do good to us.

Interviewer 1:	Alright

Participant 2 F:	Am pleased with what you said that you follow up with them for 3 days to see if there will be any side effects, the people who are involved already have TB others HIV. It depends on person’s body genetic make-up and how the body reacts you look after them for three days we let them go give them a date to come. My opinion that I can give is there should still be a link with study team and where participant stay in case an emergency happens you can communicate so that the person does not suffer too much due to participating in a study that’s my opinion.

Interviewer 1:	Thank you very much Zikomo, other views.

Interviewer 2:	Maybe on the same point what were you thinking how long we can keep following up people when they are in a study to be following on them. We should be linking with them for how long, views just thinking.

Participant F:	I don’t know you said after three days to follow them up for how many weeks.

Interviewer 2:	Yes

Participant F:	Yes for me weeks it’s ok what I was saying is that they can go there the 3 days he is sent home maybe to come after 7 days if anything happens within the 7 days there should be a way that he is comfortable if anything happens I can call them that I have faced with this problem or maybe there is someone close by to me who I can reach out that this happened. Yes, we know that some things are coincidental but still if something happens there should be someone who can help immediately.

Participant 10 F:	To add on the same point, I was thinking mode of communicating if an emergency occurred there should be readily available transportation to pick them from home to hospital, because in some areas are hard people stay on their own without guardian, he joined that study maybe there are only children they cannot pick him to hospital.

Interviewer 1:	Others

Participant 4 M:	Yes, just to add on the same comment I feel like the agreement with participants must be forever because if we do our study today results are out, we are not in touch with the person on the agreed time. After two years side effects appeared on him because of the study he participated two years ago. How will we know there is a need if we have a person in the same community, system in place we can link with them. If he gets sick, there should be a way the relationship has to be forever if he suffers flue or what but if he was in our study, he is ours we should not leave them.

Participant 1 M:	You said when a person has been released after 3 days not so you check if bacteria that were introduced in his body to check if the bacteria are still alive or not, I think I heard like that not so. So maybe security comes to a person on the time we are parting with him that bacteria that were introduced in his body are no more, that you take blood to test to check if bacteria is still there or is no more that is what you mean not so

Interviewer 2:	Yes

Participant 1 M:	Aa ok, alright

Participant 6 F:	A person has come to Grace Bandawe you treated him and have released the person is back in community is it not possible to involve HSA of that community, the purpose is if something happens to participant HSA has to be informed that there is a client at this place they can monitor if there is anything the HSA can rush and communicate with you (study team) if that fails he can go to HSA of the area to help him to go like this to link up with you. Maybe that can be good involving HSA a person in my community taking part we move with them like this, not that the HSA will know everything that he is doing but just involve community HSA just in case something happens he can reach out to them.

Interviewer 1:	Alright, different views/ opinions have been given helpful I believe we can move on

Participants:	All mm

Interviewer 1:	Alright, I want to know which message you think is the most important that should be shared clearly to people when explaining about this type of study.

Participant 2 F:	I feel the study is important say am telling someone about side effects about what is happening, explain to him step by step come out with benefits of the study because when conducting study, we look at benefits of what can happen to a person, but risks to a person we don’t explain them clearly. So please we are involving people some are already sick it is better to explain clearly that is what I see.

Interviewer 1:	Alright she has explained her part others which message we see as most important when explaining about the study.

Participant 1 M:	Yes, I think mien will be a question people enrolled in the study will there be benefits that they can have when they participate in the study that when explaining about study that maybe there will be a package that people will be informed about benefits that when you will be coming to join study your benefit within that period that you will be in the study the benefit will be this. I don’t know if it is like that or not.

Interviewer 1:	Ok alright.

Participant 7 F:	When explaining about vaccine to rural masses explain clearly explain straight to the point because the rural masses when they hear of research study what comes on their mind is that they will get something. When they hear word research, they rush there to receive something the purpose of research study they don’t know. So, when giving the message to communities look closely at the message when people come find out from them purpose of study or they are coming for money.

Interviewer 1:	Mm, alright some additional views.

Participant 8 F:	To add on the same people look for money and because people did not understand about the study, they refuse to join research studies that could have benefited them because of how they understood, what they heard from other people not that they heard it from the ones conducting the study I want to stress on that point tell people the truth they have to join because they have been informed not because they want money. Other people refuse important things that could have helped them it’s important to send strong and correct message.

Interviewer 1:	So, on sending strong and correct message can you share other ways that can be used when giving out correct and strong message guide us on things like what.

Participant 7 F:	When you are about to start this thing sometimes you call village headmen, we have this we want to do when you go back in the community inform people. The village head goes back to the community remain quite has the message been passed no. When you call village headmen plan to meet with people in community the village head explained maybe with a different explanation but maybe they did not pass the message when you go it will be good to other people there to have enough information thank you very much

Interviewer 1:	Thank you, other views, these are the only views that we have on important message.

Participants:	Mm

Interviewer 1:	That’s all I want to know do you have questions that have not been addressed on the plan of this type of study, questions that have not been answered.

Interviewer 2:	Maybe saying that questions have not been answered I believe there were two, three questions before going to the nest section, we help each other answering some of the questions, we know you asked a certain question can you remind me.

Participant:	Maybe let me remember I asked in terms of HIV will you start checking on the status if he is HIV negative

Participant:	For me it was similar the same thing if a person is on window period

Interviewer 2:	Responding to your question when doing a study for those people claiming they are HIV negative we conduct test, when they come at the study site they can tell us that he is negative yet it’s not like that the can bring someone’s health passport book, or a book which they have written somewhere we know sometimes that can happen we have our test kits. Everyone who is negative we conduct test to confirm on our own that it’s true for those that are positive we collect health passport books we want to check if a person has been on medication as well as how long has the person been on treatment. This we will do if we accept this can be done. So, these are the opinions that we thought of we will be taking health passport check viral load range, when he started treatment or is CD4 count high or low as I said I think for a person with low CD4 or viral load which is high should not be enrolled in the study. In responding do we conduct test or not we conduct tests to confirm. For people on window period this was over looked because we thought it is difficult to know this person is on window period or not, we will agree here how to go about on that, what we thought of is to conduct confirmation test but for these on window period if there can be other views on how to go about on this I open the flow how to go about, or you can help with how to handle this issue.

Participant 4 M:	For those on window period it is difficult I don’t know, or I have never heard there is a machine that can be used to detect a person on window period that can be difficult on my side.

Interviewer 1:	Thank you very much.

Participant 3 F:	That is true how can you know I feel like for people who are negative after testing them and have accepted to join study I feel there should be counselling to know more about them for the past days, months who have they slept with maybe just asking for that.

Participant 2 F:	I agree with her as you said that you overlooked it that is true because there are others who are risk group let’s take example of female sex workers we can test on her while on window period or maybe has been promiscuous, you never know who she had slept with. Issue of window period is difficult; I don’t know what others can suggest but it’s a difficult issue.

Participant 8 F:	To add on that people on window it is difficult I think the issue is after testing and are negative that is the result that can be used on that even if they try finding out number of people slept with these people cannot be straight forward to say I have done this with these people, the issue is they can say I did not sleep with anyone, and they take that because they are not carrying out further test the thing is they will use a person is negative because on that there is no evidence that can be there.

Interviewer 1:	Alright we can continue.

Participant F:	Before you continue, I will be like taking you back, you mentioned about issue of TB patients, a person who does not have TB or the one with TB on that can you explain maybe I missed out.

Interviewer 2:	A person we are TB bacteria.

Participant F:	We are back on recruitment criteria are we giving TB bacteria to people with TB already or we are taking those without TB.

Interviewer 2:	Ooh ok we explained two things, we talked about a person who does not have TB, and HIV we give them bacteria for TB, and we talked about a person with HIV Aids we give them pneumococcus bacteria like the one causing pneumonia we were thinking of expanding like that, it is understood there.

Interviewer 1:	We can continue.

Interviewer 2:	To continue with other questions that were asked, there was a question asking how we will know if a person received BCG or not when we go on TB part, for TB anyone can receive even those who received when they were children or those who did not receive. Because what has made us to conduct this study although a person already received TB vaccine we find still more when we get old people are suffering from TB. You can be witnesses some people who have been suffering TB we find them with the scar, but people are getting sick. We were thinking maybe this vaccine has time limit of its effectiveness that is the reason we thought if we can find vaccine that can be effective without any effect for a person who already received when a child was and receiving the second time. So, if we know that you received or not it will not change the plan of conducting the study, I have answered that one. There was another question on what the benefits of the study are, what are we going to tell people. Our thoughts were the study can have different benefits before explaining my views, what do you think can be the benefits if we conduct the study

Participant 2 F:	The main benefit that I see as you said about TB you said people received vaccine but still more, we get sick we hope the vaccine that will be developed will help to reduce numbers of people suffering from TB that is the benefit.

Interviewer 2:	Thank you very much, there are other benefits of the that we see.

Participant 4 M:	Benefit is there to say the one participating in study will have the chance of receiving treatment when he has experienced any problem during the study, maybe he did not have the chance to go to Mwaiwathu the 3 days he was at Grace Bandawe

Interviewer 1:	Another benefit.

Participant 3 F:	Will start like question, after the study has been successful there will be vaccine for HI.

Interviewer 2:	No, that part we did not consider it yet.

Participant 3 F:	On that part of HIV bacteria that they will be given, what will be the benefit.

Interviewer 2:	Bacteria that we are giving is for pneumonia.

Participant 3 F:	Ok I missed.

Participant 2 F:	Just to add on benefit of study that it is being carried here it has not come from outside country it has been tried on people from another place and they give people here, but this is taking place here on us we are the ones to receive this vaccine that is a benefit there.

Interviewer 1:	What can be the benefit if you can expand clearly that the study has been done right here.

Participant 1 M:	To add on what she said for example Covid vaccine was introduced earlier people were refusing that had problems like. Yes, so what she is saying this has been tried on us here in Africa to say it means vaccine that will be developed will match with how our bodies are.

Interviewer 1:	Alright

Participant 3 F:	On that he is saying that they will use like our bodies even our temperatures for keeping vaccine everything will be local from here maybe there will be few side effects as it will depend on our bodies how the body works because when vaccine has been developed from Europe maybe genes there and ours are different when they tell us to receive that vaccine there can be more problems. But for things that have been tried on us that will help in future side effects will be rectified that this is the problem that will be there in future or to enlighten to reduce the risk.

Participant 7F:	In addition I mentioned of giving messages to the community meaning when a community has knowledge on vaccine and research studies that are taking place, when vaccine is introduced people will have already accepted it not be a problem, we will not face problems as the other as explained by my friend if it will be tested on Malawians, when vaccine is developed to be given to Malawians people will accept it.

Participant 6 F:	I want to comment the benefit is more that we are doing this right here in Malawi we conducted BCG study we did that the whole Malawi others were given water others were given real vaccine but that time people were not aware but now children are given vaccine, children that received at first this was done on my child it helped in this way because they did not know that they were given water it is like all are receiving the same thing not knowing one receives something the other another we find that helps vaccine is still there. Because we started that here in Malawi, we see vaccine that can try on two people it can help a lot we can give example we were giving people this and people will say when my child was young as it goes it’s a good thing you cannot bring wrong things to people in the community

Interviewer 1:	Thank you very much for your helpful opinions responding to your question on benefit of the study when explaining to people all that you have explained here are benefits that we will be explaining to people about the study. Just to add this is not a benefit that we emphasize we give something to thank people for what they have used on transport we give them a certain amount of money, but benefits is what we have discussed here.

Participant 1 M:	Thank you that is the benefit I wanted you to explain is this all that you had already explained, the question I asked is this we had explained benefits of vaccine the benefit I wanted mostly is this individual a person has wasted time joining what will be the benefit that’s the benefit I want to know then thank you.

Interviewer 1:	Thank you very much, I want to know on what we discussed on message to explain to people on what we are doing with everything involved in the study, and what additional information people need to be informed before they decide to join this type of study.

Participant 2 F:	What I wanted most is for people to get rid of the impression that he is saying what are going to get because most people join studies because of money. But the purpose of study they don’t know it you will agree that a person can join study, you explain to him but his mindset is not on that when you tell him you will receive this amount of money his for people to know that they are not doing that for money, but to help to come up with vaccine to help mind sets on that I don’t know what can happen switch to that but they are to help contributing to health being of the next future generation . Emphasis must come here because most people if we can go even today that we have come with a study am telling you if you say you will receive money you will have a lot of people. We saw this from a study at Gateway there were a lot of people there was a long queue we hear people were coming from Chilomoni that am going to Gateway because they were getting something.

Interviewer 1:	Here we need to help each other what can we do to deal with the issue of hand puts that are given in a study, what way can be used trying to make sure that people join study because they have understood their role, what they are going to do what can we do.

Participant 4 M:	Let me start first I feel you mentioned how you will explain to people when you go there if you go, and mention benefit you have made a mistake because in English they don’t call it benefit its incentive. Now if you explain as incentive, incentive can be anything like packet of sugar, we want you to have tea here but because time was not on our side take sugar to have tea home that is an incentive. But if we go and say at the end there will be this benefit, if we use English word of benefit it will mess up.

Interviewer 1:	Alright

Participant 9 F:	My opinion as explained by my brother I feel the main thing start explaining about this study, its purpose and you ask the person if he is satisfied with what you have explained if he agrees to join study at that point tell him about the incentive.

Participant 10 F:	To add to that if a person I feel the benefit a person will get it as a surprise.

Interviewer 1:	Here we have explained first we explain purpose of study all study procedures on everything that will happen in the study after explaining has to decide from the other thing to be a surprise.

Participants:	Surprise

Interviewer 1:	We leave it there they should not be told or at what point should they be informed about this.

Participant 9 F:	I feel like after explaining has accepted to join study you explain at the end, we give transport yes.

Participant 3 F:	I feel like surprise can happen to the first group because I have come here you told me as a surprise at first but because I will share with neighbor’s it will no longer be a surprise. I feel the surprise will not be possible people will still know that they are getting something because people will be informing each other there is a study. Maybe if you give a health talk when you go in the community or clinics for those people who will go to your clinic for separate talk with them those will tell their friends it means the surprise is no more. Because when I will be going what will make me not to get the message is that I already got the details, heard of the surprise when I will in in that room, I will be like looking attentively yet I have already heard of the surprise will not understand. But I feel the communication is a bit difficult somewhere just a matter to work on it.

Interviewer 1:	Alright here you want to add.

Participant 8 F:	I want to add what has been said already by colleagues that the surprise will be to the first group the rest of the group it will no longer be a surprise we will come knowing that there is this, but surprise will be to the first group, I feel after getting the first group it will be good.

Participant 5 F:	Whether it’s a question or what when approaching people, it will be reaching them as a group like a village or it will be reaching them one by one.

Interviewer 2:	People joining study.

Participants:	Yes

Interviewer 2:	The ways that we have been using from the beginning of the other studies when we go to health Centre as it is at Gate way after explaining that we have this study we have a room at 3A clinic come. When they come, we start explaining the entire study process how it should be. When others tell each other as it was said as people tell each other that they have heard there is a study we tell them to come on this date to learn about the study. After learning we follow other processes be it they tell us they have accepted to participate and then we proceed with recruitment I have answered.

Participant 6 F:	Mien is a question for a person in the study when you want to give incentive you tell him in advance that we will give you transport or after finishing everything here is your transport to use on your way back.

Interviewer 2:	Aa it depends on what the protocol is saying about the study but here we are planning on how to do the study we will use what you have explained. In previous studies we were explaining to people about based on what we explained this is the purpose of study, benefit is this when people come there, we check them BP if they are eligible, it was found out that some since birth, they haven’t checked their BP they don’t know whether they don’t know how low or high their BP is. That is a benefit on their health life we were explaining that as benefit of participating in a study. Money issue that we explain at the end. On the study information sheet, it states how will we thank you for participating in a study, thanking people not as benefit of study at the end after explaining to them everything that will take place in the study, your views are acceptable how you see we can do it with how others have explained it.

Participant 5 F:	I see this money issue is in the package yes in the process that you have to follow not so, but I feel this should be after you are done with a person it should be as a surprise that you have dealt with the person like thank you very much this is your transport. Not putting it as part of information to explain because when messages will be going to people it will go like we are going to collect money, but it should be like a person has just been thanked, it must be explained right there and when a person is leaving like thanking them. This money is to add on transport not putting it as part of study information.

Participant 7 F:	I can see it on the form they have put a figure a person will read consent form will see I will go with K10000 meaning everything here they will sign to agree to it what he is looking at is K10000, I feel like putting the figure, maybe you can discuss at office but putting the figure people for look for money. Importance of their body and how it helps the study or about side effects that they can face they don’t mind if there is money.

Participant 4 M:	But don’t forget we are in town when people are coming and they ask you this question, you will face this have you prepared an incentive, you will meet with this question they will want to know how much incentive is.

Participant F:	Yes, they will respond that is where to tell them we just arranged transport.

Participant 1 M:	People no matter how it goes people will be given incentive not that there should be another way, issues to do with money are difficult we should not cover it or that people should not be informed this they will know the same as about circumcision people were informed that you will be given money.

Participant M:	If the people will be given something at the end in the program (study) there will be somethings people will experience they can get sick there should be something at the end of the program for people will fall sick they will need to buy food raffs they will need cash that’s fine and understandable not so. People have joined study explain to them because of what we have done you will have certain feelings you may want to have a drink or to buy something to eat or you are feeling weak on issues like those it’s fine.

Participant 3 F:	Let me talk on this issue related to money, it’s not only here in Malawi everywhere my relative went to school abroad there when conducting research to contact a person they were looking for money. So, to say about money the message will be heard about money if we say we don’t tell them at the beginning or they are told because we have said surprise will be to the first ones and it’s not only here everywhere people receive money, consider the package of money.

Participant 4 M:	I just have a request we are in hard times devaluation every now and then it can be possible the package you are planning today can be good but come the time to start implementation of our study money has been devaluate, want to request if you can leave it flexible that you can adjust depending on status of economy.

Interviewer 1:	Alright let’s continue we started discussing about benefits of this study, now I want us to discuss what can be the benefits of conducting this type of study how can you explain that in brief.

Participant 5 F:	Benefits I feel this has been mentioned already that it will benefit to reduce TB cases in future.

Interviewer 1:	What about benefits of conducting the study in people with HIV what could be its benefit.

Participant 2 F:	We know that when a person has HIV, he is prone to other illness not so the lower the immunity we find a person has TB so, if we conduct the study in these people, we will find a way to help the people saying even if they haven HIV we will find a way to help them from TB complication.

Interviewer 1:	Alright another benefit that can be explained.

Participants:	No response

Interviewer 1:	Let’s come on risks that we can see they can be there conducting this type of study, we will start with conducting TB study what are the risks can be there

Participant 10 F:	The risk that can be their level of illness for people you did not mention it, there will be limit or you will get up to which level because there are other people who are critically ill they cannot walk a person like that one will you give bacteria will that not put him on higher risk to finish him

Interviewer 1:	Another risk.

Participant 9 F:	For a person with HIV and has also been diagnosed with TB if you give him pneumonia bacteria, he will not be at risk of becoming seriously ill.

Interviewer 1:	Another risk here we are looking at the risks that can be there what are the risks.

Participant 2 F:	This stage is like experimental we don’t know what the outcome will be we put people on a risk that they may get sick or develop other problems as the other one said it can be like we are putting a person on a risk, a person can suffer from other things or die.

Interviewer 1;	Ok, alright any other type of risks.

Participant 3 F:	For me whether it’s a question or I feel like maybe these people can come twice and you find a person is affected or getting critically ill because has come twice for money.

Interviewer 2:	What should we do to restrict people coming two or three times.

Participant 3 F:	Maybe you should get identity.

Interviewer 1:	Another risk

Participant:	No response

Interviewer 1:	So, looking in terms of benefits and risks of this type of study which side of the two outweighs the other between benefits and risks.

Participants:	All benefits outweigh.

Interviewer 1:	Benefits are more why are you saying benefits are more.

Participant M:	Benefits what happens is risk is within that time of period while benefits are extended to generations so what we are looking at now is something that is extended to generations we have said risk happens to a person at that time and the risk is something that can be controlled but the benefit that we are talking here be it vaccine or what it means all the others coming unborn children they will also benefit

Interviewer 1:	There are other views in addition on the two benefits and risks that can be there, between the two which one outweigh the other.

Participant 2 F:	We have said benefits outweighs why when you give a person the vaccine you look after them, that means challenges that can be there have been taken care of, meaning that’s a benefit, benefits outweighs when one has developed side affects you are able to help them.

Participant 3 F:	to add on benefits you mentioned you will look after a person maybe that be on the part of benefits if you can take it there should not be period that you are done with participants. A person has helped the future work to go well as well as for others to get help don’t part ways with that part that will be a benefit to the whole nation maybe they will continue taking them to Mwaiwathu or how many people you want to take when its approved or maybe they did not go to Mwaiwathu or just communicating with them is valuable that’s a benefit.

Interviewer 1:	Alright we want to ask your views your friend or your relative if they tell you he wants to join a study of this type for example for TB where he will be given the bacteria how can you feel.

Participant 8 F:	With what I have understood I can encourage him to join because I have seen benefits, have understood, I know what he will undergo where he is going that he will receive help this if things happen this way, because I understand I can encourage him to continue with the decision.

Participant 2 F:	If my relative tell me, I have heard and have followed what I can enlighten is on how his healthy is to understand, screening has to be done to check if he is eligible to join the study because I can check him physically at home to join but I don’t know I don’t know how he is in his body if his body is fit to join study. I can enlighten on that I can’t deny him but that this must be investigated clearly.

Interviewer 1:	Other views/opinions we are agreeing that friend or relative we can give him ok to join this type of study without any reservations.

Participants:	All yes

Interviewer 1:	We don’t have any worries about what has been discussed on the study.

Participants:	All

Interviewer 1:	So, other studies of this type have been conducted here in Malawi we are talking of studies that used pneumococcus bacteria in healthy people who are no sick. How can you differentiate that study and this one we will start with TB the difference that can be there doing this type of study doing it in healthy people what can be the difference that can be there.

Participant 4 M:	The issue am seeing here is that the other one we were doing it with healthy people now these are people who their lives are already at risk their immunity is low that part there is a difference.

Interviewer 1:	The difference we can point out in several areas, the difference if we just say the difference is on immunity we will cover everything expand on that, point out the difference is coming in what way , different people will look at this information some non -medical like myself who are just doing interviews part of the study, others medical personnel s for them to have a clear picture of the differences considering which areas.

Participant 7 F:	The difference as my friend has explained because we are looking at a person with HIV who their immunity is already is compromised chances are very high that person, we give bacteria which causes pneumonia, or you are giving TB bacteria to that person the person can get critically sick. It will depend on at first you started with healthy group the challenges that you faced with healthy group be well prepared because this group is not stable. Be well prepared if these people will come with serious problems unlike the other ones you have to be able to assist them quickly.

Interviewer 1:	The others you have other opinions, I saw like someone want to start talking the other side. Alright we have discussed in terms of HIV what about in terms of TB the difference that can be there between healthy people who participated and this group for TB the difference that can be there.

Participant:	No response

Interviewer 1:	Similarity or difference

Participant 2 F:	I see the difference can be there, the difference is that it will give you a true picture of vaccine effectiveness because if you give a person with TB, it works effectively you will know if the vaccine has worked effectively on the ground.

Interviewer 1:	Alright there is another difference, just want to go back a little bit we discussed on risks that can be there when a person participates in the study. I want you to help us on the risks that can be there and how we can explain the risks in a way that we don’t scare people from participating in the study or risks to send people away people from participating how can we explain about them to people who want to participate.

Participant 8 F:	I feel like in the discussion if we can tell them the truth that when we are giving vaccine we tell people that it can happen you will notice some changes in your children’s body it is not dangerous, don’t be scared if this happen because the body has received something strange, it’s the same explain to them to understand that what we give you will notice something in your body because the body has received something which is not part of the body. Explain to them that something will happen when they hear that they will accept it and if that happens, they will know that it will happen

Interviewer 1:	There is another way on how we can explain the risks that can be there.

Participants:	No response

Interviewer 1:	Or so it is.

Participant M:	Yes, it is.

Participant 2 F:	To add on other things look small but to come out with big results telling them if they face anything with the vaccine they receive as we said every vaccine some children react to it, and they react differently one can have mild sickness, another serious sickness the main thing goes to hospital. When you explain that they can understand and rush to hospital for treatment

Interviewer 1:	Mm alright want to know concerns that you have when your relative or friend take part in a study like this what can be your concerns that you can have.

Participant 8 F:	The concern that I can have been this thing is a study not so the results have not yet come out, the results will come out through my relative. The concern that I can have is about results that will come out I don’t know what results will come out it be positive that you have achieve what you wanted in the study or it will fail that is the concern that I can have or how it will end for my relative because we don’t know as we are trying this thing what will be the results, will results be positive we expect two possible result outcome and we don’t know of the two what will come out.

Interviewer 1:	Alright another concern /worry

Participant 2 F:	Another concern is we anticipate good news or bad news, the concern is if something bad happens adequate support will be available

Interviewer 1:	There is another concern, let’s come to comments we are wrapping up comments that you have on the topic that we were discussing this afternoon. What comments can you give at the end of the discussion?

Participant 1 M:	What is bothering me a little bit is one thing you come with what you are here for a while, this has been coming many studies have come but mostly as community people we don’t get feedback. Feedback saying the study we conducted these are the results coming on the ground this does not happen. This we tell people that it will help to come up with vaccine and then we find that is not done people end up having questions, what are these people looking for. That is the mentality that we have with study, what are they looking for is this true or they just say they are looking for something behind the study. We have, people have questions because we don’t get feedback you conduct a study at the end of the day, years passing the study does not bring any benefit to people or the community. We remain with unanswered questions.

Interviewer 1:	Another comment

Participant F:	As explained when the study is taking place inform us as you have invited us that what we started this is the results because it looks like you need us at the beginning later have no business with us.

Participant 2 F:	Another comment is that studies must be conducted through studies is how we are fighting with different diseases it is an encouragement, to have studies being conducted because through studies we have fight different diseases through the vaccines that are given.

Interviewer 1:	Alright another comment

Participant 3 F:	If from all the groups that you interacted with we talking of the same thing I believe you will follow that to conduct the study as the study is not starting soon you are gathering views of people, I believe if all of us we are talking the same thing the study has to be conducted so that all of us can benefit in future. Any medicine goes through this process perhaps the help for our friends in future will be helped if this does not help us.

Interviewer 1:	There is another comment.

Participant 1 M:	During the time of conducting the study will you need us or not or the way we have done you will not need us I don’t know maybe I did not understand.

Interviewer 2:	Ok responding to your question when we have started the study it will be your choice to decide to join study or not that is the importance that we can mention as you said it should not be we just invited you at the beginning and it ends there. We will consider giving feedback on what we discussed this is the feedback.

Participant 1 M:	Ok

Interviewer 2:	But in the main study it will be a choice to join the study.

Participant 1 M:	If you meet criteria requirements.

Interviewer 2:	Yes, if you qualify to join, we will welcome you.

Participant F:	Mien is a question for the people who you will take those with HIV as well as Tb how you will go about with them.

Interviewer 2:	Ok as we said in the studies that we had conducted we were going to health center’s explaining to people that we have a study those people with this, when you feel free you can find us at 3A clinic to explain more when we reach out to people in that way, they find us on the day we tell them. In that way the first group tell their friends we don’t have problems getting people we don’t go to health center’s people come on their own.

Participant 4 M:	Maybe a question I should ask, aa that’s all.

Participant F:	You answered yourself, thank you very much.

Participant F:	Let’s hear from you.

Participant 4 M:	I want to ask are you planning to conduct the study only here.

Interviewer 2:	Thank you, very much good question, so far, we have finished questions not so. As one of team members who will conduct the study thank you very much for participating your views/opinions that you have shared are important. Questions and comments shared we will use them as stated. We have heard your complaints that we will discuss we will give feedback. Thank you for your time and all the views that you shared. Thank you, the rest my colleague will take over.

Participants:	Thank you

Interviewer 1:	Thank you very much we are at the end of our discussion, thank you for coming, today the weather was not good thank you for the commitment coming at this place to help us to give us your views. All the information shared is important and will help us moving forward in planning for this study in future, thank you very much.

Participants:	Thank you very much
