# Supplementary material for: Acceptability of advanced Controlled Human Infection Models: The case of a pneumococcal challenge in people living with HIV and mycobacterial challenge with Bacillus Calmette Guerin in Malawi
Source: PLOS Glob Public Health. 2026 Aug 18;6(8):e0006236. doi: 10.1371/journal.pgph.0006236 (PMC13485124; doi:10.1371/journal.pgph.0006236)
Supplement: Supplementary file 1 [file pgph.0006236.s001.zip › Documents/ACHIMA_IDI_05_TB SPE_CG_27.02.24.docx]

Interviewer:	Welcome today to tis interview today on 27 February where we are having interviews with senior doctors working here at Queens. Feel free the interview in this discussion we can use both Chichewa and English. For your part much of the questions will be asked basically asked on TB challenge that we are talking about. Much of the questions will be based on that.

Participant:	Ok

Interviewer:	Have you heard or read anything about controlled human infection model study

Participant:	No, I think this is the first time

Interviewer:	It’s the first time.

Participant:	Yes

Interviewer:	Ok, to understand more about human controlled infection model studies there is a small animation that you will watch, that explains more about this

Video play:	It’s a given that vaccines prevent diseases and saves countless lives, but this takes ten years or more to develop new vaccines. We need to find a faster way, one way to speed up the process is by making better use of human infection studies, volunteers are given a sample of vaccine under development followed by a careful monitor infection medical care is always close to hand if volunteers fall ill and if they stay well, it means the vaccine works and can be made available much earlier. The idea is almost 300 years old but using todays advanced technology this type of study is not just scientifically sound but ethically responsible too and of course follows strict rules. Human infection studies show huge promise against fighting diseases like Typhoid and malaria but to develop vaccines most effective at the people at risk we need to test on those at most risk by expanding human infection studies and targeting them better the people most at risk could help develop vaccines better and could save millions more millions live.

Interviewer:	Ok, we can proceed.

Participant:	Yes

Interviewer:	Having watched the video, to what level would you agree or disagree to conduct a TB CHIM, TB challenge in health adult volunteer? You would agree, Disagree, Neutral, Undecided

Participant:	I would agree

Interviewer:	You would agree

Participant:	Because it says it will be done in health volunteers, yes I would agree

Interviewer:	In your view what would be the benefits of conducting a TB CHIM in health adult volunteers?

Participant:	The aim is to develop a vaccine not so

Interviewer:	Ok

Participant:	I think it’s one way of, it’s part of research, it’s one way of developing vaccine done in health people that will help even those people that might have compromised immunity later on if they get the vaccine, I think they are likely to have some sort of immunity. So maybe it will be essential it’s like if you give, the research is done and then maybe the vaccine is done as well and accessible to people I think in the end we will have people who are immunized against the disease because currently we have got a high pattern of the disease especially in Sub Saharan Africa.

Interviewer:	Thank you, in your view what are the risks of conducting a TB challenge in health adult volunteers. The risks that you feel they can be there.

Participant:	The only risk that can that am worried about is them developing a full brown infection from the I mean the vaccine that will be given.

Interviewer:	The BCG, the challenge agent that will be

Participant:	The challenge that will be used, am not sure weather they are, will set some measures that will make sure or ensure that people that are volunteering are really health. That is because some people may come in because they want to benefit financially but maybe hiding something. I don’t know if there will be measures to make sure that whoever participates is declared health enough so that maybe there shouldn’t get the disease from participating in the research project.

Interviewer:	To answer on that basically they have to do the screening part like to confirm that the person the participant who is joining is really a health adult not just basing on the information that they have provided on their side. They will also do the screening part like to confirm that this is really a health adult. Any other risks that you feel might be there?

Participant:	Not really just some minor minor things if they can develop a hyper sensitivity reaction from the vaccine or maybe some infection local infection on the injection site if it will introduced through the skin yes.

Interviewer:	Mhi,

Participant:	Yes, those are the risk full brown infection, hypersensitivity reaction and maybe local infection.

Interviewer:	Hypersensitivity reaction and the other one is the third one.

Participant:	Local infection as in on the injection site

Interviewer:	Ok, thank what other concerns do you feel could arise conducting	a TB challenge in health adult volunteers. Other concerns that might be there

Participant:	I don’t have any concern now apart from the ones that I said earlier, since people will be screened, I don’t have any other concerns

Interviewer:	So, I would like to ask you some question that are regarding participation do you feel it is acceptable for a male adult volunteer or a female adult volunteer to participate in these type of studies

Participant”	Yes

Interviewer:	There are, you don’t have any preferences that males can participate unlike females

Participant:	I think they equally have a chance to participate I don’t think there is any gender disparity for. I think as far as someone is healthy gender to me doesn’t matter.

Interviewer.	Like for the previous study that MLW conducted that was under CHIM model study, it was noted that more males joined that study unlike females, what could be some of the driving factors that lead more males showing interest in joining or participating in that study?

Participant:	I may not know but if you think of our cultural practices some females that are married, they have to get consent from the man. And if the man is not comfortable, they will not come unlike our male counterpart who may not need to get consent from anyone. So, it depends on culture or maybe which part of the country they come from am just speculating but maybe culture may play a role. And also, maybe education level may play a role I don’t know I don’t have statistics maybe males are more literate than females they would understand the importance of maybe participating in the study.

Interviewer:	So as moving on forward for the study that is being intended to be conducted the one that we are talking about the TB challenge, there is a plan like to engage those females that will be joining like participating in the study as we are talking someone an adult like someone who is above 18. For female participants who would like to join in the study during the time they will be participating in the study these women will be asked like to be practicing family planning methods what can be your views on that, this will be during the time when they will be participating in the study. What are your views?

Participant:	Ok, I understand that maybe the aim is to make sure that we remove all risks as in if someone get pregnant the immunity may be compromised unlike someone that is not pregnant and also the risk of transmitting if they get to have a full brown infection from participating in the study. Then they are also putting them at risk the conceived baby and also any way am not an obstetrician if someone is infectious or they are at risk of loosing their pregnancy, yes I agree with the plan of making sure that they don’t conceive during the study to minimize the risk of transmission of the infection or development of the infection on the part of the mother or even loosing the baby that’s a miscarriage.

Interviewer:	So going now into the details of the study procedure going into details of the study. As part of the study participants will be injected with BCG as a challenge agent, this is the same BCG vaccine given to children in Malawi as part of vaccination policy, but this will be given at different doses to be able to collect it by taking part of the tissue from the injection site (biopsy) and also to understand how the body’s immunity responds to BCG. How do you feel about injecting BCG?

Participant:	I don’t think there is any problem, but the taking of, the getting of Biopsy that’s invasive I don’t know weather there will be a hiccup along the way if people will not be comfortable to have an invasive procedure done on them. I know even the BCG is invasive, but it is not invasive enough to get the tissue from their body.

Interviewer:	So, where on the body will you prefer to inject with the BCG

Participant:	I think on the upper arm, the usual

Interviewer:	Why the upper arm

Participant:	Maybe because that’s where we get injected and also maybe the upper arm of course there are several areas that are muscular but for absorption that’s a good place

Interviewer:	You have already mentioned the issue to do with biopsy, there might be a problem having this done

Participant:	Yes

Interviewer:	I would like to ask how this should be explained for people to understand about the procedure of biopsy

Participant:	I think researchers have to give them enough information before they consent initially to give them, because you have told them weather them participating in the study will make the uncomfortable or not. Because you know getting an injection some people are not brave enough to get that and later on getting a biopsy but I guess someone cannot get a biopsy without giving the something like a local anesthesia so I think it will be nice if people are told beforehand. Ok we will give you an injection, but we will also get a biopsy which you will not get any pain maybe you feel some pain but not much because we will give you a local anesthesia.

Interviewer:	There are several ways of introducing BCG in the body, one of this is by putting BCG in the lungs. This methos uses a tube that passes through the nose or mouth the throat to the lungs, (bronchoscopy) this method is also used to collect samples from the lungs. What are your views on this?

Participant:	It’s also invasive

Interviewer:	Ok, this is also invasive, so in terms of the study how can this be properly explained like for participants to understand

Participant:	Let me ask you a question are they going to choose to like as a mode of introduction of the BCG or you will use all the ways in one person, like all the methods like you inject them maybe bronchoscopy what was the other one.

Interviewer:	Injection and bronchoscopy

Participant:	Are they going to choose, or they are going to use both ways

Interviewer:	As it is now because we are just at the first stage, they are just exploring the ways that are being used in the hospital set up to do this. For the study after collecting the information and analyzing everything it’s when they will come up to say this will be the proper way, the way that the study will use, for now they are just using the ways that are already in the health sector that are being used like when treating patients, I should say.

Participant:	I think bronchoscopy is more invasive so if I were a participant, I would rather go for ana injection. Ok the other question is if you do bronchoscopy to introduce the BCG how you will get the sample for histology as in because you said an injection and then you get a tissue right.

Interviewer:	Yes

Participant:	So, when you introduce in the lungs using bronchoscopy how are you going to get the sample where

Interviewer:	As to just as I have already said that am not medical personnel for the other tests that will be done, they will also be getting4 some blood samples for further testing on this for the bronchoscopy part I will not be like in a better position to respond that directly.

Participant:	Ok, for me I don’t know how the tissue or what type of sample you will get after bronchoscopy, I think and considering that its invasive enough because they have just to make use just my arm, I think I will go for the arm

Interviewer:	Alright, s moving on to the other part for this bronchoscopy and biopsy how best can this be explained for a lay may to understand this easily about these procedures.

Participant:	You know am not sure this will be of any help on that one but maybe because I prefer the arm its easier to tell the participant how the kids because kids get vaccine so it’s straight forward to tell them ok, we will give you vaccine but it will be given the way babies get their vaccines. Bronchoscopy not very common procedure, I don’t know how better we can explain it so that participants should understand. Like you said inserting the tube and what but am not sure whether it will have a positive reception from participants. Because you know when we are explaining we have to explain the risks as well so am not sure as well weather the risks of bronchoscopy I don’t know but it’s easy to explain about the injection.

Interviewer:	So as part of the study participants participating in controlled human infection studies are sent to a residential stay in order to monitor them closely during the early days of introducing a challenge agent. What do you think about the arrangement of sending participants to a residential site for the study.

Participant:	How long will they stay there

Interviewer:	It’s a question that I will ask you that what will be the recommended number of days that participants can be kept there after a challenge agent has been introduced.

Participant:	The other, the aim is to monitor them

Interviewer:	They will have a close monitoring if there is anything that may come up

Participant:	Mm, that’s a huge task, imagine a family man or a career person or even a businessperson confining them to some places, how are you going to make sure that their work is not compromised. What are the things that you have put in place to make sure that their lives are not disturbed. Think of someone that is married looking at their children a mum with a one-year-old, two year old a toddler they can’t go together so I think my concern is disturbing the family structure by taking out the mum who cooks for the children, the man who brings food in the home or the wife who brings food in the home or someone who solely depend on business. Am not sure, am not sure I feel like there will be a disturbance of some sort, I know they maybe there will be some compensation, but will the compensation be enough to make up for the lost time, the lost opportunity yes.

Interviewer:	Ok, in this case for the study that they have done previously for the participants after they were introduced a challenge they were sent to the site in the evening to monitor them from the day they were introduced the first day they go there after finishing their normal routine days, if they go to work after work they go to the site where they are monitored for the first three days.

Participant:	But who monitors them during the day

Interviewer:	During the day they are not monitored they are at the usual place at school

Participant:	So, what’s the difference between the night and day because it’s the same person, it’s not like during the day the vaccine will go away.

Interviewer:	The Idea was that if some of them may develop some other signs and symptoms like to trace them because they are coming from different areas to follow up on them

Participant:	And what about during the day, how are going to trace them

Interviewer:	That was the understanding about the study so in this case we wanted to have the ideas what can you say about sending participants to residential site is that feasible or unnecessary

Participant:	I don’t think it’s feasible, I have already given the reason, the second reason I said you are taking the opportunity but you said it’s during the night so they will go to work they will do their business, but monitoring I thought it’s the whole 72 hours of monitoring not taking out the daytime. I don’t find it I don’t know, someone has to convince me why you don’t monitor them during the day do diseases develop at night only or there are some measures that have been put in place that they are safe during the day.

Interviewer:	I would like to ask you in terms of

Participant:	I hope we are finishing

Interviewer:	Yes, participants who are intending to join this study who would you recommend who they can give as another person who can be contacted in case of anything that has happened during the study

Participant:	As in the next of kin or

Interviewer:	As in someone like a proxy someone who can be contacted on behalf of the participant

Participant:	I think someone who is close to them

Interviewer:	Ok.

Participant:	If they are married, they are close to the wife, if they are living with their parents, they are close to the parents, the relationship doesn’t matter someone who will be around them during time they have got the vaccine.

Interviewer:	Ok, if someone close to you tells you that he/she want to participate in this type of study how would you feel

Participant:	As far as they are healthy, I would encourage them to

Interviewer:	Ok, you will encourage, what will make you to encourage them

Participant:	Ok, the thing is you are saying if themselves they are already willing its not like am making a decision for them. They are already willing I don’t think I can take them out.

Interviewer:	They have made the decision to participate and you just like support

Participant:	Mhi, just supporting their decision

Interviewer:	Alright, will you be worried with anything about them participating in the study

Participant:	It depends on what they are doing, if this will disturb their or because I guess at the end of the day, they will be healthy people right.

Interviewer:	Yes,

Participant:	So, my only worry will be, will they develop the disease like full brown infection that will be my only worry.

Interviewer:	Developing the disease, ok any other comments that you may have

Participant:	No

Interviewer:	We are at the end of the interview I would like to ask your personal information like date of birth month and year

Participant:	6 June 1979

Interviewer:	This is the end of the interview that we were having this morning. I would like to thank you for your time and the contributions that you have made towards this interview. Thank you!

Participant:	You are welcome
