# Supplementary material for: Acceptability of advanced Controlled Human Infection Models: The case of a pneumococcal challenge in people living with HIV and mycobacterial challenge with Bacillus Calmette Guerin in Malawi
Source: PLOS Glob Public Health. 2026 Aug 18;6(8):e0006236. doi: 10.1371/journal.pgph.0006236 (PMC13485124; doi:10.1371/journal.pgph.0006236)
Supplement: Supplementary file 1 [file pgph.0006236.s001.zip › Documents/ACHIMA_PLHIV FGD _13_English_11.03.2024.docx]

Video:	Alright. Let me just quickly explain that it has been discovered that vaccination prevents various diseases but for the vaccination to be developed, it takes a long time. So, one of the ways for the vaccination to be developed is in a short period is to test the vaccination with human beings by first infecting the trial participants with a bacteria that causes some disease and then see if the vaccination is effective. The video that we have watched was showing some of the things that happen during the process of testing the vaccination. The video showed a person being injected with a bacteria and then being observed if the immune system was able to fight the bacteria without showing any side effects or if there were any challenges afterwards. So, if the person is okay and has come out with no side effects, it entails that the vaccination has successfully worked as expected. However, if a trial participant has showed side effects, then they are kept under observation. So, the video we have just recently watched is showing us and explaining about vaccination and observations for its successes and challenges.

At this juncture, we would therefore like to start our discussion and we will be using the numbers that you have picked to show who is speaking at any time. I would therefore like to ask you all to be open during this discussion and speak loudly so that we can clearly hear your views during the discussion. So, I would like you to start by telling me what you have learned from what I have explained about our discussion today. What do you think is the reason for our discussion today? You should first mention your number before speaking.

Participant 5 F**:**	What I have learned today is about the vaccination for pneumonia that will happening in future in order to help people not to suffer from … after they get the vaccination.

Interviewer:	What other thing have we learned from the discussion

Participant 9F:	The research that we are undertaking here is teaching us is telling us that the vaccination will help more the people who are on ARVs. The vaccination will protect the people because even when taking ARVs some people may have underlying diseases such as pneumonia so the vaccination will protect the people from pneumonia hence the study would like to test the vaccination with those people who are on ARVs and see if it can be effective to be administered to people to fight pneumonia.

Interviewer:	Alright. Other things that the others have learned?

Participant 4F:	The person that has been vaccinated with this stay’s health as in the past and does not get sick frequently hence the vaccination helps people not to be regularly sick. You have also explained that after a person gets the vaccination, you put them under observation to see if the vaccination is safe or not. You have also said that this study was previously done where the recipients did not show any side effects which means that the vaccination worked well.

Interviewer:	Alright

Participant 2 F:	Speaks inaudibly as there is background noise overwriting her voice.

Interviewer:	Alright. So, in your own views after learning about this vaccination study, do you think it is a study that needs to and has to be done? Do you think it is a study that has to be accepted to happen or not to happen?

Participant 9 M:	The ideas that we have learnt about this study show that this study can be accepted to happen because as you have told us, the study has to be there to help the testing of the vaccine that fights diseases. I believe if the study is to help fight diseases, then it is acceptable to be conducted.

Interviewer:	Alright

Participant:	I had the same view…

Interviewer:	Alright. You can also explain because explanations may differ. (Interviewer referring to a different respondent)

Participant 7M:	This study seems approvable because as you have already said it reduces infections, so it is acceptable.

Interviewer:	Alright

Participant 6 F:	This study is acceptable because as you have already said, the vaccination does not have side effects on those to take it hence it can be approved.

Interviewer:	Alright,

Participant 5 F:	The study seems it can be approved to be done because if there is a challenge there is assurance that the one affected can be treated. And this vaccination was accepted by the government to be implemented.

Participant 3 F:	This vaccination study is acceptable because if fully implemented, the vaccine will help to prevent people living with HIV from dying from pneumonia.

Interviewer:	Okay. Number?

Participant 1 F:	If this study is accepted it can help so that the next generation not to have diseases like pneumonia while also avoiding deaths that comes due to this problem.

Interviewer:	Alright

Participant 4 F:	This study will be accepted by people because recently TB and pneumonia are the diseases that are highly spreading and killing a lot of people.

Participant 2 F:	This vaccination study is acceptable because clinicians, nurses and even chiefs see it possible…not very audible.

Interviewer:	Alright

Participant 8F:	This study is acceptable, and we are happy about it here because when there is side effects we will see signs such as body temperatures getting high hence we will rush to the hospital for assistance from Doctors. So, we have received this study wholeheartedly.

Interviewer:	Alright. So which message do you think is very important about this study?

Participant 3 F:	We are finding a way in the vaccination to defeat pneumonia and TB.

Interviewer:	Alright so, our discussion should mainly focus on pneumonia. The discussion just touched on TB but our focus today is on pneumonia. What message do you think is very important from this study?

Participant 9 M:	The other message that is very important about this study is the message about wishing to defeat pneumonia which has been wreaking havoc and has been killing a lot of people.

Interviewer:	Ehe…alright. Another important message that you are getting from this study.

Participants:	…silence from respondents…

Interviewer:	Alright. So from your understanding, which message do you think should be added when disseminating information that lets people know about this vaccination study?

Participant 6 F:	One of the messages that could be added when informing people about this study is for us who have first heard about this study to be spreading words of mouth to others.

Interviewer:	Alright

Participant:	For this message to reach a lot of people, there is need to use a car with megaphone spreading the message just like they do when spreading information about diseases such as typhoid. So I think for the message to reach more people quickly, they should use a vehicle that has megaphone/ PA system mounted on it.

Interviewer:	What is another additional message that should be included when reaching out to people about this study?

Participant:	…silence from participants…

Interviewer:	Alright. So what questions do you have concerning this vaccination study?

Participant 8 F:	So, the bacteria injected through this vaccination cannot affect a person other thing?

Interviewer:	Alright. Your question is part of some of the things that are to be discussed here so we will discuss about it when we reach that part, since this discussion is about discussing everything pertaining to this study.

Interviewer:	So when you think about it, do you think there are any benefits for doing this CHIM study on people on HIV treatment (where people are injected with virus)?

Participant 4 F:	Yes, there is a benefit because we will be able to know that how diseases like pneumonia have increased or reduced among the patients on ARVs.

Interviewer:	Alright. Another benefit that could be there?

Participant 2 F:	The other benefit that could be there we will have an opportunity about our status and pneumonia (not audible)

Interviewer:	Alright.

Participant 3 F:	The other benefit is that if the study is approved and commissioned, there might be an opportunity to discover a drug that should be administered to people who have been found with pneumonia and are on ARVs.

Interviewer:	Alright

Participant 9 M:	The other major benefit is that completion of this study will help everyone in Malawi who is on ARVs to accept getting this vaccination.

Interviewer:	What other benefit could be there on both sides?

Participant 7 M:	It will help other people who will be able to access this vaccine when they are on ARVs.

Interviewer:	Alright. Let’s now talk about the challenges. What threats do you think could be there when conducting this kind of a study?

Participant 9 M:	The threat that can be there is that maybe the bacteria that is injected into a human test cannot heal hence the person might die which is a major threat.

Interviewer:	Alright.

Participant 4 F**:**	You explained that the study subject involves those who do not have pneumonia hence the subject might hide the fact that they already have pneumonia in order to be part of the study.

Interviewer:	Alright

Participant 5 F:	A person should not be enrolled without being tested first so they have to be tested for pneumonia and when found with it not be enrolled for the vaccination study.

Interviewer:	Alright, lets continue. What other threats can be there? What threat do you think can come through during this study?

Participant 4 F:	The danger comes in when the vaccination fails to fight the injected bacteria hence the person might be regularly sick hence it cannot be good when the person is also on ARVs.

Interviewer:	Alright. Another threat?

Participant 6 F:	Just as my friends have said, the threat is there because the study is to be undertaken by those on ARVs treatment hence it may affect their health which is already weak. Don’t you think this can just cause further problems for these people?

Interviewer:	Alright, lets continue expressing our ideas on the threats that can come in when a person takes part in this study

Participant 9 M:	I think we have finished.

Interviewer:	So when you weigh between benefits and threats about this study, which side do you think overcomes the other between benefits and threats?

Participant 5 F:	The benefits overweigh the threats.

Interview:	Ehe…can you explain

Participant 5 F:	Because if this study is done and the results are positive, some more people will benefit as they will be able to get the vaccine that will prevent them from pneumonia. Therefore, the number of people on ARVs suffering from pneumonia will reduce or completely drop.

Interviewer:	Alright. For the others, which one do you think overpowers the other between benefits and challenge?

Participant 3 F:	It is the benefits because each and every medication has to be first tested before approved for use. So I believe even when being tested, the producers know that the vaccination may not cause too many challenges to the subjects hence benefits overweighs threats.

Interviewer:	Alright. So let’s continue with our discussion. We were talking about which one overpowers the other between benefits and threats where is has been seen through this discussion that benefits overweighs the threats that may come with the vaccination study. So I wanted to now mention that this kind of study about pneumonia was conducted but with health people who are not on ARVs. What difference do you think could be there in conducting this same study but now with different type of people who are now here?

Participant 9 M:	There could be a big difference because you have said that the previous study was conducted on people who are healthy and are not on ARVs while this one will be on people who were found to be HIV positive. The difference is that those with HIV might have several problems in their body as they are dependent on ARVs while the first one was about people not regularly sick as their immune system is perfect. Now there is a huge difference because now the person has low immunity and depends on medics to improve it hence this study will help more those subjects on ARVs as they will discover the right medication that will deal with pneumonia.

Interviewer:	Alright. The other difference?

Participant4 F:	The difference between a healthy person and this one on ARVs is that people might think that the one on ARVs will be regularly sick but since you have said that you will involve subjects whose viral load is well hence you will be able to differentiate between the healthy subject and the one

on ARVs and see which one will be regularly sick after administering the study vaccine and the bacteria infection.

Interviewer:	Alright…Any other views?

Participant 1 F:	As you have already tried the healthy ones (HIV negative) and now you want to try the HIV positive subjects, you would like to know how this vaccine can assist those on ARV treatment as those on treatment already have low immunity. Hence you would like to determine how they can benefit if administered with study vaccine.

Interviewer:	Alright, now let’s get back to the threats that may come about due to this study. How do you think these threats can be communicated to people before the enroll to be part of the study vaccine? The method of communicating to the people about the threats that may be there in the study

Participant 3 F:	So when we are giving counselling when a person is starting to take ARVs we tell the people that we all have different bodies that respond differently to medications hence I think the study subjects should be informed the same way that everyone is different hence some people might face challenges when they partake in the study while others will be just fine.

Interviewer:	Alright. Another view?

Participant 9 M:	I think just like my friend is saying, every medication has the benefits and challenges, but the challenges are not huge. So I believe there is no big threat as you have said that the vaccine was already tested among healthy subjects and you just want to determine how it will work with people living with HIV. However, the people have to be told about the benefits and threats of the same so that they are in the know.

Interviewer:	Alright. Let’s now go on to another issues. I initially mentioned that when a person has accepted to take part in the study they go and stay at a place where they are observed during the study. What can you say on this arrangement that a person stays in the study area?

Participant 9 M:	This person when he agrees to take part in the pneumonia vaccine study and moves to a place to be observed, I would say it’s a good thing because people react differently to medications hence when left in their locations there may be challenges in accessing quick help when sick due to reaction of the study vaccine. Therefore, the person needs to stay close to the hospital personnel administering the study so that they are looked after well and determine the challenges instantly as they emanate.

Interviewer:	Alright. So on the issue of keeping the study subjects on the same place where they are taken care of by hospital personnel, for how many days do you think the people will be staying under observation?

Participant 9 M:	Its will depend on you as people administering that study hence you are the one who have to tell the people that they will stay for such a long time under observation.

Interviewer:	Alright. There I wanted to solicit your views on how many days could be acceptable for one to stay under observation.

Participant 4 F:	As this study was already administered to healthy people (HIV free), how many days were these staying under observation? Because the healthy people and those with HIV cannot be the same

Interviewer:	I mentioned 3 days for those involved in the earlier study. So now I am asking about you.

Participant 4 F:	For us, it maybe should be 5 days.

Interviewer:	Alright, how about the others? How many days do you think the study subjects should stay under observation?

Participant 7 M:	I think the person should stay under observation according to how he is responding so we can’t really say how many days the person should be visiting the hospital for checkup.

Interviewer:	These observation days are referring to those days that you stay at a study facility. How long should the subject stay at the facility after being infected with the bacteria.

Participant 9 M:	I think the person should be staying for 3 days just like the other healthy participants in the previously administered study. Why am I saying this is because you have said that you are not just taking anyone with HIV but rather have to first measure their viral load and immunity and when the person has high immunity and low viral load, it’s the same as a healthy person who can work well and not regularly sick hence there should be no difference?

Interviewer:	Alright.

Participant 8 F:	I feel the 3 days are ok, but it will depend on the determination of the doctor to see if the person is back to normal (well) in those days and if the vaccination has fought the bacteria. The subject can be released if it’s well in 3 days, however is the subject supposed to go back when they don’t feel ok after those 3 days?

Participant 6 F:	I feel like you should differentiate the stay in days from those stayed by healthy subjects. If a negative person stayed 3 days, then the positive person should stay for 5 days at the observation facility so that progress is observed.

Interviewer:	Alright. In response to the question that participant number 8 has asked to when the person has completed the facility observation days and has returned home. The thing is even after completing the facility observation days, the subject is told to alert the study facilitators if they face any challenges so that they keep in contact and see what is happening and that the subject can be given the right treatment when having side effects from the study.

Participant 4 F:	I have a question, so after how many days does they test if the injected bacteria has been defeated from the immunity during the days of subject observation at a facility?

Interviewer:	It depends on the procedures that are laid down for the study being undertaken so each study has a criteria pertaining to after how many days should a person be tested after being subjected to the vaccination. So the days differ depending on the type of study hence I cannot say here that it will take such days for one to then undergo the test. I hope I have answered your question.

I now want to ask if you feel that the procedure of keeping the study subjects under observation at Grace Bandawe is important or not important to this study.

Participant 8 F:	I feel this is a very important aspect of the study because when the subjects go home and end up facing a challenge that hinders them to make it back to the hospital/	Grace Bandawe they may end up maybe dying but if they are kept at the place the Doctors will be right there to treat them in case of anything.

Interviewer:	Alright.

Participant 4 F:	I think they made a very good decision because as we are here, we come from different locations such as Lunzu, Machinjiri, BCA, Chilobwe, Manase which are long distances to the facility and would make it difficult for the doctor to come when we call them after facing challenges. For instance if say 3 study subjects call for help. However, it would be easy to handle if all the participants are at the same place because they may just walk from Grace Bandawe to Mwaiwathu hospital to seek medical treatment.

Interviewer:	Alright. So when a person is enrolling for this study there is need to them to submit name and contact of the next of kin person who can be contacted if there is anything so I wanted to know what kind of a person should you want to be the next of kin for any contact.

Participant 4 F:	The person should be a relative who when you get back home and is not feeling well to make a call, they can call the study facilitators for you to get help. So it has to be a relative because a guardian has to be a relative and not just any other person.

Participant 6 F:	We have to think deeply about the next of kin for the study because usually most relatives would not be in agreement for us to be part of the study subject while we want to support this study. So I just wanted to enlighten my friends here that when giving the next of kin contact we have to rightly think on how to go about it.

Interviewer:	Alright. That’s another view pertaining to enlisting of next of kin. So on relatives we have different relatives, some we live with in the same house while some other relatives live somewhere else. So in this case, should the next of kin be the one we are staying together with or someone who is even staying away?

Participant 9 M:	I will concur with what has been said because there are two things about relatives. There are some relatives who care and there are other relatives who you tell things just because you can’t do otherwise based on how they talk. This is different from those who are in a marriage and have a wife/husband because you cannot do a think without telling the other person hence not difficult for a next of kin. However, there are other people who we stay with in our houses who likes talking hence you will find that some people take someone who is not a relative to be their next of kin or guardian at the hospital even though they have relatives. So I don’t know how we can go about it on this issue.

Interviewer:	Here I wanted to hear your views on who you would be comfortable with to be your next of kin, whether it should be someone you live with(relative) or someone you are not related to but you understand each other.

Participant 9 M:	It will be a decision of the study participant whether to have a relative or a friend who he/she trusts as a next of kin contact for the study.

Interviewer:	Alright.

Participant 1 F:	On the part of guardian/ next of kin, you can take anyone who you are open with and knows your secrets because that person will be on the forefront to know whatever happens to you. However, the person should have a contact number for easy tracing.

Interviewer:	Alright. Let’s now move from this discussion about next of kin to the next section. So on this kind of study, who do you think should take part between a man and a woman?

Participant 3 F:	Anyone can take part regardless of gender.

Interviewer:	Alright. Everyone should take part. Other what do you say?

Participant 4 F:	Everyone can take part regardless of being a man or a woman as long as they are adhering to taking the ARVs and have a good viral load and cd 4 count. So everyone knows their CD4Count hence your test will just be confirming that the person is taking the ARVs with good adherence.

Interviewer:	How can you feel seeing a man or woman taking part in this trial study?

Participant 9 M:	To me, I can see it as a very good thing because both of these people are taking the ARVs hence there is no difference between a man and a woman.

Participant 3 F:	I feel it is a very important thing and there is no difference regardless of gender because this study will be beneficial to everyone without regard to being male or female.

Interviewer:	Alright. So in the previous study that we conducted about this trial, it was seen that many males showed interest to partake in this study compared to females. Why do you think this could be the case that there were many men than women taking part in the study?

Participant:	For me I feel it was the case because many women depend on the opinion of others in order to decide to take part in the trial study while for men they make their own independent decision. Hence when a fellow woman paints a bad picture about the study that you will be injected with a bacteria then you will have some fear to take part.

Interviewer:	Alright.

Participant 4 F:	Women are the first ones to go to the hospital when they are sick as compared to men hence you will see that many men will be sick for a short period of time before they die. However, many men rushed to partake in this study because they wanted to receive money.

Participants:	….respondents laugh in a low voice…

Participant 3 F:	For me I think most women who are married cannot have a chance to sleep at the observation facility like Grace Bandawe as their husbands might not allow it. Maybe thinking that they will be doing promiscuous activities while at the facility. While for men its easy as it is their decision

Interviewer:	Alright. Any other views?

Participant 1 F:	My view is that maybe the messages about this study did not reach enough women as compared to men.

Interviewer:	Alright. How about participant 9?

Participant 9 M:	I wanted to say the same thing.

Interviewer:	However, you might explain it differently so go ahead.

Participant 4 F:	I feel men are open with each other and may tell their friend when they hear about something while as for women, if I know about the study, I wouldn’t want my friend to take part in it even though I am taking part hence hiding about it.

Interviewer:	Do you have any idea why is it like that for men that they can easily share information than women? Maybe male participants can help us responding to this.

Participant 9 M:	Men are more open to each other about everything when chatting while for women it is difficult as they look down upon each other. Most women might have issues but find it more difficult to share with their friends hence just as we are saying here that not many women took part in the initial study compared to women. The other thing is that even what we are discussing here I can easily share with my wife and she will understand than the women here sharing to their husbands as the approach is different. For instance when the husband asks the wife about this meeting she might not feel comfortable to share the information there and then which might in the end make the husband jealousy and not allow the woman to attend another meeting even though it’s about an important thing.

Interviewer:	Alright. So we are still in the middle of discussing the reasons that you feel were behind having more men than women attending the previous trial study that was done on the pneumonia vaccination. In the forthcoming study on the vaccination, there is an arrangement that women who are of child-bearing age during the study should be on birth control methods. So I would now like to hear about your views on this arrangement that women taking part in this study should be on birth-control methods

Participant 6 F:	My view is that the trial cannot go well when tested on an expectant woman hence the need to have it done on an unexpectant lady so that the results tally with women. So I think it is good to have this trial on someone who is not and cannot fall pregnant during the study period.

Participant 4 F:	For instance when ARVs were being introduced it was said that women should not conceive but it happened that women could conceive and have healthy children. But I think you are encouraging birth-control during this study because you can never know how the child can be if conceived during the vaccination trial period.

Interviewer:	Alright. So do you think this arrangement is good and acceptable to be followed or you think it might disrupt the study when women are told about this?

Participant 3 F:	The arrangement is important because we can never know the implications that can come in if the woman is pregnant and then subjected to the trial study. So it is important that a woman enrolling for the study should not be pregnant. However, I have a question, is the birth-control for everyone or only for those that are sexually active? Should the birth control also be administered to single people when they enroll for the research study?

Interviewer:	I cannot respond to this question In a straight forward manner because this arrangement was put in place as the participants should be 18 years and above. On people who are not sexually active or are single, this idea would be an eye opener to us hence there may be a need to know if a participant is married or not or if sexually active or not so that we determine how to consider these. I can respond to that question that way.

Participant 7 M:	I also have a question. Sometimes it may happen that a woman is on Norplant and feels cannot get pregnant but then ends up getting pregnant anyway. Isn’t it like that ladies? So what could you do if say a person has been enrolled for the study but while in the middle of it gets pregnant while on birth control?

Interviewer:	Alright. So at the beginning of the study, the participants will be tested and one of the tests will be pregnancy test for women in order to ensure that the woman is not already pregnant when enrolling for the trial study. However, in order to avoid the woman getting pregnant in the process of the trial and has been injected with the bacteria that’s why there is the arrangement to be put on birth-control. We cannot deny that sometimes women fall pregnant while on contraceptives but to avoid whatever can be avoided that’s why there is a requirement for uptake of contraceptives by women.

Participant 3 F:	What if the woman has undertaken the permanent birth-control method, will you also test them for pregnancy?

Interviewer:	That’s why we are saying your ideas and views are enlightening us and will be looked at when considering people for this study which involves people from the age bracket of 18years to for instance 45 years which means it might include some people who stopped conceiving so all this is why we want to hear your views on how to go about it and shape the preparations of the study. I don’t know if I have responded well to your question.

Participant 3 F:	Yes.

Interviewer:	Alright. Now let’s come to the section that talks about the processes in this trial study. In your own opinion, what method do you think will be used to get the bacteria into the body of the subject for the study? Is it either though injection or insertion through the nose?

Participant 9 M:	For me I feel the best way of getting the bacteria into the body will be through injection.

Interviewer:	Through injection. Alright. Other views?

Participant 8 F:	Injection

Interviewer:	Alright.

Participant 1F:	Insertion through the nose.

Interviewer:	Alright. We will come back to this. However, for those who are saying through injection, why do you think that will be the method?

Participant 9 M:	For me, I feel like inserting something in the nose might lead you to choke which might make the participant think otherwise and make them feel sick while for an injection it’s a once off thing where you feel the pain once and for all.

Interviewer:	How about the others who have said it will be through injection, why do you think that will be the method used?

Participant:	I feel injection is better than insertion through the nose because with injection will be just at one place like on buttocks while for nose insertion it might be wrongly inserted in a wrong section which might threaten the trial study participants. For instance, during the Covid 19 tests, people were being tested through the nose and some people could say they were bleeding hence could act as a fear factor to others.

Interviewer:	Alright. How about those who chose the nose insertion, why do you think this could be the best method?

Participant:	I have supported the nose insertion because the injection is painful and some people do not inject well hence the place might swell.

Interviewer:	Those who have chosen the injection method, which place of the body do you think could be best for the injection?

Participant:	On the bums.

Interviewer:	Alright.

Participant:	Even on the shoulder or thighs will be fine.

Interviewer:	So how would you feel or react if your friend or relative told you that they have enrolled for this pneumonia vaccination trial study?

Participants 9 M:	I would encourage them if they explain to me well about this study, its importance, benefits and challenges but it would be difficult if they don’t explain well about it or do not know why it is being implemented.

Interviewer:	How about the others? How would you feel if your relative or friend told you they are taking part in this study?

Participant 1 F:	The way we have heard here about its importance and benefits, I wouldn’t have any reason to discourage them from partaking in the study. I would therefore encourage the person to go ahead based on the benefits that have been explained here.

Interviewer:	Alright. Other views?

Participant 6 F:	I just have to add that I would encourage the relatives who have the possibility to be part of this study because we will both have learned about this study, its importance and benefits.

Interviewer:	What concerns would you have?

Participant 4 F:	The anxiety that I would have would be has the bacteria really been healed after the study period or it will come back to haunt me in the future. That’s the kind of anxiety that I can have.

Interviewer:	The anxiety that we are talking about is the one you can have for your relative or friend who would tell you that they are going to be part of the trial study

Participant 8F:	The anxiety that I can have is that if the relative told me they are enrolling for the study and I encourage them to take part but in the end they get to have problems and side effects. They may think that I encouraged them to partake in a bad thing by lying that it is good.

Interviewer:	Remember we are asking about the anxiety that you can have when a relative or friend comes to tell you that they are taking part in the trial study and are just informing you about it

Participant 3 F:	I cannot have any challenges or anxieties because I would know that the person has been trained about the study just like I have been and has thought that its okay to be part of the study.

Participant 6 F:	We cannot stay without having any anxiety about it bearing in mind that this is a trial study hence the anxiety of what the outcome will be from this trial.

Participant 9 M:	I don’t see any reason for me to have any anxiety because it’s a thing that I already went through, and my friend is just explaining to me that he will be going through something that I already have an experience of. Infact I am the one who can encourage the relative or friend because by telling me, it shows that that person has trusted me hence encouraging them that nothing bad will really happen.

Interviewer:	Alright. Do you have anything else that you think we should discuss before we reach the conclusion of this discussion that we are having?

Participant 9 M:	For me I feel like there is nothing else that we can discuss further because what we were supposed to discuss is exactly what we are discussing and have been answering. I don’t know if there is anything else that needs further discussion or more explanation.

Interviewer:	So to conclude this discussion, do you think this trial is something that should really be commissioned. And do you think people will really volunteer themselves to be part of this trial study if we reach out and explain about the importance of this trial study.

Participant 9 M:	Yes. I believe a lot of people can take part in this trial study bearing in mind what we have been trained here on this study. And if we may meet friends, we might be able to explain this to them well so they can understand it, be satisfied, and decide to be part of it.

Interviewer:	Alright. How about the others? What do we have to say?

Participants:	…participants remain quite…

Interviewer:	Now we have reached the end of questions that we had about this study. I now invite any comments or further views that you may have to make before we close the discussion.

Participant:	….participants remain quite without any comment…

Interviewer:	It seems there are no further comments on this pneumonia vaccine trial study discussion. Let me wholeheartedly thank you for coming here to be part of the discussion and being open so we can learn more on the forthcoming trial study that is expected to happen in the near future. As part of all the friend that I work with at Malawi Liverpool Welcome Trust, I thank you all for showing up this afternoon and taking part in this discussion. All the views and comments that you have expressed here are very important and will help in shaping a good arrangement towards this forthcoming research study. Thank you very much.

All Respondents:	Thank you. (speaking together)
