# Supplementary material for: Acceptability of advanced Controlled Human Infection Models: The case of a pneumococcal challenge in people living with HIV and mycobacterial challenge with Bacillus Calmette Guerin in Malawi
Source: PLOS Glob Public Health. 2026 Aug 18;6(8):e0006236. doi: 10.1371/journal.pgph.0006236 (PMC13485124; doi:10.1371/journal.pgph.0006236)
Supplement: Supplementary file 1 [file pgph.0006236.s001.zip › Documents/ACHIMA_RELIGIOUS LEADER FGD_Chichewa_15.02.24.docx]

Interviewer:	It’s a given that vaccines prevent diseases and saves countless lives, but this takes ten years or more to develop new vaccines. We need to find a faster way, one way to speed up the process is by making better use of human infection studies, volunteers are given a sample of vaccine under development followed by a careful monitor infection medical care is always close to hand if volunteers fall ill and if they stay well, it means the vaccine works and can be made available much earlier. The idea is almost 300 years old but using todays advanced technology this type of study is not just scientifically sound but ethically responsible too and of course follows strict rules. Human infection studies show huge promise against fighting diseases like Typhoid and malaria but to develop vaccines most effective at the people at risk we need to test on those at most risk by expanding human infection studies and targeting them better the people most at risk could help develop vaccines better and could save millions more millions lives.

Let’s repeat.

Participants:	It’s clear.

Interviewer:	In short, we can say the video is explaining about the procedure of using what we are explaining inoculating people with bacteria’s that cause diseases in their body to see how the body responds to it. It is known to develop vaccine it takes many years this way they want to start using it as one way to help to speed up development of vaccine be it different vaccines. This way is like a way that can be used to help to develop vaccines faster for vaccines to reach out people faster.

Video play:	It’s a given that vaccines prevent diseases and saves countless lives, but this takes ten years or more t develop new vaccines. We need to find a faster way, one way to speed up the process is by making better use of human infection studies, volunteers are given a sample of vaccine under development followed by a careful monitor infection medical care is always close to hand if volunteers fall ill and if they stay well, it means the vaccine works and can be made available much earlier. The idea is almost 300 years old but using todays advanced technology this type of study is not just scientifically sound but ethically responsible too and of course follows strict rules. Human infection studies show huge promise against fighting diseases like Typhoid and malaria but to develop vaccines most effective at the people at risk we need to test on those at most risk by expanding human infection studies and targeting them better the people most at risk could help develop vaccines better and could save millions more millions of lives.

Video play:	Ok, now let us go into our discussion. Welcome this afternoon to this discussion session where we want to discuss about our research. Research that is called controlled human infection model which in Chichewa we call it research where participants are introduced bacteria in their body. We are MARVELS group from Malawi Liverpool Wellcome Research Program we are the ones conducting this research in short, we call it CHIM. We have plans to extend our work on Pneumococcal to reach out people with HIV. Your views on the research will help us to know the procedures to follow on the research in future. People who can participate are those people who had TB, healthy people, those people who have participated in CHIM study, doctors, nurses’ providers care to HIV or TB, community leaders, religious leaders, community advisory group research experts on HIV or TB and research ethics committee. Meaning to say in our discussion we are having discussions with these different groups as in preparation for the research in future because currently we are just having discussions with these groups as explained that there are nurses, clinicians, community leaders and religious leaders and other groups. Out first part I want to ask on what has been discussed today what message have to go, the message discussed today is about what.

Participant:	I have gathered that you want to do research, there are planning to come up with vaccine in future there is need to conduct research there is need for volunteers to join this research, there is need for different people according to their responsibilities

Interviewer 1:	I have shared pieces of papers with numbers when responding mention, the number for us to know the sequence of the numbers how they were going.

Participant 6 M:	It was number 6.

Interviewer:	Yes, number 5

Participant 5 M:	I have gathered that there is a research that will be conducted looking into those people who are sick and those that are not sick, all the groups are needed religious leaders, community leaders, community members to follow them the aim is after discussing here we should inform people that there is research coming I believe it’s that. That is what I have gathered mainly is that before the research is done there will be gatherings/meetings to discuss way forward how to move on that is what I have gathered before we continue with the discussion that is what I have gathered.

Interviewer 1:	Others

Participant 1 F:	What I have gathered is that as religious leaders we have responsibility to pass the message that there is research coming in future to reach out to older people whether those who are 18 years old. But this research can help to develop medicine or a way to overcome the disease that we can do research.

Interviewer 1:	Good

Participant 3 M:	Yes, I feel like the issue is that the research that is to come is to find vaccine but it has to be developed faster because when there is a research it takes a long time to find medication for these diseases so, this one the vaccine we are discussing will be developed faster I should say so. But because developing vaccine it involves injecting a person with bacteria, so you want to hear people’s views, among us few people is it right for you to do that or not to do that.

Participant 4 F:	I have heard that pneumonia bacteria is that us who are here when we have done this we are accepting this to go like that but when we go in our community (words not audible enough) maybe for a short time for them to understand what we are discussing that becomes a challenge they say they want to finish all of us so maybe if there can be an example or we call it giving testimony that I received this what we are talking about am a testimony am the one you see or maybe I received what we are saying here through giving testimony for the people to believe if the woman is testifying she did not die, she did not get sick many people can accept it to join the research thank you

Interviewer 1:	Good

Participant 2 F:	In my understanding this research is about us who have been trained we should have time to teach others benefits of this information so there is need to mention example as you can see me, I joined that you see me dead. Because most people will look at infecting them with disease in their body it is necessary to show example for people to accept, yes, an example is important.

Interviewer 2:	Thank you very much.

Interviewer 1:	Good, beyond that our discussion this afternoon mainly I should we will be looking in terms of TB, giving a person a bacteria can it has potential to start TB inside the body, this one we are looking at a healthy person giving like on the part of TB. In terms of TB, we know that there is vaccine the bacteria that will be given will be in that form like the way they give TB vaccine. So, when discussing our discussion, we will focus mainly on introducing TB bacteria in the body there have we understood. I want to know on the information that you have been provided today which message do you take as very important.

Participant:	The message that is very important.

Interviewer 1;	Number

Participant 4 F:	Very important message is pneumonia disease in most people, most people are suffering from what we call malaria when they go to hospital, they say it’s pneumonia. So, this is important the disease need to be completely gone for TB it is important there is need to take part because some people are suffering in the communities, but they don’t know it’s TB

Participant 1 F:	For me we should this TB has claimed many lives of people if this research can be conducted it can help maybe to reduce the disease or to completely deal with it.

Interviewer 1:	Good, others

Participant 5 M:	It shows that here in Malawi TB is well established, the purpose of this has to be put forward for people to be given vaccine, it shows that it’s good maybe the disease that is troubling Malawi nation and not only Malawi but the whole world TB and pneumonia is killing a lot of people, people are dying because of that for this aim that there should be this procedure it should be tried it’s good to protect lives of people suffering as there are different areas that are showing there. It’s not only the sick even those who are not the goal is to find out what is going on or to find real vaccine that can protect our lives, I think that is the aim.

Participant 6 M:	I also see that to conquer with my friends as they are saying I also think it can help if people are well informed about this bacteria that cause TB to be introduced in people’s bodies, it is good it can help to remove fear in people in many people on the procedure on medicine development or on rumors that people say that is not true because it has been explained people can be aware of that because maybe other people in the areas know what is happening, it happens in this way or even if other people say I have been given infection (disease) how will it go but if it can be explained it can remove fear

Interviewer 1:	So, explanation, the explanation here this is the time to help each other that the time of explaining for things to go smooth at the beginning of the research. We want you to help us in terms of that part how can the explanation be structured, which ways can be used to support when explaining to people for better understanding.

Participant 6 M:	As I have seen on publication of messages or when informing people to know about this. There are different ways like here you have already chosen community leaders, religious leaders if you can reach out more, they can help informing people who follow them or those who believe in their faith about the importance of taking part in that. Whether they are people who work in business, health sector all these people can reach out to their people easily when doing under-five, for community leaders during the time they are conducting meetings, during registrations for beneficiaries, during funerals community leaders can give these messages like that. Without forgetting other ways of disseminating messages that are there like arranging short radio messages these days we have WhatsApp for most youths putting interesting messages through social media through that people can get the message faster in discussing, asking questions people can know the right information on truth about what is about to take place.

Participant 3 M:	First let me ask before this research has not been done here in Malawi before, trying out vaccine this will be the first one? In the past vaccines were investigated in what way because what is needed here is to convince a person to understand. Am also asking or you cannot try this on animals giving like TB vaccine and trying on it instead of trying in people that can help us because if you can announce this in any other ways or convincing people it cannot work.

Interviewer 2:	Ok, is that a question to explain or

Participant:	Yes, explain so that I can understand

Interviewer 2:	Explaining in brief in the video it explained saying that to develop vaccine it takes a long time for a vaccine to be developed as in the ways that they were developing vaccines in the past we are not sure on what ways they were using what we know is that it was taking long like ten years like TB is bothering us so to get a vaccine it means we have lost lives of many people. This way came in to develop vaccines and things than can save lives faster that we can use an animal it is possible but genes for the animal body and human body they can respond differently. The animal body can get healed the same medicine, but putting the same medicine in a human being not being healed the same medication a huma being developing other problems because our bodies are different. So, this way came in because we wanted to be working with the same person whom we want he should get better that one can give us a true picture of about how things are going in his body. We thought of taking a bacteria weaken it with the aim that it should not have a big effect that can be noticed that is the reason we don’t give real TB to people. We cannot take real pneumonia bacteria the effective one and give a person to see how the person responds to it this makes the person not to get sick. The research that we have been conducting the past if people were complaining it was just sore throat in not more than 2 days, they were fine we want to use your views is the reason we are here discussing, the views that you give we will be considered to do this I have explained clearly.

Participant:	Yes

Interviewer 2:	Thank you.

Participant 2 F:	That is good it’s well understood, HIV vaccine can help many people, children, adults, and the elderly.

Interviewer 2:	Wait before you finish the vaccine is not for HIV but for pneumonia.

Participant F:	Vaccine can help children and adults it’s the same as in the households they are moving testing annual site in children to see if they have pneumonia disease to get help immediately. So, this will do us good when the vaccine is introduced because pneumonia is dangerous most people are dying so if there can be this vaccine to overcome it will do good to everyone.

Participant 4 F:	For our friends who are positive it is good because these people they don’t take long to suffer from cough, they suffer from any illness it comes through pneumonia, cough maybe headache because of their status but if there can be as it is happening maybe they will have care for other illnesses they will not suffer from dangerous illnesses that will be good.

Interviewer 1:	Good, thank you before we continue, he asked a question that he had, other questions that we may have on what we have explained of part of this research, what questions do you have or there are no questions.

Participant 1 F:	My question is to say there, there is research, after the research they will take a bacteria introduce it in the body of a person so there will be another way after introducing it in the person’s body the process maybe how it will be maybe like bacteria getting weak there will be away when vaccine is found maybe it will be injected to all the people I want to know on that, on the message to inform people about vaccine on introducing bacteria in the body it will be done on the other hand or it is like that.

Interviewer 2:	Thank you for a good question. Most times when conducting research people who benefit from the results of research are many more than those people who participated in the research. For example, today we take drugs like Panadol and other drugs there are people that time who volunteered to test the drug on them how they work or all of us today we benefit when we have headache we buy Panadol we take but they did not take the whole community to test on them there were few people. So, there will be people who will volunteer to test on them we will join the maybe we can search for vaccine that can deal with this, and then we will find group of people that can volunteer to test vaccine on them, when vaccine works, we will know that now we can give to the nation it can work. But this we don’t force people to join research or forcing everyone to get vaccine everyone receive voluntarily it’s the right of everyone, but what we want now is to know if this research can be done or not and for the other area that is how it will go

Participant 6 M:	Mien is a question mainly like commenting that the drug that we are using or vaccine that we are using currently has it been tested on other people already in the past. Here I understand clearly if we can conduct the research like this one can it be done or not. I think research on feasibility on this is very necessary because if research is not conducted, we will not get truth for that time or in future. I feel like it is necessary for people to know before you start taking Panadol drug there are other people who volunteered to be tested, I know there are a lot of people who will take part to be tested on them to see how the vaccine works inside my body. Many people would volunteer after being informed what is happening people, people will be told. People will say a lot of things as already said by my mother that the research they want to kill us, that is said because its rumors that people speculate but when people are well informed. Like they come as we are here in a group, I address my group of people how it goes among the people listening some would say they can participate there is nothing that can happen through animals only other things take place through people. So, I think this research is important to be conducted so that you can hear and know what people think.

Interviewer 1:	Good so, want to know you have shared your views that you feel it is necessary for this research to be conducted people have to be informed. Just want to know which other additional information we can give when informing people to make a decision to join the research like this one. What information do you feel we can add on?

Participant 6 M:	The research that you want to conduct about hearing views will be conducted in Malawi as a whole or you targeted maybe some districts that you want to ask, or you will do in Blantyre only.

Interviewer 2:	This is the start we will hear views as we are in Blantyre before going far we want to know views from people who are within Blantyre, based on what you can explain we can proceed with other areas or we end right here from the picture that we can have after meeting several groups but we cannot say we can do in all districts because of resources and other things that cannot allow us

Participant 6 M:	Sorry I asked a question, I did not give response to the question you asked, I was waiting for my friends to respond maybe I will have something to say.

Interviewer 2:	Good

Participant 2 F:	Number 2, for people to understand about this we have to talk about it and we need to announce using mega phone using vehicles in the community for people to hear the message others will hear from community leaders or from other group where women gathers village banks to pass on the messages, and road broadcasting announcement people will hear everywhere.

Participant 5 M:	In addition to that in clinics you need to involve them to be aware, when they know this when a client go to the clinic it’s possible for HSA to give information to people because most people go there, when they go there they can be informed that there is the vaccine people can understand as we do, we religious leaders when we stand to make announcement, because rumors comes they start saying they have changed the system the issue is the same that they have bring a new system the people are from the community they are not from abroad they are the same people who have their pastor /reverend who they listen to them, when we take the message explain to them the message will reach out to people. I want to say sometimes HSA’s distorts messages / information the same health workers distorts information yes. I come from Mbame I will give example, this clear example we have an organization called Evidence for Action it distributes chlorine they take what we call dispensers like buckets placed them in every bole hole, But because HSA’s were not involved during the time they were training on this, HSA’s were telling people that they distribute their own chlorine we don’t know about that from that the issue was they have changed the system, they have bring a new system am giving the example because it is clear we have been discussing about it for some time in our area, we have been discussing with the organization that is a clear indication that when training people we religious leaders and the health center we have to discuss with them, they can go there and be well informed when they come back they can think about it that a vaccine is coming but just staying quite it’s us people who distorts the messages because of different our belief as these days when we see HSA’s are like enemies to people, they say they have come to kill us

Participant F:	They are the ones who start saying am just saying to you, our children we cannot take them to do that, these are people working in the health they are not allowing their children or their relatives not to take part what about us, we will not go that will kill us as what we said, people are afraid to die. The same there was Typhoid vaccine most people refused to take their children saying they will, I went with my child, my child has not been sick up to now or being admitted in hospital no

Participant 3 M:	I’m going to give a different opinion sorry.

Interviewer 1:	All views are acceptable.

Participant 3 M:	Information for research like this one to reach out to people to say the truth not many people will accept it my opinion. It is another way to say research has been conducted and then you want to inject people with vaccine like volunteers that we want to investigate on you. Or even if we can say we want it to be done on us maybe there is not even one who can accept to be injected, or we can accept after going home that will be a topic under discussion you want to be given TB. Maybe it is good it can help it can be done through enforcement but there can be challenges to get volunteers to be injected the vaccine that’s my opinion.

Interviewer 1:	Good, what can we do to get volunteers because I see that as long as the disease is there, we need to find solution and the solution that was there is this one, we thought of trying on people. How can we make sure we get volunteers?

Participant M:	Maybe in few people maybe some will be identified if you can ask here one can volunteer to be injected, the issue is the same, but it will not be easy.

Interviewer 2:	Thank you very much.

Participant 5 M:	I must add he is not lying, he is saying the truth, rumors are a lot , beliefs are more as explained the aim of our meeting is what we are expecting should take place for them to understand teaching them properly the procedures and what we have mentioned others mentioned use of mega phone announcements in moving vehicle, others mentioned having meetings at community leaders, adding on HSA’s not forgetting clinics. Our reading we found that it’s not mandatory not so a person has to join voluntarily. That is a good approach for a	person to accepts to participate it’s not easy.

Interviewer1:	Good than you, what do you think can be benefit of doing this research we call TB CHIM in health adult volunteers. Benefit that can be there conducting TB CHIM research in health adults

Participant 3 M:	This is a healthy adult, he has problems already it’s possible the vaccine that you want to try it, it, possible but because you are trying in a person who is already sick the results will not be good, but you need a person who is healthy to give you real results.

Interviewer 1:	Ok, other views on the benefit that can be there conducting the research how can it help.

Participant 4 F:	Benefit is there because you are trying once, when a drug has been identified it will benefit millions of people that is the benefit.

Interviewer 1:	Other thoughts/views

Participant1 F:	Benefit is there, let me conquer with the mother, trying in two, three people those to benefit are millions of people, government will reduce money spent on drugs that they just give not knowing what they are giving out for, the truth will be known I believe government will benefit from this as well as people.

Participant 3 M:	Let me ask maybe I don’t understand you said benefit that can be there when you try in healthy people.

Interviewer 1:	Yes, benefit that will be there testing it in healthy people.

Participant 3 M:	Not, for the Malawi nation.

Interviewer 1;	IF they try it, if it goes on well, it means it will be given to all people.

Participant 3 M:	Ok

Interviewer 1:	So, let’s look at benefit on few people when something is starting, we start with a small group representing the rest, how will it benefit or the benefit that will be there that these ones volunteered to do this that is the benefit, or there is any other benefits.

Participants:	That’s the one

Participant F:	Let’s take for example someone with TB or be it is HIV positive suffering from cough that we have mentioned if the patient is tested on that through it that one will benefit, to the people they will get care/ support.

Interviewer 1;	So, let us come on the issue of risks that can be there. Risks or hinderances that can be there conducting research on TB CHIM. What risks can be there or that you can think of

Participant 6 M:	I feel like maybe it can be like that a person who was healthy example we have tried on a person who was healthy, maybe he get sick we never know when sickness can come, or maybe death is unpredictable that can tarnish the image of the research if we try it on a healthy person maybe the person die, that can give a bad picture on the work that we have done.

Interviewer 1:	Ok, there are any to add on what number 6 has said.

Participant 1 F:	In my understanding, or my opinion I feel like the risk/ hinderance that can be on this research to be conducted, on giving information to people sometimes it is the information/ message that can promote vaccine because of the way the message/ information is being disseminated. Because when the message/ information starts from the ground telling people about the thing, when the thing comes acceptance is not an issue, example just coming today telling people as my friend has said	that you have not announced vehicle moving around announcing of the research, you just get there and say there is vaccine people will not accept it. Acceptance will not be there but if the message about research was announced and, in the end, it was mentioned that there will be a vaccine, now people want that you should start with them step by step just coming to them that there is vaccine at school, children have to receive vaccine no one can accept. I feel like the hinderance can be that if people will not be approached from the bottom that they have that at the back of their mind.

Interviewer 1;	Ok

Participant 3M:	I feel the risk can be there if the research is conducted and comes with results because when conducting research, we are just investigating if it can be practical, or it cannot be done. So, I f the research is conducted but we did not get what we wanted it means for other research studies it will be hard for them to be conducted.

Interviewer 1:	Ok so, when you weigh on benefit and risks of this type of research TB CHIM where we give participants weakened bacteria for TB, which side between benefits and risks do you feel is outweighing the other.

Participant 3 M:	Benefit of trying we should say because for a person to do a perfect thing sometimes you need to take a risk, you need to try it out to save lives of people in future I can say benefit is on higher part.

Interviewer 1:	Other opinions/views when we outweigh benefit and risk that can be there, what are your views.

Participant 2 F:	My opinion is that when you try on them, when they inject you hindrances will be there that where are you going you want to die before reaching 50 that will be the hinderance most people will not come because of their friends, people are easily taken by peer pressure of friends, friend I want to go to do this on me while on the way, yet he has come with the decision from home that am going to be injected he meets a friend he return on the way going back home did not go the clinic that is the hinderance

Interviewer 1:	Other research studies of this type have been conducted here in Malawi using pneumococcus bacteria that causes pneumonia in healthy adults those that are not sick how can you differentiate that research with this one doing it in people who have HIV.

Participant 3 M:	It’s different because people that have HIV their immunity is low, so someone with low immunity it is not good to try/ test conduct this research on them I think I can say that.

Participant F:	Trying it should be in other people, maybe they will benefit because it’s cure for everyone, everyone will benefit like for those who are weak when you do that to them, they can say that you have killed our person he was ok, it becomes difficult, yet he was already sick.

Participant M:	Kikiki you have killed our person.

Participant F:	Yes, it goes that way, yet he was already gone.

Participant 6 M:	I just want to comment on what she has said that it is true but because research is safe it had to be done, they are monitored closely I believe the risk cannot be there, when the bacteria is introduced in a person’s body there are other things the bacteria has been weakened or if there are other hinderances that a person is facing treatment is nearby. Trying in people who are sick is ok because disease causing organisms does not choose it can go to a health person or someone who is sick, there is need to try to see in these who have HIV but they don’t have TB, others are both HIV and have TB when you try you can see how it works in these people it will help when results can give a good picture or good direction on how these people can be approached and those that are healthy. That can give a picture and direction on how people can be injected, or people can be introduced to these. Because when it only goes to healthy people what about the other people how will you know that this group need to be tried on. I remember some time back when we were in secondary school we were told we should try having gardens, the other garden we put fertilizer, the other one we put manure, the other one just leaving it without putting anything, three gardens we saw that where we put animal dung without using fertilizer, the other we used fertilizer, the other one just leaving it like that, the other one putting tobacco and other things how can it be all that is trying which is good and what is not good, we should try it.

Interviewer 1;	So, lets come to risks that are like risks, the risks that you have explained how we can explain them to people, how can we explain them to people.

Participant M:	It is not clear, maybe in my head.

Participant F:	Explain clearly.

Interviewer 1:	Ok, we have talked of risks that can be there in conducting this type of research so, I was asking that for these risks how can we explain about them to people.

Interviewer 2:	Ok maybe just to clarify saying maybe for a person who is HIV giving him like the bacteria maybe he can get sick my mother responded that maybe the person has died people can blame us that we have killed a person. So, when every research is being conducted there are risks that we expect that maybe this might happen or not. Now we know that this can happen we want to be telling people about what can happen we will not hide for them that after introducing a bacteria this can happen because we want a person to make decision that he has made after being given full information about the research. So, we want to find away how to explain to the people not to scare or encourage that the research will move like this, that is what we want to discuss on how we can tell them for them not to be scared but we should not hide any information for them, the question is clear.

Participants:	Now it’s clear.

Participant 3 M:	Maybe approaching them in the way we were talked about convince them but still telling them everything and that it’s voluntarily if the person feel can support, can help if you feel you are scared you cannot participate, we are not forcing I think they can understand in that way.

Interviewer 2:	Thank you very much.

Interviewer 1:	Ok, we can continue so, now want us to discuss on the other section where we want to explore about interest participating in research between males and females. We want to discuss on interest that people show between males and females show in participating in research. How would you feel if a female or a male want to join/ participate in this type of research like this one CHIM where a person is given a bacteria?

Participant M:	Feeling as in a person is your wife?

Interviewer 1:	Let’s take it in general as a man or a woman.

Participant M:	How everyone can feel individually

Interviewer 1:	How would you feel if like I go to take part in this type of research for any person a man or a woman. How would you look at that differentiating between males and females taking part in this type of research. You can see that there is any difference or not.

Participant M:	There is no difference, both are lives same blood if you put in the nose, it’s the same there is no difference.

Interviewer 1:	There is no difference.

Participant M:	No difference.

Interviewer 1:	Others what are we saying, there may be differences between female and male participation in research like this.

Participant 5 M:	There is no difference but the way we think is different because the research is for both sides males and females but there is no difference, thinking level pf a female and a man are different you see that most hospital based research mostly women take part in large numbers, research are for women because men are gentlemen they don’t want to do that. I moved with a research group in my area women know better about children vaccines, there is measles she joined research was given vaccine the woman got the vaccine but when the man heard he did not allow his wife, he said I don’t want that at my house you have come with other things because a man and a female they use different thoughts. But when we talk of research there is what difference no, research has to go to both sides males think in a laugh.

Participant 3 M:	Sorry, I have other thoughts I said I think there can be a difference based on a condition a person is like women sometimes they are expecting.

Interviewer 1:	Sick?

Participant 3 M:	As in expecting, maybe we cannot try on a person like that one because we will put at risk the child, men we just stay all the time we are like this, we don’t have conditions we are like this maybe the difference can be on that.

Participant 5 M:	To add on what my friend has said I will give another example there is a certain medication we call it Meclizine, meclizine they started distributing in 2000. People have been receiving this Meclizine at the beginning people were unwilling, but these years people have accepted that Meclizine is good. When giving Meclizine they don’t give to pregnant women, critically sick patients they don’t allow to give critically sick when you Meclizine and they die they say you have killed a person as she said they don’t allow that. So, in this research there is a difference and that a woman can be expecting you cannot that is true.

Interviewer 1:	So, in our previous work should say in the previous research for example we saw more males that females are the ones who joined research of this type, what do you think could be the reason that made more males to show interest in participating in this type of study.

Participant 6 M:	Reasons to have more men during that time can be difficult we cannot say it was this what happens in the areas or should say approach in the area, how we will approach this time is what will make more men or women to join. I see approach is what convince them to join that research.

Interviewer 1:	Mmhi

Participant 6 M:	So, maybe we find a good approach to reach out to people that can make men to be more, but the issue is about rumors if these rumors are well explained as my friends have said it can do good, we can reduce rumors but the main this is this. When information or message is properly disseminated, I believe it can make people to join 50 percent men 50 percent women and even youths’ males and females depending on the age that you mentioned.

Interviewer 1:	Mmhi ok, lets continue in the research that is coming in future there is a plan that women of childbearing age those that are ready to participate in the research they have to practice family planning methods during the time they are in the study, when taking part what are your views on this point.

Participant 3 M:	It’s a good point understandable when we talk of research we are investigating on something which we don’t know its answer so to prevent complications that maybe a person can be expecting and then she joins and then falls sick that can be a bad thing that is why there is that plan to be provided with family planning for them not to be disturbed with other things

Interviewer 1:	Ok, women what are your views, how do you look at that?

Participant 2 F:	We see that a person who has to take part should not be expecting but they should be given contraceptives for them to take part, because she can join maybe she has passed 3 weeks and has conceived but she doesn’t know that she has conceived so it will be like she started another problem, but they should be on contraceptives, and they take that

Participant F:	The question is coming that an expectant woman can join.

Interviewer 1:	Now we are asking about a woman who want to take part in the study the time of joining study she must follow family planning methods what are your views on this type of arrangement. It can be a good way we want to hear your views/opinions.

Participant M:	It is a good way because it is better that a person joining research study should join on her own you said it that everyone joins voluntarily without being forced. When you mentioned of 18 years it means someone who can think properly, a person who has decided to join for someone to join while expecting the person inside did not have a say if something can happen to that one it is better for a person to join research study the way she is let me agree to that.

Participant F:	That is good.

Interviewer 1:	Ok so, as part of this research people participating in the research the first few days, they are sent to stay at research residential site. What do you think about the arrangement that the first few days when they have been given the bacteria, they are sent to study site for monitoring. What do you think on this arrangement?

Participant 3 M:	The arrangement must go like that, but it is difficult let’s say married person to leave his /her partner who has not been involved with decision making that am participating like to leave a partner like for a week staying out. I think it will not only affect the one participating, but it will affect the whole family that is where the story is getting twisted because other cannot accept for their partner to stay away that is the problem.

Participant 1 F:	I see that is difficult maybe there can be for example where they stay someone saw that is missing and that she/he has gone to stay somewhere people can be talking, it will be like we have underestimated him or that what he is doing is not important. But for a person to be protected against talking if there could a way as she has said earlier that for married couples, they need to discuss man and woman to agree to give a go ahead, but I don’t think what I can do my husband can accept like this is difficult. If there can be another way or his/her home maybe doctors or the research team to monitor the person on daily basis but not the person staying there for the whole process that can be difficult.

Interviewer 1:	Other thoughts/views

Participant 5 M:	The opinion is the same to take participant to stay in isolation that is hard we can end marriages.

Participant F:	Where they stay is it at the hospital or at a certain place, because they can be saying he is sick has been admitted at hospital.

Interviewer 1:	Here we are not saying a person is sick we are just keeping them at a safe place to monitor them the first few days after they have been given these bacteria for them to stay at a place close to a hospital in case something happens there should be a clinical person close who can attend to them if need be, to take them to hospital

Participant F:	Keeping them for monitoring.

Interviewer 2:	In addition, the arrangement of sending participants to study site we don’t keep them at hospital, the research that we conducted in the past. After giving them bacteria like today we were keeping them at Grace Bandawe maybe others we know we were keeping them there every evening for three days. They go there around five and come back the following day around 6 or 7 going back home for them to do other things. The main reason why we keep them the first few days we take them as the critical days after introducing bacteria in their body other things can change. The second thing is that at home it can happen neighbors’ children getting close to him he coughs on the child, yet you are safe your immunity is ok but the one you have coughed to can get sick even	you have received a bacteria your partner a can get sick you have received TB you cough to your partner you are putting him. Her at risk for these reasons we said why don’t we keep them for few days we monitor them. So maybe we were enlighten based on these points.

Participant 5 M:	I have a question after doing that there was not any problem that you found.

Interviewer 2:	Maybe because openness is a problem on family issues but there are not any reports informing us that at home there is this problem that is the reason am saying openness that there is this at home because I joined a study, they cannot tell us maybe problems can be there, but we don’t know.

Participant 6 M:	As my friend is asking that there is nothing that happened when you kept people most of the time when people are joining a study if the information get to families properly they make decisions for men and women who are married they agree on who should join if it’s a man joining the study the wife is aware that the man is joining a study if it’s the wife joining the man is aware most times. But if its children joining their parents are the ones who can give consent for them to join the study. Like for me my child joined a study at Queens when he was young about HIV, he stayed there he was staying right there I don’t know if it was you Malawi Liverpool or John Hopkins, I don’t know which organization at one time he was in a research study about HIV for older children that time he had started speaking now the child is in third year at Malawi College of Health Sciences studying medicine, he remembers that he joined research but it was that we had to discuss myself and the mother that the child want to join a research I told her as long as there should not be a problem. So, for all the time he was at Queens he was admitted I don’t know I did not follow for the time they were there I was I ware that my wife is in a research study. But I feel like the aim of disseminating information here should be established clearly there should be enough awareness to ensure that there should not be any problems for those people joining the study with the issue to do with families, families have to be aware when one is joining the study, when you join these are the requirements so that when a person is joining the study the family has to be aware before the time of making the decision

Participant 3 M:	Just to add on family issue sometimes maybe a person can accept after seeing the benefit as in a family because you understand that when my partner joins after the study you will get something you can tell her to go. But if there no benefit let me not lie that can be a problem on this issue to take a person to stay there yet you know you will be here you wake up you to put water for bath by yourself knowing that my wife will come with nothing, I feel that can be a problem, but when you know you will benefit

Interviewer 1:	To show that accepting here, accepting on condition that there is benefit.

Participant F:	Yes, that she will come with something.

Participant M:	You will be prepared for something.

Participants All:	Laugh

Interviewer 1:	Ok,

Participant 5 M:	I agree what I said that there was research in my area a person was told that because of time we were going around listing after we finished listing, they come selected families. We visited a lot of people when choosing they selected few families at first the time when we were listing there were rumors. They are bringing disease people were run away. The research targeted children. When they come with list of families selected after they have finished the discussion with them they were receiving K5000 for we have delayed you, you waisted time yet it was at his own house they were receiving K5000, the people who run away when they heard that they were receiving money, they said how was the selection done, why did you choose few people were selected this was because of K5000 I agree with my brother this is true

Interviewer 1;	Ok, still on the same on the part of a person participating, just want to know for a person participating who can they give as their representative (proxy) that when there is anything that one can be contacted for example when the one who joined study is needed but the research team cannot get in touch with him his phone out of reach, who can be listed as a proxy who can be contacted to pass the message to participant.

Participant 3 M:	That is a person who lives with the other person, or neighbors.

Participant 4 F:	Yes, or your child you can, don’t hide tell that one he can make it happen.

Interviewer 1:	Ok so, on the child which child should this be as people we have children some, we live with them others are living elsewhere, which child between the two here

Participant 4F:	Let him be the one you live close to not the one far away.

Interviewer 1:	Why do you say the one living close by?

Participant F:	We live together, am going to this place I have been called to do this he has to know where parent has gone and what is doing. When you call if you make phone calls, he will respond he will rush to where you are.

Participant 5 M:	To add on that when you join a study the child has to know, your husband has to know so on the point of messages this cannot be a problem because you have informed the child but when you put any other person it cannot be good.

Interviewer 1:	Ok so, let move on to the nest section here we want to discuss about study procedures, which we are taking it as TB CHIM where people are given bacteria for TB. As one way of the study participants will be injected with BCG as a way of introducing bacteria. This BCG is the same as the one given to children here in Malawi in the vaccination policy, but this BCG that will be given as part of the study will be given at different doses and times to be able to collect body tissue from the site of injection to conduct test to see if BCG is still on the injected site and other samples blood for testing the aim is to see if BCG is present in the body. How would you feel to be injected with BCG?

Participant 3 M:	Let me ask maybe I can get rid of ignorance, I hear of pulmonary TB, TB of bones I think it’s what I have heard I don’t know if these TB’s the vaccine is the same that we are discussing or there are different are bacteria’s that are given.

Interviewer 2:	Responding to the question the bacteria that cause TB is same it can go in the lungs and TB detected in the lungs or TB cannot be detected in the lungs but detected in bones that can be possible the bacteria is the same it’s just the place where it attaches itself is different.

Participant M:	Ok, we have learnt.

Interviewer 2:	Thank you.

Interviewer 1:	I was explaining that as in procedure participants taking part in the study will be injected with BCG which is like the vaccine given to children when young, I believe all of us here we received this type of vaccine. This one will be given to older people will be given at different doses and they will take skin sample from the injected site for testing to see if BCG is present and they collect	blood and other samples from the body for testing to see if BCG is present. So, I asked a question how you would feel about being injected with BCG. Here we want your consensus about how you would feel to be injected with BCH.

Participant 6 M:	Any injection is the same the way like how we receive vaccines, so testing as well is the same as you can test like for me the other time I went to Queens you tested for TB, HIV Aids, BP testing is testing there is nothing like how we can feel it’s the same as health care workers do their work it can be the same. If it’s just injecting you feel pain you complain a little that you feel pain in the same way, there is nothing sweet that can be at getting vaccine like BCG there is not any sweet

Interviewer 1:	Ok

Participant 2 F;	There is no problem to be injected because people we get injection, but the danger is after injection taking the fresh. What kind of fresh are they going to take, when removing fresh you will not feel pain

Participant:	They go away with piece of skin

Participant M:	On the issue of injecting BCG let me ask does vaccine expire because we receive vaccine when we are young children is it the vaccine expires that it is no longer working because everyone receives BCG.

Participant 1 F:	To add on asking on what you are saying on BCG I believe everyone received this BCG vaccine, it is helping in terms of it can be vaccine for research study that is taking place. What work is it doing that we should be injected the vaccine, or it can be the one that can reveal that this person is like this or how can I explain. You want to see If this BCG can be one part that can support on that on maybe I don’t understand clearly.

Interviewer 2:	Thank you for the good questions maybe when clarifying we explained that young children after birth everyone receive BCG vaccine. What gives us question is that despite all of us receiving BCG vaccine still more people are suffering from TB now we are trying to ask ourselves questions does this BCG have timeframe that from this point or maybe is it the food that that we consume that makes BCG to stop working in our bodies. So, we want to see what the problem is, to see if the problem is BCG that is provided, or the problem is our bodies that is the reason we are conducting this research because we cannot just come up with another vaccine while the other one is already there. Starting point will be on the same BCG The one we receive that is the reason we are saying we will inject them with BCG, and take part of fresh from the injected site, the fresh that we will be getting is very small that you cannot touch it, we just prick its very small to check if BCG is present after its given is it present in the body. The question we want to know even if people receive BCG, we are still suffering from TB so, we want to try testing the vaccine.

Participant 4 F:	What scared us is about taking part of fresh that is what we were wondering why children after receiving BCG vaccine where do they take part of fresh that is what I meant so, because it’s very small you will know the plan how to take it when moving syringe out, we cannot know so that the wound does not take time to heal

Interviewer 2:	No, there will be no wound up to dressing it, it’s not a wound.

Participant M:	Thought it’s 1kg (all laugh kikikiki)

Interviewer 1:	Ok, let us continue still on the same BCG just want to know which place you would prefer to inject this BCG, now we are talking about the research the place to inject.

Participant 3 M:	Better on the arm where they inject us when we are young children, maybe we can just go below a little bit because the scur is visible, they can go beneath it.

Interviewer 1:	Ok, other view/ opinions

Participant 6 M:	Let me agree with that one it should be on the arm.

Interviewer 1:	It should be on the arm ok, what do you think on the issue of biopsy on what has already been said anything that you raise what is a concern on taking of biopsy to conduct a test.

Participant M:	There are other conditions like Diabetes they say that they should not have wound are these people encouraged to participate in this because you want to inject even a scratch does not heal, I don’t know on the issue of these people are they encouraged to join.

Interviewer 2:	Ok thank you very much for a good question. When conducting research, I will be basing on the research study that we have been conducting previously. We screen healthy people to check them we screen them of all diseases that can out them on risk where there are illnesses like Asthma, BP, Diabetes all those people with these illnesses we don’t encourage them to join the research study because we know they can have other problems like what you have explained like a small wound getting worse all diabetic people and other illnesses that I have explained we don’t allow them to join. Or you had other opinions maybe we can allow them, and we do this.

Participant M:	No, just want to know will you allow them.

Interviewer 1:	Lets’ go on still on the issue of BCG, there are several ways of introducing BCG in the body, one pf the way is by putting BCG in the lungs this method uses a tube that pass-through nose or mouth passing through the throat up to reaching the lungs. This methos is used to collect sample from the lungs what are your views on this procedure.

Participant 3 M:	This way on my part is not a good way unless if you say like the drugs that we take, there is injection people prefer injection saying injection works quicker and so we prefer that way we say we prefer injection over tablet drugs for them to get dissolved unless if this method has special importance over injection, I don’t know but if there is no any importance its better injection

Participant F:	Injecting

Interviewer 1:	Ok, other views/opinion

Participant 2 F:	My view is the same what has been said injection.

Interviewer 1:	Injection this way of putting through pipe in the lungs no to this, what are your concerns on this way.

Participant 3 M:	This way, lungs are delicate considering that I don’t know where the pipe reach inside we are talking of the inside of the body if there is another way they can inject, a person can be scared for a pipe to get in the lungs, here maybe health professionals if you feel there is special importance of doing that unlike injection but otherwise I see injection is better

Interviewer 1:	Let’s continue here I want to hear your views on the issue that your friend or relative come to you telling you that he wants to participate in a research study like the one we are discussing TB CHIM or study where participants are given bacteria BCG what can you say, or how will you feel.

Participant 4 F:	Someone come to tell you, a relative that am joining research I will go there to see. That is all his right and decision, we cannot deny him we can just encourage.

Participant M:	But dint forget to pray you will be given bacteria mention God all participants laughed.

Interviewer 1:	Other views/ opinions your relative, I have come telling you my brother I have joined the study how can you feel.

Participant 3 M:	The issue is the same we can encourage but pray life is unpredictable.

Interviewer 1:	Other thoughts or all of us we on the point of encouraging.

Participant F:	Just encouraging him.

Interviewer 1:	Encouraging him, you will encourage, in what direction will you encourage him, I should say that.

Participant F:	The person has selected be it injection or biopsy he has selected, he will go by his choice be it he has selected pneumonia or TB the choice is his that he is joining to for pneumonia or TB let’s not deny them because tomorrow he will say you denied him good things, he will complain that he could have been better off.

Participant 1 F:	For me if he is my relative, I can encourage him/her for choosing correctly, as I know the thing already, I have nothing to tell him don’t go there no but encouraging him.

Participant M:	When things go wrong it will not affect me, people laughed.

Interviewer 1:	Oo, when things go wrong that should not affect you.

Participant F:	Encouraging him sister, brother go because if you tell him that is not good that means the research will not be possible, but there is need people who are courageous enough to lead them for their courageousness to be seen because without people volunteering the study will not be possible.

Participant F:	Will not be possible.

Participant F:	A goat cannot volunteer but a human being must volunteer.

Interviewer 1:	Thank you, what can be your concerns about a relative, friend taking part in research like this, what is the concerns that you may have.

Participant 6 M:	Concerns are there because the person is given bacteria in his body, because of the word putting bacteria in his body that can be the concern.

Interviewer 1:	Its bacteria a virus is dangerous.

Participant 6 M:	Yes, bacteria that is what can be the concern to a person.

Interviewer 1:	Concerns can be in which area, we should know the concern can be in which area, that they get sick or in terms of consequences that can happen to the person the concern will mainly fall on which area.

Participant 3 M:	Concern about the disease can it not happen that he has been treated but later in future we found that it has reoccurred as he did not have it but you introduced it, it cannot happen in future. Because it is possible that am healed after some time start suffering seriously because the bacteria were introduced in the person’s body concern will be around that

Interviewer 1:	Ok, thank you another concern.

Participant M:	The concern is that I want to agree with my friend the concern is the same will the person not die. But I can say that I will not be much worried because we have been enlightened that people will be safe as they will be monitored with doctors closely as you have explained we know how it will go there is nothing to worry about mainly.

Participant F:	He said we should have faith yes, we should not doubt.

Participant 6 M:	I did not understand there can be many problems but the way I have understood here it’s clear because you want to take them at a separate place for the reason to avoid spreading to others if there can be a risk that is understandable and clear. We can encourage because you know the benefits, sometimes we don’t allow people because we don’t know what the person is going to do.

Participant 1 F:	Just to add maybe it can be good because the bacteria may be inactive as you put it for the person to go there we can still be worried the thing is we can pray telling God that these are this is for testing it should not turn to be real because I believe, I believe that those who believe the word of God the same even the Israelites the time they were in the wilderness Mose told them when they get sick they should look at snake made of bronze, when the bacteria get in the body God keep my life because we have to pray

Interviewer 1:	Thank you very much we are at the end of the questions that we had comments from all of you one by one on, what is your comments on the discussion that we had this afternoon.

Participant 2 F:	To comment on what you have taught us, saying you will take the person to stay with them you will test them, when they have high immunity, the bacteria will die inside and that you will test to see if the bacteria is still there or not that is the benefit that I have seen to comment on.

Interviewer 1:	Another comment lets go round.

Participant 3 M:	Let me appreciate saying that it’s a good plan to reach out to people on issues like these because there are times maybe people conduct research secretly without people knowing, researching on a person without his knowledge that you are conducting research. Let me appreciate that before coming with this vaccine or research you have identified people to hear their views, I must appreciate that mainly.

Participant 1 F:	I appreciate too, after seeing that you cannot just do this on your own you thought of reaching out to people when you thought there is need to do this research out have invited people, I believe we are not the only ones representing different churches. This is a good thing let me agree with others saying that there are other things that are done without people’s knowledge when people know about that if the thing are for them to receive in their bodies most people refuse to get because we don’t know where its coming from and where it is going to end on that I must acknowledge for bringing this in a right way even when people will be approached it will not be news because some of us we will be already aware of it, thank you

Interviewer 1:	Another comment

Participant 6 M:	I just want to thank you that God who gave wisdom to people in the brains that we must use them. It will take from how we use the brain to help other people. We know that all the ways that are used by health professional are ways that God gave wisdom that is showing this, even though we hear some speculations to tarnish e people but mostly it is to help people because of wisdom that God gave us. We should pray for it to continue for many people to get peace to be helped on different problem that they face. One day when Jesus was walking he came across a blind man he tried doing research scratched mud and put in the eyes of a blind man when he saw it was not working he asked what can you see he said I can see people like tree that was trying out something to see how it will go later he put his hands on his head that was a research, research are different so the man started seeing clearly. Research began by the creator of universe we must be proud of it. I was talking about this the other day with someone in a meeting with other leaders who are pastors during Covid time people were taken to isolation around Kameza round about people blame that people that were sent there were to be sold, the people will be just miss that rumor maybe us here we should avoid rumors, we should say the truth we should love to know truth Lord God Jesus did not allow someone found with leprosy to be among others they were taken put outside the village the old testament did not talk about that, people with leprosy were taken out

Participant M:	It was a law indeed.

Participant 6 M:	Leave him somewhere far God said that so isolation that was happening started with God but because its being done by health personnel we blame them but it’s in the Bible. Thank you so much that you thought that you can find believers who can hear your thoughts, that we can pray for you to find a way better ways for people to be safe from pneumonia and TB to save millions of lives coming in future for them to live healthy, for us who we are already done but for those coming in future God to give more wisdom.

Participant 5 M:	First let me thank you from the bottom of my heart for our gathering. In the communities we have several community leaders who are under the leadership of Traditional Authority, Group village headmen. At first traditional Authority first talk to them like you are warning them. If possible, call traditional leaders with their group village headmen that we want you at your office those people they have an office. You discuss with them their group village heads will inform their community leaders that can be good. Don’t rush going in the community leaving these ones please I want to ask from you when you will be starting the work when we go back, we will inform them what we you have told us, you know a lot of people don’t know others even don’t understand the Bible In our communities they tell each other lies that they are taking drugs. There is another group of people who don’t go to hospital if you read Exodus 23 verse 15 there is words saying all male children were to be killed but God did not want on the issue of law all male children were to be killed but they were afraid the result if you read the same male children are the ones who said all Israel females when they go to hospital when they deliver a male child give them meaning in Israel they were going to hospital that is where people have different opinions. In our village areas even in town there are some people who say going to hospital is sin they are building on that. I want to thank you for inviting community leaders we are the ones taking care of the flock we will tell the people that this is the work. I have a question I want to ask finish I will ask later. What way are you using for people to come to the hospital, there are volunteers or what we don’t know who the people who you will use on that let me stop there when you answer me, I will go to the village?

Interviewer 2:	ok, for people to volunteer to come to the main study.

Participant M:	Yes

Interviewer 2:	What we have discussed here we have agreed to give message to people, views that we can use using megaphone maybe going to community leaders, HSA explaining to them clearly what you have told us is what we will use. We will explain to people after understanding clearly, they will come thank you.

Interviewer 1:	Ok, there are no additional comments.

Participant F:	The comments are these ones they are enough; we appreciate that you remembered to invite religious leaders to come to discuss one or two things because you could have done this without us knowing but you, but you saw it there when you sat down that to sit down to discuss if this is feasible or not. So, we have seen that this is good and feasible, when you need to call us on other things, we will be free to come to listen, but this is possible thank you.

Interviewer 2:	Thank you, speaking from the bottom of my heart from Malawi Liverpool Wellcome Trust I want to thank you for coming to this gathering today. I know being religious leaders and fathers and mothers you had a lot of things at home that you could do. Some of you have left sheep at home you have spared time this time to come to discuss about our health. There are some people as pastor said that they take spiritual and physical life when you tell them to discuss about health care and research, they would not agree it would look not Christianity to them. Thank you very much when we work with religious leaders like this it makes us very happy because we know that our lives all aspects are balancing, but if we just use the body our spiritual life will suffer. So, next time when we call for you come to help us come up with decisions because for us to make decisions as heath personnel we mostly	depend on religious leaders. So, from the bottom of my heart thank you very much for the time that you gave us today God may the Lord restore everything that you have used, thank you very much.

Interviewer 1:	This is the end of the discussion this afternoon, thank you very much.

Participants:	Thank you
