# Supplementary material for: Acceptability of advanced Controlled Human Infection Models: The case of a pneumococcal challenge in people living with HIV and mycobacterial challenge with Bacillus Calmette Guerin in Malawi
Source: PLOS Glob Public Health. 2026 Aug 18;6(8):e0006236. doi: 10.1371/journal.pgph.0006236 (PMC13485124; doi:10.1371/journal.pgph.0006236)
Supplement: Supplementary file 1 [file pgph.0006236.s001.zip › Documents/ACHIMA_TB CARE PROVIDER_FGD_02_18.01.2024(1).docx]

Interviewer 1:	Ok so we welcome you to this interview in which we will be accessing discussing controlled human infection in people with HIV and healthy volunteers. We are MARVELS group from Malawi Liverpool Research program conducting CHIM studies. We plan to extend our Pneumococcal challenge work to people with HIV and use Tuberculosis Challenge in healthy adults, your views on the planned work will inform planning of future research studies so for today in this discussion we will focus discussing, seeking your views whether it’s acceptable to do a challenge in HIV negative adults. So, we will play the video to give you the concept of what controlled human infections are or in short CHIM, and then my colleague will explain the video so that you are able to get the concept is that alright?

Participants:	Yes

VIDEO PLAY:	It’s a given that vaccines prevent diseases and saves countless lives, but this takes ten years or more to develop new vaccines. We need to find a faster way, one way to speed up the process is by making better use of human infection studies, volunteers are given a sample of vaccine under development followed by a careful monitor infection medical care is always close to hand if volunteers fall ill and if they stay well, it means the vaccine works and can be made available much earlier. The idea is almost 300 years old but using todays advanced technology this type of study is not just scientifically sound but ethically responsible too and of course follows strict rules. Human infection studies show huge promise against fighting diseases like Typhoid and malaria but to develop vaccines most effective at the people at risk we need to test on those at most risk by expanding human infection studies and targeting them better the people most at risk could help develop vaccines better and could save millions and millions of lives.

Interviewer:	we watched the video

Participant All:	Yes

Interviewer 2:	In brief what the video is telling us as we have seen on the issue of Covid without vaccine, Covid couldn’t have been contended, so what they are saying is most diseases people are protected when we get vaccine. So, to develop a new vaccine is not an easy thing It takes years to develop a vaccine that can work effective as intended as it happened with Covid. Imagine during Covid we were still waiting for ten to fifteen years to develop how many people could have survived.

Participant:	Few

Interviewer 2:	So, following that close monitoring of disease is close by all the time if volunteers fall sick, but when they don’t get sick it means the vaccine works. One way of investigating about these procedures is by studying the disease in volunteers who are given a sample of the vaccine that has been developed. What we mean is that to speed up the process to develop a vaccine there is need to identify people who are not suffering from the disease, give them the vaccine later give them a bacteria that works on the vaccine and monitor them in a controlled environment. When people get sick, they are not controlled but when doing a CHIM study it is controlled there are responsible nurses, doctor’s, laboratory, it’s a controlled environment the person is followed up in order to make sure the volunteers does not get sick. In CHIM studies when we are giving a person the bacteria our intention is not to make the person sick, are we together?

Participant All:	Yes

Interviewer 2:	But we want to observe them when they have the bacteria what happens, what happens to immunity that information will help us to develop the vaccine. So because it’s a controlled environment people are trained, people have experience things happen faster than waiting for a normal way for a person to get sick and then to start developing for over ten years, are we together?

Participants All:	Yes

Interviewer 2:	Studying about diseases brings a huge hope in fighting against diseases malaria recently you heard about Typhoid vaccine it was also the same way in other countries they conducted malaria CHIM they were giving real malaria how we fell when we get sick doing it in a controlled environment see if the vaccine can work. All the vaccines which we are enjoying now some where someone volunteered to be tested on the vaccines, are we together?

Participants All:	Yes

Interviewer 2:	We need to test them for it to work better, I have said most vaccines come from abroad for vaccines to work effectively vaccines have to be tested in people who are the target group, and we are saying there is PCV13 vaccine given to children yes, it’s available here in Malawi but does not work exactly as expected because it was not tested on us, Europeans volunteered so we already know that genetically other things are already different. So, our goal is that we want in future to be able to produce vaccines which were tested on Malawians, Africans in that way we think we will make vaccines which will be better for our generation you understand.

Participants All:	Yes

Interviewer 2:	Generally, that is the summary of the video to develop a vaccine how long does it take. But what is the better and fastest way is CHIM studies so that is what we are discussing today. As explained by the doctor we are MARVEL’s team we conduct different studies but for today our discussion focus on people suffering from TB, related to TB, the questions that will be asked your focus should be on TB I know you have taken care of HIV positive people but today we are focusing on TB issues are we together.

Participants:	Yes

Interviewer2:	From there are their questions from the video, ok when you have questions you can ask us to pause, we will discuss if we feel that question is something we will discuss later we will keep that question for that time. Are we together?

Participants:	Yes

Interviewer 2:	I will ask to keep your phones on silent so that you can concentrate for a short time, if we keep this responding on time, it will not take longer as expected because we are already late. Any additional information doctor or Charity. That is that so

Interviewer 3:	We are all free that we can start the discussion.

Participants:	Yes

Interviewer 3:	As instructed earlier on when a question has been put forward you are not supposed to use your names, so we have given you, identified you with numbers so when started responding you are going to say am number 2, 1 then you start giving your views. That is how we are going to do the discussion no mentioning names but just the views and the number. For a start I would like to know what you have understood from the information that you have been given from the beginning of this meeting. We are free to use both Chichewa and English.

Interviewer 2:	But you know most of the medical terms to explain them in Chichewa sometimes it becomes hard, but we are all Malawians feel free to express in either Chichewa or English but audible enough that another person who has not undergone through a medical training can understand to get information.

Interviewer 3:	As it has been said feel free what have you understood from the information that you have been given about this research?

Participant 8:	They have explained to us that in this research those that will be used are people who are HIV negative the aim is that they will help in developing a vaccine that can be more effective. And these HIV negative people they will be given bacteria’s like PCV vaccine not so, which are weakened bacteria’s, the people will be monitored about what happens when they have been given the vaccine, the aim is to help in future. But if it happens that one of the participants fall sick they will be assisted accordingly.

Interviewer3:	Other views, different view?

Participant 3:	In my understanding on what has been explained, you have explained that there is a study that will be conducted that will support to identify a vaccine faster because for vaccine to be developed and approved it takes a long time, but the study that will be conducted will be conducted for the reason for vaccines not to take very long, vaccine can be developed within a short period of time. In addition to that I have understood that this research is better because it is being conducted right here in Malawi the vaccine can be used here in Malawi there will be effectiveness, or we can say there will be more benefits because it will be done on native people.

Interviewer3:	Ok, thank you.

Participant 1:	I have heard that we have been called to give our views on a research study that is to be conducted so we will be asked questions to answer by giving our views in agreeing or disagreeing to the plans of the study. We are free to say whether the study should be conducted or with what I see with these points I feel this study should not be conducted.

Participant 2:	Likewise, as my friends have spoken, we have heard that those people who participate in the study will be followed up, in case something has happened there will be nurses and doctors closely monitoring to them, what is happening after receiving the vaccine.

Participant 4:	I have learnt that those who are participating in the study are the age of 18 above.

Interviewe3:	Any additional views about the information that you have been given from this discussion.

Participant 6:	This study will also involve those people who are HIV positive yes that’s what I have.

Interviewer3:	Can you explain on that.

Participant 6:	Yes, the main aim is to find out on immunization that can be given, that can be developed in order to prevent people from getting TB infection.

Interviewer1:	Yes, so very good understanding from everyone as we are going deeper into the discussion the main thing we should remember is, we are seeking your views on one topic and the topic is it acceptable and what are the issues if we could do a Tuberculosis challenge in HIV negative people. So, as we go into the discussion let’s not shift our focus to any other population groups but one group HIV negative and what we will be discussing are what are the issues, can we really do a Tuberculosis challenge in HIV negative people. I like not I like, am excited about the understanding that you have as we go into the discussion let’s remember our focus should be HIV negative, can we do a Tuberculosis challenge.

Interviewer3:	So, from that do you feel the information that you have been given about this study is important or useful.

Interviewer 1:	What Charity is asking is from the discussion that we have had from the video from the beginning everything what did you find most useful to the discussion according to you?

Participant2:	According to me it’s because this study has highlighted what will be happening from the beginning up to the end I think you have explained how important the study is and the video has also highlighted that this study can help quick development of vaccine rather than waiting for many years to come up with a vaccine.

Interviewer3:	So, what are the important areas that have been mentioned.

Participant 5:	Mostly what I have seen is that people, participants when they are coming to join the study, they will be well informed and there they will be guaranteed that everyone will be taken care of properly everyone, the people will join with confidence without, if any problem arise, we will be taken care of.

Interviewer3:	Others, another important area.

Participant 4	I think it’s the time frame, time frame to develop vaccine from taking years and to the place that we are doing it at a faster rate whereby we do the vaccine that’s the only matching thing but the rest of the things the others were doing the same.

Interviewer 3:	In terms of the time frame, you are comparing to.

Participant 4:	The way they were developing vaccine in the past, it was taking many years to develop vaccine, when a person falls sick, they collect samples go to laboratory they analyze they collect bacteria, they weaken it after weakening it then they inject in other people who are healthy later they get results, so it takes time.

Interviewer 3:	So, what will be the benefit of this that you can point out of this

Participant:	It is the turnaround time to develop vaccine

Participant:	I think that’s the long run it means

Interviewer 3:	Number?

Participant 2:	in the long run if it becomes successful am looking at, it means population at large will be protected I think.

Interviewer 3:	Other views? Others what are we think what information have we found to be most useful from what has been discussed from the beginning. You have mentioned about timeframe time will be reduced and there have been other two points that have been mentioned, the other point was. It will help large number of people in future. What are other useful important things that you feel.

Participant 3:	Mien I see it as an advantage as this study will be done in Africa mostly in Malawi so as the vaccine will be developed if all works we will adopt our own locally making vaccine locally than getting them from outside as most vaccines are done

Interviewer 3:	Ok, other different views

Participant 8:	I agree with participant number 3 but if it is, if the study will happen here in Malawi meaning that effectiveness for the Malawians will be high.

Interviewer 3:	What unanswered questions do you have about this CHIM model study? The questions that you feel they haven’t been answered or haven’t been provided with enough information.

Participant 6:	I just want to find out more because there are, as the theme it is we are talking of HIV negative people but in terms of TB most of those people are those who are HIV positive and with this leaflet, it’s talking about the people who are HIV positive, so I don’t know are you going to tackle or to take the samples to get much more data on these HIV positives that you can rate the response that you are seeking from.

Interviewer 3:	Ok, so in your comment there has come a question where you are asking that in the paper there is somewhere it’s mentioning about HIV positive people, as it has been said earlier we have to put our focus that we are focusing on TB, on people who are HIV negative at the back of our mind we must focus on people who are HIV negative. The information that has been given on the paper is just general across what has been done or what will be done like in future but for this discussion our focus should be towards HIV negative people so in this case am asking what your unanswered questions regarding the information are that you have been given regarding people who are HIV negative to be given bacteria to them. Do you have any unanswered questions that you have been provided from here?

Participant 4:	Yes, we have unanswered question like for example for some of us we received PCG at childhood, I mean when we were little babies so now you are introducing PCG at the age of 18 or above. How does that correlate in terms of your results findings?

Interviewer 2:	Ok, I think that will be answered in the course of our discussion.

Interviewer 3:	So, in terms of unanswered questions do anyone have other questions that have not been answered or that have not been addressed or we should move on.

All	we should move on.

Interviewer 3:	I would like to know what additional information do you need to have for you to make an informed decision, a decision about joining this CHIM model studies or not, do you need any additional information that you feel has to be added to what has been provided to you?

Participant 1:	Yes, I just, I would like to know more on the participants that will be like volunteers, are these people going to have a certain place maybe when they have received the PCG vaccine. Are they going to kept somewhere maybe outside the general population or maybe they will be just left to go into the general population considering that maybe they might be infectious to others? That’s what I would want to know.

Interviewer 2:	Can I take that now, so they will be kept, our plan is that they will be in the general population because they will not be able to transfer it to other people. It is like when we get vaccine when our children have gotten the PCG vaccine I don’t think we isolate them because they will not actively transfer it to other people unless in this case where we are giving them the bacteria which is a different case. But for safety reasons all our participants who participate in our CHIM studies we request them to go to Grace Bandawe where they stay for three nights so it’s not necessarily isolating them that they will be infectious or they will transfer it to other people but we want to make sure that they are safe because the first three days is when they can, there is a little chance that they can get sick. So, because people are coming from different areas someone from Chilomoni, and other areas so because we usually encrypt 4-8 because they are many if one or two fall sick during the night we won’t be able to assist them on time. The only time we keep them at Grace Bandawe is just for safety reasons otherwise it’s not that they will be able to transfer the bacteria or the vaccine to other people. PCG vaccine is safe it has already been tested and it has passed all the requirements.

Participant 2:	Ok, we you have said that you are targeting adults those who are HIV negative. Are you still going to conduct HIV test to confirm that they are HIV negative?

Interviewer 2:	Yes, if the arm of the study says we are recruiting HIV negative people you know people will come that they are HIV negative, HIV positive until you confirm so for us to confirm one of the when we are screening them on the day of screening one of the test that we do is the an HIV test and this is consented we consent them can we do an HIV test so when you are consenting to a study it ,means you are allowing all study procedures that they can be done on you, one we do HIV test.

Participant:	You will be using Gene Expert or

Interviewer2:	We are using Oral Quick, we use Oral Quick if Oral Quick is negative it’s negative, if it’s positive we take it as screening test we confirm with unigold what is the other test.

Participant:	Determine

Interviewer2:	We call a trained counsellor to come and confirm that, and then the person goes to

Participant:	Because my fear is sometimes when you use Oral Quick, Determine, Unigold to those who are HIV positive, and they are taking medication their viral load suppressed sometimes they come negative but it’s only Gene expert that can tell us whether it’s truly negative or positive so that’s where my fear is

Interviewer 2:	I think we will look into that.

Participant 5:	I would like to know when people have joined you have done the study will the people be given feedback on the results or after doing when the results are successful, they will see the thing has been established doing this this.

Interviewer2:	We do, as it was explained before consenting how will the results be shared so they are shared differently like at research dissemination that we do people share results, by publishing a paper you share results. But usually, we call people who are willing and who are within you know when we have approached different people, we found some have moved to other districts but for those who are within we call them for feedback that is after everything. You know when a study is done today it’s not like the results will be in tomorrow there are samples to be tested and it can take up to two years before we have the final results.

Interviewer 3:	Ok, does anyone have any additional information that you feel it has to be provided for you to make an informed decision or opinion to proceed with CHIM model study?

Participant 4:	The information that you need to add is a person is ok being fine and you have given vaccine leading into a disease are you going to start him TB treatment because we are focusing on TB, are you going to initiate on TB treatment, if it proceeds to get the disease. So, if you are starting the TB treatment is there any compensation because this one play that I was ok, I was fine I was not getting a disease, but you injected me up to the case that I get the disease am on medication how are you going to do about it.

Interviewer3:	That will be (not clear background noise from outside) Alright thank you know I think we can move on, now we want to go to our next category of questions. We want to look at the risks and benefits that can be there in conducting this CHIM model study. What do you think are the benefits of conducting a TB CHIM in a healthy volunteer like the HIV negative person what can be the benefit?

Participant 1:	I just think maybe it will give us a true reflection of the study or the vaccine, because someone who is HIV negative is considered as at a lower risk of developing some other diseases so maybe this one will give us a true reflection unlike someone who is already at risk of complicated diseases.

Interviewer 3:	Here the true reflection you are referring to?

Participant1:	The results of the study

Interviewer 3:	The results of the study other views, what benefit is there to do this research or there are no benefits?

Participant2:	I think the benefits as there as participant number 1 has highlighted, because when we are doing with those who are HIV negative these are the people who are not at risk of developing TB so we trying to test at these people to see how their immunity is going to fight. But if we go those who are HIV positive sometimes, they are already at risk so maybe the results will have some bias maybe we will have bias results that maybe these they are already maybe at risk that’s why the vaccine did not work. So, when you do with those adults who are healthy it will give the true reflection.

Interviewer 3:	Ok, another benefit that can be there let’s look at the benefits in broader terms we should look at all areas what can be the benefits that can be there in doing a TB CHIM in health adults or in HIV negative volunteers.

Participants:	No response

Interviewer 3:	Is that the only benefit that can be there that there will be a true reflection how things are or there are other benefits.

Participant 8:	Another benefit can be that the vaccine will be developed in Africa in Malawi let’s just imagine the people to benefit are Malawians that can be like a benefit.

Interviewer 3:	That side there is any other benefits or there no benefit at all

Participant	Of course I was looking at (TB ithe campaign) end TB campaign I was thinking that it will help in Africa if this vaccine id developed in Africa it will help in the reduction of TB infection yes.

Interviewer 2:	Before we get to vaccine development we should look at your relative, we are saying we are doing the foundation, a good house need to have a good foundation we should look at, we want to try the study if your relative want to join the study you have been working in TB ward and you have seen how people struggle with TB so we want to put intentionally we want to put a TB bacteria to see how their immunity responds this person is healthy he is negative he does not have any other issues. What do you feel can be the benefit, you should reflect with the video we watched the introduction what are the benefits of CHIM? Here we are looking at an individual who does not have TB we give them TB bacteria but in a form of vaccine.

Participant:	I think it’s more or less the same as stated earlier on that once this vaccine is introduced to this individual the healthy individual it will help to activate the antibodies that were already introduced while he was young because we still received the BCG1 when we were young. So, it will like activate those ant bodies which will at the end of the day increase the immunity of this individual

Interviewer 2:	Others what are we saying?

Interviewer 3:	Others what are we saying, or we should ask it in Chichewa, what benefit do you think could be there in doing a Pneumococcal CHIM in HIV negative people or healthy volunteers. The benefit that can be there

Participant:	I don’t know maybe am overthinking or it is going that way

Interviewer 3:	It’s number.

Participant 5:	I feel like HIV positive people in future will also benefit because we will already be aware of this, that if we are giving these people there is a risk or a benefit if it is ok we will not doubt when giving them that they are going to face different problems.

Interviewer 3:	Let’s go, that side another benefit that will be there, or this is the only benefit that can be there. There is other views?

Interviewer 2:	I have a question considering that we want to do this research, do you feel there are risks/barriers that can hinder us from doing the research?

Participants:	Yes, barriers are many

Interviewer 3:	We can start on the barriers / risks.

Participants laughed.

Interviewer 3:	What are the barriers. Tell us what the risks or barriers are conducting the research we are interested, what are the barriers/ risks to do the research.

Participant 8:	There are myths in society that with technology like today I saw one they say they want to depopulate through these vaccines this will make people not to accept the vaccine, they might say maybe this vaccine is for that. Even Covid vaccine most people did not get it thus literate as well as illiterate ones because of these myths that they want to depopulate.

Interviewer 3:	Let’s go with views, it will be one after the other.

Participant 6:	With this vaccine that will be developed if someone has been injected with vaccine and it happens if someone gets sick unfortunately the person dies as she has highlighted that so many myths are there. So it’s one way as of today I was also reading and I even circulated on our group I said there is Virus X which is to be developed and that virus is there to depopulate around 6 billion souls because they have put souls with this if a lot read this information concerning that Virus X is coming in and then you inject this person in the name of the research that you want to develop vaccine for TB, and then unfortunately that person dies they will consider it as one way of depopulation.

Interviewer 2:	They will say it has come.

Participant 6:	Oh yes that virus has come maybe in a form of TB research and then a person has gone. Yes, so you should expect those.

Interviewer 2:	You agreed, another risk we will hear from everyone you all agreed. Speak louder for the recorder.

Participant 4:	Aa, you are saying that you are giving the vaccine like in bacteria to a person who is HIV negative, yet we know already that those who are HIV negative their immunity is already boosted and we are living in the pandemic whereby TB is not new. So, with an exposure that we have because we are exposed to TB, and we never know everyone is coughing	it is likely we will develop Tuberculosis. Like this other time, they were doing tool test to those people who are HIV negative, but it was found out that it was coming positive the tool test so like on the tool test it is found positive for a person who is HIV negative and does not have Tuberculosis symptoms. And you are taking TB bacteria’s giving to that person what exact outcome that you get from there will the result be positive or negative	with respect that it’s an HIV negative, what will be the result? It is likely that the result will be questionable because it will be negative, you did not react, unless you are taking another population HIV positive, this one HIV negative then you give the people you want to come out with the results how it reacts in HIV negative how it reacts when it’s in HIV positive how it reacts that will at least make sense but in HIV negative of course the results will be all the same, it’s not reacting in the long run the vaccine is not reacting in people with HIV yet you were bias because you considered only those who are negative whom immunity has developed they come from a community which is exposed to TB bacteria if they have been exposed they will develop TB immunity .

Interviewer 3:	Thank you, other views.

Participant 2:	I think there will be fear of unknown this is just a new thing that is coming so I think that will meet resistance to find the participants because people will be afraid as my friend have already said that a lot has been spoken, not concerning this study but introduction of virus and what. maybe if this is the one of the viruses that are coming to finish us.

Interviewer 2:	To kill us, these are by fellow Malawians.

Participant 2:	There will be that fear.

Interviewer 2:	Fear and myth have been mentioned several times, most people have mentioned myth which is true what are other things.

Participant 3:	Another challenge that you might face is when someone is given the vaccine and he actively develop the TB after hearing the length of taking medication as it is on the ground 6 months, compliance I think it might be a challenge that someone can say I was just ok and this issue has come in then you are telling me I should take medication for 6 months this is just too much.

Interviewer2:	Yes, other what are saying we should finish. You have something to say, you want to say something.

Participant 4:	Yes, I want to say something another issue will be you considering for those who had TB before they are another population that are HIV negative but they had TB before, they are coming into your study ok fine am HIV negative recruit me give me the vaccine then I think the outcome of it I don’t think it will be desirable.

Interviewer 2:	I think that will be looked into on our exclusion. So, you have mentioned of barriers that can hinder us, if it were you, you are the research team you are in our shoes, how should we explain the risks to someone who want to join the study, on the risks for one to understand, what’s the best way to explain to them the risks.

Participant 3:	To my understand if am the one who want to join the study, I will sit down with them despite that we are targeting HIV negative people, we will also exclude non communicable diseases people. What we are going to do is our aim is not to kill but our aim is to introduce something that was already introduced in you when you were young, now this time it’s coming while you are an adult it is something that you received so now we will give you because we want to develop vaccine through that thing that is not a strange thing it is a thing that everyone when he was young received and I can go to an extent that the scar that we have is what	we will be giving. Such an explanation I feel some people can accept it.

Interviewer 3:	You have given a point that we can give an example of another vaccine which was received earlier on top of that you mentioned of communicable diseases. I just want you to explain in terms of the non -communicable diseases you are looking at then on what angle.

Participant 3:	Ok, what I meant on non-communicable diseases was referring to people with Diabetes that their immunity is challenged, a person like that one despite that he is HIV negative I will tell him not to take part because we will be introducing a bacteria because his body is weaker because has challenge of Diabetes for this study they should not participate the ones we want people who are HIV negative who don’t have other health problems like Diabetes, BP and other things. They should not find a case on the existing problem.

Interviewer3:	Other views, how should we communicate to people about risks for them to understand.

Participant 6:	When they come the first thing I believe we have to ask if he has suffered from other diseases like TB, we have to ask if he has ever suffered from TB or not, if he has Asthma problem they are not allowed to participate because Asthma is already respiratory condition now we are introducing TB If anything happens it will be like you are the one who have hurt the person with the condition. They have to be informed that they are getting the vaccine, there are two things that can happen one can get sick with the vaccine or can be ok after receiving the vaccine those are desired results that we need to expect.

Interviewer 2:	About the risks we should not tell them, a person wants to join the study should we tell him about the risks or not?

Participant:	That’s why I have said after giving the vaccine one can get sick right because we have given the vaccine it will activate if he already has that condition it will be activated, he can get sick or it can happen that he is ok without any problem.

Interviewer 2:	How can we tell someone about the risks if you are the research nurse you want to communicate to someone who want to join the study. How best can we communicate?

Participant 8:	We should start by explaining the benefits, explain the risks we should also explain in case something happens we should tell them the support that they can get, I feel we can do that.

Interviewer 3:	There you seemed you wanted to say something.

Participant:	Yes, I think the way she has explained when a person has come so the best thing is to explain the benefits of the study, that we are conducting this study the benefits we want to monitor how the body reacts if we introduce attenuated bacteria of course that can cause Tuberculosis which will give us true reflection on vaccine if it works best. Maybe if things don’t go as we planned drugs are there and we will support you.

Interviewer 3:	Other views

Participant 1:	My views are not different from my friends the main thing we have to tell the person that there are other risks that can you can face if you join the study but for me the main thing when we want to conduct a research we want to recruit people I can give assurance as mentioned by my friends that we will give you contacts that when you face any problem even when at home you can call us, the person can trust you that even if I face a problem I might call the people and they help me they will see that we are not running away we will be with them throughout.

Interviewer 3:	Ok, thank you anything. Let us come to our last section. I would like to know if your close relative is willing to join a study like this how can you feel about it. If someone close to you tells you that he has joined a study like that, how would you feel. Here we want to hear individually how you would feel. We have discussed about benefits and risks now we want personal feel how would you feel.

Participant 4:	I don’t think I can feel something one everyone has a choice if he wants to join a study it’s his choice, someone’s choice cannot make me to feel bad no, if they have explained to him that this study does this and there is informed consent and he has accepted to participate in the study, he is my relative it is fine.

Participant:	Like wise

Participant2:	Number

Participant 2:	Of course, we have been struggling with different diseases and to get where we are it’s because of vaccines it means some have volunteered themselves to participate so it is just the same you will support if things go well so I will just encourage people.

Participant 1:	Maybe it will be based on the relationship if the person is very close relative, I will tell him to wait take a while other should start joining.

Other participants were laughing.

Interviewer 3:	Why

Participant 1:	You have to wait in future after seeing how things are going you can join, because we are dealing with Tuberculosis, TB is not a simple disease, it is a serious disease that if something happens he will give me the burden to look after him, he has to take medication for 6 months if he was working he will be weak other things will be on stand still. I cannot just agree for him to join I will tell him to wait for other to join later after how you see how things are going you can join after two to three weeks.

Interviewer 3:	Ok, that’s her view, other views we want to hear everyone’s view.

Participant 2:	Your sister has come saying she has joined already how will you feel, what will you do.

Participant:	Mmhi all laughed

Participant 8:	Personally, I don’t think I can be worried of anything because BCG vaccine the way I look at it, I take it as the same that we were taking our children before being discharged from the hospital children were getting and children were growing without any problem. What more an adult person who has developed immunity in a country where Tuberculosis we are facing it. IN future like me I can see that in our family we contributed to development of this vaccine, we will be proud that that in our family we participated in development of this, it will not be hard that my relative has participated even myself I can participate maybe because of age as I have not reached 18 years

Participants:	All laughed

Participant 3:	I cannot deny him if the owner has confirmed to participate, to my experience with TB treatment my concern is when things go contrary like as actively, he has suffered from TB that is my main concern but participating that everything goes well I don’t any issues with that but in the case that one has active TB now. I have seen how TB treatment goes so	participating in the study I don’t have issues with it the main issue that I can see it can bring a burden like in our family is during the time of treatment which is a 50 50 chance. The issue is that it is something that can happen or not for example the usual vaccines children receive we stay 2 -3 days sleepless nights it is 50 50 chance so for my relative to say I will participate in the research I will tell him that if everything goes well will thank God but if things go contrary I will enlighten to him if things go on the contrary TB is curable but what is the most important thing is compliance to treatment so that you don’t have recurrence of the disease but that’s the thing

Interviewer 3:	This side

Participant 5:	The same with me, that will be a personal decision he has seen the benefits and how things will go for me as a person that my relative still more I will have a certain burden I will be busy to check how he is feeling, does he has fever it will be like all of us we are going together he is the one who has joined but I will be the one who will be restless with what he has done I will be together with him.

Interviewer 3:	How will you support him on that.

Participant:	When a problem arises, we will rush to do the needful because we will know that these are signs of TB we should start the process to have diagnosis, treatment and see how treatment will go. If everything goes well, we will just be thankful.

Interviewer 3:	That side other views, a lot has been said concerns raised, those in support have supported everyone has expressed his side. Let’s come to comments that you can have concerned the whole idea of this CHIM model.

Participant 6:	In case this person has developed the disease while while monitoring after receiving the trial vaccine. What are the measures that you are going to take despite after being given the vaccine and you have observed him at Grace Bandawe as she has highlighted for three days, he has gone home maybe after a week he or she develop that disease or signs and symptoms of TB and then comes here the diagnosis is confirmed that it is TB how are you going assist or how are you going to as (background noise)?

Interviewer 2:	So everyone who is in the study they sign a consent they are under us whatever disease that you have, you have diarrhea, you have malaria, cholera you are under the study care. Our plan we admit patients at Mwaiwathu but we have never admitted anyone in the past, we have had several CHIM studies but every study has insurance so in case you are sick, in case you are injured in case anything happens you are supposed to claim and it’s a lot of money no one has ever claimed that because we have never messed up.

Participant 6:	Because I was looking at the mount of money which is supposed to be compensated to this person its 3000 US Dollars

Interviewer 2:	Oh, the 3000 Dollars you mentioned was.

Participant 6:	No and according to the studies when you are doing research, and the participant has developed anything which is in line with the research that you are conducting it’s supposed to be compensated with 3000 if it’s not 4000 dollars so I was looking at that.

Interviewer 2	WE have to confirm with our documents if that is in line with MLW because we have guidelines, so it’s uniform so don’t have that information now, but I have to ask the PI or anyone on how compensation goes in case of what you are saying

Interviewer3:	Other comments

Participant:	Mien was a question because on exclusive criteria there is HIV positive and those who are below 18, we did not ask why.

Interviewer 2:	That why HIV negative or

Participant:	Why are we excluding HIV positive and the below 18

Interviewer2:	We feel that when you are starting something new, this is a new concept though we have already had other CHIM this is not the first time, I also have a question I hope I will not preempt it because my question was I wanted to ask that we have had other CHIM we told you, we already did PCV 13, now we are doing with HIV negative but different bacteria’s. So, I wanted to ask you later how you can compare with the research that we are doing on TB. But answering your question we want HIV negative because we know that this group has immunity as the doctor mentioned it, and they are easy people to deal with but when you are HIV positive if you are not maybe taking drugs properly you have not started ART immunity is susceptible to anything so we cannot put this burden again on those people. There is the other study where we are giving participants the same bacteria that causes Pneumonia, we are giving to HIV positive but it’s not everyone also where there is HIV positive, we also have inclusion and exclusion criteria.

Participant:	So that was going in my head, but I was thinking that you have excluded them because of issues of immunity like someone mentioned about Diabetes they have also compromised immunity, Asthmatic people those maybe they have taken chemotherapy they have also, all those that you did not mention.

Interviewer2:	Ok today we were not able to preempt our exclusion and inclusion criteria we did not want to give you that information, we just wanted to look in general a negative person who is healthy but in our exclusion criteria that is also looked into. In all our studies which we have done we have never included those who are, some of the exclusion criteria are those who are HIV positive, those with BP, those with history of TB, anyone who is taking any immunity medication, those who are pregnant those with an under five children at home, so it is not just in general HIV negative come here no there are also other exclusion criteria’s in that HIV negative. Why we are looking at 18, 18 is the legal age in Malawi you can consent in Malawi and make a decision on your own and we also feel that this age from 15 -45 or 40 that we are talking about are very active than someone who is over 50 they will be like saying my back even though they are HIV negative so we are looking at heathy and active population are we together?

Participants:	All yes

INterviewer2:	So, my question is we have told you that we are doing research, this is not the first time, we conducted in HIV negative, we were recruiting 18 - 30 years of age, now we are doing HIV negative, others but we are giving them different bacteria we are also doing HIV positive this is the experience that we are having. The way you have understood with the introduction how do you think we can compare with this new one. How can you differentiate this to someone with how you have understood, with your little knowledge that we have shared. How would you compare?

Participant 3:	In your explanation you have explained that the studies that now you are conducting those who are not HIV reactive you are using, you will use different bacteria like this you said Pneumococcal one t while this one you are using the bacillus is it that.

Interviewer2:	hey are different bacteria’s but the procedure and everything is the same.

Participant:	The procedure is the same

Interviewer 2:	Yes, but how would you compare them.

Participant 3:	Ok to my understanding it’s like on the treatment when things goes bad on Pneumonia treatment or the one that you are dealing with now it has a short time treatment while the one that you want to start dealing with in TB if things goes in a negative way it means you will have a long time that you stay with this person on the treatment side yes.

Interviewer 3:	Another way how this can be compared to the other CHIM studies?

Participant	No response

Participant 2	There are no other views. So having looked at the risks and benefits what are your thoughts about the study, general thoughts weigh the benefits and the risks if you combine to weigh them what are your thoughts.

Participant 6:	I think the study is timing in the sense that we have been trying for the past years that, if possible, TB has to be reduced so with the coming in of this study I think it will map the way forward of how TB can be reduced and also the management itself.

Interviewer 2:	Doctor what are your thoughts.

Participant:	The study can proceed because this is at the beginning it’s different from when a study is in progress then you are accessing the risk and benefits that becomes challenging but now you can proceed. Where you have identified the risks you can turn them into positives, saying what should we do when we are on this issue if a person can develop how are we going to do. How do we convince people that the study is not like how it used to be in the past how are we going to deal with myths people have concerning studies, vaccines if you are dealing with these things, you will be on the positive side the study can proceed and people can accept it?

Interviewer 2:	You can start with your number and then your thoughts.

Participant 8:	I think if the vaccine will be proven to be effective, I believe that we will have a TB free generation which will be, which will help the country in terms of finances and other things.

Participant 2:	I think it’s a good start because it’s now high time we have been having this song of TB, so if the study will be successful, I think it will help so yes.

Participant 1:	I also agree that we should try this study and see the outcomes and just make sure that within the study we are monitoring the participants to see if we are to continue with the study or discontinue according to the risks that will be in our participants.

Participant 3:	To my look of things, it’s a nice study you can go on, I believe that people will welcome it positively and mostly as your targeted population the youth, it will be a nice experience that’s what I can say mostly.

Participant 5:	It should continue let’s do, it looks well covered on the information that has been provided. The main thing we must follow what has been discussed everything will go as planned it will be successful.

Interviewer 3:	Thank you very much for views, your participation and your presence here, we don’t take it for granted we really appreciate the information that you have provided will be very useful for the study in the process of collecting information to come up with a report to present in future about CHIM model studies to be conducted in Malawi, this is the end of the discussion. Let me apologize for the hiccup we had at the beginning that has delayed us to take us long but thank you very much for your presence here.

Participants:	About TB it’s a nice topic to my experience I have been in Tb ward since 2019 what I noticed mostly is that HIV negative patients are the ones who die mostly than HIV positive people.

Even chemotherapy HIV positives responds better.

Interviewer2:	You know I think in that ARV there is something which they don’t tell us.

Participant:	There is something
